# “She must have been sleeping around”…: Contextual interpretations of cervical cancer and views regarding HPV vaccination for adolescents in selected communities in Ibadan, Nigeria

**DOI:** 10.1371/journal.pone.0203950

**Published:** 2018-09-17

**Authors:** Folusho Balogun, Olayemi Omotade

**Affiliations:** 1 Institute of Child Health, College of Medicine, University of Ibadan, Ibadan, Nigeria; 2 University College Hospital, Ibadan, Nigeria; University of the Witwatersrand, SOUTH AFRICA

## Abstract

**Background:**

Human *Papilloma virus* (HPV) vaccines for adolescents are pivotal in the control of cervical cancer, the commonest women specific malignancy in sub-Saharan Africa. Knowledge about cervical cancer have been reported to be low in Africa but expressed acceptability for HPV vaccines have been high. The reason for this mismatch is not clear. An understanding of the interpretation of cervical cancer and views about HPV vaccine are important as they can affect actual decision making regarding adolescents’ uptake of HPV vaccine. This study explored contextual interpretations of stakeholders regarding cervical cancer and HPV vaccines for adolescents in five selected communities in Ibadan, Nigeria.

**Methods:**

Qualitative data were collected through twenty focus group discussions among parents of adolescents, religious and traditional leaders, school teachers and adolescents; and four key informant interviews with the identified traditional healers in the selected communities. Constant comparison analysis was done after transcription.

**Results:**

Almost every group had at least one person who had seen a cervical cancer patient. Cervical cancer was widely viewed as being caused by promiscuity of women while older participants believed that the alteration of lifestyle by civilization was a major contributory factor. There was also a general notion that it was due to a curse. The role of HPV was generally not known. Most participants were favourable towards HPV immunization for adolescents but traditional healers and some religious leaders were not. The high cost of the vaccines and possibility of side effects where the main concerns about the introduction of HPV vaccine. Decision to take the vaccine rest with the fathers whose views were heavily influenced by traditional and religious leaders.

**Conclusions:**

Awareness about cervical cancer may not be as low as earlier reported and there were many misconceptions about cervical cancer in the study communities. It is important to address these misconceptions to ensure successful introduction of HPV vaccine for adolescents in future.

## Introduction

Cervical cancer is the third commonest female specific malignancy globally and about eighty percent of new cases are found in developing countries [1],[2]. It is responsible for the highest number of female deaths in developing countries [2]. Cervical cancer has also been identified as the largest cause of the potential years of life lost in the developing world [3]. The incidence of cervical cancer in Nigeria is 250/100,000 women [4] and it was the most commonly occurring cancer in Nigerian women in 2013, commoner than breast cancer and it caused more deaths than breast cancer in the same year [2]. It will be expected that with the high incidence of cervical cancer in sub-Saharan Africa, the people should be familiar with the condition. However, despite the high incidence rate, literature have consistently shown that knowledge about cervical cancer is generally low in sub-Saharan Africa, with some studies actually reporting zero knowledge about the condition [5], [6], [7].

The herald of the human Papilloma virus (HPV) vaccines have given a positive outlook to cervical cancer prevention aside from the screening using Pap smear. The vaccines are recommended to be given to adolescents between the ages of nine and thirteen years and a number of African countries have started immunizing their adolescents [8], [9], [10], while some are at the pilot stage of the vaccine introduction. The HPV vaccines have been shown to be safe [11], have high efficacy [12], [13] and are cost effective [14], [15]. The acceptability of the HPV vaccine have also been high in the African countries that have introduced the vaccines despite the low knowledge of cervical cancer that have been reported in these countries [16], [17].

The reasons for the disparity in the knowledge of cervical cancer, the occurrence of the disease and acceptability of HPV vaccine in Africa should be investigated if programmes for prevention of cervical cancer are to succeed in the continent. There is a possibility that the language used to connote cervical cancer is not familiar to the people and so they do not really have cervical cancer in mind whenever they participate in cervical cancer research. It is also likely that the presentation of cervical cancer during research is different from the context in which cervical cancer is known, resulting in the non-linkage of what is being presented with cervical cancer. These first two possibilities are fundamental issues which are required to be adjusted by pretesting research instruments [18]. The other possibility is that the disease may be shrouded in secrecy as it affects the reproductive tract which is normally treated as a private thing generally. This can affect the extent of awareness and familiarity with the disease in the general population as only close contacts of patients with cervical cancer are likely to be familiar with it.

Understanding the contextual interpretations of cervical cancer in Africa can help in illuminating the African outlook regarding the disease and this can give insights into the various ways in which researches regarding cervical cancer should be structured in order to induce correct and appropriate responses. It is also important to understand the cultural perspective of the diseases, its prevention and management. These can affect the way interventions targeted at cervical cancer control (which includes HPV vaccines) are viewed and provide possible ways of promoting these interventions, at the same time preventing their rejection. This study explored the understanding regarding cervical cancer and the different interpretations of the disease among stakeholders in selected communities in Ibadan, Nigeria. It also described the views about HPV vaccines for adolescents in the prevention of cervical cancer.

## Methodology

### Study area

This study was carried out in the five communities which made up the ward 3 of Ibadan North Local Government Area. These communities are: Òjé, Yemetu, Òkè Aremo, Beere and Aláàádorin. The traditional system of government was still operational in these communities and the traditional chiefs were the custodian of the people’s culture. This made the system highly influential and respected. The general chief of the city of Ibadan was the Olúbàdàn who was assisted by the Baálès at the community level and Móngàjís at compound (made up of households) level. The communities were interlinked and had a mixture of both urban and semi-urban settlements which is one of the reasons for selecting the study area. This provided a good mixture of the socioeconomic classes that made the findings from this study to be as varied as possible.

The study communities were very religious and the religious leaders were highly respected, which make them weld a lot of influence on what were viewed as norms by the community members. Christianity and Islam were the dominant religions but some traditionalists who worship the traditional gods still exist. Overall, there were more churches (with more varied denominations) compared to mosques. There was a Ward Development Committee which was in charge of the political and traditional affairs of the communities made up of religious leaders, women leaders, opinion leaders and the representatives of the general chief at the city level which were the Móngàjís and Baálès. There were traditional healers in the community who were providing traditional medical care for different health conditions including cervical cancer management.

The human Papilloma virus vaccine was already licensed in Nigeria at the time of this study but was not yet introduced into the routine immunization programme.

### Study design and study population

This was an exploratory qualitative study in which twenty focus group discussions (FGDs) were conducted among stakeholders who will be involved in decision making about adolescents’ uptake of HPV vaccine. Four key informant interviews (KII) were also conducted with each identified traditional healers in the communities. The FGDs were carried out among parents of adolescents (one each for males younger and older than forty five years; one each for females younger and older than forty five years) and school teachers teaching adolescents (two). The other FGDs were among in-school adolescents (two each for junior and senior secondary school girls; two each for junior and senior secondary school boys), out of school adolescents (one each for males and females), traditional (one) and religious leaders (two for Christian and one for Islamic religion). One of the traditional healers was a traditional religious leader.

### Data collection instruments

Data were collected using interview guides with both English and Yoruba versions which were developed based on the study objectives and information from the literature. The questions covered were knowledge and prevention of cervical cancer, knowledge about HPV and HPV vaccine and concerns about administering HPV vaccine to adolescents. They were also asked to suggest how HPV vaccination for adolescents should be organized. Face validation of the interview guides were carried out among selected participants in a community that was not among the study communities. The version of the interview guide used depended on the makeup of each discussion group. For example, almost all the interviews conducted in the schools were conducted in English as majority of the participants understood and could spoke the language fluently. During each session, the symptomatology of cervical cancer was presented to the discussants to ensure that they had a clear understanding of what cervical cancer is. Pictures showing the female genital system were also used to illustrate the location of the cervix in the human body. Similarly, when none of the discussants/interviewees knew about HPV and HPV vaccines, a brief description was given before the discussion/interview continued.

### Data collection procedures

Three research assistants conducted the interviews: a facilitator, a note taker and a time keeper. Data collection took place between February and March, 2017. All interviews were conducted in Yoruba language except those carried out in the schools where a mixture of English and Yoruba were used. All interviews were also recorded using three digital recorders simultaneously.

The FGDs with parents, traditional leaders and the KII of two of the traditional healers took place at a central community hall, while the remaining traditional healers were interviewed in their homes. These study participants were identified with the assistance of the Ward Development Committee chairman. Those among in-school adolescents and school teachers were conducted in the school premises, while that of the out of school adolescents were held at a vocational training centre. Participants in the schools and vocational training centre were identified through the assistance of the various heads of the institutions with the aim of getting adolescents that can express themselves in group discussion with confidence and no inhibitions. The religious leaders were interviewed at their places of worship. They were approached directly by the research team.

Each FGD group comprised minimum of four and maximum of twelve participants with the sessions lasting for 45 to 75 minutes. The participants were assured of the confidentiality of their responses and each was identified with a code so that names were not mentioned during the interviews. The sociodemographic characteristics of the respondents were obtained and they were introduced to the sessions by a brief description of the symptomatology of cervical cancer. This was then followed by the questions in the interview guides.

### Data analysis

The digitally recorded interviews were transcribed verbatim and the Yoruba interviews were then translated to English. The transcripts were supplemented with the hand written notes. Data analysis was carried out using thematic analysis method [19]. The data was read critically and divided into smaller segments and codes (data driven) were manually developed using inductive approach independently by three members of the research team (including one who was not present during the interviews) to ensure the codes were validated. Themes were created by axial coding which was used to group the codes into categories with similar characteristics. These themes were then reviewed to ensure that they were robust enough to stand alone and also to make sure there were no repetitions [20]. Selective coding was eventually used to integrate the themes through critical reviews within each discussion and across the others, and relationships were determined [20]. The quotations that best illustrate each theme were then selected.

### Trustworthiness of the research

Trustworthiness was ensured in this research by following the four criteria for assessing trustworthiness for qualitative inquiries, which are: credibility, transferability, dependability and confirmability [21],[22].

#### Credibility

The two researchers were familiar with the participants’ culture and this reflected in the study design and the scope of the participants included in the study. The participants covered a wide range of stakeholders who would be involved in influencing decisions about prevention of cervical cancer. This helped in triangulating the data sources and the data formed a good representation of the community members, providing a variety of perspectives which were as close to reality as possible. Participation was also voluntary and the participants were assured of the confidentiality of their responses. All of them gave informed consent/assent before they were included in the study and they participated actively in the research. Three members of the research team also coded the data independently and the codes were later reconciled the codes.

#### Transferability

Detailed description of the study area and study participants have been given which can assist in the understanding of the research finding. This can also assist in the replication of the study in a similar setting.

#### Dependability

Detailed report of the study design and the steps in its implementation have been stated, including the steps taken in the analysis of the data. This can enable reproducibility in future.

#### Confirmability

This was ensured through the data collection from a broad base of stakeholders. All the discussions and interviews were moderated by trained research assistants and not by any of the authors so as to avoid undue bias due their medical background. The avoidance of the use of the word “cervical cancer” but the use of its symptomatology to present the disease to the participants was also a step to ensure the participants understand what was being discussed.

### Ethical considerations

The study was approved by the University of Ibadan/ University College Hospital Ethics Review Committee. The communities were accessed through the Community Development Committee Chairman who introduced the research team to the other community leaders. Written informed consent was taken from all adult participants including parents/guardians of adolescents who were less than 18 years of age. The consent forms for the parents of the adolescents were given to the selected adolescents in school/training centre a week before the interviews were conducted. Only those whose parents/guardian gave consent were included in the study. The young adolescents also gave assent. The names of the participants were not mentioned during the interviews, rather, they were identified using codes. The participants were assured of the confidentiality of their responses.

## Results

### The study participants

Twenty focus group discussions and four key informant interviews were conducted. In all, there were 190 participants and 110 were males. All the traditional leaders were males and almost all the religious leaders, except the second group of Christian religious leaders that had two women. Table 1 shows the other details of the study participants.

**Table 1:** Sociodemographic characteristics of the study participants.

Focus Group discussion group	Number of particip-ants	Mean age/ Age(years)	Religion		Range of years practice/ teaching experience
			Christian	Islam	
Senior girls private school	9	15.6	6	3	
Senior girls public school	9	15.7	2	7	
Senior boys private school	10	16.6	6	4	
Senior boys public school	9	15.9	7	2	
Junior girls private school	10	12.4	8	2	
Junior girls public school	10	15.2	2	7	
Junior boys private school	9	13.4	7	2	
Junior boys public school	10	14.4	4	6	
Out of school adolescents female	10	17.8	7	3	
Out of school adolescents male	6	17.8	6	2	
Christian religious leaders I	9	39.6	9	0	
Christian religious leaders II	11	56.5	11	0	
Islamic religious leaders	11	72.3	0	11	
Mothers of adolescents <45years	10	37.7	4	6	
Mothers of adolescents ≥45years	12	59.7	7	5	
Fathers of adolescents <45years	10	37.8	4	6	
Fathers of adolescents ≥45years	6	66.5	2	4	
Community leaders	4	75.0	0	4	
Female teachers of adolescents	10	35.3	8	2	2–12
Male teachers of adolescents	11	41.5	8	3	3–18
Traditional healer I^a^	1	78	-	-	53
Traditional healer II	1	40	-	1	20
Traditional healer III	1	52	-	1	25
Traditional healer IV	1	45	-	1	18

^a^ He was also a traditionalist.

### Themes arising from the data

The following themes were identified:

- Awareness about cervical cancer
- Perceived causes of cervical cancer
- Perceived ways of prevention of cervical cancer
- Views about HPV vaccine for adolescents in the prevention of cervical cancer
- Suggested structure of a successful HPV vaccination programme for adolescents

### Awareness about cervical cancer

After the presentation of the symptomatology of cervical cancer, almost all the groups had at least one person who had seen a patient with cervical cancer before. More women had heard about cervical cancer compared with men. The commonest places where contact with cervical cancer information occurred were health facilities and church outreach programmes. These were supported by the following quotes:

“I heard it in church …somebody testified… she started bleeding from the vagina and it was giving that foul odour”

FGD, Male teachers of adolescents, participant 1

“I heard it on the television and in a hospital”

FGD, Mothers of adolescent ≥45years, participant 8

### Perceived causes of cervical cancer

The perceived causes of cervical cancer was diverse but there were some common grounds across the groups. Promiscuity of women was mentioned in all the groups as being the cause of cervical cancer. This was usually mentioned first and with a lot of emphasis all through the discussions. This view is reflected in the following quotes:

“…if a woman have sex anyhow with different men, it can cause cancer. If you sleep with 5 men that is 5 different diseases, 8 men means 8 different diseases. So if a woman sleeps around she can have cervical cancer”

FGD, Christian religious leader, participant 7

“it is caused by sexual intercourse that is not protected and dirty”

FGD, Mothers of adolescents <45years, participant 5

“…when a woman is promiscuous, …there is no way she will not have the cancer, so that is what I think can cause the cervical cancer”

FGD, Fathers of adolescents ≥45 years, participant 4

Only the females said promiscuous men can make their partners to have cervical cancer without being probed.

Some other perceived causes was also related to how sexual intercourse take place among couples as shown in the quotes below.

“…so after 8 days of giving birth they will start having sex… Having sex that early after birth can cause this disease…”

FGD, Mothers of Adolescents ≥45years, participant 3

However, this view was not shared by younger mothers but some young fathers of adolescents shared this view as shown in the quote below.

“It is when you are always having sex that you can have such disease”

FGD, Fathers of adolescents <45years, participant 2

The traditional healers and the older men believed that women are reservoirs of diseases as they can have infections that can be concealed for a long time, but men usually manifest diseases earlier. Generally, the older participants believe cervical cancer is a new disease that came about as a result of civilization and change in life style as depicted by the following quotes.

“All these things are as a result of civilization and this is what is causing all these diseases. We didn’t know about all these cancers growing up. It is possible there was cancer then but it wasn’t this common… But these days it is so common…”

FGD, Mothers of adolescents ≥45years, participant 6

“Those days, these diseases were not rampant, or common, we wonder at anyone who gets it”

KII, Traditional healer I and traditional worshiper

This belief also reflected in the conflicting perceived causes of cervical cancer among the older and the younger participants as shown in the following quotes about what was appropriate to use during menstruation and condom use.

“…by not using cloth during menstruation and even if you have to use cloth, you should keep it clean and if it is pad you are using you should change morning and night”

FGD, Junior girls public school, participant 9

“In our own days, there was nothing like pad, we would use cloth… But nowadays… if you see the pad some of them use- they will throw their pads in very open places where people will see and curse them, how will such a person not have all these diseases”

FGD, Mothers of adolescents ≥45years, participant 9

“Cervical cancer is caused by condom use…you know it is similar to inserting a stick in the vagina…there will be friction”

FGD, Islamic religious leaders, participant 6

“…it is because of this thing that there is condom. I don’t know of any other drug that can be used to prevent it but I know the use of condom is important”

FGD, Mothers of adolescents <45years, participant 5

Poor hygiene and westernized diets were mentioned by some participants as causes of cervical cancermainly by females (older parents and students) and traditional, religious leaders and older fathers.

“when we eat too much of flesh it can cause cancer whether cervical or other ones,… all these- what do we call it? pizza…all these junk foods can cause cervical cancer…you will see some with too much salt… and the seasoning they put inside it.”

FGD, Male teachers of adolescents, participant 2

“Not all these things are transmitted through intercourse but by what we eat which is different from what God had placed in our blood”

KII, Traditional healer I

Other perceived causes were curses, eating of sweet things, eating unripe fruits, use of orthodox drugs, modern food seasoning, use of the mill for food processing and wearing of trousers by females.

Only one of the female teachers of adolescent reported that cervical cancer was caused by a sexually transmitted infection which may have a genetic link as shown in the quote below.

“I have read about it and I know that it is a sexually transmitted infection and I also look at it that some people, it is not as if they led a promiscuous life and they still came down with cervical cancer, I am also thinking maybe it is genetics, in some families, if someone has cancer, it is down the line in the generation, some other people may also have it…”

FGD, Female teachers of adolescents, participant 4

### Perceived ways of prevention of cervical cancer

The perceived ways of preventing cervical cancer was also as diverse as the perceived causes and in most instances, it was the reversal of the perceived causes. However, some perceived prevention methods were not exact opposite of what was stated as perceived cause. This was illustrated in the quote below.

“…by not sleeping around while menstruating”

FGD, Junior Girls Public school, participant 5

The use of traditional medicine was mentioned in every discussion group (except among Christian religious leaders) but there were many reports that these were no longer potent as it was in the olden days. The quote below represented this view.

“…in those days, our fathers knew all these things but these days it’s like all those things no longer work.”

FGD, Community Leaders, participant 3

The traditional healers and some participants however still believed that the traditional medicines were still effective in the management of cervical cancer. These are shown in the quotes below.

“A way I believe we can put an end to it is … If we can be drinking ‘Agunmu’… the incisions made today work well.”

KII, Traditional healer III

“You will see that an elder will put a ring on a boys toe… for some it will be ‘gbere’, for some it is ring and for some they will give them something to eat such that if he is close to contacting such disease, something will just happen that will prevent him from contacting it”.

FGD, Fathers of adolescents <45years, participant 5

However, none of the participants, except the traditional healers had ever seen a woman with cervical cancer cured by traditional medicine.

The Christian religious leaders also believe that it is prayers and fasting that that can prevent it as show in this quote

“We that are Christians, we fortify ourselves with vaccine of prayer because you don’t know when trouble will come, it is the prayer you have already said that will protect you”.

FGD, Christian Religious leaders I, participant 7

### Views about HPV vaccine for adolescents in the prevention of cervical cancer

Many participant believed the HPV vaccine was a good idea but there were some who were not willing to take the vaccine or allow their children or worshippers to take it. This latter view was expressed more by both the Christian and Islamic religious leaders as well as few of the other participants. Both views are illustrated by the quotes below.

“I can’t get vaccinated because most times when you get too much of anything it will have something it will damage in your body”

FGD, Senior Girls Public school, participant 3

“Because that thing kills people… it is better to prevent it with this vaccine than the one you will spend later, if you prevent it now, it will be better”.

FGD, Out of school adolescents male, participant 3

The main concerns about giving HPV vaccine to adolescents were the high cost, possible side effects, fear of injection, queries about the potency of the vaccine, some adolescents being sexually active before 10 years and the fear that children may be encouraged to be promiscuous. Some parents however believed that with good moral upbringing, the vaccines would not encourage adolescents to be promiscuous.

### Suggested structure for a successful HPV vaccination programme for adolescents

The suggested steps for a successful adolescent health programme include: increasing awareness about the vaccine, subsidising the cost of the vaccine, obtaining parental consent, males should be vaccinated as well, opinion leaders should be involved and adolescents in hard to reach places should be covered.

## Discussion

The contextual interpretation of cervical cancer varied among the different groups studied but there were some common grounds observed as well. Cervical cancer was generally believed to be caused by female promiscuity. The adolescents and many of the discussants accepted the idea of administration of HPV vaccine to adolescents to prevent the disease. Even though most of the perceived causes of cervical cancer were incorrect, it is important to look into the significance of these notions and the likely impact they may have.

The general notion that cervical cancer was caused by promiscuity of women can result in the stigmatisation of cervical cancer patients, and this can affect the quality of care and support they receive. This can negatively affect their quality of life and chance of survival. This is in addition to the already existing complex of late presentation, inadequate manpower and equipment to treat cervical cancer and lack of supportive network that are common in the study area. This implies that patients diagnosed with cervical cancer in this environment were likely to have had harrowing experiences. A similar view about cervical cancer was reported by women in a South African study which looked at the constructions of cervical screening and womb cancer among rural black women [23]. The female participants in this study said they would rather not report the symptoms should they have cervical cancer because the disease meant “disgrace” due to its association with promiscuity. This implies that there would be late presentation should the women develop cervical cancer and unnecessary mortalities. There is a need to investigate the overall experiences and quality of life of women who have had cervical cancer in these communities in order to offer them the appropriate support they require and re-orientate the populace.

The women were viewed as a reservoir of diseases and this may be as a result of men’s experiences with gonorrhea (a common sexually transmitted infection in the study area) which manifests earlier in males compared with females. However, this contradicted the report by Wood *et al* in which men were blamed for sexually transmitted diseases on one hand, while women were blamed for cervical cancer [23]. These viewpoints showed the poor knowledge of the epidemiology of cervical cancer, bringing to the fore the lack of understanding about the relationship between HPV and cervical cancer. It is therefore important that more efforts should be channeled towards improving the knowledge about the HPV and cervical cancer, as this will remove the stigmatisation associated with the disease, thereby improving the acceptability of the preventive measures for cervical cancer.

The view that cervical cancer is a “curse” can also contribute to stigmatisation of women with cervical cancer. The average African believe in the supernatural and has a strong belief that the supernatural influence daily living, including wellness and the occurrence of diseases [24, 25]. That is why Africans believe that ancestors and good spirits can protect against diseases while the anger of ancestors and witches cause diseases [26, 27]. Curses are believed to be as a result of wrong doing and one of the results are diseases, which in this case is cervical cancer. This belief can negatively affect the way women with cervical cancer are viewed in the real world and it can extend to cervical cancer preventive services.

The notion that immunizing adolescents with HPV vaccine would be a license to indulge in sexual activities is a concern that have been expressed by different stakeholders in different settings [28], [29]. This is as a result of the link between sexual activity and HPV transmission. In many communities in Nigeria, it is still desirable to be chaste before marriage which in part is as a result of cultural expectations and also due to religious inclinations. It is rare for Nigerian parents to talk openly about sex with their adolescents and this has a lot of negative implication on Nigerian adolescents’ sexual and reproductive health. Parents also believe that it is their responsibility to ensure that children, especially the girls remain chaste till they are married. Many of them however, do not appreciate the fact that their control over their children’s initiation of sex is highly limited with the exposure adolescents now have to information around them and the experimental nature of adolescents themselves. However, this concern about HPV vaccine and promiscuity among adolescents have been repeatedly shown to be unfounded in the literature as adolescents who had HPV vaccine were not found to be initiating sex earlier than those who did not receive the vaccine [30, 31]. Some adolescents who had the vaccine were even delaying sexual activities as some of them had sexuality education in the process of receiving the vaccine.

Despite this concern, many participants were in favour of adolescents getting immunized with HPV vaccine even when they were told that HPV was a sexually transmittable disease. This is in contrast to findings among South African and Ugandan population who frowned at the word “STI” and so HPV vaccine had to be introduced in these countries as a “cervical cancer vaccine” for it to be acceptable [32]. This may be an indication of cultural variability or differences in perception. It means the stakeholders in the studied communities were not shying away from the fact that they had adolescents who could be sexually active and therefore required protection. This was also expressed in the concern that some children become sexually active before their tenth birthday. Even though some of the participants were concerned that giving adolescents HPV vaccine could encourage promiscuity, there were also opinions that a good moral upbringing and sex education could forestall this.

There appears to be “genderlisation” of causes of illnesses including cancer as females were believed to be able to “hide things in their bodies” for a longer period without manifesting any symptom unlike men who will manifest the disease faster. This may be the reason why it was only women who mentioned the possibility of promiscuous men as the cause of cervical cancer without being probed. It may be that men were not tagged “promiscuous” even when they are because it is socially acceptable for men to have sex outside their intimate relationships in the study setting. A similar finding was reported in a South African study where the traditional healers believed that cervical cancer was as a result of women’s promiscuity, and it was when they were asked about the possibility of a faithful female having a promiscuous male partner did they agree that men can be the cause of the disease [23].

The association of contraceptive use with cervical cancer can impact negatively on the use of contraception in the study communities. The Islamic religious leaders’ associated condom use with cervical cancer and almost every group mentioned contraception as a cause of the disease because they have seen women bleeding due to complication of contraception use. It is also important to note that younger women believed the use of condom can actually protect against cervical cancer but the stigmatisation associated with possession of condom in the study area was seen as a barrier to condom use in cervical cancer prevention. The use of modern contraception have remained suboptimal in Nigeria and this stigmatisation may be one of the reasons responsible for the low uptake [33]. This wrong notion can be corrected with intensive and well targeted public health enlightenment.

The older participants and traditional healers generally believed that change of lifestyle as result of civilization was responsible for cervical cancer. This was reflected in their concern about the change in quality of agricultural products, food content and processing, dressing etc. These views were not shared by the younger age groups. Younger people are known to be favourably disposed to innovations and are usually more adventurous than older people. This can explain the differences in views. It is important to target older people for enlightenment about the correct cause of cervical cancer as they may find it difficult to allow their adolescents to uptake HPV vaccine if they don’t appreciate that HPV virus is responsible for almost all cases of cervical cancer. They are likely to view HPV vaccine as another product of civilization and reject it. The important positive roles that grandmothers play in the uptake of HPV vaccine by adolescents was reported in a study in Johannesburg [34], this shows that they can be useful in ensuring adolescents are immunized. Older women in South Africa have also been reported to have authority over younger women regarding decision making on health issues [23]. This further stresses the importance of enlightening the older people in the study environment to ensure they understand the importance of HPV vaccine for adolescents. Traditional healers are also very influential in Africa with a high patronage which is as high as 80% in some areas in South Africa [35]. Training them on the association between HPV, the vaccine and cervical cancer can help in increasing the acceptability of HPV vaccine for adolescents as they can be change agents in the circle of their influence.

The perceived causes of cervical cancer mentioned by the older mothers reflected how important personal hygiene (including menstrual hygiene) was to them. Mothers generally ensure good hygiene at home and poor hygiene have been associated with different diseases which may explain why they believed cervical cancer was one of the numerous outcomes of poor hygiene. However, their association of cervical cancer with the timing of commencement of sex after childbirth need to be addressed as there are long existing myths about sexual intercourse in nursing mothers which had no medical basis. It is however reassuring that the younger mothers did not have these views.

The trust in the efficacy of traditional medicine to cure cervical cancer in these communities appeared to have dwindled as the study participants mostly believed they were no longer potent due to changes in life style. None of the community members (apart from the traditional healers) reported knowing anyone who was treated by the traditional healers for cervical cancer. The traditional healers however still had confidence in their ability to cure cervical cancer. There might have been some unsatisfactory experiences that the community members have had with the services of the traditional healers for them to have this notion though this was not explored in this present study. The reports of earlier successes by the traditional healers in handling cervical cancer was difficult to verify as the traditional healers usually do not keep records and where they exist, details of what was seen and done were usually kept secret. This secrecy have hindered the development of African traditional medicine and it makes it difficult to modernise the activities of traditional healers. Collaboration among the traditional healers could help in developing this trade but this apparently was uncommon as only one of the traditional healers interviewed talked about having collaborations with other traditional healers.

Most of the perceived means of prevention of cervical cancer were targeted mainly at women which was expected, since women were mainly believed to be responsible for the disease, and the mostly reoccurring prevention method mentioned was women should avoid promiscuity. Many did not feel men too should avoid being promiscuous. This portrays the societal expectation that females were to be sexually faithful to their partners but this was not usually expected from men. It is interesting that condom use which was reported by the younger mothers as a possible prevention tool for HPV infection in order to prevent cervical cancer was believed to be a cause of cervical cancer by Islamic clerics. This could negatively affect the way condom use was being portrayed to their congregation members. This may actually be one of the reasons why contraceptive use has remained low in Nigeria over the years [33].

Refusal of some religious leaders to accept HPV vaccine may be detrimental to the success of the vaccine programme for adolescents. This is because of the influence they have on their followers who form a significant part of the population. This is of great concern as a Catholic Bishop’s banning of HPV vaccine for adolescents in Calgary, Canada resulted in low uptake of the vaccine in the past [31]. The roles of religious leaders in the polio vaccine boycott in northern Nigeria some years ago also showed the great influence that religious leaders have [36]. Some Christians are known to believe in faith healing, rejecting every form of orthodox and traditional medicine, whereas there are Christians who also believe in faith healing but use medicines as well. High parental religiosity have been linked with HPV vaccine acceptance in the past [31]. Although HPV was presented in this study as being sexually transmitted for research purpose, there is a need to desexualise HPV vaccine by emphasizing the other routes of transmission like instrumentation and mother to child routes. This may be useful in removing this misconception.

Concerns raised about HPV vaccine in this study were similar to earlier concerns raised in the literature and the most occurring was the cost of the vaccine. The high cost of HPV vaccine was one of the reasons why the Global Alliance for vaccines and immunization (GAVI) subsidised the cost of the vaccine for developing countries to be able to make it available for adolescents [9]. Presently, a huge success is being recorded in the few African countries who have started this programme (including South Africa, Uganda and Rwanda) [37, 38]. Cost had been a problem for HPV vaccine uptake even in advanced countries where people outside the catchment age have to pay out of pocket for the vaccine [39].

The other commonly occurring concern was the fear of side effects, even though literature had consistently shown that the side effects were mild and were mainly pain and swelling at the injection site which disappear within a few days [40, 41]. The frequent mention of side effect as a concern is likely connected to the participants’ previous experiences with other vaccines. These fears need to be addressed so that there will not be confusion with concurrent illnesses that may occur during vaccination as it happened earlier Japan where the HPV vaccine uptake is currently almost zero after an initial successful take off [42]. Awareness about HPV vaccine was generally low in this population and so the concern about side effects was not surprising. Although HPV vaccine is yet to be introduced into the routine immunization schedule in Nigeria, awareness should be flagged off in preparation for its introduction in order to have high uptake when the vaccine is eventually added to the routine schedule. This will also be of help to parents who can afford the vaccine as different brands are already available in the Nigerian market.

Only communities in Ibadan were studied and this could limit the generalisability of these research findings. This is also purely a qualitative study making it impossible to draw up associations or causalities, but salient views which can be very important in the design of a successful HPV vaccination programme for adolescents have been identified.

In conclusion, knowledge about cervical cancer in Nigeria may not be as low as earlier reported in Nigeria as evidenced the increase in the number of people who reported having seen people with cervical cancer when they were presented with the symptomatology of cervical cancer in the present study. Cervical cancer research in Nigeria and Africa as a whole may give different outcomes if this approach is used, and this brings to fore the need to investigate local names cervical cancer is called in this region. The contextual interpretation of cervical cancer was highly varied in this study population and reflected the cultural values of the community about women being faithful to their partners. It also showed in their perceptions about what constituted unhealthy living which were believed to be responsible for cervical cancer. These interpretations are significant and it is important to address them to avoid stigmatisation of cervical cancer, improve disease detection, presentation and promote uptake of prevention procedures. HPV vaccine was seen as a good idea by many who were also willing to encourage its uptake, but health promotion intervention is required for the groups who were not favourable towards the vaccine.

## Supporting information

S1 Interview Guides Study instruments. (DOCX)

S1 CaCx data Study transcripts. (ZIP)

## References

[1] van Bogaert L. Are the currently existing anti-human papillomavirus vaccines appropriate for the developing world? Annals of medical and health sciences research. 2013;3(3):306–12. Epub 2013/10/12. 10.4103/2141-9248.117924 . 24116304 PMC3793430

[2] Fitzmaurice C, Dicker D, Pain A, Hamavid H, Moradi-Lakeh M, MacIntyre MF, et al The Global Burden of Cancer 2013. JAMA oncology. 2015;1(4):505–27. Epub 2015/07/17. 10.1001/jamaoncol.2015.0735 . 26181261 PMC4500822

[3] Cunningham MS, Skrastins E, Fitzpatrick R, Jindal P, Oneko O, Yeates K, et al Cervical cancer screening and HPV vaccine acceptability among rural and urban women in Kilimanjaro Region, Tanzania. BMJ Open. 2015;5(3):e005828 10.1136/bmjopen-2014-005828 25757944 PMC4360576

[4] Ahmed SA, Sabitu K, Idris SH, A R. Knowledge, attitude and practice of cervical cancer screening among market women in Zaria, Nigeria Nigerian medical journal: journal of the Nigeria Medical Association. 2013;54:316–9. 24403709 10.4103/0300-1652.122337 PMC3883231

[5] Marlow LA, Waller J, Wardle J. Public awareness that HPV is a risk factor for cervical cancer. British journal of cancer. 2007;97(5):691–4. Epub 2007/08/10. 10.1038/sj.bjc.6603927 . 17687335 PMC2360359

[6] Poole DN, Tracy JK, Levitz L, Rochas M, Sangare K, Yekta S, et al A cross-sectional study to assess HPV knowledge and HPV vaccine acceptability in Mali. PloS one. 2013;8(2):e56402 Epub 2013/02/23. 10.1371/journal.pone.0056402 . 23431375 PMC3576405

[7] Perlman S, Wamai RG, Bain PA, Welty T, Welty E, Ogembo JG. Knowledge and awareness of HPV vaccine and acceptability to vaccinate in sub-Saharan Africa: a systematic review. PloS one. 2014;9(3):e90912 Epub 2014/03/13. 10.1371/journal.pone.0090912 . 24618636 PMC3949716

[8] Turiho AK, Okello ES, Muhwezi WW, Harvey S, Byakika-Kibwika P, Meya D, et al Effect of school-based human papillomavirus (hpv) vaccination on adolescent girls' knowledge and acceptability of the HPV vaccine in Ibanda District in Uganda. African journal of reproductive health. 2014;18(4):45–53. Epub 2015/04/10. . 25854092 PMC4536292

[9] Hanson CM, Eckert L, Bloem P, Cernuschi T. Gavi HPV Programs: Application to Implementation. Vaccines. 2015;3(2):408–19. Epub 2015/09/08. 10.3390/vaccines3020408 . 26343194 PMC4494350

[10] Mbulawa ZZA, van Schalkwyk C, Hu NC, Meiring TL, Barnabas S, Dabee S, et al High human papillomavirus (HPV) prevalence in South African adolescents and young women encourages expanded HPV vaccination campaigns. PloS one. 2018;13(1):e0190166 Epub 2018/01/03. 10.1371/journal.pone.0190166 . 29293566 PMC5749739

[11] Lu B, Kumar A, Castellsague X, Giuliano AR. Efficacy and safety of prophylactic vaccines against cervical HPV infection and diseases among women: a systematic review & meta-analysis. BMC infectious diseases. 2011;11:13 Epub 2011/01/14. 10.1186/1471-2334-11-13 . 21226933 PMC3034689

[12] Crowe E, Pandeya N, Brotherton JM, Dobson AJ, Kisely S, Lambert SB, et al Effectiveness of quadrivalent human papillomavirus vaccine for the prevention of cervical abnormalities: case-control study nested within a population based screening programme in Australia. BMJ (Clinical research ed). 2014;348:g1458 Epub 2014/03/07. 10.1136/bmj.g1458 . 24594809 PMC3942076

[13] Cutts F, Franceschi S, Goldie S, Castellsague X, Sanjose Sd, Garnett G, et al Human papillomavirus and HPV vaccines: a review. Bulletin of the World Health Organization. 2007;85:719–26. 10.2471/BLT.06.038414 18026629 PMC2636411

[14] Goldie SJ, O'Shea M, Campos NG, Diaz M, Sweet S, Kim SY. Health and economic outcomes of HPV 16,18 vaccination in 72 GAVI-eligible countries. Vaccine. 2008;26(32):4080–93. Epub 2008/06/14. 10.1016/j.vaccine.2008.04.053 . 18550229

[15] Drolet M, Boily MC, Van de Velde N, Franco EL, Brisson M. Vaccinating Girls and Boys with Different Human Papillomavirus Vaccines: Can It Optimise Population-Level Effectiveness? PloS one. 2013;8(6):e67072 Epub 2013/07/11. 10.1371/journal.pone.0067072 . 23840589 PMC3694081

[16] Becker-Dreps S, Otieno WA, Brewer NT, Agot K, Smith JS. HPV vaccine acceptability among Kenyan women. Vaccine. 2010;28(31):4864–7. Epub 2010/06/23. 10.1016/j.vaccine.2010.05.034 . 20566394 PMC2906622

[17] Dairo MD, Adeleke MO, Salawu AT, Adewole AD. Parental support for human papilloma virus vaccination by adolescents in Ibadan North Local Government Area, Ibadan, Nigeria. International journal of adolescent medicine and health. 2016 Epub 2016/12/04. 10.1515/ijamh-2016-0034 . 27914213

[18] Czaja R. Questionnaire pretesting comes of age. Marketing Bulletin-Department of Marketing Massey University. 1998;9:52–66.

[19] Leech N. L., O AJ. Qualitative Data Analysis: A Compendium of Techniques and a Framework for Selection for School Psychology Research and Beyond. School Psychology Quarterly. 2008;23(4):587–604.

[20] Green J, T N. Qualitative Methods for Health research. 3rd ed: SAGE Publications; 2014.

[21] Guba EG. Criteria for assessing the trustworthiness of Naturalistic Inquiries. Educational Communication and Technology. 1981;29(2):75–91.

[22] Shenton AK. Strategies for ensuring trustworthiness in qualitative research projects Education for Information. 2004;22:63–75.

[23] Wood K, Jewkes R, Abrahams N. Cleaning the womb: Constructions of cervical screening and womb cancer among rural Black women in South Africa. Social Science & Medicine. 1997;45(2):283–94. 10.1016/S0277-9536(96)00344-9. 9225415

[24] Gouteux JP. [The supernatural, health and community action in Black Africa]. Bulletin de la Societe de pathologie exotique (1990). 1992;85(3):256–60. Epub 1992/01/01. 1422280

[25] Jegede A. S. The Yoruba cultural construction of health and illnesss. Nordic Journal of African Studies. 2002;11(3):322–35.

[26] Pilkington H, Mayombo J, Aubouy N, Deloron P. Malaria, from natural to supernatural: a qualitative study of mothers' reactions to fever (Dienga, Gabon). J Epidemiol Community Health. 2004;58(10):826–30. Epub 2004/09/15. 10.1136/jech.2003.016089 . 15365107 PMC1763324

[27] Tenkorang EY, Gyimah SO, Maticka-Tyndale E, Adjei J. Superstition, witchcraft and HIV prevention in sub-Saharan Africa: the case of Ghana. Culture, health & sexuality. 2011;13(9):1001–14. Epub 2011/07/01. 10.1080/13691058.2011.592218 . 21714753

[28] Penta MA, Baban A. Dangerous agent or saviour? HPV vaccine representations on online discussion forums in Romania. International journal of behavioral medicine. 2014;21(1):20–8. Epub 2013/09/24. 10.1007/s12529-013-9340-z . 24057409

[29] Mupandawana ET, Cross R. Attitudes towards human papillomavirus vaccination among African parents in a city in the north of England: a qualitative study. Reproductive health. 2016;13(1):97 Epub 2016/08/24. 10.1186/s12978-016-0209-x . 27549328 PMC4994299

[30] Monk BJ, Wiley DJ. Will widespread human papillomavirus prophylactic vaccination change sexual practices of adolescent and young adult women in America? Obstetrics and gynecology. 2006;108(2):420–4. Epub 2006/08/02. 10.1097/01.AOG.0000228509.11502.d2 . 16880314

[31] Grimes RM, Benjamins LJ, Williams KL. Counseling about the HPV vaccine: desexualize, educate, and advocate. Journal of pediatric and adolescent gynecology. 2013;26(4):243–8. Epub 2013/09/21. . 24049807 10.1016/j.jpag.2013.04.002

[32] Harries J, Moodley J, Barone MA, Mall S, Sinanovic E. Preparing for HPV vaccination in South Africa: Key challenges and opinions. Vaccine. 2009;27(1):38–44. 10.1016/j.vaccine.2008.10.033. 18977271

[33] NDHS. Nigeria Demographic and Health Survey 2013. International NPCNNaI, editor. Abuja, Nigeria and Rockville, Maryland, USA: NCF and ICF International; 2014.

[34] Francis SA, Battle-Fisher M, Liverpool J, Hipple L, Mosavel M, Soogun S, et al A qualitative analysis of South African women's knowledge, attitudes, and beliefs about HPV and cervical cancer prevention, vaccine awareness and acceptance, and maternal-child communication about sexual health. Vaccine. 2011;29(47):8760–5. 10.1016/j.vaccine.2011.07.116. 21855591

[35] Nelson JA, Francis SA, Liverpool J, Soogun S, Mofammere N. Healers in a non-traditional role; a focus group study of Sangoma’s knowledge of and attitudes to cervical cancer prevention and screening in Johannesburg, South Africa. Sexual & Reproductive Healthcare. 2010;1(4):195–6. 10.1016/j.srhc.2010.07.004. 21122621

[36] Jegede AS. What led to the Nigerian boycott of the polio vaccination campaign? PLoS medicine. 2007;4(3):e73 Epub 2007/03/29. 10.1371/journal.pmed.0040073 . 17388657 PMC1831725

[37] Moodley I, Tathiah N, Mubaiwa V, Denny L. High uptake of Gardasil vaccine among 9–12-year-old schoolgirls participating in an HPV vaccination demonstration project in KwaZulu-Natal, South Africa. South African medical journal = Suid-Afrikaanse tydskrif vir geneeskunde. 2013;103(5):318–21. Epub 2013/08/24. . 23971122 10.7196/samj.6414

[38] Gatera M, Bhatt S, Ngabo F, Utamuliza M, Sibomana H, Karema C, et al Successive introduction of four new vaccines in Rwanda: High coverage and rapid scale up of Rwanda's expanded immunization program from 2009 to 2013. Vaccine. 2016;34(29):3420–6. Epub 2015/12/26. 10.1016/j.vaccine.2015.11.076 . 26704259

[39] Mortensen GL. Drivers and barriers to acceptance of human-papillomavirus vaccination among young women: a qualitative and quantitative study. BMC public health. 2010;10:68 Epub 2010/02/16. 10.1186/1471-2458-10-68 . 20152055 PMC2829006

[40] Giacomet V, Penagini F, Trabattoni D, Vigano A, Rainone V, Bernazzani G, et al Safety and immunogenicity of a quadrivalent human papillomavirus vaccine in HIV-infected and HIV-negative adolescents and young adults. Vaccine. 2014;32(43):5657–61. Epub 2014/08/26. 10.1016/j.vaccine.2014.08.011 . 25149430

[41] Goncalves AK, Cobucci RN, Rodrigues HM, de Melo AG, Giraldo PC. Safety, tolerability and side effects of human papillomavirus vaccines: a systematic quantitative review. The Brazilian journal of infectious diseases: an official publication of the Brazilian Society of Infectious Diseases. 2014;18(6):651–9. Epub 2014/05/02. 10.1016/j.bjid.2014.02.005 . 24780368 PMC9425215

[42] Tanaka Y, Ueda Y, Kimura T. Struggles within Japan's national HPV vaccination: A proposal for future strategy. Human vaccines & immunotherapeutics. 2017;13(5):1167–8. 10.1080/21645515.2016.1267085 28059676 PMC5443375

