# Supplementary material for: “She must have been sleeping around”…: Contextual interpretations of cervical cancer and views regarding HPV vaccination for adolescents in selected communities in Ibadan, Nigeria
Source: PLoS One. 2018 Sep 17;13(9):e0203950. doi: 10.1371/journal.pone.0203950 (PMC6141096; doi:10.1371/journal.pone.0203950)
Supplement: S1 Interview Guides — (DOCX) [file pone.0203950.s001.docx]

**INTERVIEW GUIDE (English version)**

(For parents of adolescents, religious and traditional leaders, school teachers and adolescents)

The purpose of this discussion is to explore your understanding and knowledge of a particular condition. Please feel free to express your views. The information you provide will be kept confidential.

1. Have you heard about or seen a woman who is 40 years and above starts to have abnormal bleeding from the vaginal (in between menstruation, after sexual intercourse etc.), foul smelling vaginal discharge, waist/ back pain with or without weight loss and may leads to death when discovered late. (Show them the diagram of the female reproductive system and show the location of the cervix where the disease starts from)
2. What did you hear about it?
3. Do you know anyone who has had it before?
4. In your locality, what are the known causes of this condition?
5. What do you personally know about it? (probe for their source of information)
6. Describe what you think can cause this condition?
7. Please, describe how it can be prevented.
8. Have you heard about human papilloma virus before? If yes, what do you know about the virus? (If no, a brief description of the virus will be given by the facilitator of the focus group discussion).
9. Have you heard about human papilloma virus vaccine before? If yes, what do you know about the vaccine? (If no, a brief introduction of the vaccine will be given by the facilitator of the focus group discussion).
10. What are the advantages of administering HPV vaccine to adolescents?
11. What are the disadvantages of administering HPV vaccine to adolescents?
12. What are your concerns/ fears about administering HPV vaccine to adolescents? (probe for any concern/fear)
13. Why would you allow/not allow your adolescents to receive HPV vaccine? (probe for as many reasons as possible)

OR (for adolescents)

Why will you agree/not agree to receive HPV vaccine?

1. If HPV vaccine is to be made routine for adolescents in Nigeria, what will be the best approach to ensure the adolescents get immunized? (Probe for preferences for community based, school based and facility based programme, parental consent, ensuring vaccine completion etc.).
2. Knowing that HPV is sexually transmitted, why will you allow your adolescents to be given the vaccine?

**IWE IFOROWANILENUWO (Eda ti ede Yoruba)**

(Fun awon obi manjesin, olori esin ati ibile, oluko ile iwe ati awon manjesin)

Idi ijiroro yi ni wipe a fe mo imo ati oye yin nipa aisan kan pato. E le so bi o ti ri lokan yin ifokanbale. Gbogbo ohun ti e ba so ni ibi yi, eti keta koni bawa gbo o.

1. Se e ti gbo tabi ri arabinrin ti ojo ori re to bi ogoji odun tabi ju bee lo ti o n ri eje lati oju ara re lai se nkan osu tabi leyin ibalopo, ti eda oloorun buruku n jade ni oju ara re, eyin tabi ibadi le maa dun kio si maa ru, o si le ja si iku ti ko ba tete gba itoju (Fi aworan eya ara ti o n sise fun ibimo ti obinrin han won ki o si fi enu ona ile omo ti aisan ti a n so yi ti maa n bere)
2. Kinni awon ohun ti e gbo nipa re?
3. Se e mo enikeni ti o ti ni iru aisan yi ri?
4. Ni adugbo yin, kinni awon ohun ti a mo ti o n fi okun fa iru aisan yi?
5. Kinni e mo nipa aisan yi fun rara yin (bere nipa ibi ti won ti gbo ohun ti won mo)
6. Se alaye ohun ti iwo lero pe o n fa aisan yi?
7. E jowo, e salaye bi a se le dena aisan yi.
8. Se e ti gbo nipa okoro ti a n pe ni “human papilloma virus (HPV)” ri? Ti o ba beeni, kini e mo nipa kokoro naa? (bi beeko, alakoso iforowanilenu wo yio se apejuwe ranpe bi kokoro naa se je).
9. Se e ti gbo nipa abere ajesara ti HPV ri? Ti o ba je beeni, kinniemo nipa re? (Bi beeko, alakoso iforowanilenuwo yio so oro ni soki nipa abere naa)
10. Kinni awon anfani ti o wa ninu ki a fun awon manjesin ni abeere yi?
11. Kinni awon aleebu ti o le wa bi a ba fun awon manjesin ni abeere yi?
12. Kinni ohun eru tabi ipaya ti o le de ba yin ti a ba fun awon manjesin ni abeere yi? (Beere fun ohun eru tabi ipaya)
13. Kinni o maa je ki e gba/ ma gba abeere yi fun awon manjesin yin(Beere fun gbogbo idi ti o le fa eleyi ti won ba mu)

TABI (fun awon manjesin)

Kinni awon idi ti wa fi gba/ma gba lati gba abeere HPV?

1. Bi abeere HPV ba di okan lara abeere ajesara ti a n gba dede fun awon manjesin ni orile ede Naijiria, ona wo ni a le gbe gba ti o dara julo lati ri wipe awon manjesin gba abeere yi? (Beere fun bi won ba fe ki won maa gba ni adugbo, ni ile iwe tabi ni ile iwosan, bi yio ba pan dandan ki awon obi koko fowosi iwe ki awon manjesin too gba, bi a se le ri daju pe won gba pe abbl)
2. Bi e se mop e ara ibalopo ni a ti maa n ko kokoro HPV, kinni idi ti o fi maa je ki manjesin re gba abeere yi?

**INTERVIEW GUIDE: Traditional healers (English version)**

1. If a woman of forty year and above starts bleeding from the vagina, different from her menstruation or has bleeding from the vagina following sexual intercourse and sometimes, the bleeding may be foul smelling, what do you call such condition? (Show them the diagram of the female reproductive system highlighting the location of the cervix where the disease is located).
2. What do you think is responsible for this condition? (Probe for whether it is infectious, hereditary etc.)
3. What do the Yoruba people believe is responsible for this?
4. Can you share your experiences about women that you have managed for this condition before?
5. Tell us how it can be prevented.
6. Describe how such women are managed.
7. There is a vaccine that can be used to prevent this condition. It will cost N21,000 to immunize an adolescent fully with the 3 required doses. It is to be given to adolescents before the onset of sexual activity for maximum efficacy. What do you think about this vaccine for adolescents? (Probe for concerns about the vaccine, acceptability etc.)
8. If the government want to start an immunization programme to get this vaccine for adolescents, how do you think the program should be organized?

**IWE IFOROWANILENUWO TI AWON ONISEGUN IBILE (Eda ti ede Yoruba)**

1. Ti obinrin ti o to ojo ori bi ogoji soke ba ri eje lai sepe o n se nkan osu tabi ri eje bi o ba ni ibalopo, ti omi po mo eje ba bere si jade loju ara pelu oorun, oruko wo ni a ma n pe iru nkan be? (fi aworan ile omo obirin han won ki o si fi enu ona ile omo ti aisan yi maa n wa han won).
2. Kinni e gbagbo pe o n fa nkan yi? (Bere boya o le ran elomiran lati ara eniti o ni, se aisan idile ni abbl.)
3. Kinni Yoruba gbagbo pe o n fa nkan yi?
4. E se alaye iriri ti e ti ni pelu itoju awon obirin melo ti o ni awon nkan ti a ka sile yi.
5. Ona wo ni a fi le dena re?
6. Bawo ni a se n toju awon obirin ti o ni iru nkan yi?
7. Abeere ajesara kan wa ti o le dena aisan yi. Yio na yin ni N21,000 lati gba meta abeere yi fun manjesin kan ki o to gba pe. Awon manjesin ni o ye ki o gba ki won to bere ibalopo ki o ba le sise daradara. Kini ero yin nipa gbigba abere yi fun awon manjesin? (Bere nipa ohun ti o je ipaya fun won, boya won yio gba wole abbl.)
8. Bi awon ijoba ba fe se eto igbekale abeere yi fun awon manjesin wa, bawo ni e se lero pe o ye ki won se eto naa?
