# Supplementary material for: “She must have been sleeping around”…: Contextual interpretations of cervical cancer and views regarding HPV vaccination for adolescents in selected communities in Ibadan, Nigeria
Source: PLoS One. 2018 Sep 17;13(9):e0203950. doi: 10.1371/journal.pone.0203950 (PMC6141096; doi:10.1371/journal.pone.0203950)
Supplement: S1 CaCx data — (ZIP) [file pone.0203950.s002.zip › FGD SENIOR GIRLS PRIVATE.docx]

**TYPE OF PARTICIPANTS: SENIOR GIRLS PRIVATE**

**TYPE OF INTERVIEW: FOCUS GROUP DISCUSSION**

M: Good morning, as I said earlier, My name is ………… and we are here to discuss issues relating cervical cancer with you, we want to know what you know, we want to know what you know about HPV and some other issues , the conversation will not take a long time, I want us to cooperate with us and say things the way it is on our mind, don’t let anyone intimidate you, I already took your permission to record this conversation, do I have your permission to record the interview

All: Yes

M: Thank you very much, so the first question I will ask is, have you heard about cervical cancer, have you ever heard about it, and if you want to say anything, you say your number first, number 3, this is what I know, number 2, this is what I know and we cannot all talk at the same time , you won’t be able to pick who is talking, you will tell us your number , then you will tell us what you have to say, have you heard about cervical cancer before? Has anyone heard about it, the recorder cannot pick your nods, so you have to express it, has anyone heard about cervical cancer, who is saying no, number

P5: number 5

M: you have never heard about cervical cancer, is there any other person that wants to comment?

P3: number 3, no

M: no 3, no, number?

P7: no

P8: no

P2: no

M: you have never heard about it

P4: no

M: no 4, no, so most of us, we have not all heard about it, we have not all heard about it

All: yes

M: all of us

All: yes

M: how about cancer, have you heard about cancer? If you are talking make sure you mention your number, have you heard about cancer

P1: yes,

M: speak up so this thing can capture your voice, where did you hear about it, what kind of cancer? Are you shy, so speak up, what have you heard about cancer?

P1: breast cancer

M: so have you seen someone who has it,

P1: no

M: but you have heard about it, where did you hear about it?

P1: in my neighbourhood

M: your neighbour

P1: in my neighbourhood

M: speak up, so you heard it in your neighbourhood, has any other person heard about cancer

All: yes

M: where did you hear about it?

P3: in my grandma’s house,

M: your grandma’s house, what kind of cancer

P3: breast cancer

M: breast cancer too, any other person? Number, that’s number 7, where did you hear about it

P7: my brother

M: what kind of cancer?

P7: breast

M: okay, he told you about breast cancer, any other person?, is that all we have, we have all mentioned breast cancer? But do we know that cancer can actually come from anywhere, we can have eyes cancer, brain cancer, nose cancer, any kind of cancer, throat, skin, anything, cancer can come from anywhere, cancer is an abnormal growth, but that is not why we are here, we came because of a particular type of cancer, that is cervical cancer, this cervical cancer is peculiar to women and why did I say its peculiar to women, its because it’s women that have cervix, the cervix is the entrance to the womb, do men have wombs, it is a peculiar thing to women, women have cervix and they are the ones who can give birth, is that not

All: yes

M: or have you heard a boy giving birth before

All: no

M: women have it, and how do we know that this cancer is there, if you see a woman that is over 40 years old, not a young woman, a woman that is over 40, at least 40, may be 40 to 100 or to 150 or to 200, if that woman is bleeding in between menses, you know when a woman is 40, she is almost nearing menopause and she starts seeing blood when she is not menstruating, it is not menstruation but she sees blood, the blood does not really stop like that, It is almost like every day , not only that, she may be losing weight and the blood that is coming out, the blood has a foul smell by the time you pass, except if the person uses a very strong, what is it called, odour neutralizer, when the person passes, you can perceive the odour, it is a strong smelling blood and the person is getting leaner by the day, the person may not even be leaner, the person will have back pain, and what else, the person is over 40, have you heard anyone with that kind of symptoms, this person is bleeding, it is not menstruation, it has a foul smell and she has back pain with it, have we heard something like that, probably we have heard someone or somebody gave a testimony in our church, or someone gave a testimony in our neighbourhood, or we know someone that knows our mum and told her about it, or somehow, somehow, we can get information anyhow, have we heard that kind of a thing before, if you have not heard, you should say it, talk, don’t just keep quiet, if you have heard , express yourself

P1: no

P3: no

P2: no

P8: no

P6: no

P5: no

P7: No

M: so we have not all heard or seen anything like that, you have not seen anyone with that kind of condition, spotting, bleeding, you have not seen anything like that, okay, that is the first thing we see when we see someone with cervical cancer, so we are going to look further, this cervical cancer, what do we think can cause it, what do we think can make a woman to be like that, what do you think can cause it, before I tell you, I will still tell you somethings but I want to know what we know first, what do we think can cause cervical cancer, anyone, number 5, what can cause cervical cancer

P5: I don’t know

M: you don’t know, okay, but what do you think can cause it, yes, number 6, is that number 8, number 6, what do you think can cause it

P6: no idea,

M: no idea, okay who has an idea, as I said it is not an exam, express yourself , I am sure this thing will be hooking in our heart , that this thing can be caused by this or this, so we should just express ourselves, what do we think can cause cervical cancer?, yes number 7

P7: I think like shock

M: shock

P7: because there is one woman in our area, thief come and attack her, they collected money from her, and I think it is that shock as they attack, so she is bleeding

M: so you are saying shock can cause cervical cancer, you have said something, so who else is going to tell us about cervical cancer, who else has an idea, who else, who else has something to say, number 7, number 8, number 9

P: when someone is afraid

M: afraid, so if someone is fearful, the person can start bleeding, number 5, what do you think can cause cervical cancer, number 4, you have something to say

P4: no

M: number 3{NO} , number 2{NO},number 1[ no] , what is the meaning of no idea, you people are in school , are you not getting information ,I don’t want to hear no idea, it is not a good word from a student, so I want you to talk the way it is, say it, whatever you have in mind, it is not an exam, some people said some reasonable stuffs, apart from fear and shock , what do you think can cause cervical cancer

P2: when someone is thinking

M: thinking like may be the person is not happy about something , so it can cause cervical cancer, okay, that’s something , who else, so how can we prevent, how do you think we can prevent cervical cancer, how can we prevent cervical cancer, if we don’t want this thing to happen, how can we prevent it

P7: the person should stop what she is thinking about

M: okay, the person should stop what she is thinking about

P9: by eating fruits everyday

M: by eating fruits everyday

P1: the person should be courageous

M: okay, any other person, now let me give us this further information, cervical cancer is caused by a virus and that virus is human papilloma virus, human papilloma virus , that human papilloma virus is transmitted during sexual intercourse, it is done what , it is transmitted during sexual intercourse, so when a person has sex with someone who has the virus already, the person will become infected with the virus, now the person may have been infected , if the person had sex early may be like 10 years old, or 8 years old, or 12, 13 , 14, 15, the virus will be in the person’s body, for some they can be healed, while at times , it does not heal but it will remain in the body till 40, till when, when the woman is over 40 before it becomes cervical cancer , but then, now there is a vaccine, there is a vaccine for that virus, that if you take this vaccine, you will not become infected with HPV and this vaccine is available in the hospital, do we think it is a good idea?, do we think it is a good idea to have this vaccine? And this vaccine is given to adolescents , people like us , may be from 10 to 12, but then for those who have not, who have not yet had sex, this vaccine is given to them and they will just take two doses and they are secured for life against cervical cancer, do we think it is good idea

P1: yes

P7: yes

M: please wait, before we continue, you will tell me why you think it is a good idea, number 1, why do you think it is a good idea

P1: because it will prevent adolescents from having this cervical cancer

M: no, I didn’t say adolescents will have cervical cancer, they can be infected with the virus, if they have sex with someone who has the virus but if they get the vaccine, they will not be infected with the virus even if they choose to have sex at that age, you get, but the cancer itself does not manifest until the woman is above 40 years old, so do you think it is a good idea to have this vaccine at this time, why is it a good idea, number 1

P1: it will prevent the cancer from coming when the person is over 40

M: to prevent the cancer from affecting the person, any other person, do you think It is a good idea, if you think it is a good idea , you will also give me the reason you think so, he might be what somebody else said , it does not have to be anything new, if that is what you have in mind, just say it, any other person, do we think it is a good idea, if you are not responding that means it a bad idea then you will tell me why it is a bad idea, so that is it, you cannot be In the middle, number

P9:it will save the person from that disease

M: to save the person from the disease, thank you number 9, who else

P7: number 7, yes

M: okay why is it a good idea

P7: because if the person take the vaccine before he or she is 40, it will help he or she, because later on, if he or she is now an adult, he or she will not

M: so it won’t affect, if the person take the vaccine, it won’t affect the person, number 8, is it a good idea, why is it a good idea? Hmm, you don’t know, number 6 why is it a good idea

P6: it will prevent the person from contacting the disease

M: number 5, is it a good idea, why is it a good idea

P5: so that, it will prevent the person from becoming infected

M: number 4, why is it a good idea

P4: because it will fight the disease

M: okay, number 3, why is it a good idea

P3: to secure the person’s life before the person dies

M: thank you everyone, we all think it is good idea, we can have the vaccine now, and we will be protected later, is that not what we are saying, we will not be able to have the infection, we will not have the cervical cancer disease later, you are fine, now, do we have any concerns, may be, I will really like to take this vaccine, it is a good but this can come up, that can come up, do we think there may be any problem, what do we think can be the problem if the person take the vaccine, before I continue, I want us to know, we will pay for the vaccine, the vaccine is 7000 per dose, and you will take two doses , that’s how much, 14, 000, what do we think can be the major concern, we are all adolescents and we can personalize it, you have heard about the vaccine, you think it is a good idea, why will you not take the vaccine, number 9, what do you think may be the concern , what will you consider before you go for the vaccine, you know I said think, so everybody will talk, once I mention think , we should all be thinking

P9: the person will think that,

M: you, you are an adolescent, are you not, what will be your concern

P9: if I use this drug, I will die or that drug will affect me

M: in another way, like side effect abi, okay so you will think about the side effect, so what if you already know the side effect, that may be like if I take the vaccine, it will just swell a little, will you still go ahead with the vaccine,

P9: no

M: why not

P9: it will spoil my skin

M: it will spoil your skin but you will be protected, you want your skin protected first, so number 8, what will be your concern, if they say number 8 come and take this vaccine, what will you think about, what will be your concern before you take the vaccine, what will be your concern, or you will just take it, you won’t think about anything,

P8: no

M: so what will you think about, if you are told everybody is going to take this vaccine tomorrow, what will you be thinking about, what will be your concern, okay, lets much to number 7, number 8, gather your thought, you are going to say something, number 7, what will be your concern

P7: assuming I am the one there, if they introduce the vaccine to me, first, I will be thinking some bad things about the drug before I take it

M: bad things like what

P7: because I will be thinking that if I take the drug, may be it will affect me or not

M: okay

P7: May be it will bring another thing to my body

M: you will want to know if there are side effects to the drugs or maybe it will aggravate what you have in your body, aside that you will not consider the money, you can afford to pay for it

P7: the money is a concern and not a concern

M: can you explain further

P7: the money is a concern because you may not have it at the moment and it is not a concern because what you want to use the money for is to secure the future

M: thank you, that’s very good, so number 5, number 6, what will be your concern if you are to be given this vaccine

P6: I will want to know that it will not have any side effect

M: Okay, it is the side effect you will really consider, number 5

P5: May be it is going to damage part of the body, so if I take it and there is nothing then I will continue to take it

M: no, you only take it twice

P5: okay, the first time I take it, I will see if something happens to me, if something happens, I will not take it again

M: okay, so number 4

P4: I will wonder if I take it, I will not be dead

M: dead! No life?

P4: yes

M: okay, aside that, what will you think about, these are issues that affect us, if the vaccine is going to be made routine, it is likely going to be girls first, because if it is not going to be widely available, there are other things to consider though, but girls will be given priority because they are the ones who get to have the cervical cancer, what will you think about

P4: because I have not yet used it

M: because you have not used it, so what will that make you do, hmm, you don’t know, number 1, you want to say something

P1: as for me, I will consider the side effect then I will think of the money

M: why will you think of the money?

P1: the money is too high,

M: okay, what do you think can be done, you don’t know, okay number 2, what will be your concern about the vaccine

P2: I will ask the person giving it if it won’t bring damage to my body

M: okay, aside that

P2: I will think of the money too because the money is too high

M: okay number 3

P3: as for me, I will consider if it won’t damage my body

M: so we will all consider if it won’t damage our body may be it has side effects then the money, side the money, we didn’t mention anything about our parent, do we think our parent will give us the opportunity to take it, what do you think may be going on in the mind of your mummy or your daddy, if you tell them, you need money for this vaccine, this vaccine is to prevent against cervical cancer, what do you think will be,I am already seeing some expressions, so you will verbalise those expressions, number 4, what is that expression, verbalise it, what do you think your mum will say if you tell them that you want to take a vaccine, say it anyhow, say it in Yoruba, even if it is the way your mum would say it, will she allow you to get the vaccine or not

: she will say that the money is too much

M: say it anyhow, even if it is in Yoruba, what will your mummy say, it is not an exam now, why are you writing, you want to say something, number 9

P9: my mum will not allow me, because they will first of all say the money is too high, then they will think that if I use this drug or vaccine, it will damage me or anything

M: anything like, I don’t understand, what do you mean by anything?

P9: it will damage my body and I am not going to be dead

M: or maybe you will die, but they know that it won’t kill you, did you not take immunization when you were younger , so I am sure they won’t think dying so what do you think may be their concern, apart from the money, what do you think may be their concern, yes number 7

P7: first if my parents hear about it, they will think that the money is too high , secondly, they will say that if I use the vaccine , what did not happen to me before may start happening,

M: like what

P7: like the disease we talked about, okay, they may think that it may actually bring the disease to you

M: aside that what do you think they may be thinking, there is something I want to hear but I don’t want to put it in our mouth, what do we think

P9: they will be thinking that, like me now, if I take the vaccine, like me now, if I am doing my menstruation, when I now take the vaccine, I will not see it again, so they will be thinking about that

M: okay that it may affect your menstrual period [yes] aside that, is there any other person

P6: my parents can give me the money, but then they may start thinking that, since someone can contact it through sex, may be that I am now having sex or something like that

M: so they may think it will give you the license to start having sex

P6: yes

M: that’s good, I have been meaning to here that, because we keep seeing that coming up, okay so, is there any other thing, what do you think will be the concern of your parents, if you go home and say mummy, I want to get this vaccine, what do you think they will feel

P3: she will be thinking about the money because the money is too high and secondly, she will be thinking maybe it won’t affect my body

M: if this vaccine were to be available, we already said we were going to take it, is that not, but then we will consider the side effect, is that not, what do you think, how can we ensure that adolescents get this vaccine, what do you think we can do to ensure that adolescents get it, if this vaccine was to be introduced into routine immunization like we have people going round to give injections and all this kind of things and you go to the hospital too to get the vaccine, if this vaccine too was to be included like that, may be this is the time for this vaccine, everybody come ,routine, from time to time, how do you think, what do you think that we can do to ensure that every adolescent has access to this vaccine, how do we ensure that adolescents get this vaccine. Who are adolescents, is that not?

All: yes we are

M: you know we don’t know about the vaccine, is that not, I asked , have we heard about it, we said no, have we heard about the vaccine or the cancer, we said no, so now that we know about it, how can we ensure that adolescents get the vaccine, it is we, all of us, you tell me how I can ensure the vaccine gets to you, it is you , all of us, you will tell me what you can do, what your parents can do, what your teachers can do, what the government can do, what everybody can do, to ensure that we are protected because if people have cervical cancer, will they not die?

P7: we can be advertising about the drug

M: where do we advertise , who will do the advertisement

P7: people that know about the drug, people that do it

M: so who are the people that know about the drug, who are the people you think should know about the drug [no 9} let her finish please, so who are the people you think should know about the drug and health related issues, teachers, trader in front of the house, so who do you think should know about the drug, I want you to mention those involved, say it, okay you don’t want to say it, okay number 9, lets hear you

P9; doctors, they can advertise

M: aside that , do you have any other person that may be involved, number 1 , you want to say something, how do we make sure that number 1 gets the vaccine, what do you think we can do to make it easy for you to get this vaccine

P1: advertise it through those that have used the drug, if they advertise it, do you think that is sufficient

M: so if they advertise, will you be willing to look for it anywhere

P1: they should make it available

M: make it available, where, by bringing it to this table? So how do we make it available, or we should bring it to your class, or we should bring it to your house, talk now, how do we make it available, I want us to answer that question, how can we make it available, number 1 said we should make it available, how do we do that, number 3, how can we make it available

P3: pharmacies

M; we should put it in the pharmacies, it is a vaccine, it is not a drug

P3: and the hospitals

M: and the hospitals, where else, how else can we make it available, what kind of hospitals, government hospitals, which one, which one of the government hospitals, which one are you referring to

P3: UCH,

M: so everybody goes to UCH

P3: Adeoyo

M: people don’t go to

P2: John b

M: hmm, John B, that’s a private facility, so we can take it from private and public hospitals, apart from that, what else can we do?

P1: we can go to people’s houses

M: yes, we can go from house to house, okay, this is Number one’s house, we are trying to ensure that everyone gets the vaccine, how else can we make it available, how do we ensure that everybody gets it, what else can we do apart from making it available and advertising, we have mentioned advertisement , we have mentioned making it available, what else can we do to make sure it gets to us

P5: through announcing

M: we have already said that, it is the same as advertisement, what else can we do

P7: if we can reduce the price of the vaccine, if people know that the money is small, they will take the vaccine more

M: so if we reduce the money, people will take it more, okay, thank you, who has more points, okay if we don’t have any more point, then that will be the end of the interview, thank you for your participation.
