# Supplementary material for: “She must have been sleeping around”…: Contextual interpretations of cervical cancer and views regarding HPV vaccination for adolescents in selected communities in Ibadan, Nigeria
Source: PLoS One. 2018 Sep 17;13(9):e0203950. doi: 10.1371/journal.pone.0203950 (PMC6141096; doi:10.1371/journal.pone.0203950)
Supplement: S1 CaCx data — (ZIP) [file pone.0203950.s002.zip › FGD FEMALE TEACHERS 1.docx]

**TYPE OF PARTICIPANTS: FEMALE TEACHERS**

**TYPE OF INTERVIEW: FOCUS GROUP DISCUSSION**

M :Good morning ma, as I said my name is ……… and I will be the one to moderate this session and my partner is ……… and I want all of us to talk to say everything , because what is happening to us personally, it affects all f us and it is not about our socio economic status or where we come from, wherever you are as a woman in Nigeria today, cervical cancer is something that is really important , it is not even limited to Nigeria , outside Nigeria it is also relevant, it is important we know about it , so this study will explore our understanding, how well we understand, what do we know about this cervical cancer, what do we know about the things that can cause cervical cancer, soo I will like to ask us, have we heard about cervical cancer, who has heard about cervical cancer. Have you ever heard about cervical cancer, please mention your number, what is your number, number 2

P2. I have heard about it, but I don’t really know much about it, I have heard one or two things about it

M. like what and what

P2. It affects the private part of a woman and it is something that can be cured, also if you detect earlier, that is what the check up is for, to know the way your body is, at least once in a while one should go for check up, at least twice in a year, as a woman , we should know the state of our body because once the disease is detected, we will be able to treat it

M. number 4, you want to say something

P4. The first time I heard about cervical cancer was when I went to the hospital to have my third child, so at oluyoro, we were asked to come back for post-natal check-up that after certain month, we should go and do screening, one of the nurses was saying that when she had her child she went for screening , there was when she discovered that she had the traces and because she went earlier , she was able to detect it, so that it was good for all of us to do it, but I didn’t do anything about it until last year when I lost a friend, colleague, I mean one of our old students, we were old students together , infact it was two people who died from it that last year and that was when I was like so this thing is something serious, when I was to go to oluyoro, I won’t lie , I did not go and when it now happened to my friend, I now thought about it ,so this thing is real, it happened to someone that I really know, that was when I thought about it , it is real

M. has any other person heard about it, who has heard about it, let’s feel free to talk, what have you heard about it, no 6

P6. What I heard is that it affects woman,

M. apart from that it affects woman you have not heard anything about it, what about you no 3, what have you heard about it

P3: I have not heard anything about it

M. have you heard anything about cervical cancer

P4: I have not heard anything about it

M: have you heard anything about it, that is different

P7: I did the screening last year at vine branch, Dr Kolade’s wife and some other people they organized the programme for us

M. so I will just describe cervical cancer, cervical cancer is a cancer peculiar to women above 40 years, when you see a woman that is supposed to be approaching menopause starts to bleed uncontrollably and the bleeding is not just the usual bleeding and it starts to smell very foul smell, you have low waist pain, the bleeding comes in between menstruation after sexual intercourse, and it is not menstruation, it is continuous, it is something that smells bad, there can be weight loss and the person may not loose weight, that is how we know that it is cervical cancer, the person is just bleeding and the person is more than 40 years, we then know that it is cervical cancer, it can smell or it may not smell, these are the things that point to the fact that someone has cervical cancer, and some people may not pay attention to it, before you know it , the person is dead, may God not allow us to see something like that . have we heard anything related to those kind of symptoms, have you seen or known anyone who has been involved in anything like that , the symptoms I mentioned, is there anyone who has seen something is like that, have we seen anything related to that or similar to that

All: no, no

M: you have not heard about it, may be someone that something like this happened to, in church, in school

All: none

M: so you have not seen anyone who has had it

All: we have not

P4: I have seen something like this but I don’t know that it was what we are discussing about, we were just told that it is cervical cancer that killed her

M. all the things we have mentioned, where did we learn about it, P4, that information you have, where did you learn about it

P7: from friends, then I heard about it in school

M: which school

P7: At Ado Ekiti, university of Ado Ekiti in Biology class, they told us in Biology class

M: so you heard about cervical cancer in your biology class, so ma where did you hear about it

P4: actually, my mother is a nurse, I knew about it through her books, I read about it in her books, the first time I heard about cervical cancer, was when they were talking about the use of intra uterine device, that it could increase the risk of having cervical cancer, then we just use to read, we don’t really know what cervical cancer is about, but it is not as if I have seen someone who has it, I have not seen someone with cervical cancer and I did microbiology too, so all these HPV, HIV , I know about it, but it did not really sink, we just thought that It was a figment of scientist imagination, so it is when you now see someone there that it will now sink that this thing is real

M: where did you hear about

P: church

M: what about you

P: church too

M: We have heard in church, read in nursing books learnt about it in class, so this are all the sources, so what do we think can cause cervical cancer, what are the causes, what can cause cervical cancer

P1: I don’t know,

M: you don’t know, you don’t know what can cause cervical cancer, you have not heard anything about it, p6, what can cause cervical cancer

P6: I can really say this is what causes it,

M: you just know that people have the cancer, no, maybe we should place our numbers in front of our chest, number 3, what can cause it

P3: I don’t really know what can cause it, I don’t know, I just know that it is a viral infection, it is a virus infection

M: What can make the virus infection stay, virus don’t just jump on people

P3: it can be because of dirt, or anything , STD sexually transmitted disease

M: number 2, what can cause this things

P2: it is the same thing,

M: what is the same thing, she said a lot of things, we know that there was a lot of things said, what do you think personally

P5: the thing I know is that it is sexually transmitted, may be due to the stress that woman go through during delivery

M: child bearing stress,

P5: child bearing stress

M: what do you think causes this diseases number 4

P4: I have read about it and I know that it is a sexually transmitted infection and I also look at it that some people , it is not as if they led a promiscuous life and they still came down with cervical cancer , I am also thinking may be it is genetics, in some families , if someone has cancer, it is down the line in the generation, some other people may also have it, may be it is transmitted from one person to another in the generation

M: so you are saying it can be in the blood, yes it can be in the blood

P4: yes, in some families, some people are more prone to have it than others, that’s my own thinking

M: please speak up

P5: may be abortion

M: abortion, what number

P5: number 5

M; can you explain further

P5: I don’t know

M: that is a really big word, how can you break it down for us, how does abortion relate to cervical cancer

{ session becomes rowdy}

M: let her talk, what do you have to say, you talk

P: tell us your own opinion, so we now add to it

M: you said abortion, and she may not be able to say what is on your mind directly, so say your own, if it is contrary, she will now say what she has in mind

P5: I don’t really know

P1: if someone goes to a substandard hospital for the procedure

P4: what I think is that, as she mentioned abortion, if one goes for abortion in this road side clinic, you know the instrument they use they might have used it on somebody that has the virus and if it is not sterilized and they use it for someone else, the person can contact it, then by this uterine device , may be the person who inserted it did not clean up well, did not take the necessary precaution, it can make the person become exposed to this things

M: so you are saying that one can become infected through family planning

P4: someone can have it, if they go to a place where they will not do it well, I have read it that inserting that intra uterine device exposes one to the risk of cancer, so it is possible , it could be because of that

M: what do you think cause cervical cancer

P7: I don’t even know, I don’t know if it is unhealthy lifestyle, may be the kind of food we eat, I will want to ask you if it can cause it

M: umber 7, unhealthy lifestyle

P7: The kind of food we eat , I think it can also cause it, i don’t know if it is causing it, may be use of contraceptives, I don’t know if it is causing it too

M: Contraceptives, which kind of contraceptives, they are many, I don’t know the specific type, I just think it may cause it too

P4: You know some people use tampons, you know when you use tampons when they are having their menstrual cycle, it could, I don’t know, but I think when you insert these tampons, it could also be a risk factor

M: Thank you

P8: from what we have been saying since

M: what do you think can cause cervical cancer, have you ever heard it

P8: yes

M: So what do you think cause cervical cancer

P8: To me, you know I just joined the group,I was not here before

M: Where did you hear about it

P8: Through the interview, through the television

M: So what do you think causes it, what were you told on the television what did they tell you causes it, where did it come from, where is it from

P8: Cervical cancer, what causes it

M: yes

P8: I cant say much on it, because it is not my line, it is true, I cant say much on it, if I am in medical line, ehn ehn,

M: But as a layman, what do you understand. As a layman, you still have some knowledge of what it is, you have heard about it, what do you think it is, what have you heard about it

P8: What I was thinking is that may be through this ehn, family planning, it can cause it

M: How do you mean

P8: For example now, for someone using copper T, I know someone that I was told the story, I am not sure of it, the person said they did copper T , along the line, when it was time for the person to go and remove the thing, eventually the person had it,

M: Copper T is the same IUD you mentioned

P8: Yes, the person heard it, and the person said it, and I have someone that did it and did not have it, so you see what a person does and gets away with it, another person might not be able to do the same, this our body, our skin is different from each other, so through this family planning somebody can have it

M: Thank you very much, so how do we think we can prevent cervical cancer, lets talk about all the thing]s we have mentioned about the causes, how can we prevent cervical cancer

P3: To me, since we don’t even know what causes it exactly, I will say that it will be good if we go for constant check up, since a person is aware that he or she has it, he should treat it, then seeking health advice, that is what I think can help

M: How can we prevent it, number 1

P1: One should be proper attention to one’s body,

M: When you say pay attention, what does that mean, what are you saying exactly

P: I mean one should take care of the private part very well during menstruation, and if one wants to use the toilet may be if the person is outside, one should be careful of where to use

M: so you are talking about hygiene right, ma, what do you think we can do to prevent cervical cancer

P: firstly, what I think is we should be doing proper hygiene of our bodies, then we should caution ourselves about using toilets, if we want to use toilet, we should make sure that we clean the toilet first before we use it, so that we will not contact any disease

M: so if we are doing the hygiene of our toilets, we will not have cervical cancer, p8, how do you think we can prevent cervical cancer?

P8: The way we can prevent it, number one , for example, whoever is the spouse, I mean your spouse, whoever you are married to that is your spouse, you know there are different diseases you can pick from men and once you have someone steady, fine, you go for check-up, if the man has gone for medical check-up, the woman too can go for medical check-up, because if you don’t go for medical check-up, through all this sexual intercourse, infection will come in, and if you pay attention to it, from there, it can cause cervical cancer but the way to prevent it, is it is better we go for medical check up always

M: By that always, what is your definition of always, Is it every day we should go,

P8: No, no, no, it is either twice in a year, [okay] or three times in a year

M: Ma, how can we prevent cervical cancer, number 4

P4: What I think is that one should avoid having multiple sexual partners especially the men, you know that men can bring it from outside and it does not manifest in men, it is the women that suffers, so the men should be enlightened about it , so that they know this is the risk they are putting their wives through by going around ,may be they will show them mercy a bit, and even the women that like jumping from one bed to the other, once they know that there is something like this they can be infected , so they will take caution because there is no body that wants to die, apart from the money we will be spending, it is stressful so people will stay away and there should be more public enlightenment, I can tell you that people don’t really know about it, I can tell you that as learned as I am, I didn’t know about it until I went to the hospital to deliver that was when I knew there was something about cervical cancer , I have actually heard of it but I didn’t think it was something important, until I started seeing people actually die, if people know that this thing is happening , if you go to UCH you see cases, people will have the fear , you know the time of AIDS , when people started seeing it themselves, and they saw that people are dying of AIDS people started doing what they are supposed to do,

M: Thank you, so how can we prevent cervical cancer, no 5

P5: What I think about it is that, there are some people that when they want to menstruate, they will use cloths and this things cause different types of things because when some people use it like that, they will not wash it clean and when they want to use it again, they will just pick it from where it is, you know, if you know that it is cloth you want to use, you don’t have money for pad, you will take care of it, you can even iron it, so that is what I think about that

M: So you are saying menstrual hygiene is important, no 7

P7: It is as they said

M: What did they say, many things have been said

P7: Hygiene, we should be taking care of ourselves regularly, and go for check up may be once or twice in a year and once you are feeling any discomfort, the best thing is to go to the hospital for check up, so that you will be able to know what is wrong, just go for check up from time to time, so you know what is happening

M: We have all mentioned check up, for most of us here, within the last year, how many times did you go for check up

P7: Once

M: How about others, no 8,I want to ask , what do you know about human papilloma virus, have you heard it before

P2: I have heard it

M: You have never heard of HPV, you have heard of HPV, what number is that

P2: I have heard of it, but I really don’t know what it is, one of my lecturer mentioned it

M: Okay, okay, is there any other person that has heard of HPV

P4: I have heard it before, because I did microbiology, so one way or the other, I have heard it

M: So what do you know about HPV, what do you know about the virus

P4: All I know is that , it is a sexually transmitted disease, the virus is transmitted through sexual porter and I think it is the same one causing cervical cancer,

M: Is there any other person who has heard about it, okay as mentioned earlier, it is that HPV that causes cervical cancer, it is the virus that causes cervical cancer, that cervical cancer it is HPV that causes it, if the HPV does not heal, it will become cervical cancer, so it is this HPV that causes the cancer and it is sexually transmitted, so I want to ask , have we heard about this HPV vaccine, do we know if it has a vaccine, we don’t know if the vaccine is available, we don’t know there is a vaccine for HPV, lets talk , there is no way the recorder will capture our nonverbal clues

All participants: We don’t know

M: so you have not heard about the vaccine, you didn’t hear about it from anywhere

P5: it is as if I have heard it, I have heard about it, but it is expensive, I think they mentioned it in vine branch, last year when we went for cervical cancer screening , I think they mentioned it , some people they pay for it, I think they mentioned it

M: So you think there is something like that, you are not sure if it is available or not

P5: It is available

M: Okay, it is available, who else knows of its availability, who knows if it is available or not, okay, there is actually a vaccine, that if we give our adolescents boys and girls, it will prevent them from having cervical cancer, and this cervical cancer that we are talking about, there is a way you can do it that if you attend to it early , you will be able to get rid of it, so what I want to ask is that, what are the advantages , what are we going to see, what do we think will be the advantages if our adolescents are given this vaccine to prevent cervical cancer

P4: sorry that vaccine for how many years

M: The dose, once we finish, we will elaborate more on it, so the vaccine should be given to adolescents between 10 to 19, those who are not sexually exposed before they will become exposed, we will give to the adolescents and they will be protected and as she said, it is financially implicating, we have to pay some money to get it, so what do we think are the advantages of giving this vaccine to our adolescents, if we give our children this vaccine

P5: It will prevent them feom not having this infection in the nearest future

M: Apart from preventing them from having this infection, what are the other benefits

P4: It will help us put our mind at rest

M: Please let’s mention our numbers before we say anything

P2: It saves life, we will have less worries about them because they are protected

M: Asides that what are the other benefits of having the vaccine

P2: We have reduced the risk of transferring the virus,

M: No 2

P2: We won’t be at the risk of transferring the virus

M: Number one, what do you want to say

P1: It will not let them have the infection

M: It will not let them have the infection, in what way?

P1: Because that thing they are taken will go into the blood and water and prevent them from having this things

M: What else can we say , we didn’t even think that if they take the vaccine, it will make them more promiscuous, a child we are trying to protect and you are saying the child should come for a vaccine

P: May be there will be an orientation, number 2, may be the adolescent will have been orientated about the virus, don’t say because you have taken this vaccine then you will be moving around, there should be awareness, more awareness about them being infected and at the same time, they should be discouraged from jumping around

P4: As she said, it is very important because this is a deadly disease, and nobody wants to die and like you said that will it not promote promiscuity, what happen about that is that when they are teaching this children, they will let them know, that HPV is not the only sexually transmitted infection, if you sleep around you can still get HIV, you can get gonorrhoea, you can get syphilis, so don’t say that because you have the immunity you can flirt around ,there should be proper orientation so that they will take it to, and in our own ways too, the way we treat a child is the way they will grow, let your child have values , as a Christian as a muslim, let them know that well before marriage you are not supposed to sleep with anybody, that will stop them from sleeping around

P: no 2

M: please let number, what is your number ma, number 3, what is the benefit in getting this vaccine

P3: as they have said earlier, it will prevent them from having the disease

P2: I think there should be more announcement about it, the radio stations should air it, parents should be aware, also in hospitals, place the banner all around, so that people will see, those who cannot read it, may be through pictures, they will see and deduce from it, even the laymen will be able to respond

M: do you have any addition , number 8 , number 5, do you have anything to add, okay so what do we think are the disadvantages in getting this vaccine, what is the disadvantage, or it does not have any disadvantage

P: the disadvantage there is that ,it can expose some other children may be those that don’t have before, they will become exposed to promiscuity

M: so it can make children more promiscuous

P: yes, because they will look at it that since they have the vaccine, they will not be infected with the disease anymore, that they will be going around

M: do we all agree that it will make the children promiscuous

All: yes

P1: I don’t think it will expse them to promiscuity because it is not only thorugh sex that someone can have the infection, if the person is not hygienic too, someone can become infected with it, so I don’t think it can expose the children to promiscuity

M: okay ,so it is not only sex, do we have any concerns or fears about the vaccine, that if the adolescent should get this vaccine, this or this can happen, so for this reason we will not allow the children to get this vaccine, do we have any fears or anything that may be of concern to us about getting the vaccine

P4: there should be no fear because with the way , with the way, the occurrence now is many, you just see people that you know dying, and if you ask what happened they will say is cervical cancer, something that was not like that before, and once it becomes like that, it is better to be safe than to have it, it is better to give them and if a child decides to misbehave because of it, she will face the repercursion by herself, although her life is secured ,s eh cannot come down with cancer ,if she has other sexually transmitted infections, she can still treat it, it will not lead to death, but this one there is no cure, there is no cure for it

M: so no concerns about being infected with the disease,

P4: no concern

M: if there is no concern then as parents, can we allow our children to be vaccinated, I want us to talk one after the other

P5: I will allow my girl child it is for her protection

M: so you can allow your child to be vaccinated, number 7, you can allow your child to go to the hospital, and tell her, lets go and take this vaccine

P7: yes, she can take it

P4: I can take it very well, it is not so they can sleep around , even when they get married in the future, the person they will be married to I don’t know, once they are protected, everybody will be safe and secured

M: no 7, can you allow your adolescent to get this vaccine

P7: yes

M: you don’t have any complain, you don’t have any fear, you are at peace with it[p7: yes], what about you no 8

P8: yes, I can take it, you are protecting your child against virus,

M: can you take your child to get the vaccine

P1: yes, I can give my child, it will protect her in the future

M: what about you , number 3

P3: I will want to have more knowledge about it before I give my child, you know, you can get a vaccine now, that it will have other side effects, so I will like to know that before I allow my child to have it, I will want to have much knowledge

M: so you are thinking the vaccine can have side effects

P3: yes,it can have side effects

M; what kind of side effects are you looking at

P3: you they said that some people after getting that family planning , the injectables, they could not have other children again, that was there, and may be that one too can have other side effects, it may not be related to having children

M: but you will like to ensure that

P3: I know the side effect

M: do we all agree that we should know the side effect before we get the vaccine

P8: sorry ma, number 8 just to add more, you see what she is talking about , fine you should have knowledge about what you are to be given, the same thing applies to the drug we use, I don’t think there is any drug without side effect,the same applies to the vaccine we take, look at all this family planning injection or this copper t, there is nothing without side effect, except you want to lie, the same thin applies to this vaccine too, there is nothing without side effect, you see at times, when you buy the drug, they write this side effect on the prescription note in th e drug, if you want to take this, don’t do this, don’t do this, after taking this, make sure you rest, the same thing applies to the vaccine, you see the vaccine we give to our children, when some children get it, some will not even have any inflammation, and some, there hand will be swollen after three days and some children will get it and nothing will be wrong with them, such that within few hours of having it, they will have started playing, if they have temperature and you give them paracetamol , the temperature is gone and some will still have temperature for 3 days unending, the same thing with this vaccine too, you must have more knowledge

M: p4 you have something to say

P4: what I want to say is that there is no big deal about the vaccine, vaccines are just may attenuated form of the micro organism, so if it will have any reaction, it will be a minimal thing , there is no long term effects , may be for someone to be feverish or something, and that far outweighs, being exposed to the virus, so vaccines generally don’t come in with much problem,

M: that was what I was about asking, that are we going to say because of the effect of the vaccine, that the side effect is so much , we wont give our children

P3: don’t say that, there are some side effects that you will feel like even if she is exposed to the risk ,it is much better than this, you know now, if a child gets the vaccine and at the end of the day, she is told, your womb , the vaccination you had is the reason[ other participants laugh] why you cant have children or is the cause of this thing you have, and then the child will be like, if they have left me alone, may be I wont even be exposed to the virus, it depends on the side effect, how is it, if it is too strong then

P8: well, number 8, about what she said , some of the drugs that I have read the prescription note, they will make you understand that they have tested it on animals, do you understand what I am saying, before they ask humans to come and use it, the whites that want to give us the vaccine, they have tested it and they have seen that what they are given will not be so harmful , the worst that can happen is for the person to be weak and that may be for 2 days , after 2 days, the person will bcome fine again, it is still preferable to have it, having it is better than not having it , if you get it, is not to protect your self, it is better, I prefer that you have it and it is better than not having it at all

[ participants talking underground, you are correc, you are correct}

M: do we all agree with what she has said or do we have anything we want to add

P3: I number 3, I don’t agree

M: you don’t agree

P3: yes, I don’t agree, you see that paracetamol that we are using ,it was recently that I heard that it is affecting the liver because of too much use, talkless of the vaccine that will not allow that kid of micro organism to come in

P4: you know cervical cancer is a form of cancer too and I heard that if you use garlic and all these othr fruits, I haeard that if you take it, whatever form of cancer it is it will kill it and I heard it has been tested , although I don’t know how true is it, but I heard that it works , then this sour sop too and then I heard that there is no kind of cancer one may have , once the person takes that thing, so I heard but this one is not for this forum, may be later I want to ask[ laughs] that

M: okay, lets put it in the question and answer forum

P6:in addition to what they have said, there is nothing one can eat, if you eat too much, that will not have an effect, but since this one is just prevention, is not that we will keep getting it, that one cannot have much drawbacks , so that we will not get the vaccine, you know we mention paracetamol now, before you take paracetamol to the extent to the extent of developing liver problem, it must have been too much, it is not that, you are asked to use paracetamol three times, and you have used it three times, if it didn’t work then it can’t work for that problem , so discontinue, but if you continue to use it, it will have effect on your body, so that is, it, even if it is water you take too much, it will have an effect on the body, if you take the normal proportion there will be no effect, but anything that you eat more than the normal dose, it will have an effect

P4: in addition to what they have said , yourbas say that whoever has experienced the wrath of sango will not join in derogating it, anyone who has sees a child die, like the person whose child died , the person was my teacher, and she is still alive and the child that died was very promising and she died at her prime, the mother is the one taking care of the children she left behind now, if it was during her time she heard that there is a vaccine even if it will not work, even as I am if someone should ask me to come for the vaccine, will I not go, it is true now, so you will first forget about anything concern, if you have seen the problem of cervical cancer, you will know that it is not something that you should joke with

M: thank you very much for your patience, we are gradually coming to the end of this interview, so I want to ask, if there is a plan that this vaccine is made routine like other childhood vaccines that we give our children, how can we ensure that our adolescents are able to access this vaccine, what can we do, what can we do to see that it works out fine

P1: there should be a place that they will set aside may be in the hospitals and the private places too, then they can ask them to come and be taking the vaccine, then they will tell people about it, please come and take this vaccine , then you can have more people coming for the vaccine

P3: if they want to set up a place for adolescents to have this vaccine, the first thing is they will need to create an awareness first because most people don’t know about what you are talking about, when you are done , then you tell them how it can be prevented and I am verysure people will take it, but if you just put in on ground without it, people will not get the vaccine, even me, that is what I will say , God forbid sickness

[ everyone laughed}

P8: it is good they make it public very well before starting it, for example look at HIV, when it first came, people did not really know about it,not until federal govt started creating awareness, on the television , all around, they were going round to tell people about it, even this women who trade in the market, they will talk to them, mama, we want you to know that, from there, gradually, since they started creating the awareness, I have not seen any govt hospital that you go there, even if its your child you want to see the doctor, they will ask you to first do HIV test, that is the only way , if they want people to hearrr, they will first create awareness and the federal govt too should realise that it is their responsibity , sothey have to take it up first, all the hospitals will have it, the same way they say go and do HIV test, it is free, so, then they will say that so and so test is this amount, someone may just open mouth, me that I don’t have enough money to feed{ laughter from participants} you see when you asked that how many of us has gone for check up, only one person raised her hand, that’s the way it is

P7: the reason why HIV test is easy for people to do, once they get there is fast, look at Malaria test too, MP test, when you get there, most of the government hospital, it is free, they will ask you to bring your hand, in the next 5 minutes, the result is out, because it is free , if it is money money money, people will not do it,

M: so you are sayin that HPV will not be acceptable

P7: except the government makes it free

P8: awareness, they should let us know, they should create awareness, when people know about it , fine

M: apart from awareness, what else

P8: they should make it free, if they do that it will make thigs easy

P4: it is too expensive, like the one I was asked to do then, I was still trying to convince my self to do it, when I got there and they said 20 000 for the test, I said ,how much is my salary , 20000 for test, may God forbid sickness, [ participants laughed] I just left that what is it, 20000, abeg, but if it was like 2000, I will convince my self , and even then I didn’t know it was something this serious, if it is reduced, even then, when people know the magnitude of the problem and they see their friends and relatives dying from it, even if they call it 20,000, people will not mind to have the vaccine, don’t lets deceive ourselves, one will look for it, are we not spending more than 20000 on clothes, it is the awareness that really matters, may God forbid sudden death, this thing is really happening and it is happening to people that you really know, you know some people , when you mention cervical cancer, they will just be like, they have stated , they will just be writing what ever they like, research[ laughs]

P1: may be the cream that they used in bleaching is the thing affecting them

P4: or may be they are trying to scare us

P5: what she said is really important, go to church, mosque, market, make sure people are really aware that there is something like this, then the money is an issue , it may make people draw back, some people don’t have that kind of money to take care of themselves

P1: when I didn’t have money , I managed my ill health at home, so if there is no money, It can be a great threat, if it is not affordable, it will stop people from taking the necessary steps

P4: the level of poverty too, if someone knows that this thing can kill, she may be like let it kill me, there is no money to spend , malaria is killing some people because they don’t have money to buy the drugs, so the level of poverty too, is a serious matter, even if they keep emphasing it, will be like I don’t have any money, the person will rather resort to faith healing, she will be praying, so poverty can be a real issue

M: now that we know that HPV is sexually transmitted, are we still going to have it

P3: we have said that before, and we said yes, they can take it, but we need to orientate the parents, so that they can know much about it, you cant just ask me to come and take a vaccine for my child, you see that one they give on the arm, I have a sister that said she can never give her child ,never, she can say that the pains, the children go through once they have that vaccine is unbearable, and I thought about it, she is reacting that way because she does not know what that thing is working for in the body, so if you just say, go and take one cervical cancer vaccine, I will not take it, because people believe that the federal government wants to reduce the population, that is what they will think first, that once they take it, now the children will not be able to conceive

{ laughter from participants]

M: if I didn’t ask now

P3: I know what I am saying, yes now, it is true now, that is what people believe, you will see that people are not doing family planning like before apart from the inserted ones, it is true, people are not taking injectables, so that is what is happening, if Government can create the awareness, they should make sure that people know about it, they should let the parents know about it, so and so and so , if you just ask people to bring their children, even me, I wont answer

M: thank you for your patience, lets clap for ourselves

{participants clap}

This will be the end of the interview
