# Supplementary material for: “She must have been sleeping around”…: Contextual interpretations of cervical cancer and views regarding HPV vaccination for adolescents in selected communities in Ibadan, Nigeria
Source: PLoS One. 2018 Sep 17;13(9):e0203950. doi: 10.1371/journal.pone.0203950 (PMC6141096; doi:10.1371/journal.pone.0203950)
Supplement: S1 CaCx data — (ZIP) [file pone.0203950.s002.zip › SENIOR BOYS public.docx]

**TYPE OF PARTICIPANTS: SENIOR BOYS Public school**

**TYPE OF INTERVIEW: FOCUS GROUP DISCUSSION**

M: As I said, my name is …………… and my partner is …………, and we are here to know your perspectives, we want to know what you know, about cervical cancer , about human papilloma virus , about human papilloma virus vaccine, and everything we will be doing here , every information you will be giving us here, it is confidential, it is not going to be used against us, I want us to feel freeto express ourselves, feel freee to do what

All; to express ourselves

M: don’t be quiet, I don’t want anyone to be quiet here, it is not an exam, there is no wrong or right answer, don’t even make fun of your colleague, your colleague is making a comment and you feel the comment is not correct, it is your colleague’s opinion, not yours and as far as I know, everyone has a right to their opinion, you have a right o yours, I have a right to mine, let no body condemn another, everything you are saying is all correct, don’t look at another, and say what is he even saying rubbish, don’t comment, no side comment, if you have anything to say, just raise your hand, I am number 3, this is my own opinion, this is what I think, os the interview will not take so long,I want us to talk express ourselves, and I am sure that what we are going to be saying will also benefit us, so can we start with the interview, so everybody I want us to talk, we are not here to be quiet, so the first question I want to ask is, have you ever heard about cervical cancer, have you ever heard and no nodding , no non verbal language, I don’t want any non verbal language, everything you are going t say, let it come out so that the recorder can capture it , you know if you are nodding your head ,t he recorder will not capture, so if you have something to say, let me know, so have you ever heard about cervical cancer

P4: yes,I have heard of it before

M: you have heard about cervical cancer, so no 4 has heard of it, any other person

P2: yes

P3: no’

P1: no

P9: no

P8: no

P6: no

P7:NO

M: number 5 tell us what have you heard about cervical cancer

P5: I have not heard about it

M: number 4 what did you hear about it

P4: I heard that it is a very deadly virus, more like , if care is not taken, can kill very fast

M: cervical cancer is deadly, if you don’t take care, it can kill fast, so where did you hear about that

P4: let me say, TV

M: on TV, thank you, so number 3, you have heard about it

P3: no

P2: I heard about it, during the 2016 period, during the Christmas, one of iur uncles told us, that a male can be a partaker of it from the female during sexual intercourse, that we should be careful in doing this thing and, the person may not know , and if the person becomes infected , there is no drug to survive from it, it is death

M: who gave you that information

P2: it is one of my elderly brother in my home town

M: in your home town, it is not like a church, or anything, it was just a family discussion , so you learnt that it is dangerous and it is transmitted from man to woman, okay thank you, any other person, hmm, nno other person, so you are right, cervical cancer is deadly, cervical cancer is dangerous, cervical cancer is sexually transmitted, you are all right, now, let me just describe cervical cancer to us, cervical cancer is a cancer that affects the woman and it affects women above 4o years, when you see a woman above 40 years old that is bleeding, you know what the cervix, is, the cervix is the entrance to the womb, it is part of the woman’s reproductive organ, when you see a woman above 40 years that is bleeding unnecessarily, it is not her menstruation, it may come with waist pain, and at time, it may not come with waist pain, she may be loosing weight, and she may not loose weight, but she is bleeding, if you don’t take care and it is not menstruation, it is not postpartum haemorrhage, you know some people continue to bleed when they give birth, it is not menstruation and the blood is smelling, it smells very bad and the woman is above 40 years, then you should suspect cervical cancer, have you ever heard or seen someone with that kind of

P: no

M: don’t just talk randomly , I want you to mention your number

P2: I can say that I have seen that kind of person before, but the person is not up to 30 or 40

M: you have seen someone like that before, and the person is not up to 30 or even 40, if the person is not up to 30, then it may be something else, at least research has shown that the person , though the virus may be present in the body much earlier, it will not become cancer until the person is above 40. So there are 2, it may be another condition, you get, it may be another condition, different things make people bleed, we talk about that later, so has anyone seen anyone who is over 40 years old, may be your friend, or your friends mummy, or your mummy’s mummy, or someone in your neighbourhood, or somebody in your church or someone somewhere, somewhere, they just told you about it, may be you were even passing and they just told you that woman bled till she died, and it smells , have you heard anything like that,number 6 , you have something to say[ no] you have not seen anything like that, but you were really looking as if you had, you were trying to see if you have seen or heard about it, you don’t know or you are not sure, okay, what have you heard , what have you seen, and I want us to know that we are free to express ourselves in any language that is convenient for us, do we understand, it is not compulsory we speak in English, everything will be captured, we can speak Yoruba, we can speak English, so have you heard anything , so o ti gbo anything, abi eni Kankan ti iru nkan bayen se ri, there is no one you know, may be in the neighbourhood or your kinsman or your parents colleagues or people they are in the market with, that has had that experience, so we have not all heard that experience, number 8, [no], it will not be used against you, what about you number 7, have you seen it, [ yes], where

P7: in my mother’s shop

M: can you tell us what happened exactly

P7: I have someone that had the experience once, when the person coughs , there is blood

M: is the blood coming from the private part, or wher

P7: yes

M: the blood is coming from the private part, so when the person coughs, blood comes out from the mouth and also comes out from the private part, so is the person over 40 years old

P7: she is over 40 years

M: where is the person now

P7: she is still there

M: she is not yet okay, so it can be cervical cancer, who has seen anything like that apart form number 7

P4: my mummy told me,

M: so tell me about it

P4: at her place of work the person is bleeding, and its spot, but she said that they have done something about it

M: where did they take the person

P4: the hospital

M; not the traditional healer, so how can one have this cancer

P4: through sexual intercourse, or through blood transfusion

M: where did you hear and how did you hear that

P4: its just my own idea

M: your own idea, you did not hear it from anywhere

P3: through unscreened blood transfusion

P2: I can say that , you know it get to some time that the period will not function again, when its that time then the period stops,

M: so you think menopause can cause cervical cancer, if someone is not having periods, the person can have cervical cancer

P1: my ideas is that, about that cervical cancer, I think it can be caused with this environmental pollution, s environment that is not well, a dirty environment , the kind of ari people are breathing in, from the environment, if the environment is unclean or something like that, it will affect the

M: so if the environment is unclean, 0someone can come down with the virus, number 2, you have something to say

P2:from what she just said now, I can say that , you know some people go from one area to another, so through that, they can partake of some food which can affect them, and some food we take outside now, now a days,

P4: through what we see now, the air stuff, there are some microorganism, they have their own disadvantage, those microorganism, they destroy some organs in our system,and through what we take in ,like in the case of Ebola now, when you say, when we expose our food to air or something, we don’t know what has already touch the food

M: if I get you, you said, what we ingest, microorganisms, and exposing our food

P3: from what they said now, the same microorganisms, they can transfer it from someone who has it to another person

M: so it can be transmitted, so, any other point from this angle, number 8, I have not heard anything from you, feel free

P4: it can be transferred from a mother to child through breast feeding

M: from mother to child

P1: I also think, what we eat, because I at times we don’t know the preparation, like I heard about this preparation of sharwarma in china, they said its not , the meat, they said they don’t really take care of it, so it may be one of the reasons why we have this cervical cancer

P9: it can be caused by use of shared instrument, if a person share sharp instrument, it can cause it

P7: may be someone, like drug abuse, the person can catch it,

M: what kind of drug abuse, is it injectable or oral

P7: it is not injectable,

M: thank you very much, now, my next question, is, as I said earlier, cervical cancer is caused by , it is sexually transmitted and before a person will become, or have this cancer, the person must have been infected with the virus, and the name of the virus, is human papilloma virus, what did I call it

All: human papilloma virus

M: that’s HPV, it is when this HPV enters the body that you know we have read about HIV, is that not, that it is transmitted through sex between a man and a woman , when this HPV comes into the body, at times, the body can heal itself and at times, it remains and becomes cancer, so before I continue, have you every heard about human papilloma virus

P7: no

P8:no

P1: yes

M; you have heard about human papilloma virus,where did you hear about it

P1: on TV

M: on TV, can you tell me , what you heard exactly about the virus

P1: I heard that it can destroy the immune system

M: that’s the only thing, that it is destroys the immune system, has anyother person heard about, you have heard about it before

P3: idea, I just want to share my own idea, it cause untimely death

M: okay, human papilloma virus causes untimely death, so this human papilloma virus is the causative agent for cervical cancer ,if you don’t have HPV in your body, you cannot have cervical cancer, so it is this HPV that causes cervical cancer, we have said we don’t know about it, except for number 9, do we know there is a vaccine, do we know,

P1: what is a vaccine

M: vaccine is a preparation that is given to protect against the virus, okay before we continue with the vaccine , can we quickly discuss on how we can prevent cervical cancer

P3: we should screen the blood

P9: if we have any sickness, see the doctor

P4: we should make sure the environment is clean

P2: be careful of what you take in

M; is it possible that you are careful of the air you breath in

P8: we should be careful of using sharp objects

P1: we should always try and go for treatments when necessary, may be if you feel that kind of symptoms , you should go for treatment, to know whether you have, that is positive

P9: tell others , if you know that you have it and how to treat it

M: so how will you know that you have it

P4: what I have to say is, if the person discovers that he or she has it, they can use that advantage to ridicule the person

M: so you are thinking of stigmatization, so the person should not tell people , so what should the person do

P4: the person should just stick to what the doctor says and continue using the medications and continue praying

P2: if the person feels that something is wrong with his body any time, the person should go for check up and any drug given to him by the pharmacy should be taken the way it has been specified, the person should also take care to make sure that the thing does not affect those that are living with her

P1: based on what he said, I think because of some illiterates, because some people they are not, instead of keeping it to oneself, you can tell another person, the person will know how to tell the person to go to the hospital or to see a doctor, so the person should not keep it to his or herself

P4: if they are illiterates, they may want to use that , to harass the person, you will hear, see that woman there, don’t let me see you near her, she has this and that

M: we are not here to fault opinions, everybody has a right to their own opinion, so after we get this things, now do we think, do we know If there is a vaccine for cervical cancer,

P1: if we know that someone has this thing, you have to make sure the person gets medical attention, for the person to leave the virus, for ,if at al there is no cure for it right now, I will suggest the person should take vegetables, t

P2: let me just add to what he said, any thing that has negative must have positives, so, the government must ensure that , if there is a disease that enters the country, we must look for a way out ,like now, lets look at the time ebola came in, the time they knew that ebols was being transferred within the country, they look for a way out, so if government know that this disease is being transferred within the country, they should look for a way out, what is the way out, for those that are affected and those ones that are affected with the thing, should not treat themselves at home, they should look for a way out and government should help by distributing the drugs free of charge

P9: I want to say that the people who are not infected with the virus should always encourage the people who are infected with the virus because that can make them more sick

M: okay, thank you, so there is a vaccine now, matter of fact, there is a vaccine for human papilloma virus, and the vaccine is given to people that are adolescents,10 to12 girls, although boys too should take it, but when you don’t have enough resources , you can give the girls first because you know they are the ones who can have cervical cancer but if you give the boys too, they will not be able to transfer cervical cancer, they wont have the virus, is that not, so they will not be able to transmit it, so there is a vaccine for cervical cancer and it is called human papilloma virus vaccine, it is in two doses, youjsut take two shots, they give you injections twice,now, what do we think about this, do we think this is a good idea

P2: sorry , is there a research on that

M: there is a vaccine, I am telling you that there is a vaccine, if you take the vaccine, you will not be infected with, you will not have HPV

P2: for the people that are below 40, you said its common for those that are 40 upward

M; that’s when the cancer will manifest but the HPV can be prevented, you can become infected with the virus, anytime, even if it s a ten year old that has sex, she can become infected with the virus, the virus will be in the body,but the cancer will not manifest until the person is 40 and above, [what if the person gets the vaccine] someone who is above 40 may not be able to get the vaccine because the vaccine is targeted to those who are not sexually exposed, who have not had sex, the vaccine is majorly for adolescent ,that’s why they said 10 to 12, but if the person is older and has not yet been exposed , the person is secured for life,

P: do we have the vaccine for people who have had the disease and over 40

M: we have a treatment plan for those who are ver 40, can we now continue, with what we are doing, okay, so If we can have the vaccine, do you think it is a good idea

P4: it will reduce death in women

P3: it will save their lives

M: number 6, I have not heard from you since, do you think, it is a good idea to have the vaccine, do you think it is a good idea, is it something good, do you think it is okay

P6: no

M: why

P6: it is not good idea

M: why do you think it is not a good idea, it is your own opinion, feel free to express yourself, why do you think it is not a good idea, are you Yoruba, you can express yourself in Yoruba, kilode ti o se so wipe kin se oun to dara, okay, let me explain again, we say that this cancer is killing, and there is a vaccine that can help protect against the cancer, do we think it is a good idea

P6: yes, it is a good idea

M: why is it a good idea

P6: if the person goes to the hospital and the doctor sees the person, they will know what to do

M:okay so, you think it is a good idea

P9:it is good , it will not let people die untimely

P7: do you think it s a good idea,

P3: it cures illness and decrease birthrate

M: nobody wants to die

P9: its good and its not good because there is nothing for those who have the cancer

M; there are sometimes when people are not careful, they just continue to treat the disease out
