# Supplementary material for: “She must have been sleeping around”…: Contextual interpretations of cervical cancer and views regarding HPV vaccination for adolescents in selected communities in Ibadan, Nigeria
Source: PLoS One. 2018 Sep 17;13(9):e0203950. doi: 10.1371/journal.pone.0203950 (PMC6141096; doi:10.1371/journal.pone.0203950)
Supplement: S1 CaCx data — (ZIP) [file pone.0203950.s002.zip › FGD JUNIOR BOYS PUBLIC.docx]

**TYPE OF PARTICIPANTS: JUNIOR BOYS public school**

**TYPE OF INTERVIEW: FOCUS GROUP DISCUSSION**

M: so you will have to speak up, can I record the conversation, this tape recorder can not pick non verbal responses, so I will ask again , can I record the conversation

All: yes

M: and do I have the permission to go on with this interview

All: yes

M; you were not forced to do this

All: yes

M: thank you very much, as I said earlier, my name is ………., and I have my colleagues here too, the person who will be writing our responses is…….. and my other colleague who will be overseeing logistics here is ……….., so we will all be making contributions to what we have come here to discuss, so I will want us to really say what is on our mind, do we understand

All: yes

M: so what we have come to do is, we want to know the understanding we have, we want to know what we know about cervical cancer, human papilloma virus and human papilloma virus vaccine, so where I will start from , this numbers that we have been given, let us put it in a visible place so that if we have anything to say, we will just bring out the numbers, it is not that we will all be talking at once, we cant all be talking at once, so if we want to talk, make sure you raise your hand, then you say, I number 1, this is what I have to say, I number 2, this is my own contribution, so that is how it is going to be , do we understand, there is no need for us to murmur or have another group discussing while there is an ongoing discussion, who ever has something to say, will just have to raise the hands, do we understand, do we understand

All: yes

M: if you whispering or murmuring, it will not capture your voices but if you speak up like I am doing then it will capture your voices, my first question is, have you heard about cancer,

P10: I have heard about it, they said it use to affect people on their breasts, that is what I know about it

M: okay, is that what you have heard, that it use to affect people on the breast, is there any other person who has heard about cancer

P2: they said if one is using all these bleaching creams that one can come down with skin cancer

M: so if one is using bleaching cream, the person can have skin cancer

P4: they said if one is putting money in the underwear , it can cause cancer

M: so if someone is putting money in the underwear, the person can have cancer

P3: those women, who are bleaching and are not maintaining it, it can cause cancer,

M: if women using creams don’t maintain it, it can cause cancer, please mention your numbers before you say anything,

P: if a man and a woman have sex, there can be cancer

M: okay, so if a man and a woman have sex, they can have cancer too, do we still have someone who wants to talk,number 5, what about you, you have never heard of cancer, number 6, number 7, you have not heard of cancer before

P7: I hear about it

M: that’s what I want to know, you hear about it, where do you hear about it

P7: in our place

M: where is that?

P7: Oke Aremo

M: at Oke Aremo you just hear that someone has cancer, so what do you think it is

P1: it is a deadly disease

M: it is a deadly disease, where did you hear that it is a deadly disease, I cannot really hear you, what kind of cancers do you know?

P7: if one has bleached and the body is smelling

M: so if one has bleached and the body is smelling, is that cancer, where did you hear about it, where, is it when people are discussing or is it on radio or television?

P7: when people are discussing

M: when people are discussing, number 3

P3: when a woman has bleached and she is pregnant, when she wants to deliver, it won’t be easy for her, she will have cancer because her skin is too soft

M: it won’t be easy for her because her skin is too soft, where did you hear that

P3: I heard it on the radio and I also heard it from people

M: you heard it on radio and you also heard from people, number 2

P2: those who smoke marijuana, that as they smoke that marijuana, that it destroys the organ that makes them breath, their lungs, it destroys it , and when it is destroyed, it will become black, it will cause cancer for them

M: those who smoke marijuana do have lung cancer right?, it causes cancer, okay, do we have any addition, do we have any addition, everything we have said is good, do you have something you want to say, number 8

P8: when drinking alcohol, it can cause cancer?

M: so what kind of cancer is that[ I don’t know] but you just know that when you drink alcohol, you can come down with cancer, everything you have said is good, someone mentioned breast , it means that one can have cancer there, another person mentioned lungs, it means one can have cancer of the lungs, is that right?, someone mentioned skin, if you are using bleaching cream, it means one can have skin cancer, so that cancer , it means it can affect every part of the body ,is that not? Cancer can be on the skin, or lungs, or breast but there is one that affects the entrance of the womb and that is why we are here today, cervical cancer, where did I say it affects?

All: the entrance of the womb

M: it is that entrance of the womb that the cervical cancer affects, has anyone heard about cervical cancer? That this person had cervical cancer, have we heard anything like that, you have never heard of it, that this person had cancer at the entrance of the womb, we have not heard of it, have we heard of it, no, okay, so this cervical cancer is peculiar to women, do we understand, and how can we know if someone has cervical cancer, it is women that are more affected, and that women is 40 and above, what did I say

All: 40

M: that person is 40 and above, when the person is 40 and more, that’s when they are usually affected and how do you know, the person will be bleeding from the private part, it is not menstruation and the blood will have a foul smell, it may come with back pain or not, the person may loose weight and if the person is left and no intervention occurs, the person can die. so have we heard anything like that , may be someone, my mum told me about someone, or they were talking about someone who had it in our environment

P3: one of our church members complained of bleeding and had to go to UCH, when she came to UCH, I don’t know if the issue was resolved or not

M: the person was bleeding and was more than 40?

P3: yes, she was more than 40

M: it may be, it may not, the person went to UCH and the person is still alive

P3: yes, she is still alive

M: may be the person has been treated for the cervical cancer, is there any other person who has heard or has had experiences of someone with cervical cancer, no 3, has given us one, who else has heard or has seen experiences

P10: we have seen it in a movie

M: in a movie, what happened to the person,

P10: the person was raped and had cancer from the rape

M: she now started bleeding from the rape, that’s not cervical cancer, you see that cervical cancer, one may have been infected with it while young, it is when the person is now 40,that the bleeding will start, the person will have been infected with the virus that causes it while young, but it is not that it will start immediately

P2: the one I heard is that cervical cancer blocks the uterus

M; it blocks the uterus, where did you hear that

P2: it was someone that works in UCH that told us,

M: someone who works in UCH told you that cervical cancer blocks the uterus, have you seen anyone with that experience or you heard it,number 1, you want to say something

P1: ma, please I want to ask, if a woman who has children has that cancer, will it affect the children she has

M: I didn’t say it is from the mother to the child, is it from the mother to the child

P1: you said it will have been in the body since the person is young

M: the reason I said while young is that, as we are now, adolescents, some of us may have been exposed to the virus but it will not manifest yet, it will hide itself in the person’s body, it will be waiting for the time the person will be more matured, for some their body heals up while some others, it waits for them till they are 40, you know that at a point in a woman’s life ,menstruation ceases, so when these women are approaching menopause, they will then start to experience bleeding, so it will continue like that, so that is it, I didn’t say from mother to child, whoever will have it, would have had it, some heal up , some don’t, if the body is not able to fight it itself, then it manifests as cervical cancer. So has anyone had this experience, you said you have seen the experience, where did you say you saw it

P: it is from an animal

M: what kind of animal

P: she caught mumps from the animal, she had mumps…. inaudible

M: cancer , the animal had cancer, I cant really hear you, okay , he used to remove ticks from the animals, so she got it from there, when they got to UCH, they said its cancer, the cancer, was it cervical cancer in UCH, they didn’t tell you, okay, number 5, what do you have to say, what is the meaning, okay, this cervical cancer, what do we think can cause cervical cancer,I have asked if we have seen it before, some of us said we have seen it, someone said one can be infected through animals, do we have any other person that knows what can cause cervical cancer, what can cause, the person who had it in your church , what did they say caused it

P2: I don’t know,

M: they didn’t mention that this is what caused it

P: inaudible

M: the person had it a long time ago but did not tell anyone about it, our voices are not audible, I can barely hear you though I am sitting so close, please lets speak up, what do we think can cause cervical cancer, number 2

P10: when one is young and the person is having boyfriend, it can cause cervical cancer

M: how do you mean, having boyfriends, in what way

P10: if she is sleeping with the boyfriend, she can have cervical cancer

M: so you are saying if one is having sexual intercourse when young, the person can have cervical cancer, who else has an addition, number 6, you don’t have anything you add, number one, hmm, number 4, Number 7, please talk , the question I asked is, how can one have cervical cancer, how can one have it, number 7, how do you think one can have cervical cancer, what you think, like the way this cancer is mentioned, one can have it through this way, one can have it through that way, how ? okay , you don’t have anything to say, if you don’t have anything to say then you will say it, I don’t have anything to say, you will not just keep mum, saying you don’t know is a response , number 2

P2;

M: so how can we prevent this cancer ,number 10, you said one have have it through sexual intercourse, so how can we stop it, how can we prevent it, how do we ensure that people don’t have it, we are all going to say something, number 9, what do you think we can do to prevent this cancer

P10: one should go to the hospital

M: one should go to the hospital, when you get there, what doyou want to do

P10: they will do test for the person

M: so the person should be tested. Thank you, number 9, what can we do to prevent this cervical cancer, if you don’t know, you will say that, I don’t know what we can do

P9: I don’t know what we can do

M: number 3, you have something to say

P3: sanitation,

M: how do you mean sanitation?

P: we should be taking care of our bodies, ensure we are clean, drink good water, eat good food

M: that is not sanitation, that is hygiene

P2: when one is sleeping , one should take care, avoid mosquito bites and all the things that can make one sick

M: okay so one should be careful all the time, one should take care should ensure a clean environment all the time

P3: one should not just be using cream

M: one should avoid using cream, what kind of cream? Or one should just avoid creams, okay , number 6, number 6

M: thank you very much, we have discuss how one can have cervical cancer and how it can be prevented, so I will just brief us on a virus I call Human papilloma virus, what did I call it

All: Human papilloma virus

M: it is this virus that causes cervical cancer and this human papilloma virus cannot be seen, what did I say

All; it cannot be seen

M: so this human papilloma virus is transmitted during sexual intercourse, what did I say

All: it is transmitted during sexual intercourse

M: so has anyone heard about human papilloma virus, has anyone heard about it, if you have not heard about it, you will say it, that we have not heard about it

All; we have not heard about it

M: so there is no one who has heard about it

All: yes

M: okay, so it is this human papilloma virus that causes cervical cancer, if one has sexual intercourse when young, if it is in the body of the person one had sex with, then the person can be infected and it will be hiding in the body, when it is in the body, some people if their defence system is very strong, it will fight, their health will be restored, while for some others, it will not leave until the person is 40 , so when the person is 40, it may then become cervical cancer, they will now be seeing symptoms of cervical cancer, and some people will not see the symptoms, now there are things people can do to prevent this cervical cancer, you have something to say

P: so you said that the woman will be infected with cervical cancer, how about the man, will the man still have the cancer infection

M: the woman may develop the cancer, the virus will still be in the man, I didn’t say the woman will be infected with cervical cancer, what did I say the woman will be infected with

P2: human papilloma virus

M; it is the virus that one can be infected with, not the cancer, so it will not leave the man, because it is there already, how is it supposed to leave the man

P2: this cervical cancer, can it lead to death, like HIV?

M: it can lead to death, if one has the cancer and did not have proper care, it will eventually lead to death, so the cancer will be in the body, now if people can have this cancer, if we now say there is a vaccine that will help to prevent the spread of this cancer, what do we think about it? Do we know what a vaccine is

P2: vaccine is like immunization, something you take to give you immunity

M: so it is like immunization, that’s number 2, does anyone else has something to say, no one else, so it is only number 2, that has something to say, do we all agree with number 2, okay so we go with number 2, vaccine is like immunization, so if we hear that there is a vaccine for HPV, that if you take this vaccine, you will not have this infection, do we think it is a good thing, if we say there is a vaccine like that , is it a good idea

P2: it is a good idea

M: why is it a good idea,

P2: because it will prevent people from having the infection in the future, it is not for today, because one cannot say the person one will marry, and when one has the vaccine, it will push all these things out later, so it is a good idea, if one can take it now, it will help in the future

M: okay , so if one takes it now, it will work in the body in the future, number 4

P4: if one takes it now, it will help one not to have it in the future,

M: if one takes it, it will not let one have it in the future, number 5,

P5: I don t have anything to say

M; what about number 6, number7, is it a good idea

P7: yes

M: why is it a good idea, why is it a good idea, you just know that it is a good idea, why is it a good idea, number 1

P1:it is a good idea because once one can have it now, it will help in the future

M; number8, why is it a good idea, you understand Yoruba if not English, number 9, why is it a good idea, if they say there is a vaccine that if you take the vaccine, you will not be infected with the virus. Why did you think it’s a good idea, is it a good idea, number 6, number 5. Why is it a good idea, or is it not a good idea number 6,, why is it a good idea

P6: when one has that cancer thing and one takes the vaccine, it will push the thing out

M:thank you for all your comments, now, I say that there is a vaccine now, and its 7000, each person take doses, now that we know that the vaccine is available, what do we think can make people not want to have this vaccine

P2: if people are not given a seminar to make them understand the benefits of taking this vaccine , they may think the vaccine is meant to harm them

M: okay so people may not want to take it because they think it is for something else

P2: if someone takes it, others may think that the person has it and the person does not want to be exposed so if there is a seminar, it will make things better

M: I didn’t say the vaccine is for those who have cancer already, the vaccine is given before one begins to have sexual intercourse with a man or a woman, when someone is like 10 to 12, before the person has sex, it is for virgins, that’s when you get to have cancer, I didn’t say it is for someone who is already infected , it is for someone who is not infected and we said it will prevent the person from having , is that not

All: yes

P2: people may not want to have the vaccine because people may be in a group, and in that group, nobody is taking that vaccine, and you go ahead and take the vaccine, they may think it is because someone is infected with the virus,

M: so what can make people not want to take it what can stop people from being vaccinated

P: if they don’t have the money that they will take to the place where they will be vaccinated

M: okay , you have heard about it personally now, what can stop you from being vaccinated, number 6

P: I don’t have anything I want to say

M; you don’t have anything in mind what can make you not take it now, I didn’t say people, you have heard about the cancer, what can stop you from having the vaccine

P6: because people will think may be one has cancer already,

M: people don’t believe that

P6: like as we are now, if someone should leave among us and is going to get the vaccine, people will be like don’t go , that thing is not good, or may be the person is infected , maybe that’s why the person will not want to have the vaccine

M: so if people are discouraging, that can stop people from having the vaccine, because of what other people are saying, thank you, number 5, what can make you, say, okay, I have heard about the vaccine, the vaccine is 7000, and If I have 2 doses, , I will not be infected with cervical cancer, what will make you not want to have the vaccine inspite of this information you have already

P6: if I know that they are giving people and I tell them that I am going to have the vaccine, they can start making fun of someone that I already have cancer

M: okay, so because they will make fun of you right, okay, who was raising hands the other time, number, is that number 3

P3: if one does not have the money, the vaccine is expensive, for some of us that don’t have the money, they can think the money is too much

M: they can think the money is too much,

P1: if one is not properly enlightened about it, that if they give the vaccination, some people think it can cause something else in the person’s body

M: that it can cause something else in their body

P: yes, some people even think the needle, they have used for someone that they are going to give the person

M; but once they are sure it is not the same needle,will they still take it

P: yes, they will take it

M: number 8, what will make you not want to take the vaccine, after you have the information about it, is your mind here, you can hear, you can speak, you have something to say

P: some people they don’t like taking injections because they feel that it may cause something else in their body

M: some people may think it will cause something else in their body

P6: some people may really want to take but because there is no money, they cannot take it

M: so if there is no money, it is a source of concern, all of us here, we have talked about what can make us have it and what can stop us from having it, how many of us, will have our parent consent to us being vaccinated or not, that vaccine you talked about, you cant have it,, or that vaccine you mentioned, you can have it, and this is the reason, if you say my mummy, please give me 14, 000, I want to get this vaccination, why will the mother refuse

P; the mother may be thinking he is lying

M: what if the mother heard about it from the radio, what will make the mother not to have

P4: the mother may still not believe

P6: the mother may not believe that they asked them to bring 7000 for the vaccine, they may think that he is just trying to collect money from them to spend anyhow he likes, but if they know, they will give it because, it is for the health of the person too

P2: they can think that if he takes the vaccine for cervical cancer,that it can lead to something else

M: they can think that it will lead to something else, it can have side effects

P2: yes

M: so because of the side effect, they may not want to have it, but if they know the kind of side effect it has, they will take it, okay, so far, the only side effect experienced when given this vaccine is that, it may make the area where the injection was given swollen, does that seem too much,

P5: no, for some others, it may be that they cannot take injection

M: but these are grown up children, as you are now, if you get the vaccine, will you cry,

P: not likely

M: thank you very much, so if this vaccine will be made routine, how can we ensure that adolescents have access to it , and that every young person like us get the vaccine, what can the government and every other person do to ensure it is a success

P1: they should go to radio stations, go to the internet, they should tell everybody, they should let people know, communication sha, they should let people know, so and so is out there, and these are the symptoms, then they should let people know that there is immunization, communication, they should really explain, they people will come out

M: number 3, you have something to say

P3: they should let the money come down, if they want people to have the vaccine so that it would be easy

M: they should reduce the cost to make it easier for people, number 4, what do you have to say

P4; they should take it schools, like that meningitis

M: so they should take it round and give to people like meningitis, okay, number 5, what can they do to help young people get the vaccine, you are less concerned, if they want you to get the vaccine,, what can they do to you to help you get vaccinated

P5: they should bring down the money,

M: they should bring down the money, so if the money is reduced, will you be able to get it, will you be able to get it, it is made for people of your age, is that not, will you get it if the money is reduced

P5: yes

M: okay , number 6, how can you be helped

P6: they should bring down the money, because there are lots of benefits if they bring down the money, the benefit of the vaccine

P7: they should reduce the money, if they reduce the money, people will be more encouraged to come out and take the vaccine

P8; If they bring an identity to show that it is from the government

M: identity, so if it is not from the government, you will not take it, but if it is from the government you will take it

P8: then they should let us see people that have had the vaccine

M: okay, so they should like bring people, that this person has had it, and nothing is wrong with the person, then you will get the vaccine, number 10, how can you be helped to get the vaccine, if it were to be made routine

P10: if I see the way people are getting the vaccine, the way they take the vaccine, then I can be encouraged, when I see that a lot of people have been vaccinated

P2: then they should go to the villages, and tell them too, take it to them, there are lots of people there, that they should meet and talk to about the vaccine

M: so if they go to the villages and let people know about the vaccine then they will take it too

P3: they should put the vaccine in centres

M: what is that, do you mean govt owned hospitals close to us

P3: yes

M: who else has something to say

P2: the government should establish organizations and hospitals where they can produce that vaccine for cervical cancer, and they should let people know too

M: so they should give them public enlightenment and start up places where people can access the vaccine, thank you, number 5, number 4, number 9, you have been hiding your face from me,now tell me, what addition do you have to everything that has been said, hmm,hmm, I know that your voice will be the loudest in class, may God help you, if we don’t have anything to add,then we have come to the end of this interview, lets clap for ourselves.
