# Supplementary material for: “She must have been sleeping around”…: Contextual interpretations of cervical cancer and views regarding HPV vaccination for adolescents in selected communities in Ibadan, Nigeria
Source: PLoS One. 2018 Sep 17;13(9):e0203950. doi: 10.1371/journal.pone.0203950 (PMC6141096; doi:10.1371/journal.pone.0203950)
Supplement: S1 CaCx data — (ZIP) [file pone.0203950.s002.zip › FGD JUNIOR GIRLS private.docx]

**TYPE OF PARTICIPANTS: JUNIOR GIRLS private school**

**TYPE OF INTERVIEW: FOCUS GROUP DISCUSSION**

M: so I will be recording , do I have your consent to go on with this interview

All: yes ma

M: and the permission tp record this conversation

All: yes ma

M: thank you, my name is …………I want to know what you know about HPV, cervical cancer and cervical cancer vaccine, so I want to ask you, have you heard about cervical cancer

All: no

M: before you answer mention your number

P6: no

M:okay, any other person

P10: no

P7: no

P4: no

P3: no

P1: no

P5: no

P8: no

P2: I have not heard anything about it

M: you have not heard anything about it, but have you heard about cancer before

aLL: yes

M; you have all heard about cancer

All: yes

M: so what have you heard, your number first

P6: I am number 4, [no?] number 6 sorry, I heard about cancer , breast cancer, some people that are putting money into their breast

M: you hears about breast cancer, some people that put money into their breast, so who else has heard about cancer, you want to say something , number 3, you have not heard anything

P10: people that are putting phone inside their breast

M: so people that put phone into their breast may have breast cancer, apart from that, is that the only type of cancer you know about

All: yes

M: that’s the only type of cancer you know, okay then, I want us to know that cancer can spring up from anywhere, it can do what

All: it can spring up from anywhere, cancer is one thing , it is a growth, you know we have cells in our body and the cells ,from time to time they replenish , now it can happen that one cell will just decide to grow more than the normal size, that happens,and it can be benign or malignant, benign means it isnot gong to do you anything, malignant one is the cancer,it can happen anywhere, it can happen on the head, on the leg, it can happen in the mouth, you canhave mouth cancer, throat cancer , skin cancer, eyes cancer,brain cancer, breast cancer, you can have cancer on the male’s reproductive organ too, that’s the prostrate cancer, so different kind of cancer can spring up, but the one we have come to discuss about is the cervical cancer , the cervical cancer, the cervix is part of the woman’s reproductive organs, have you done it in science

all: yes

M: down the womb ,we have the cervix, the cervix is like the entrance of the womb, when cancer happens to a woman there , it is called cervical cancer, it is called what

All: cervical cancer

M: so what do you think can cause the cervical cancer, okay before we go on le me just tell us how it happens, we said don’t know, but we may have seen it, when we see someone that is older than 40, the person is 40 years old and above , the person is more than what

All:40 years old

M: and the person is bleeding when it is not menstruation, it is not period, it is not menstruation ,the person is bleeding and the blood is smelling, the blood is what

All: smelling

M: it is smelling, the person is having back ache, the person is loosing weight and all those things, have we seen anything like that before, have we heard anyone that has that kind of sickness like that before

All: no

M: no, we have not seen or heard anything like that,okay so that’s the symptoms of cervical cancer, what do you think can make a woman to be bleeding like that

P6: If that person wants to give birth to a child

M: but this person is not giving brith to a child, she is not a pregnant woman, the person is almost entering menopause or even menopause,you know what menopause is, that’s when a woman stops menstruation , the person is not menstruating, she is not pregnant, she will just be seeing blood, the person is may still be menstruating normally, if the menstruation has not ceased, but it is not menstruation and it can be prolonged, may be like bleeding for a month or more, have you seen anything like thay

P7: is is abortion

M: it is not abortion , but you think abortion can make a woman bleed, have you seen any one like that, you have never seen or heard , may be someone has told you what happened, that you know, may be the person was passing blood, may be in your church or something ,something, you have not seen or heard , so what do you think causes cervical cancer

P6: may be the person has sexual intercourse and the person has a disease

M: okay, if someone has sexual intercourse and the person has a disease, the person can have cervical cancer, thank you, who else has something to add

P3: if someone is taking too much alcohol.

M: too much alcohol, if someone is taking too much alcohol,the person too can have cervical cancer, thank you, who else,is there anyother person that wants to add to what number 3 and number 6 said

P10: through sickness

M: what kind of sickness

P10: any kind of sicknes

P3: if someone is having other people then the person can hvae it

M: okay, so if someone is having other people, like multiple sexual partners, apart from the person the person married,so it can cause cervical cancer too, you are nodding, you agree with what she said

P5: maybe that person has contacted disease from somewhere

P2:may be you want to urinate, and you are putting your butt inside the gutter, or you just bend down anywhere,it is very bad, they say it can cause tetanus

M: so if you want to urinate and you don’t care for your body well especially in the gutter, you can have cervical cancer, okay so we have been discussing things that we know can cause cervical cancer and we have talked about , so how do we think we can prevent all these things, what we have said, how can we prevent cervical cancer,p7

P7: we should not have sexual intercourse

M: is it that we should not have sex at all, if you marry, you will have sex now, is that not,what kind are you talking about

P4: the person should seek medical attention

P2: if someone has this cancer, she should go to the hospital and see the doctor

M:yes,the person should go to the hospital to seek medical attention, okay, p7

P7: to avoid HIV/AIDS,

M: to avoid, I don’t understand,

P7:we should not move close to someone that has HIV/AIDS

M: it is not HIV that causes cervical cancer now, I will still tell us what causes it, okay, so this cervical cancer that we have been talking about, we have talked about what we think can cause it, and what we think , how we think we can prevent it, cervical cancer is caused by a virus, It is caused by a what

all: a virus,

M: that virus is called human papilloma virus, the human papilloma virus, is that virus that causes cervical cancer, and how will someone have it, when someone has sex with someone that already has it and the person has sex with that person, the person will become infected, but when the person, may be the person had sex at 10 , or 9 or 8, or 15, or whenever, it will not become cancer until the person is 40 years old, until the person become what

All: 40 years old

M; the virus will just be in the person’s body gradually developing ,it will not do anything, for some people, the body will heal itself while for other,it will not be, its when they now get to 40 and above that it will now be, it will now become cancer, so that cancer is caused by what what did I say is the cause

All: virus,

M: what kind of virus,

All: human papilloma virus

M: okay , has anyone heard about the virus, no body has heard about it, so this virus there is a way you can prevent it, there is a vaccine and it is called human papilloma virus, that vaccine is used to prevent cervical cancer, have we heard about the vaccine before?

All: no

M: you have not heard about the vaccine but you know what a vaccine is, what is a vaccine

P2: it is like medicine

M: it is like medicine, so what does it do, okay, vaccine is not used to cure illness, it is a form of preventive medicine, it is something you put in the body, like all this small babies now, taking a baby to the hospital for immunization, have you heard that before

All; yes

M; so immunization is getting the vaccine, so do we know what immunization is, it is when you are protected against something, they vaccinate you, give you that thing, to stop that thing from coming into you body, do we know, have we heard anything about HPV vaccine?

All: no

M: okay, so the vaccine is available now and everybody can take it, it is for adolescents, both boys and girls can take it but those who are between 10 and 12 , are we getting me, those who are between 10 and 12, particularly who have not had sex, but if you are more than 12 and you have not had sex, you can take the vaccine and the vaccine , you take it twice , two doses, when you take those two doses , you will be protected through out your life time , you will not have the infection from HPV and you wont have cervical cancer, and the vaccine, a dose is 7000, two doses is 14, 000,

P3: it is too costly

M: number 4, why did you exclaim

P4: the money is too much

M: the money is too much, okay, we will talk about it , but you take two doses and you are protected for life, what do you think are the advantages of taking the vaccine,

P6: it will help you fight against the virus

P5: you will not be able to contact the diseases

M: you will not be able to contact the disease, it’s a disease, and its the HPV that causes cervical cancer, it does not protect you from other diseases, okay so ,what are the other advantages, number 7

P7: to fight germ

M: what kind of germ, what germ are we talking about, [virus], what kind of virus

P7: human papilloma virus

M: that’s the only one it works for , it does not work for other things, did I say it works for HIV[ no] it does not work for tuberculosis , it does not work for other germs, it s only HPV that it works against, so what do we think are the advantages, I want to hear from you number 9, do you think the vaccine is a good thing?

P9: yes

M: Why is it a good thing?

P9: it will cure the virus

M: cure, cure, it does not cure, it will not let you have the virus, thank you,

P6: please how do you get the virus

M: the virus is passed through sexual intercourse, I think I said it now, when you have sex with someone who already has the virus, do you think it is a good idea to have the vaccine, do you think you can take the vaccine,[ yes] , so why will you not take the vaccine, what will be your concerns, what will you be think about,if for instance, someone calls you to come for the vaccine, what will come to your mind, what will you be thinking about,

P6: it will also have it own effects

M: you will think about the effects, aside the effect, is there any other thing you will think about before taking vaccine

P6: it may not work for another person after paying the money

M: so you feel your money may be wasted

P4: if the person does not have the money, and may be it will affect the children they born too

M: okay so, you will think about the money and if its genetic/ transferrable

P6: it may destroy something in the body

P5: if the person did not use it according to what is in the carton , can it cause another thing

M: the person is not even going to take it home, when you go to the hospital for vaccination, they give you just what you need, one dose, that’s all, they wont give you more than you need, does that answer your question, it is not like benylin with codeine or paracetamol syrup. It is not like that, it is just one dose and its premeasured, they just give the person, why will you not take the vaccine

P6: some people will not want to take the vaccine because they will feel it cannot do anything to them because they have never had it

M: but its for the future now, it s not for now, when will the cancer come

All; its in the future

M: you just take the vaccine now, it will protect you later

P5: if it’s a pregnant woman, she may think that she should not use it,it may affect the baby inside

M: we don’t give pregnant women, who did I say we can give

All; 10 and 12

M: is it likely a woman is pregnant then

All: no

M : and I said the person must have not had sex, can someone become pregnant without having sex,, okay so if the vaccine were to be made routine and given to everyone, how can we make sure you take the vaccine,what do you think can make you take the vaccine

P6; if you have the money, when you have the money

M: so what can we do about that

P6: if there is someone who can give me the money

M: if you see someone who will give you the money , you will take it

P4: if I see someone to pay for me,I will take it, or if its for free, I will take the vaccine

M; we are all laughing, is that what we want

All: yes

M: they should give you for free

All; yes

M: you will take it ,if its free

All; yes

M: will your parents allow you to take it,

All: yes

M: what do you think will be your parents reaction if you say you want to take the vaccine

P4: if you discuss with them , they will agree

M: okay, lets say you have discussed with your parents and they agreed already, how do you get the vaccine , how do you hope to get the vaccine

P3: we don’t know if you will come back

P6: you give us your phone number or you go to the school authority

M: did I mention that I brought the vaccine?i didn’t bring the vaccine, I am not even in position to give you the vaccine, am not the right person to do that,so how do we getthe vaccine, what do you think we can do to get the vaccine

P4: visit the hospital,

M: so if you visit the hospital, you get the vaccine, is that right, so what if the vaccine is not in the hospital

P6: I am sure the vaccine will be in the hospital

M: you are sure, your are just sure that it will be there

P5: everything is about money

P4: we can go to the government since they are the ones who help us to provide medical treatment, we can tell them, please can you help us to provide this vaccine

M: okay, that’s one way, but how do you intend to get to the government

P7: we can send it through letters

M; we also mentioned that if we let our parents know,they will support,how will they know

P: , they should do leaflets

M: how about parents that cannot read

P10: if they hear it in the radio

P5: they should advertise

P8: if they give them the leaflets, their children can help them to read it

P6: and some parents will not agree, they will think that the drug will affect

‘

P10; the parents may not have time to read it

P2: if you tell them the benefit of that vaccine, I think they are going to read it

M: so how do we ensure that adolescents get the vaccine

P3: telling the hospitals too , so that all of them can have it

M: what about those who are living in far away places, where there are no hospitals, what can be done for them

P6: they should get a bus and go there

M: what will the bus do, is it to take the vaccine to them or to bring the people

P6: they will take the bus there to announce to them, then they can give them there

M: okay, so we have mentioned some things, is there any other thing we want to add

All: nothing

P4: if some people knows about it, they can help with the money

M: its not generally affordable, how can we make it affordable

P5: may be we should reduce the money

M: reduce the money to like how much

P5: 500

All; ahh, don’t even pay

M: so if the vaccine is reduced to 500, everybody will take it

All; yes

P4: aunty, is the vaccine with you

M: no, I don’t have the vaccine, do we have any other thing to add

P4: no

M: okay , so if we don’t have naything to add , that will be the end of the session

P4: please aunty , how can we get the address of the place
