# Supplementary material for: “She must have been sleeping around”…: Contextual interpretations of cervical cancer and views regarding HPV vaccination for adolescents in selected communities in Ibadan, Nigeria
Source: PLoS One. 2018 Sep 17;13(9):e0203950. doi: 10.1371/journal.pone.0203950 (PMC6141096; doi:10.1371/journal.pone.0203950)
Supplement: S1 CaCx data — (ZIP) [file pone.0203950.s002.zip › FGD OLDER MEN 45 and ABOVE.docx]

**TYPE OF PARTICIPANTS: OLDER MEN 45 AND OLDER**

**TYPE OF INTERVIEW: FOCUS GROUP DISCUSSION**

M: I need to ask from us before I start recording ,that’s why I asked, do I have the permission to conduct this interview, that we are not here out of compulsion to be here, that we came here of our own accord,

All: yes

M: I want us to answer, so that the audio recorder can capture our voices

All; we came here by ourselves

M; thank you very much, as I said earlier my name is ……….., and my colleague here is …………., and the third person with us is …………., we are here to do a study on our knowledge and understanding of cervical cancer, human papilloma virus and human papilloma virus vaccine, may God help us, so the first question I will like to ask is that, have we heard about cervical cancer? Have we ever heard about cervical cancer

P1: I am number 1, I have heard it on television as well as radio, and again, I have read it in the newspaper before

M: you have read it in the newsprint, you have heard it on tv and radio, okay, has any other person heard about cervical cancer

P2: I have also heard about it, I have not seen it, but I just know that there is something like that, being that I am not a woman, but then we have heard about it

P4: I am number 4, I have heard it well, I heard it on radio as my colleague said, I have also heard it on TV,

M: Okay, number 5,

P: you omitted number 3

P3: I have also heard about it, is jejere different from cancer,

M: it is the same thing, it is the same cancer,

P4: it is cancer, but it is known as Jejere in Yoruba, you know for some of us that have not really gone to school, have you heard about it

P5: I have heard it, infact I have even seen it

M: is it cervical cancer

P5: no, it affected the breast that they had to cut the breast off and I have also seen the one that affected a man that at the end of the day, they cut off the penis, it affected a lot of things, to the extent that if he will defecate, he will have to use a pipe, to urinate and defecate, the person was really close to me, the person was my half-brother, when I went to check him in the hospital and he was to unrinate, it had odour from faeces, the internal organs were already destroyed, so, I went to check him on Sunday, and he died before the next day break, that thing affected him , so if I say that I haven’t seen it, we are only lying to ourselves,

P1: sorry to cut you, you said you are students from where

M: UI, UI

P4: please speak Yoruba because of those of us that don’t understand English can flow along

M: we are students from UI but our department is in the university college hospital,

P3: UCH here, we call it oritamefa

M; sir have you heard about cervical cancer

P6: I am just hearing it for the first time, it isn’t that we have not heard about it, we hear that this thing is around and they have had to take people who are famous, influential they took them abroad for treatment and they brought some people to UCH, some people succeed, some people did not succeed, that we have not heard about it is not possible, we have been hearing about it, and the one we know is the women, that one for men, may God just protect us,

All: amen

P6: may God have mercy on us, because we are seeing all the things we didn’t use to see, like one of our friends that said , once you live long and you don’t die early, you will see all manner of things, because we have been seeing things may God let us see good things[amen] since I was born, it is only esu that looks like the mosquitos that came out this year, I have not seen anything like it, it was really affecting farm produce then[ the mosquitos that came out this year is too much]I have not seen this kind of mosquitoes before

M: okay sir, as we were saying about cervical cancer, we have discussed the types of cancer we have seen , like the one he described it may be prostrate cancer, peculiar to men, but then, there is no part of the body where we cancers cannot come up, it comes up in the nose, in the eye, in the ear, on the skin, even in the blood , we have cancer of the blood that sometimes affect children, there are different types of cancer but the one we are discussing today is cervical cancer, how do we know if someone has this cervical cancer[phone rings]may be if we see a woman that is more than 40, and the person is bleeding, outside of the menstrual cycle, the person is just bleeding, and the blood discharged has a foul smell, the person may be loosing weight, once a person starts to experience all this things, then it calls for attention, it may be cervical cancer and it possible to have had it for a long time without knowing, do we know what may make a woman experience this kind of symptoms, what are the things that can make a woman have this kind of experience

P4: what I think can make a woman experience this kind of thing is, number 1, if a woman does not take care of herself and number 2, the woman is going out anyhow, that she is promiscuous

M: I wanted you to break it down so we all understand

P4: yes, when a woman is promiscuous, those two things, there is no way she will not have the cancer, so that is what I think can cause the cervical cancer

M: thank you sir, who else has something to add

P6: what I want to add is that, when people shout AIDS, AIDS, AIDS, this is similar to it, when you start to look for what is not lost, you are moving around, your eyes cant be in a place, anything can happen to you, and then nutrition, all the things we are eating now are not the thing we eat before, like when we were young, we used to take a lot of herbal concoctions, like when they pound somethings in the mortar, they will give us to take, if you try that with these children now, they wont even take it from you, they will be complaining, there is locust been, there is pepper , there is this , there is that and if you eat that kind of thing there is nothing that cannot happen to you, may evil not happen to us, we know ourselves that the steps we are taking now, we need to be watchful, someone I know was ill, and they said they were taking him to UCH, I said to him, you are going there to die, because they will use you to practice, that’s what I told him, may God show us mercy, some who are backed by their head and are older than that will go and they will come back unscathed, some others the moment they mention their age, they will be like what is she still doing, the person will just be given an injection and that will be the end, May God help us, there is no one that cannot happen to , that’s what I have to add

M; okay sir, do we have any other person with other things to add

P5: what I have to say is that, my wife is my only partner, but then all the things that we have now, that we eat, there is nothing that cannot come out of it, it is modernization that is making us eat spaghetti and all the things like that , Mr Biggs, indomie like worms , if you pay attention, you will see that most of these disease affect the elites more, it cannot affect people like us, because, this morning, before I left home , I took herbs, herbs from tapa and all that, going to this chemist, that chemist, you will get this thing to buy there, that’s not for me, if you will eat anything now, as elites you will double check to , is it me that I have mixed pepper with locust bean and some other things to make concoction, when I saw that I was having stomach troubles yesterday, I took some mint leaf and bitter leaf, made a drink and took it, I went to toilet peacefully , my body came back to normal, all the thngs we are eating now, NAFDAC and all those things, food with chemicals, in the olden days, all the things that we hear now, we don’t hear them, we don’t hear them , it is now, that we hear about cancer and all those things,

P4: will you believe that even the yams we eat now , is not the normal yam we are used to , they add things to the yam being planted, they have added chemical to the land they are planting now, they call it manure, fertilizer, it is chemical that they use to make it now, okay, look at the cassava we have now, what is most surprising Alhaji, there was a time that when they want to make gari, it takes how long in the sun before they fry it

P5: it can take 8 to 9 days

P4: but now, the moment they harvest it, they start to fry it in the evening, has all the chemical been drained out of that gari

P2: thank you, that one is even good, this morning, because every Monday, we have a market in cross river state, this morning , a woman will go to the farm, harvest cassava, they will grind it and still fry it the same day so that they can meet that Monday market, I said we don’t do that in Yoruba land , that in Yoruba land, people harvest cassava and leave it for 8 days before they will fry it, so there is someone in that place, she is Yoruba, I will tell her, please I am going to the south west and I need gari, don’t do this style they do here for me then number 2, all this thing that we are seeing now, it is because of the fertilizer we are using, because everything we eat now is fertilizer, good tomatoes, fertilizer, other things, fertilizer, there is no, sorry to say this, you look at it, [ dragging of chairs, a new participant comes in, greetings are exchanged]Then, if they plant things, it grows by itself there is no need to fret, there was no fertiliser, you will see that now, if you have yam, there is no how it is matured, it will spoil itself after a while, its fertilizer that is causing all of this problem

P3: what I have to add is that, this maggi they add to stew, is causing problem for us, in our body, because,

P4: speak up, this thing will not capture your voice

P3: let it be, it will even capture it better

P4: I hear you

P3: because I think its because of those things they sell to us, it is the cause of this problem , it is eve the one that is causing more issues, when we were young, my parents don’t add maggi to their stews, they use onions with leaves, and [ then] when we eat that stew, we will so much enjoy it , now we don’t have all of that, it is that maggi that we have, and all this things, is it good for people our age, are we supposed to eat those, is it something they are supposed to add to our stews

M: we met this things that way,

Participants laugh

M: I may not be able to answer the question but may be

P: me that you are looking at, I eat locust bean everyday, I eat raw bitter leaf, I also eat yanrin because of my eyes, its because of this things

M: so how can we prevent this cervical cancer

P5: greet alhaji and update him on our discussion

M; welcome sir, we have been discussing about cervical cancer and we want to know, how we can treat it, and the things we can use in its treatment be it orthodox or traditional , and any other thing you may have that can help this interview especially as it relates to how we can reduce the incidence of cervical cancer, you will number 7, I don’t know if you have anything to add to what we have discussed about what you think is the cause of this cervical cancer

All: your number is 7, that’s your name

P7: for what I have to add, we thank God,[Alihamdulilah] all this cervical cancer issues that we have discussed, what we are experiencing today , is a product of our society , it was not like this in the days of our fathers, starting from the pepper mill, there is no way, you will be milling a pepper that its iron will not mix with It, and we eat it all together, then those who gave birth to us, that we met any pepper that they grind at the mill, they don’t eat it, they make sure it is in done locally, all these things by the time it accumulates, it becomes a disease in the body, look at some people, milk is now part of our diet which was not so then, and it is not what some people can tolerate, from what my people said previously, there are so many things from the garden, that are more beneficial than all these things we eat now, if you eat some ewedu now when you go out , the way it will be sweet, you will know that there is more to it, which is not good for the body, so for us as men, if not that God has made us alive, it is a product of what we eat, if you eat something today and it does not suit your body, if you see it tomorrow, you are likely to stay away from it, all these junks that we eat does more harm to the body, no one can say specifically that this is what causes breast cancer if not all these junks we consume, the earth has been in existence since and I am not sure that I heard all these breast cancer, nose cancer, private parts all these things, secondly is the misbehaviour of our children, [ thank you] the way our children behave now, you see fair skinned, you taste, you see a darker one, you taste, if not that we will continue to ask God for mercy, if you sit outside at times, you will see two , three girls holding on to a boy, all these thing lead to trouble, you don’t know what is in a person’s body, you just go around with them, what they are coming to pour into your own body that you don’t know what is in it, all these things add up, it did not just start, it is man made mostly, eating rubbish ,its all man made, like they said, eating bitterleaf, worowo, water leaf , all these things are locally available and they are good for the body, that our mothers cook for us, all these things have made us strong , before you see someone born recently who is as old as we are, it will take effort, by the time they are like us, they are already worn out, how many children of these days will be 70 , 70 something that are not already looking worn out, they will have to hold their waist before standing up , and look at us, if we still want to marry more wives, we can conveniently, it is because we have been contented, it is eating junks that I think is the root cause of all these, all the things that we say causes it, if you mention it to a lot of women, they will not listen, they will say you don’t know what is happening, you are not exposed, may God almighty just have mercy on us, so those are the reasons

P2: and again you see the federal government is taking a measure, you will see that of recent, they deported some people, from overseas, plenty girls, they said they are involved in prostitution , that is what they were doing there and its part of what is causing this problem, a prostitute that just came back, sorry to say this, those from edo state were so many , when they deported them, ]like 170, when these ones come back, what kind of disease do we know that they came with from there, and they will be doing this and that, and if you make any attempt to have sex with that kind of person, you have compounded your problem, that’s what is causing problem

M: hmm, so how can we prevent all these things, [someone asked to leave the group and group members said he could leave, you can leave] how can we prevent this disease

P7: the way I think we can prevent this disease is that, it is more with our government, how is it with them, they need to announce, they should announce on the radio, if we can do this, the prevention is with the government, the reason why it is with them is announcement, don’t do this, don’t do that again , the way we have heard it now, like how many people can we tell about it, and it keeps spreading, what we think is not good, everyone has their own local food, if you get to the north now, you cant get anything tangible, you will find their own foods there, you meet fura , nunu all those things, its Yoruba that you see eating a lot of things, we are always looking for sweet things and someone who is eating sweet things and cannot take herbs, if you know how to eat sweet thigs, know that you will also take herbs, there are no more herbs like that, in those days when they make concoctions from herbs in the villages and all that and they give it to someone like this, even it is the pounded ones that smells, by the time you take it, everything flushes out, if you eat junks today, and you take it, everything comes out easily, you urinate and excrete it, now we don’t have things like that anymore, it is no longer happening, may God almighty just help us

P4: in a nutshell , I will say the banning of importation by the federal government, they should ban canned foods, it is not in line with our own culture, they should ban the importation of canned foods, they have stored it in freezers, they have stored it for a long time, you just open it and eat, the tin must have been rusted and you will not know, that was what killed one of my mates now, when I was in secondary school, the tin was already contaminated, when he got to the hostel, he just opened it and ate, he gave some other people but he had the lion share, that was how he died, so these things,

P2: sorry, number 2, you will see that how is that gari from india going to taste in the mouth, how can it be like our own, did you not listen to the news

P1: I saw it in the news

P2: that’s part of the problem, if NAFDAC did not detect plastic rice that they brought from overseas, rice with plastics, that will be melting in people’s throat, if they didn’t detect that , it would have been another problem, if they want to ban, they should ban it, you know that kind of thing,

P1: these people that do research on food, [ is it NAFDAC] nafdac is doing its own, our schools , universities too, they are doing their own, all these kind of things that we are discussing now, how is the food, what is the role of the food in causing cancer especially cervical cancer for women, if they can do, these things, it is the food that plays important role, [ p4: it will mix with the blood] in cervical cancer, they should look for ways to remove the impact of these foods, secondly as part of other health programs that they do for women, sensitization, seminars that are free, from time to time, and it is possible that some things that they use to clear some infections, when some people take it, it does not go well with their bodies, if it joins some other things in the body, or it mixes with some other drugs, it may be the reason why those places are faulty, may be th drugs that they are using, the different drugs, I will really want us to examine that part well, the drugs women use, because we heard that some people take drugs so that they don’t get pregnant , contraceptives, it is possible that this is the exact thing that is causing problem in their bodies because that thing, what fears me most about it is that, I have seen women who are above 70, when they had cancer in the womb, on the private part, I have seen a woman who at 95 had cervical cancer and she died within 2 , 3 years, it is possible she may have been infected before now, what I just suspect is that, the drugs that they use for other health issues like hypertension and others,that had the kind of side effect, and it may be that somethings are lacking in their blood , that made the have those things, I am just suspecting that it may be the effect of the different kinds of drugs that women are taking, it is important that we look into those areas too,

P4: moderator, number 4, to cut everything short, is that we should implore parents to take care of their bodies, some people are with three days pant, in the process of hustling up and down, they just change their clothes , without changing the underwear, so we should beg them, cleanliness is next to godliness, lets make sure we do that and men too, some men, you cant even look at their pants, infact it happened to me too , honestly, when we went for our pensional stuff, so they said they wanted to do weight,to know the state of our body and all that, they said, daddy stand on this weight, but you will have to remove your shoes, when I removed my shoes, the socks I was wearing was already a rag, look at that was I blind, did I not see it, I removed it there and disposed it, so that’s it , lets make sure we take care of ourselves, it can cause ill health too, so that’s the way I see it

P7: like what he said that some people don’t take care of their private parts, we need to continue to tell them, it is what is supposed to be hidden that they expose, in putting money that is supposed to be in purse , they keep it in the private part, even if they put in a nylon, did God made that part for us to be putting things there, some people will even put there phone, there phone will be there, it will even be ringing there, and even this phone, it is a problem if it is making sounds while in close contact with the body,the radiation it brings out, some people will say because they don’t want their phones stolen, they will put it in their brassiers, and this brassiers itself are not meant to be, the breasts are supposed to be exposed receiving fresh air, this is part of the problem, brassiers too have bend down select, bend down pick, people will still go and buy it, how many people are still buying new ones, are they not going to buy the second hand ones, they don’t know what happened to the person that was using it before they got it, may be the person was in the hospital when they removed it and brought it down here and we are buying it

P2: thank you sir, about what you said, I was in challenge one day and I saw people parked their cars, rich people, they poured those things on the floor, those rich people were now there, picking what they want and all that, grade 1, they continued to pick, I now told the people we were together , I said this is part of what we are discussing about, the whites that used it before, what did we know that happened to them, before they sold it to us, what kind of disease was the person affected with, some people will even buy pants, they bought skirts and some very nice gowns, nobody called them before they went there, I said they wont go to town to get things,

P3: look at a lot of women now, both old and young, look at this jeans that they wear, cant this not lead to cervical cancer, can it not cause it, it will so be tight in the thighs and even squeeze up their privates, can it not cause cervical cancer, if the heat is too much, so you see, it is more of what we cause ourselves, it is not something that just happens, we caused it ourselves, that trouser that they wear, some people’s buttocks will just spread out, and the lines will pass through her privates , and they will not because of that try to remove it

P2: thank you very much Alhaji, both Muslims and Christians forbid women using things belonging to men and vice versa, but that’s what we are used to, look at what alhaji is saying , a woman wanted to hiss herself, she was wearing jeans, if you see how we made fun of her, she was just trying to find a place, I am sure she would have done it on herself before she gets a place to use, trousers are for men, so it is all their own cause, they brought it on themselves, so lets hurry, time is not on our side

P1:as they mentioned earlier, seminars,any clothes one will wear ,what is used in making the clothes as some said is important, some people they will tell them that whatever cloth you must wear must leave enough allowance for air, especially those ones closest to the body, some people cannot stand using nylon,they are supposed to wear pure cotton but if that is what is available, that’s what they will have to make do with,

P4: so we can say poverty is part of the problem[laughter]

P1: it is ignorance

P3: it is ignorance , if you go to gbagi now,will you not buy Ankara that you can use,you will buy 1000 worth and you will make different things from it, fresh air will get to your body, it is still cause and effect,

P1: what is used in making all those clothes that we see,

M: I don’t know if we have heard about human papilloma virus,

P3:whats that

M: human papilloma virus is the cause of cervical cancer according to research, this virus is transmitted into the body during sexual intercourse, it is possible a woman had sex early in life and has been exposed to the virus from the time she is 10, nothing will happen to her until she is 40 years old before becomes that cancer, it will remain dormant in the body until she is 40 then it becomes cervical cancer, for some others, they may be exposed but their body heals up it self and they don’t get to have cervical cancer, she wont know how the virus left her and for someothers it may be that the only person they married gave them the virus, but then a lot of research were done until they found a vaccine that could protect against the virus, it is now available in Nigeria, some other countries have done it and they have been giving it to their young ones, this vaccine can be given to those who have not been sexually exposed from the age of 9 to 14

P3: virgin

M: yeah, virgin adolescents, they can have the vaccine but then if they are older and they have been tested and they don’t have ,they can also get the vaccine,so we want to know about the acceptability of the vaccine, some other countries like south Africa, have taken up the vaccination, Rwanda, it is in their routine immunization schedule, so do we think it is a good idea if this is included into our immunization schedule, if this vaccine is introduced in Nigeria, is it a good thing, that our girls too, will be getting the vaccination,

P4: moderator, please don’t lets sweat too much because time is not on our side, about the vaccine you said, it is good thing , it is a good thing, if you know anything that will prevent virus so that it wont spread, lets have it now, it is not because of we the elderly , it is because of you young people that are still coming up, that’s all

M: there is a financial implication, each dose of the vaccine is 8000 and a child will get 2 doses,

P4: what is the help government is giving to us, what help is government giving us

M; the government is not involved, it is just individuals that are taking it on their own accord

P5: you will see that when they started announcing the nets, people took it more seriously, publicity is very important, so they must tell people about it

P3: and again, instead of paying 10,000, we can pay 5000, government should subsidise the pay, you will see that in the north , that’s what we want to copy now, the girls don’t pay school fees, even at that , they sdone t eally fo to school, sot hey will subsidise it for us, it is 10 naira but we are to pay 5 naira, that’s how it should be, that’s the way they did that net

P2: people really came out to get the net

P1: ( ti o ba ni di, obirin ki je kumolu, ) it is true that researches will be done before they come up with a solution, like that one that they are doing now, but then is there any damage it is going to do to the body, In the south west , we will not decline to be vaccinated because we like to add wisdom to wisdom and we also learn from other people’s wisdom , that’s one of the things that has helped us, secondly , our people who have the knowledge of vaccination will have checked the contents to ensure that its safe, or is that not, so what I am suspecting may come up is, may be they are checking the effect it will have on people’s body. You said they have given it to some African countries, it may be that those in south Africa are safe because south Africa is cold, but hee we have hot equatorial weather, the reaction of our body, the way tour body will react to the vaccine is different, for people north of Africa, south of africa, west of Africa that’s the first thing I want to note, then again , if you call it 500 naira, in Nigeria of today, is it not 9000 or something that you call it, if you call it 500 naira, 1% of women who should be vaccinated wil take the vaccine, because they will tell you that the 500 naira can buy a congo of gari, it can buy a congo of yam flour, a congo of beans, if government wants to really help, the government can borrow money from world bank to sponsor it, or they should use foreign aid, if it cannot be free, they should not let it be more than 100,by my own gross estimate of Nigeria’s income and our reaction to things, some people even drugs for headache or fever, instead of going to the hospital to get the drugs, someone will be sick , to go to the hospital will be too difficult because they can’t stay there, will they leave their wares and go to the hospital, even if it was free, they prefer to go for herbs and most of this herbs have different proportion for adults and children, the concentration for children should be different from the one adult should take, [ moderator please lets round up] so that’s one thing I want you to look into, the money to be paid for the vaccine, once it is more than paracetamol or any other over the counter drugs that is more than 100, then you will see people draw back, they wont come out to pay for it and once they go, it must be easily available, like the way you came now, if you announce ahead of time that it is 100, more people will come out to take it by the time you come, when you get there, give them 100, go to primary health centre and take the vaccine there, it is not that you will come to uch for the vaccine and 5000, I am telling you,you wont see more than 20,000 nationwide, but if you reduce it, that means the objective is to get as much children as possible immunised so because of that , you will achieve that, because it must not be more than an amount they can use to buy minerals, so that’s my own view of the situation

M: okay

P3: when you set this up, like that kola Daisi , if you go there, it will be better, you can make an arrangement with them there, as we are sitted here, if we say bring it here, some other people may be of the opinion that you should not bring it,[p1; for me , I cant afford that] but if it is like that Kola Daisi centre, they will know how to let people know about the vaccine, and they will know the kind of test they will do , they can say okay, they will do a test , when they finish the test, they will know what next to do,

P1: when they said it is true sexual intercourse, is it from the man or the woman

M: from some things we read, it can be in the man but the woman manifests the disease

P1: then we should be the ones who get the vaccine

M: there is no one who cannot infect the other, if the man has, he can infect the woman, if the woman has, he can infect the man

P1: so why is the vaccine targeted at women alone

M: because it is women who manifest the disease, it is the woman that will be sick, moreover the vaccine available is for women, there is one for men too, that one is known as gadacil, the one they give women in Nigeria, cerverix,men cannot have it, but the Gadacil that men can have is not available in Nigeria, may be because of the cost

P1: for it to be effective , they should target men more,

P2: all these things we are discussing is left with the federal government, publicity is important, publicity is important, that’s why we have this NOA and other organizations, like when they wanted to give the nets , they announced it everywhere

P4: it is true, even my mother in the village has a net, the publicity was really good, they really publicised,

P1: I hope we didn’t chase you away

M: no , you have answered most of our questions,

P4: even if you didn’t chase them, we have other things that we want to do,

M: we are grateful for your time, thank you for sharing this information with us
