# Supplementary material for: “She must have been sleeping around”…: Contextual interpretations of cervical cancer and views regarding HPV vaccination for adolescents in selected communities in Ibadan, Nigeria
Source: PLoS One. 2018 Sep 17;13(9):e0203950. doi: 10.1371/journal.pone.0203950 (PMC6141096; doi:10.1371/journal.pone.0203950)
Supplement: S1 CaCx data — (ZIP) [file pone.0203950.s002.zip › OUT OF SCHOOL ADOLESCENTS MALE.docx]

**TYPE OF PARTICIPANTS: OUT OF SCHOOL ADOLESCENTS MALE**

**TYPE OF INTERVIEW: FOCUS GROUP DISCUSSION**

M: As I said earlier , my name is …………. and I am here with my colleagues, we are here to find out some things from you and everything you tell us is really important, it is not an examination, so feel free to express your mind , it is necessary we talk, since we agreed to participate, It is important we express our views, do we understand, so as I said earlier we want to know about cancer, the cancer we want to know about is the one that affects the entrance of the womb , that is cervical cancer, we want to know what you know and what we can do to prevent and treat it so that people will not have this cancer again , how can we help people , so I will be asking us questions, the first question I will be asking is, have we heard about cancer? We are going to use the number tags with us to answer the questions, so whoever wants to talk will say , number so and so , I have heard about it, has anyone heard about it? Have we heard about cancer before?

P5: yes , I have heard about it before

M: okay, where did you hear about it

P5: I heard about it on facebook three days ago, I saw someone with breast cancer that the cancer destroyed the whole breast, it was completely destroyed

M: okay, so you saw something on breast cancer three days ago, so it is still fresh, thank you, so who else has heard about cancer, any type at all

P2: we hear about it, [hmm] we hear about it

M: you hear about it, where do you hear about it

P2: they talk about it on radio, and we hear often

M: okay, so they say this person has cancer, that person has cancer

P4: I have heard about it, infact there is someone who lived in our former area before we left there, it was that cancer that killed the person, it was that of the nose

M: the nose, so you have seen someone who had cancer, do we have anyone with additions may be you have heard about cancer or you have seen cancer or anything apart from those who had mentioned something , p1

P1: I have seen it before,

M: where

P1: it was on Facebook, I saw something on breast cancer

M: okay on facebook, p3, have you seen or heard about cancer

P3: yes, online

P6: I have heard about it, I read it online

M: most of us have seen it more online and one person has had a first hand experience of cancer, thank you , so the cancer we really want to deal with, you know cancer is any abnormal cell growth , you know our cells multiply and if when the cell is supposed to just multiply and stop , but then it does not , it just keeps spreading to other parts, , that is what causes cancer, if it is malignant, and there is no part of the body that cannot be affected, it can be in the blood, leukaemia, it can be in the eyes, you have seen that of the nose, it can be on the breast too, there is no part of the body that cannot be affected, but then ,the cancer we really came to discuss with you is cervical cancer, this one is peculiar to women and it affects the entrance of the womb in the cervix, that is where it is found, do we have anyone who has heard about this cervical cancer before

P!: I have not heard about it

M: don t just shake your head because there is no way this audio recorder will pick your nods, so do we have anyone who has heard about it

P5: I have not heard about it

M: okay no 5, has never heard of it

P2: I have not heard about it

P1: I have not heard about it

P3: I have not heard about it

P4: I have not heard about it

M: what about you , number6

P6: no

M: so there is no one who has heard about cervical cancer, okay, so I want to explain the symptoms of cervical cancer so that you can know if you have heard about it, it is possible you have heard or seen it and you don’t know it is cervical cancer, if we see a woman that is above 40 years old, the woman must have been above 40 and the woman is bleeding from the private part and it is not menstruation, the person may also have low back pain, the person may or not loose weight , you will just hear that the person is dead, now the discharge also has a foul smell, have we seen or heard someone with such an experience?

P5: I have not seen or heard that before

P4: I have not seen or heard that before

P6: I have not seen or heard that before

M: so no one has heard about it before right, so as I explained, we have not seen or heard anything like that, but what do you think can cause that, for a woman to just be bleeding after the age of 40 when it is not menstruation, to be having this kind of experience, back pain and foully discharge and you just hear that the person is dead, what can cause it

P5: may be because the person is not eating balance diet

M: if the person is not eating balanced diet, okay, any other person, who has an addition

P6: it can be a transfer in the blood from parent to offsprings

M: okay, so you are saying it may be caused by the blood the person collected from the parents may be the reason why that is happening, thank you, who else has something to add, what do we think can cause that kind of thing, if someone is having that kind of experience

P5: may be , those who do abortion, may be in the process of aborting the baby , there was a fault

M: okay may be if someone does an abortion and it is not properly done, but the abortion is not the cause of the bleeding at the time , or is it later

P5: I mean that after they are through with the abortion may be the person who did it is a quack, they don’t know about it, it can make the person to bleed

M: okay, do we have anyone who wants to add something , p2,p3

P3: in my own view what I think can cause it is abortion, may be the drugs used, so if it has damaged the womb, it can make the person to be bleeding , so what ever is abnormal , it can become cancer

M: okay, so the drugs we are taking, so it will become

P3: if someone says, you are pregnant, use this drug, and if the person is not a medical expert, may just prescribe a drug and the person just uses it and it damages the womb and it may cause an injury that may lead to cancer

M: okay , thank you very much, but it is not that , it is immediately the person uses the drug that it has that effect because the category of people we are talking about is more than 40

P3: yes

M: okay, so do we have anyone with anything to add, number 1 and 2, what do we think can cause this kind of thing

P2: what I think can cause this kind of thing some people, they must not be in a place where there is noise[ please speak up} there was someone in my area that was going to market, where she trades and something happened so there was a sudden noise, that was how the person started bleeding, so that kind of thing too can cause it

M: shock made the person bleed, so number 1

P1: I am thinking through sexual intercourse

M: through sexual intercourse, I don’t understand

P1: through sex

M: you mean from man to woman

P1: yes

P3: what I also think can cause it, if someone had a caesarean operation and may be the doctors were not careful that by the time, they did it, they cut something they were not supposed to cut and the healing resulted into the cancer

M: p6, do you have anything to add to all we have been saying, p4, thank you all for everything you have said, everything you have said is important, now we have mentioned the things that can cause cervical cancer, now what can we use to prevent cervical cancer from all the things we have mentioned , how can we prevent it

P3: though I said abortion, what I will say s that , one should prevent premarital sex because it is this premarital sex that leads to this abortion, so one must abstain

M; abstinence , one should not have premarital sex

P6:once a person goes for medical check up and they discover that the thing is in the person’s body, I will advise that the person goes to the hospital regularly, because they may have a drug that will prevent it

M: so if the person is going to the hospital, there may be drugs to use to treat , numbr 5, number 4

P5: if the person is eating balanced diet,

M: balanced diet, balanced meal

P5: because when you eat a good food your body will be functioning well

M: number 1, 2, 4, you don’t have anything to add, thank you very much, so we have mentioned how we can prevent cervical cancer, has anyone heard about HPV , [HPV] HPV is human papilloma virus

P5: I have never heard about it

P4: I have not heard that before

P1: I have not heard that before

P2: I have not heard it before

P3: I have not come across it before

M: okay, but we have heard about virus , what is a virus

P6: they are microorganism

M: that is correct, it is a micro organism, human papilloma virus, is a micro organism, we cant see it with our eyes, this human papilloma virus, is what causes cervical cancer in the body, this human papilloma virus enters the body when a man and a woman has sex, but then when it enters the body, may be the woman had sex at 10 years old, it will be in the body, doing its own thing until the person is 40 years old before it become cancer, it can be there, it will just be there doing its own thing, some people are lucky that their body heals up and they continue with life and for some people it becomes cancer, but this human papilloma virus is what becomes cervical cancer, just the way HIV is transmitted during sexual intercourse, human papilloma virus is transmitted during sex only , it is not like HIV that can be transmitted through other means , now the question is , if we have a vaccine, or a drug that can help prevent coming down with this cervical cancer, do we think it is a good thing

P: is it something to use

M: to prevent this virus, you know that once the virus gains entry into the body, it may not go again

P6: well, there is nothing that has a positive that does not have a negative side, if we want to have something to prevent , it is good, but there are some people that they cannot leave without it , and they are not matured, they are still young

M: but once they are vaccinated, according to research, there is a vaccine now, if a child takes it at 10, before initiating sex at all, once the child takes the vaccine between 10 and 12, she will be secured for life, one dose is 7000, the two doses required amounts to 14000, do we think it is something good,

P: it is good, it is good,

M: okay so why do you think it is good

P: it will reduce the population of the virus

M: okay, okay, number 5

P: some people don’t know, so if they hear about it and they know it, they will be able to make smart decisions about it , they will take the vaccine and be prevented, so they can do whatever they like at any time

M: do you consider it a good thing that you can do whatever you want at any time

P5: it depends on the person

M: okay , number 4, do you think it is good to have this vaccine

P4: it is a good thing, but some people will be considering the money , they will say the money is too much, they don’t want something that will give them problem later, it is good in the sense that if they take it now, they can rest, their body will be okay

P3: is it a good thing or not

M; it is a good thing

P3: the reason is that when you take it, it can prevent it a bit so as not to weaken the cells, but it cannot cure it

M: it is given before someone initiate sex at all, so the person will not have it at all, it is not curative at all

P5: it is good if it does not have side effects because some people they are allergic to certain drugs, when they take it, let it not be that it will cause another thing in their body, if it does not have side effect, but if it has side effects, it is not good

M: what kind of side effect

P5: some people, it may be that they are just having headache and they take paracetamol and it will react, it means that that drug is not good for their body,

M; okay, but the side effect this drug has is, when a person takes it, just like other childhood vaccines,it may come with some swelling or the site will be red, do we think that is too much of a side effect

P: it is not too much

M: number one, is it a good thing,

P1: it is a good thing, it will prevent the virus

M: if they say this vaccine, is available, come and take it, what will be your first concern before you get vaccinated

P1: I will look at it that, what will be the effect, since it will prevent it for some time , so that is what I will think before I take the vaccine

M: I can’t hear you, even though I am so close to where you are sitting

P1: since it is what I know, that it will secure it for a while, it will secure the virus for a while, so that is what I will think before I go for the vaccination, that it will secure the virus for a while, so that is what we will consider before we go for the vaccination

M: apart from securing for a while, there is nothing else you will consider before you go for the vaccination, nothing else will give you concern about the virus,

P5: what I will consider before I go for the vaccination is firstly, the money is much and we know what Nigeria is saying now, if we take a whole 14 000 for vaccination, when it is not as if you are infected already, so it is too much

M:do we have any other addition

P3: as for me , it is not too much, if there is money, you can pay for it

P4: his mummy is collecting daily contributions

P3: because that thing kills people, that’s first, and it is transmitted sexually, one does not know the kind of wife he will marry, so it is better to prevent it with this vaccine than the one you will spend later, if you prevent it now, it will be better

M: do we have any other person , who has a concern that you want to talk about , if you are asked to come for the vaccine, what will you consider

P5: if they bring it free, people will come out for it, they will take it freely

M; you will just file out with your money there is no problem, number one, what will be your concern, if you have information that the vaccine is available where you are

P1: money has said that if it is not around, you don’t make any plans

M: money is the main thing

P3: money is the main issue but then I will be wondering why I should take it, when I don’t have the virus, I cant see any symptoms

P4: people will want to be tested, they will want to know if they have it or not before they take the vaccine, some people may also have the money but they will be considering using the money for other things

M: so apart from the money, we also think we should do the test before we get the vaccine, okay , so what do we think will be the reaction of our parents, if we say that we want to get the vaccine, will our parents agree that we should take it or not

P3: if I were a parent and my child tells me about the vaccine, they will say that there are many leaves in the bush, we will make herbs from it

M: there are many leaves

P3: to make herbs

M: what will be your own parent’s reaction, that’s your own reaction

P3: another thing, is my mum will say that you know Ajimobi has not paid and that 14 000, if I have 14000 at this moment, we will stock the house with food, so that is not a priority now, we will take it later

M: number 2, will your parent just give you the money

P2: they can’t say that, ever, what they will say is that , as I am looking at you, I trust God that you can’t have it in Jesus name, that if they use the money to buy somethings in the house, we will be eating it in bits, that they have not received salary, salaries have not been paid, that’s what they will be saying, she will even add that if there is 14000 now, she will go to Bodija, we will buy yam and other food stuffs

P6: for me, although my dad has a business he feeds us from, they have not been paid for a while, and we are four children, I can’t possibly ask for that kind of thing from him

P5; I have exams to write, and if I see any money now, that is what I am going to focus on, we can hardly feed let alone going to take injections

P6: all these things we are talking about is when there is money, when there is money, you can talk about this things, what is the benefit of the vaccine self

M; It is to protect you in the future and that vaccine , if it gets to the point that the person is fully matured and has left the care of the parents and involved in sexual intercourse , if anything happens, the person is at risk, then, so the vaccine must be given before the person will be at risk,, okay so we have mentioned that money is the major issue, the major issue we have is money, okay now, so if this vaccine is to be made routine, some of us that have younger ones who are babies or we have a sister or an aunt that has a baby, you know they have to give these children vaccination from time to time, is that not

P: Yes it is

M: if we are to make this adolescent vaccine as such, so that when people go to the hospitals, they can access the vaccine and it is available, what can the government do to ensure that all of us, as we are have access to this vaccine, what can we do, what can everybody do to ensure that we get the vaccine

P6: if the government will do anything like that, there must be a public lecture, that’s first, so that is the first thing, so that everyone will be aware about it, then they should reduce the money

M: so there should be a public lecture to let people become aware and they should reduce the money

P5: they should enlighten people on the vaccine, so that they can know about the vaccine and the outcomes, so if they enlighten people they will know what to do and expect but the money is much, why do we have government, there should be free health, but then if we have to pay, the money should not be so much , people should pay token for the vaccine, Government should subsidise the vaccine so that many people may have the opportunity to get the vaccine

M: okay , lets assume that the vaccine is subsidised and all, how do we access the vaccine, where would it be, should it be brought here or what, how do we get it,

P3: there are maternity centres everywhere

M: can we all go to maternity centres

P4: the best thing that can be done is to go to schools, and give people there, whoever wants it, because even if it is free, some people will not take the vaccine, if its free

M: why will they not want to take the vaccine, because they may still pose some risk to those who decide to take it

P: if they can tell us the harm involved, the risk, the result that is in it, some people can live with the danger ,

M: so those who don t get the vaccine, what can we do for them

P3: if they can do the tablets for the vaccine

M: do tablets,

P3: another thing is if they come to schools and they tell the students that if you don’t take the vaccine, you will not be allowed to write your exams and if you don’t take it, you won’t mark the register and three if you don’t take the vaccine, there is punishment for you and the punishment is not the kind of kneel down and raise your hands punishment, the kind of punishment will be go to the field and cut grass, and by the time you do that punishment, you will be scared and decide to take it, I can’t risk my exams,

M; So it should be attached to punishment

P6: it should not be attached, there are some people that cannot take injection, that if they take injection, they can have other issues come up, they can’t take injections and by the time they forcefully inject the person, they faint or even die, so anything, there are some people that cannot risk taking injections

P5: like if they can be given incentives, it will encourage more people to get the vaccine

M: so if it is subsidised or free, we will take it right

All: we will take it

P6: but the money is the main thing

M: so if it is free, then they will take it, so apart from that we don’t have any problem,

P5: no, is it not for our own benefit, it is for our own benefit

M: thank you very much for your time, I am really grateful, we have come to the end of this interview
