# Supplementary material for: “She must have been sleeping around”…: Contextual interpretations of cervical cancer and views regarding HPV vaccination for adolescents in selected communities in Ibadan, Nigeria
Source: PLoS One. 2018 Sep 17;13(9):e0203950. doi: 10.1371/journal.pone.0203950 (PMC6141096; doi:10.1371/journal.pone.0203950)
Supplement: S1 CaCx data — (ZIP) [file pone.0203950.s002.zip › FGD RELIGIOUS LEADERS CAN.docx]

**TYPE OF PARTICIPANTS: CHRISTIAN RELIGIOUS LEADERS**

**TYPE OF INTERVIEW: FOCUS GROUP DISCUSSION**

_P1; {prays} yes , continue_

_M: thank you very much sir for that prayer[ please make it snappy, we have a meeting to attend] as I said earlier, my name is ………….., but my school is in……, I am here with ………. and ……….., we are doing a research on cervical cancer, we want to know the knowledge of people about it, we want to know about their understanding and other things that we think may have to do with this cervical cancer like HPV, human papilloma virus and may be vaccine, these are the things we want to know, that’s what we want to know that what do people know, what is the knowledge of people about this things and then how can we prevent this cervical cancer, that what can we do to see that it is much more reduced , that is why we are here sir_

_P1: whoever wants to answer_

_M: yes, we can answer the questions, the number we have been given, we are going to use them, to indicate, its number one, its number 2, it is to protect us, for confidentiality reasons, you know some people will say that whatever they might have said may be used against them, so they won’t mention anything that can implicate them_

_P3: but then this one we are talking about does not carry much risk like that_

_M: there is no risk involved but this is standard practice for all research_

_P3: all these things we are discussing does not affect government in anyway, when you are not destroying something or doing something against the law, is that not, you are not talking against any religion or something, so automatically, if one is doing well, there is nothing wrong in mentioning names, may God help us_

_M: amen, it is just that if we agree to use our names now, because we feel this research is not risky, what about when we have a risky research, so what do we do, its best for us to go with the standard_

_P3: may the lord not let you come across that kind of work_

_M: amen , so we are starting now,I will be asking you questions, , the first question I want to ask is, have we ever heard about cervical cancer_

_P: yes_

_M: you are going to use your numbers to answer, like number 3, this is what I know_

_P3: I have heard about it_

_M: thank you sir, where did you hear about it_

_P3: I heard about it a long time ago, I heard about it on radio[ okay] radio and television, then I read it in newspapers then some of my friends who are into the medical field, we also discuss about it_

_M: so what are the things you know about this cervical cancer_

_P3: briefly, what I know about it is that,it is a type of cancer that is peculiar to women, that is what I know about it, it has never_

_been said that a man has cervical cancer, it is usually women, I know about that, though I am not a medical person but I know it is very peculiar to women,_

_M; thank you very much sir, is there anyone else who wants to share about cervical cancer, if we have ever heard about it and what we have heard about it_

_P10: praise the lord_

_All; Halleluyah_

_P10: we heard about it when we went for seminar in Oyo state seminar{ continue ma} the one that is in challenge,, that’s where people , those who are really doctors came and gave us lecture, that if a woman wakes up and wants to know if she has, she should touch her breasts and once she notices any lump there , then she will know that she has it, she should just go to the doctors straight and complain , so she can do the necessary tests, then we went to hear about the one of the cervix too, we were told to do tests ,some people were running because they said they cannot allow them to be touching their private parts, so the doctor explained that it is for our own benefits, who ever does the test is for her own benefit and if she cannot do too, it is her own, many people came out that day to do the test, those who had, they told them, those who don’t they also told them, they also checked for fibroids and gave us a talk on fibroid, that If they have it or they know someone who has it, they gave us the number of that hospital that we can call, it was the doctors who came that day too, that they should go for check up, that once a woman is 50 , 48, they should make sure they are going for regular check up, blood pressure and the general state of the body , like that_

_M: thank you very much, when you had the seminar, were you told about the things that could cause this cervical cancer_

_P10: what they said was mostly on hypertension, that one should not brood or stay alone too much, that one should ensure to be in places that will make the heart happy that one should not be putting things to heart too much , that when one is committing too many things to heart, the person will not be able to sleep well and that is the beginning of problem and then anything or any place that is too noisy , we should avoid it and_

_M: thank you ma, do we have any other person who has heard or known something about cervical cancer_

_P5: praise the lord, ha, number 5, the disease is the one that affects the womb , right, the disease is the cancer of the womb right? [ no, cancer of the cervix] cancer of the cervix, may the lord help us, we will see that as the world is becoming more developed , we are seeing new things, this one we are discussing today, we will discover that before we even know the term in English, it has been known since the days of our fathers , it is not a new thing and it is causing a lot of problem for women, this disease have been mentioned in localities before we started hearing about it on the radio, this one we are discussing about will be helpful in bringing an end to this disease and this disease comes in to the woman in different ways one of which is, if a woman is not old enough to see a man and she is seeing a man, this can cause this disease, if a woman is seeing more than one man that she is supposed to see, it can cause this thing, it is a disease that a woman can become infected with from someone else and she can infect another person too because any man that has intercourse with her, it may not be obvious but then it will manifest in another woman the man may sleep with and the difficulities it comes with have been mentioned earlier, it brings pain to the woman and it is a cause of infertility in women, this disease is not a disease we should handle with levity, traditional healers are doing their part, we the prayer people too, we are trying but then majority of women with this issues cant tell the man of God that they have this problem, it is not as if prayer is not effective but their faith is to little for prayers alone to work for them because of the pain they are passing through, they may be looking at it that will prayers resolve this issue because they don’t believe, may the lord help this nation , that’s what I see to it, what you are doing is a good thing, keep up the good work, it will bring understanding to many people and it will also caution people about using their bodies the way they should not, having sex with too many men, it will be well with us and may the lord make our nation greater_

_M: thank you very much sir, number 4, you have something to say_

_P4: number4, the time God spoke with me on this issue we are discussing, it was not just one thing that God spoke to me about, he spoke to me about different kinds of disease, that is in the world that we are being infected with and God made me understand that time that what he has planned and put in place for us is to stay away from sin, stay away from sin because most of these things we are talking about is caused by sin because if we take not, if one is taking drugs the way she should use the drug as a woman, the way she meets with the opposite sex, God told me these things and I wrote it in a book to be used for teaching , especially to gather women together and teach them, as the world is evolving, sin is becoming more rampant but if one can keep himself/herself and the lord can attest to it, he has told us that he will not allow any of the diseases that afflict the Egyptians to have a hold on us, does God lie, God does not lie but people become entangled in this things because of the decisions they make which God is against, because the way they abort, they are using the things they take to destroy all the things God made, little by little{ eyi o to fio, eyi o to fio, woni fila alabikan oku peli} little by little, if you exert so much on your body, it will wear off with time, the body will know that something is happening to it, so and all this things, but if people will repent and come into God’s presence, God said he can cure them without medical treatment and if they decide to go for medical, he can still be with them to help the medical professionals to take care of them, you know the doctors says , we are taking care, but our God cures, with God’s help to cure whatever it is they present with, most of the young people who are affected with this thing, some of them have aborted more than four, five times, am I lieing,this is one of the causes, being too outgoing, over use of things, over use of drugs, if you take too much of drugs, it will bring other complications later, God did not create troubles for us but we are the ones who have decided to make things worse for ourselves, so all these things, God spoke to me about it , he spoke to me and we used all these things in teaching the women at the time, that they should stay away from sin because what it causes is not Good, starting is easy , but once it becomes an habit it is hard to stop, So, May God help us_

_M: thank you very much Daddy, do we have anyone with an addition_

_P8: what I can say when I went for a training on it, as daddy said, over use of drugs, drugs that are recommended for people that they are using, all these things can cause cancer of the cervix and different things like that that Dr Ogundeji taught us, we were receiving lectures about this things from him, that whatever drug that has not been prescribed for you at the hospital, don’t use it, because some people can see a drug in their house and they don’t know how the drug came to be and they just use the drug, that can aggravate the issue that they have, there are things that they tell people that they should not do and they do, some people they can prescribe a certain drug for them and they will be using another thing, what I can say about that is not more than that_

_M: thank you very much sir, we have contributed one thing or the other about the disease we are talking about, before we continue, I will like to explain the manifestations of this disease, I mean this cervical cancer , I want to tell us the symptoms so as to know if we have seen someone with this kind of experience, now, the cancer of the cervix, women that are more than 40 years of age ,it is more common among them and some of the symptoms are , if a woman is seeing blood, when she is not having her period, she is just seeing blood after sex or anytime, and the blood has a foul smell, the person may or may not loose weight and the person may be having back pain and before you know it , you hear that the person is dead, have we seen anyone with that kind of experience , may be you have heard someone it happened to or someone mentioned it to you, its all part of experience{ laughter in the backgroung} ma what did you hear?[ it is the same thing you are saying, exactly what you are saying, you see for this cervical cancer, there are many things causing cancer, that people talk about_

_P10: please I want to ask a question, please the family planning they are doing,is it not causing this cancer we mentioned_

_M: is that what you think causes it, if that is what you think is the cause_

_P10: I have never done it, me that I had to plead with God before I had the children I had but this family planning because they use to say it and we watch it on TV, they say there are some you insert, there are some that are injectable and after a while the people that take it are unable to conceive and it is the cause, all this things that we have never heard about , they are now happening, they will ask them to come for family planning ,it is just as though they are the ones bringing more disease home, this is what I think is causing it, you medical people should shed more light_

_[ session becomes rowdy ,participants were dropping comments on the need to shed more light about the causes]_

_P7: that family planning, whoever does not want to have children for the main time, if she does not want to children in quick sucession, there is one for the private part, there are different kinds , I went for the seminar at kakanfo, they called Christian and muslims, but the muslims were against it, they said they can have as many children as they desire that they are not interested in family planning, they allowed the Christians too to talk, they displayed all the types that can be used and when one is ready to have more children, you can stop the use,_

_P3: but that thing you said, with what people are doing , they want to take things to perfection, if the government has set up something, and we have to go to clinic to access the service but for some people , when they want to do their own , they will go to any length to do whatever they want to do, I hope you are listening to me, people who are not professionals to get involved because of money , there are different types of doctors and nurses now, such that some people do the work they are not qualified for, that someone had an injury to the leg, and the leg became gangrenous before they realised that the person is not a qualified doctor that the person was just working in the hospital, it was God that saved that woman with a lot of prayers, so some of the reason why we see these things is because some people who are working are not qualified for the work they are doing, they are just doing it because of the money, lets look at the things we are buying in the market, look at the drugs we are using there are some that they are not the perfect one and we won’t know, so all these thing are used in harming people and we won’t know about it, that’s how we know that some of the things the government is setting up, people cannot do it the way the government wants it done and until that is done before everything will become okay_

_P10: please don’t be upset about our questions and responses_

_M: they are your opinions ma, moreover , I want us to express our hearts the way it is exactly, so you are not wrong, I have told us about the symptoms, and from research , what has been shown to cause cervical cancer is sexual intercourse between male and female and this intercourse , it is not immediately that it causes the cervical cancer, may be one has been exposed to sex from the age of 10, the person will have been infected with the virus, but the virus does not become cancer until the person is 40 years old or even more than that, that is when it comes out as cancer, for some people, God helps them and the body heals itself while for some others, the virus remains in the body till they are 40 before it becomes cancer and will be manifesting in their body, as daddy said, it is transmitted through sex, and the virus transmitted during sexual intercourse is HPV which they call Human papilloma virus and it is the virus that leads to cervical cancer and they know that for now, the only way it is transmitted through sex, it is not like HIV that is transmitted through other path ways, that one is mainly through sex, now I want to ask , we know the disease,, we know the virus, so if we have something like a vaccination, that is able to take this things away, what do we see to it, do we think it is something good, what is our view about it, what do we see to it as religious leaders? What is our view to having a vaccination that will take this thing away, do we think it is something good or not,_

_P5: praise God, number 5, when you were saying now, you said as religious leaders particularly Christians that we should say if it is good or not, the introduction of this vaccine or is not good, yes, from the government line of thought and the your organization’s it is a good thing but then if we look at it, it is like encouraging people not to stop what is causing this disease because if you have sinned , the bible says you should die, when we tell people that don’t expose your children , don’t allow your children to be exposed early this has not even been effective then we go on and tell them that there is a prevention, it will encourage the children to say well, they have taken this thing let them go ahead, so if we have a cure for those who already have, that is what the problem is and it is better if the introduction of that continues, but if we have to tell the younger ones that this vaccine is available so that so and so does not happen to you and once they have it, they will think it is an express, they can do whatever they want, like the mother who has not trained her child in the way of the lord, who is telling her child in secondary school to go for injections to prevent her from getting pregnant, what do you think will happen, her mind will be at rest[p10: she is teaching her} it is like equipping her, may the lord help us, it is good as the whites have introduced this to us and it is good as your own organization came to tell us that the vaccine is available, for the question you asked it is good, apart from those of us who preach the truth,, the bible said train up a child the way he should go and when he is grown , he will not depart from it, we cannot give her prevention when we don’t want her to do what is against the will of God, if we can accept it that way, it will reduce the rate of all this misbehaviours that we should be teaching our children that those who are exposed to this early they have this problem in future, if it is so, we should pay more attention to teaching our children in the various churches, and extend it to schools, you will see that in schools, education exposes this children to what we don’t want and some teachers when they want to teach children, they will place more emphasis than what is written in the textbook to the extent that there will be so much noise in the classroom, students will be so excited the moment you mention that topic, so it is better if they minimise the way they teach this children, it will expose them, it is the danger that we are supposed to expose them too, there is danger here, may God help us_

_P4: the vaccination is good but the vaccination, do we think it affects the children, there is a minister of God in this area, the last child they had before this new one was almost out of secondary school and It is the same family planning they did, how do we say that one’s mother is pregnant, it is a shameful pregnancy and at the end of the day, she had the baby, so as much as it is good ,it has side effects, May God just help us, when I was still having children, I didn’t do family planning but I have safe time that I use, this is the time I want to have a child and there are some people that either they do it or not, they still have issues with conception,may be that one, if there is a mistake how do we know_

_M: hmm, this is a vaccine, it is not a family planning method, it is a vaccine to prevent cervical cancer_

_P4: don’t you know about the family planning too, because someone asked and I said, don’t you know about that line too,_

_M: for that , it is not the reason we are here but I can still answer your question after the program but lets finish this first because of our time_

_P3: emm, as knowledge increases, those who are doing research are doing it all over, it is not our nation alone, so if they do the vaccine, the church of God, in the church of God , there are knowledgeable people there, in our localities exactly, we think people don’t have knowledge, but many young people have knowledge , so they won’t be against it if they do it, if they can discover the vaccine to prevent this cancer , cervical cancer, what I know is that , Christians , Muslims and traditional worshippers they cannot be against the use of vaccines especially in southern Nigeria , we are much more exposed than other parts of Nigeria, people know very well about it so they can’t go against it, so it is a good thing if God can help those who are in charge of the research to discover the vaccine_

_M: thank you very much sir, apart from what he said that giving this vaccine to adolescents may want to make them see it as a license to do whatever they want, do we have other thing that may be a kind of concern to us about this vaccine, may be things we should look into , like this thing if you do it, it is good but this and this are the concerns, that this is what I am looking at, this is what I am looking at, if we can fix this thing then everything will be okay_

_P4: may the lord help us, vaccine or no vaccine , that is not the key, it cannot help anybody, the bible said we should be holy as our father is holy, the best is prevention, the moment that start engaging in sex, they will be deaf to everything you say, they are easy to deceive , this person gave her 5000 today, another person gives her 15000 tomorrow, thank God I have a vaccines, she will not know the day the vaccine will wear out , she is building a castle of problem into her own life she will now say that some people are against her in her old age, she forgot what she did, so the basic is that whatever has to do with sin , we should avoid, the key to sin and everything that has to do with sin, we must forsake them, don’t be bothered that the friend you work together is already married five years before now, everyone has their own time, may be the husband you will marry, you have not even met, maybe he is even in Lagos and you are here in Ibadan, may be it is even in 6 months before you think of going to Lagos, may God deliver us from sin that makes one fall and makes life miserable for someone, there are so many thing we hear on radio, so many things , some men beat their wives up because they lost a pregnancy, even going to the length of pouring fuel on her and trying to burn her up, you know that kind of thing will not be a good thing for the parent, may God help us that we will not walk on the edge of sorrow , because so many people are working on that edge_

_M: thank you sir, I want to ask that we as Christians leaders, can we allow the children worshipping in our churches to take this vaccine, knowing that this vaccine is available now, and its 7000 and they will take 2 doses making 14000, can we allow them to have this vaccine and then the person gets the vaccine between 10 and 12 years old_

_P: which vaccine_

_M: the cervical cancer vaccine_

_P: those who have not had sex too can get the vaccine_

_M: it is majorly targeted at them_

_P5: praise God, it is number 5, it is as if you are missing something , you just told us the positive sides of it, you didn’t tell us the negative side of it, you are only telling us the good part you didn’t mention the part that may be against what God has created, when God made us , he didn’t make the vaccine with us, but what is that thing that can manifest when you take this vaccine, then secondly where I minister, you know I already said it, the vaccine is a help for those who want to be promiscuous, it is contradictory to what we preach if we use the mouth to preach the truth and we say , well we want to neutralize the truth we have been saying, you can have this vaccine so that you don’t come down with this disease, what about the truth we have been preaching that is my own point of view, if you can answer the first question I asked , it will really be of benefit to us all, but then, it will be against what we preach, because the bible tells us in Leviticus that any child that has been deflowered must be married to whoever deflowered her and it is still happening among the whites that whoever has been deflowered cannot be married again and it is still in practice till date, and we are now encouraging it , it is against what we preach, may God help us_

_M: what I know about the negative effect of the vaccine is that, once it is given, the site may be a bit swollen and or reddish, and there may be pains for one or two days, [ phones ring] apart from that , it is similar to children getting vaccinated, babies , it is just like that, there is nothing much of a side effect_

_P: you have not answered the question, you know he asked that what is the effect, the side effect, some women will do it and they will not be able to conceive again, maybe it can affect the menstrual cycle that it will no longer be regular, that’s the kind of thing people are asking_

_P: she said it has no side effect_

_M: that’s the side effect, like if a child was immunized and the site is swollen, I don’t know of any other side effect, You know when people have their children and they take them for vaccination_

_P: do you know of anyone that has been vaccinated when they were young and they are now grown to confirm that there is no effect_

_M: is it for this cervical cancer_

_All: yes_

_M: it was not long they discovered but we don’t have adults like that per say who have been vaccinated from adolescence, most of them are still young but so far so good , there is nothing_

_P: you can say there is nothing, but that is not assured_

_M: mummy, do you have anything to say to this_

_P: theoretically there is nothing, but practically, that is what our father is asking you that has there been anyone, because among the whites, before they produce drugs for mass consumption, they will have been working on it for more than 20 years before they bring it out, they will even have people on whom they have tried the drugs and they will be sure that it is safe, that is how they do it,_

_P: have they done this one like that too_

_M: they have done it, they cannot just produce it for mass consumption without it not being tested or proved to be okay and they have done it_

_P: you see this question we just asked you , you will throw it back to the lecturers, they have gone far and wide , they will know more and be able to tell us more about this things, so that is it, May God help us, we will all succeed_

_P: I think we are through_

_M; is it about what we are discussing, because of time may be when we finish, mummy do you have anything you want to add to what we are discussing_

_p6: there is nothing, they have already said everyting on my mind, the main thing I wanted to know is what effect it will have in the future_

_M; it is the same thing I have said, there is really nothing new, what research has shhown is what we have said , the side effect is just the immediate swelling and redness and it is not something that is so serious , it is not an adverse effect_

_p4: may the lord help us, my own addition is that, what I have to conclude with is that, instead of coming to the churches, it is better for the schools they are the ones that can use it, so during the PTA meeting ask the principals, they will tell you the time to come and introduce it but coming to the church is against what we preach and it is now left for the parents, we are doing our best as ministers of the gospel_

_M: do we all agree to what he said_

_P4: I said from my own side, I am not generalising_

_M: okay, I just asked so that if we have contrary views_

_p3: It is the same thing we are saying, that we should go to schools, and tell them those things, it is not for the church_

_P5: start from the primary schools, because those primary 5 children, what they are doing is more than what the mouth , what some other secondary school children cannot attempt , it is a serious issue, then the markets too, supermarkets all those places_

_p4: those are the places you should go to, there is no time, a minister of God will be talking and he will be warning seriously against sin, is it in dating or their relationship wth the opposite sex, they must be very careful because of the future effects and we will be using it as an example to them that some people have done it before that by the time they will be married they had aborted all the babies they are supposed to have, when we are praying for some people, God will be saying that she has aborted three pregnancies, four pregnancies, God will be saying to us that he is not the one that made her barren and when we ask them ,, they will confirm to us that it is through , and what is causing all this, going with boys when it was not time, whatever you will eat for a long while , you don’t rush to eat it and this is what is supposed to be preached to them from the church, but then if they listen or not, the minster of God will have a clear conscience before God because it is not everyone who will listen to what is being said in church, it was not everyone who followed moses that listened, there is no way a minister can try that there wont be people who wont listen, satan has his own people in the church and no church is like the other, that is the problem, it is individuals in the church that must be careful, the bible is my friend or I have some people in the church that are my friends and they are not God’s people if Judas had friends that they sat together to do that work he wanted them to do, it is possible to have an apostle that will join him to do the work, that satan will use too , so they will be two and they will share the money between themselves, so that is the way it is in the church, may the lord help you_

_all: amen_

_p4: you will not be put to shame_

_all: amen_

_P10: they are saying that females should not be circumcised, those who are not circumcised they are the ones who are running after boys now, they have become so promiscuous_

_P6: they say that thing is dangerous really_

_P10: it is dangerous, you see them in the corners, you know we are the local people, all these ones are alakowe[elites}you see we that are in the locals, we see a lot of things, in every corner, you see them, most of them I don’t know them, I just pour them water and use stick to chase some others, some of them, I follow them to their mother’s house, so May God help us, may be because they are not circumcised that theyhave become dogs, they have become so promiscuous, they go to look for men by themselves, it is part of it,, may God have mery on us, we are in the end, Only God should take the glory_

_M: thank you very much, we are grateful for your time spent, we pray that God will help us make this country better_

_all; amen_

_M: thank you very much , we have come to the end of this interview_
