# Supplementary material for: “She must have been sleeping around”…: Contextual interpretations of cervical cancer and views regarding HPV vaccination for adolescents in selected communities in Ibadan, Nigeria
Source: PLoS One. 2018 Sep 17;13(9):e0203950. doi: 10.1371/journal.pone.0203950 (PMC6141096; doi:10.1371/journal.pone.0203950)
Supplement: S1 CaCx data — (ZIP) [file pone.0203950.s002.zip › FGD RELIGIOUS LEADERS ISLAM.docx]

**TYPE OF PARTICIPANTS: ISLAMIC RELIGIOUS LEADERS**

**TYPE OF INTERVIEW: FOCUS GROUP DISCUSSION**

M: thank you for the permission to record the conversation as I said earlier, my name is ……..

P1: ……… what? Full name

M: My name is ………… and my partners here are …………. and ……….., we are all together and doing this interview together, so we have come to you , religious heads , so that we can know, what are the causes of cervical cancer, this cervical cancer is what affects the entrance of the womb, so the first question I want to ask is, have we heard about cancer before now, have we heard about cancer before, it is the number we have been given that we are going to use to talk, daddy you as number one, you will say, this is number one

P1: we have heard about it, cancer is not a strange thing, it is not strange because, what usually cause it, what causes it, it is more about the things we are eating, what we eat, because in the past, what we eat is different from what we eat now, it is totally different, you understand, for example, if we eat, they will tell us to drink water, is that not, [yes sir] but now, water is no longer compulsory, it is only if you are interested, different things like that, that is one, the second thing is the fruit we are eating[fruits] yes, fruits, most of the fruits we eat now is unripe, unripe fruit is what a lot of people eat, for example , you will see that as civilization continued as people eat orange now, they will be making comments, this thing is unripe, this orange is unripe, that is one example and they will continue to eat it, and they know what it causes , that is one of the causes of cancer,so this is one of the causes of cancer

M: sir,the fruits we eat and some other foods we eat, refusing to drink water after meals, you said it is no longer compulsory to drink water,

P1: yes, when they finish eating, they will not bother to drink water again, that is no longer there again and this things have their own effect

M: is there any other person who wants to contribute, what is your number sir?

P3: it is as if this discussion is different from what I anticipated

M: please sit down sir

P3: thank you, as an example, since the day of birth, since the day man is born, I think that’s when cancer starts and all other diseases that one may have because those who are bathing the baby , if they don’t allow the germs in the baby’s body to be washed away, there will be problems later, if they don’t handle the umbilical cord well, if they just leave it, all those germs will continue to grow there, it is the way we humans sleep in the night, that’s how they sleep in the afternoon, such that when we eat in the evening and are about to lie down, that is when they will now be looking for food everywhere, starting from fluid or blood, they will start feasting on the person, when the person is no longer able to resist it, that’s when you see that the person is not in good health, it is not only cancers, other diseases that come from inside, that’s how it all starts, the reason I can say this in public is that, you are in the hospital, all those who take deliveries, they should be trained, in that before they cut the umbilical cord of a child, they should make sure that everything in it, all the diseases we used to have in our body, because most of the disease we battle with, It is not coming from outside, it is all in the inside, and it will be there, like the placenta they used to cut out in a baby, all those diseases are supposed to come out with it and they will be removed , but because people are not thinking about it, then you have it that , all the other dangerous diseases will now be left, that’s how it will continue to surface one after the other, that’s what I have to say

M: thank you very much sir, is there any other person who will like to add to what we have been discussing about cancer?

P: what I want to add is that, you see that cancer is of different types, some affects the throat, some the lungs, some the liver, and it can come from two ways, if it does not come from the father, it will come from the mother, are you listening, there are many men, who infect their wives with diseases and the wife infects the child, the same way the wife infects the husband, and this cancer we are talking about, it depends on the way you have sex with your partner, that’s what will determine how the cancer will come in, because some people will have sex with their partners in a hurry and may be thinking it will not end in pregnancy and it does ,then whoever has cancer is going to infect the child with it, if it is in the father, he will infect the mother and the child[hmm]and it is this thing that become something big, you will see they say that when a woman has cancer, that thing is likely to kill the woman, in the hospital , they will be running around that this cancer, and when you see it, it will be on different spots in their kidneys and lungs and there is no one who does not have it, our fathers then used to protect themselves with herbs and concussions such that that thing will not even affect them till they die, so that is it

M: thank you very much sir, as our father said, he told us there are different types of cancers and the reason we came here is to discuss the particular one that affects women[ interruption by one of the participants, two of the participants: be patient, you will have the opportunity to talk, okay talk] please talk number 8

P8: about cancers, I have seen the one that affects the breasts of women,[ breast cancer] that cancer, what is it meaning in Yoruba , this cancer thing is now so popular now, and it is transferred sexually, that germ , the germ among the elders , the elders of the past use herbs to wash it away, once someone is ill and they take they person to the village and it is cured, most of the cancers we see are caused by sexual intercourse

M: is that also true for breast cancer

P8: most people get infected, while for some people it is in their blood and the elders too say that when one spreads a cloth, there is an insect that can land on the cloth [ is it tanmona] it is not tanmona, it is another thing, [I remember, that thing happened in Abeokuta], secondly, someone can be born with it, thirdly they can afflict someone with it, how did I know, my wife had it, we went to the hospital, had several injections and treatments to no avail, then some people came for wedding celebration and they told me, take this woman to the village, so I decided and we went to the village as we got there, we saw my wife’s aunt, as soon as she saw her , my wife could hardly work , they exchanged greetings and all that by the time she removed the covering she had and she saw what is happening, she exclaimed, she begged me that I should let my wife stay but my wife said she cannot stay, do everything you want to do, and if it works in a week , I will be back, so I asked the person taking care of her, what are the things that can cause this thing, he said firstly, that it can be in the blood and that someone can be afflicted with it, he said the one they afflict people with is tougher than the rest, as you treat, you buy drugs , you do all the things you want to do, it will not go easily

M: Thank you very much sir, the breast cancer you mentioned is a kind of cancer, but the kind of cancer we want discuss is cervical cancer, the one that affects the entrance of the womb, that one affects the womb, you know where the baby comes out from that is known as the cervix, do we have anyone with any experience of cervical cancer, this cancer that affects the entrance of the womb, do we have anyone who has had the experience

P: so it’s the one that affect the entrance of the womb that we are talking about

P: you see about that one, it is very common but only those who are really affected can talk about it, those that are affected, they are the ones who can talk about it, for example, it is someone who has a wife that is affected that will understand that this is what they are talking about, but the real experience is rare except you are affected

M: maybe I should explain the symptoms, we may have seen it and not know that it is what we have seen [okay], if we see a woman that is 40 and above, that is bleeding, but not her menstrual period with a foul smelling, it may come with back pain, and she may or may not be loosing weight, you will just hear that the person is dead, so that is how it presents, but the causative agents would have been in the body before the time. Have we seen anything like this, that person bled to death, we did all we could

P: about that, it is whoever, it hits that can really know, we don’t know anything

[ other respondents answered, we have not seen anything like that]

P: that blood that you said, there are some women that bleed for long, if it comes everyday right

M: it comes every day and it smells

P: I am not sure, I have seen anything like that

P: so that we don’t waste our time

P: I have not seen anything like that, we have not seen anything like that, lets move on to other things

P: it is what we know that we can talk better

M: so what do we think can cause all this things that we mentioned, that can make a woman have this experiences, this cervical cancer we mentioned, what do we think can make a woman have this kind of experiences

P: I don’t know, I really don’t know

P: they came to ask for your experiences, it is your experiences they have come to know, if you have an idea that’s when you say, this is what I have to say

M: do we have anyone who has something to say, or we don’t know what can cause it

P: it is only the white man’s doctors that can know what caused it, it is when it gets out of hand that they will now take it to the herbalist

P: when you are not affected, you won’t know the actual thing that causes the thing

M: , there is a way things run in the neighbourhood,I expect us to have experiences, based on what we have seen, it may even be in our neighbourhood

P: Lessons or experiences

M: that you can give us

P: it is those affected that can really talk about it

M: please let baba share his experience

P: the one I saw, I don’t know what caused it, they took the person around for a long time,

M: so what did they say caused the thing

P: everyday, she was bleeding, if she sits and stands up like this, its blood, and she will be smelling [ that’s right], we went to the hospital, there was no solution still

P: like he said, this kind of experience, people will say, that’s how it happens, that’s how it happens, she is bleeding, then they will offer different kinds of medication to that person, they do medication it is called,” ogun awoda” there are different kinds of this awoda

M: what was the cause of the ailment this person had, was it diagnosed, or what do you think caused this

P: it is called awoda

P: It is another kind of disease that affects women, instead of the period to come in three or five days, the periods comes and is non-stop for more than 15days, then it is awoda, they will give the medication to the person, and it is mostly seen in women, it affects women, it is this awoda that refuses to heal that becomes cancer[ that’s right] and the reason why that is happening is that ,The places where the baby comes out becomes open and once this happens, it will start coming out and that’s the reason for the complication, there is nothing that once it refuses to respond to medication and It has to do with bleeding and its refusing medication or treatment, it will eventually lead to death, so all those who are experiencing this awoda, out of all the tiras we prayed with, they all mentioned it, but then, we have not been able to find the actual cause, it is not written but for sure, it is one of the diseases affecting women and cancer is also one of awoda, once it is more than 15 days, it has become a disease, so the next thing is to look for solution to it, and if it does not end, then the person may die, we have not heard that someone was affected and the person survived, it will eventually kill the person, it is a disease and we don’t know what causes it in particular, you see most of us here, grew up in the villages, most of the diseases that are just surfacing now in our adult years started when we were much younger, there is nothing we are conscious about eating when we were still in the village and even the water we drink, the kind of water that when guinea worm was still very rampant, we drink from the rivers them such that when we get home and we are asked to get water, and we take our water pots, too fetch water, we can walk from here to the upstairs there and when we get there, we will see the oil produced by the guinea worm on the surface of the water [ other participants: that’s right, that’s right]and we just use the bailer to pass it aside, and get the water we came to fetch and we still drink out of it, [ that can lead to having guinea worms] no we may not even have the guinea worms infection but it would have been in the body and when one becomes older, all this things will start manifesting, like roasting yams and not removing the back of the yam, and we eat it[ we eat it] together with the back, nothing happens to us then, [ yes then] and then there were no hospitals like that, we use leaves, we just take leaves and mix it together, irawe and all that, some of this bad residues come out when menstruates and for men, in the urine and other fluid, but is it everything that will come out , I think it is all these things that we are eating that became diseases but the most conspicuous of the things you asked, it is caused by awoda, because what we read in the tira is that once the menstruation of a woman passes 15 days, it is a disease, some diseases heal , some don’t and the one that does not heal is the same one that causes the cervical cancer

M: sir, this person you said, is the person more than 40 years of age

P: the person will be close to 40 when that ill health started because she was like a mother to us and that was a long time ago

M: let me hasten up

P: please hasten up, we are late

M: The organism responsible for this cervical cancer is HPV, human papilloma virus, and those who researched into it said, this organism is transmitted sexually from a man to a woman, and through further research they discovered a vaccine that may be used to prevent, and if there is a vaccine that people can take to prevent them from having this infection, and we know that the HPV , once it is in the body, it comes in to the body through sexual intercourse, and if it gains entry into the body, for some, they will be okay and it fizzles out and for some others it will be in the body until the woman is older than 40 and it becomes obvious, do you think it is something good if we have this vaccine and people can get it, what is our thought on this vaccine?

P: the vaccine,is it the orthodox vaccine or the traditional vaccine,

M: it is the orthodox vaccine, the type they give to young children in the hospital, do we think it is something good, is it good

P: it is good, it is good

P: is not for someone to be protected

M: no 8

P: prevention is better than cure

P: if we can solve it now, then our problem is much reduced

M: yes, our father , what’s your number, you were not given any number, number,[ number 11]

P: about that vaccine, you mentioned, is it for men or women or both

M: it is for both of them in event they are exposed to the infection, they will not come down with it, baba, you were saying something

P: I said its good

M: you also said prevention is better than cure, may be you have something to add to that

P5: it is the same direction you are going that I am going too, you see all these things that we are talking about, you are only looking for a way to get information from us, you already know the answer, you see what we are discussing, the things people said is the truth, other things that may come in are just additions, cancer does not come in a way, there are some many ways, this my teeth is cancer for instance, it is cancer, I am the one who knows that is cancerous, and the money I have spent taking care of this teeth, I am the only one who knows, I started spending from Dugbe to the UCH dental clinic and the money is substantial to do something reasonable, till date no improvement, you know if it was something that kills easily, may be I would have died,its part of cancer, passing urine non stop, it is cancer, anything that can kill, why I said prevention is better than cure is that, the medication we should take to prepare the body , that we will have the immunity already, it is different from things now happening, we will now be looking for the drug, the one we have in the body, if anything is going to happen to the body, it will push it out and that’s why we say that if there is a drug one can use, it will be very good, like that vaccine

P: as you said

M: thank you, daddy do you have anything to add

P: nothing, it is as they said

M: now, as leaders of religious bodies, what will be our attitude, can we allow those who are under us, like I was saying, can we allow those who are under us to get the vaccine, the vaccine is targeted at those who have not been exposed to sex, between the ages of 10 and 12, and it is 7000 and what will be the challenges/ disadvantages/concerns that may not allow them to have it

P5: that one you are saying, where you started from, that’s what is causing problem today, if you have seven thousand, what you will buy with it now for housekeeping, and that is what is really causing problems now, even to buy paracetamol of an ill person now, it is not easy in some places, this is one of the reasons for this things, I was with some people today, and we were joking about our president, that he travelled abroad, what has he gone to do, for what, don’t we have qualified doctors here that he has to go abroad, the kind of care they go to access outside Nigeria, they didn’t make arrangement for such things here, and that’s what is making ill health more widespread , instead of the government to help us, if you get to the hospitals now, if you will test me, you will write your own, if he is testing me, he will write his own, for a poor man, and you will say you don’t want that person to die, we took a sick person to the hospital after all the money spent, the person died and we wanted to take the corpse, they said no way, that they will do post mortem, after he died, [ after he died] that’s what is really killing us, anything that involves money, that is what is really killing us now, the major matter is the money

P1: I went to the hospital today, and the drugs prescribed earlier is finished, so I took the empty containers for the drugs, when I got to the chemist, the one they have is slightly different from the one I was given, they said it is the manufacturers that are different, that, it is the same thing they do, and now, I asked how much is it, they said 7000, 7000? Where do you want me to get that kind of money? I am a pensioner and we have not been paid for five months, like that, like that

P3: when we started work in UCH, then if you retired from UCH when It newly started, if you see a pensioner that retired or his wife, or the four children the government agrees to sponsor you are taken back for any ill health, you will be treated freely but that is no longer the case now, if you r5etired yesterday and you went back today for any treatment, the money the person will pay, it is the outsider’s rate , even those who work there, so these things are general

P11: it is the government that is killing us, all the things that we are being starved of, we have them, we have enough for everyone and the things government ought to do, they know it but they don’t do it and if anyone is sick among them, they will take the person abroad, if they have money

P4: whoever has money will go abroad , when I went to mecca, I went to mecca in 2010, when my mother became old, she had issues with her eyes, we took her to the hospital, they said she is supposed to have started taking some drugs at a certain age because she is likely to have eye problems later on, that there is a fluid in the eye that reads 75, and it should read 25, when they did medical test that there will be surgery to leak out the fluid out the eyes,time we presented my mother is too late because of her age, they said the she gave the example of the leaf on the water, and the government knows these, things, but they don’t do anything about it, with that information in my hand , I kept it, when I now went to mecca, you know I have information now, after we finished the pilgrimage activities, I went to their general hospital, they checked my credentials, when I got there I was directed to room 87, when I got there, the doctors I met there are like 3, one was elderly, another middle age and the last one was young, from that room , I was directed to another room, for you to know how our government is killing us, one of the doctors from the other room was already there, all the equipment they use here in UCH were there, already set, you don’t know the number of times you would have visited before you get to see a doctor in UCH,they will keep rescheduling for you, I didn’t spend 15 minutes in that Mecca Hospital, they did everything they needed to do, almost instantaneously, you cant compare it to Nigeria

P: please give us prayers to start, you see there is no one here, we are all imams in our mosques, that we will now tell people that, please about the test for cancer so that they wont be affected, you children and their parents, it is 7000, they wont answer you, they will be like this our imam has become something else, as they have mentioned, it is the government that is killing us, is it me that I will be asked to bring 7000, I don’t have, I will now say that I have 7000, please tell the government, as you are now, you are part of the government too, you will succeed, you will grow old, you will have honor and God will not kill you while still young, whatever help you can do for us, that’s how it is, May God show us mercy

P1: time is far spent, please lets begin the meeting, time is far spent, time is far spent,

P6; for every young child , anything that has to do with vaccination and immunization, it is free, till the child is 10, the way it is now, firstly, there is no money in town, secondly feeding is difficult, please help us talk to them to do something about the price

P1: (session becomes rowdy) who will they give the message to, please let the last speaker be the last person to talk, that’s it

M: Thank you very much sir, we thank you for the opportunity to discuss with you

P3: please pray for them

P1: we have already prayed for them, we said God will lift them higher, they will have their own organizations
