# Supplementary material for: “She must have been sleeping around”…: Contextual interpretations of cervical cancer and views regarding HPV vaccination for adolescents in selected communities in Ibadan, Nigeria
Source: PLoS One. 2018 Sep 17;13(9):e0203950. doi: 10.1371/journal.pone.0203950 (PMC6141096; doi:10.1371/journal.pone.0203950)
Supplement: S1 CaCx data — (ZIP) [file pone.0203950.s002.zip › FGD_COMMUNITY LEADERS.docx]

**Interview group: Community leaders**

M: as I have mentioned earlier my name is ………… and my colleague is ……………… and ……….., we are here to ask you questions on what you know about cancer, its prevention and we assure you that everything you tell us will be kept confidential and will only be used for this research. do I have your permission to go ahead with my questions? [yes] and I am permitted to record this discussion? [yes] thank you. Has anyone of us heard about cervical cancer?

1: we have heard it before [where?] in the hospital. They mentioned it in the hospital

M: any other person?

2: we have heard it before, for me I heard about it on the radio

3: I have seen this thing they call cancer on different people in the hospital, for a woman it may be in the breast or stomach

4: as you have asked, I have heard it and I have also seen it before, I know a woman that had cancer of the breast. They had to cut the breast. So we know it is a terrible disease, God should not let it happen to us

M: as someone has mentioned that there is cancer of the breast and others. Everything is cancer but the location determines the name, number 4 just talked about breast cancer but the one we want to talk about is cervical cancer. I will describe it then you can tell me what you call it. I will describe it then you can give me the name. if we see a woman that is above 40years and she is passing out blood from her vagina with odour and loss of weight, some die from there. Has anyone seen that before?

1: I have seen it before and I have heard it before but those days we didn’t know what it was called, they call it ‘atosi’, they may say she had the ‘atosi’ and didn’t get proper treatment . we didn’t know it was cancer those days

M: some call it ‘atosi’ any other person?

2: what I want to add is that is that we cannot see what is happening inside a woman’s body but people can say she has AIDS

3: some call it ‘iju’ such a woman’s stomach will keep getting bigger, we call that ‘iju’ in Yoruba and she will be passing blood from her vagina, sometimes they will have to do an operation to block her womb and she will be passing out blood ((inaudible))

((phone rings))

4: as they have explained, what we know is that when a woman sleeps around, she has sex with everybody and she will not know what each person is carrying in the body, that is how she will contact different things ((inaudible))

M; you have mentioned a woman that is pregnant, what if we now see a woman that is not pregnant and she is discharging blood, what is that called in Yoruba? Its not that she is pregnant [a man?] no a woman that is above 40years and she is discharging blood, is there a name for that?

3: it is ‘iju’

M: so what do we think cause it? let me mention that what we call that is cervical cancer, that is cancer of the cervix and not the womb. What are things that you think can cause this?

1: many women do no not have self control, some can have sex four times in a day. That is where they get all these diseases

2: what causes this thing is that when you get an infection and it does not get treated completely. Like he said that women contact different infections from sex, one infection would not have been healed before they get another one. And it will now lead to such a disease that cannot be treated

3: what I want to add is that when the womb ruptures, when a woman sleeps around too much her womb will rupture and she will start passing out blood

4: using too many drugs can also cause it

M: how can this disease be prevented?

1: the only way to prevent contacting this disease is to do constant check up, even routinely checking blood pressure can prevent such disease

2: what can prevent the disease is that women should have self control

3: what cause it is too much of sex and they will now use drugs, that is what will cause the blood that flows from the vagina because the womb will have been opened up

4: another thing is that women should keep themselves and not sleep around, it is not everyone you should have sex with

M: thank you sir, is there a way that this disease is treated locally? If we see someone that has this kind of disease in this environment, how will they go about treating it?

1: we have traditional healers that can heal that, if someone says she has this disease they can treat her. But its just that some people would have been suffering from this disease for a long time but they will not speak out, it is when the now get to the hospital that they will see that it has become something they cannot treat. So it can be treated traditionally and they also treat it in hospitals

M: looking at the traditional way of treatment, what are the things they give? Is it herbs or how do they treat them? I can see someone smiling that am I sure I want to go there?

2: on traditional treatment, some use herbs, some use bark of trees, it is the one each person knows that he will use but we cannot say in particular that this is what they use

3: in those days, our fathers knew all these things but these days its like all those things no longer work and this is because they said they can cut it off, they may cut the whole womb off but these days most people go to the hospital

M: my next question is that has anyone of us heard the word HPV before?

1: yes I have heard it before but I have not seen it

M: where did you hear it?

1: I heard it in the hospital ((participant coughs))

M: has any other person heard it?

3: I have also heard it before but I have never seen it before

4: I have not heard it before now

M: okay, HPV is what we call Human Papilloma virus and it is contacted during sexual intercourse, this virus will be in the body of the woman over time and ends up causing the cervical cancer in the body. This virus is the organism that causes cervical cancer. There is now a vaccine that is available for children from 10years that have not initiated sexual initiation to protect them from contacting cervical cancer. Do you think this is a good idea?

1: it is a good idea because we are giving birth to different children these days, so if we get a way to prevent these children from contacting these diseases, we should do it

2: these programs from the government is a good thing, they give children of 1year to 5years vaccines that will protect them from these diseases. In those days there were herbs we use but now they don’t know how to combine the herbs so it no longer works, if they combine it the way our forefathers used to do it, it will work

3: it is just the way they have said it, since they said the vaccine prevents the disease, once a child is 9years to 10years, we should let them go. Most of these children already have sex by the time they are 10years

4: I just want to add to the things they have said, these children start having sex early so these one they have made to protect them is very good

M: you know they say whatever has good sides will also have the bad sides, what can we say are the disadvantages of administering this vaccine to adolescents?

1: there is nothing these white people make that is not good except we don’t use it well, for example I was a girl’s house on Sunday, I met her in her school, she went to abort a pregnancy and instead of going home she went to school. If you see the way they were packing blood, I had to go to her house and tell her parents to take care of her and told them what the girl did. But there are different parents, the girl’s mother did not do anything about it. I have not seen her till now, I just saw her yesterday when she was fighting with her sister and her mother was correcting them. Some parents will be the ones that will their children to abort, they will know about it, the girl will go and abort but will not go back home after aborting but after she will go to school. So this type of vaccine is good, as a parent you can do it for your children, atleast you will know the child is prepared for this type of disease. Anytime any day she will not experience sadness over the child but we pray that God will give us children that will listen to us.

M: does any other person have something to add? What are the negative effects of administering this vaccine to adolescents?

2: after God, it is the government, this step that you literate people have brought is good. And God will keep helping you. God will let all these things you do work well for us. we don’t know any other place to get treatment asides the hospital, even for us that are this old, we no longer use all these local mixtures. All our hope is in the government and we pray that they will not mislead us.

M: so let me give us more information on this HPV vaccine, it is available in our hospitals and it is for children that are 10years to 13years. The child will get two doses and each dose is 7000, and she will get it twice. That makes 16000naira. It is available now. I want to ask if you as a parent will allow your child to take such?

1: as community members, that money you just mentioned is too much because fingers are not equal. We can see people that can afford to do that for four children at once and we also have those that cannot afford to do it for a child. The idea of the vaccine is good but that price will discourage a lot of people. If we present that to the community and inform them of the cost, see us as community leaders we use our own money to take care of this community, we tax ourselves. We are interested in this vaccine and we support that it is a good idea but how can we reduce that price? We have agreed that it is a good thing but the price is the challenge. Even if im not here, everybody will be complaining about the money, there is nobody that will see a good thing and will not be interested but you money is very essential. Most people have agreed that they will want the vaccine but the money will push them away. This period we are in, a lot of people cannot even feed, maybe if it was that time that the economy was good and you can borrow from someone but now nobody even has to borrow. Even rich men are not spending anyhow. How can you help us reduce that price, we support it but the money is an issue

M: so you are saying if the money is reduced you will allow your adolescent get vaccinated?

1: yes

2: according to what chairman said, that is exactly the way it is. The way I am interested in this vaccine, another person may not be interested in it. God should not let us and our children face sickness [amen]. When one has a sickness, you will spend more to treat it. but fingers are not equal, some people now will not even mind if the government can help them in feeding their children. Meanwhile there are some other people that can feed their children and will also go to any extent to ensure that the child is protected from any illness, just the way we sleep at night and lock the doors. So help us tell those that sent you that if they can reduce the cost our community members will accept it. God should protect us from sicknesses, we don’t follow our children everywhere, we don’t know the things they do, so God should keep them for us.

3: I want to ask a question, is this vaccine from the federal government or state government?

M: it doesn’t belong to federal or state government

3: those that organized that thing- if they want people to take the vaccine, they should reduce the price. People don’t have money to pay school fees, they don’t have money to buy food, pensioners have not been paid, workers are not being paid so where will anyone get the 16000naira for vaccine. Please help us tell them to reduce the cost ((a participant prayed for the moderator and left))

M: asides the price, can you allow your adolescent to get this vaccine?

3: yes

M: does number have anything he wants to add?

4: it is the same thing they have said, it is a good idea but help us tell them to reduce the money. With that cost, nobody will get the vaccine. It they reduce the price then I can allow all my children to get vaccinated

M: my last question, so if we say we should remove the challenge of money, how can we ensure that our adolescents get vaccinated? Where should it be administered?

1: the best way to go about it is through the community [yes it is the community] from the federal to the state, from state to the local government. The local government will take it to the CDA, this is a CDA, community development association, and community development council is different, that one is in the local government. So if they go through that means from the top to the bottom it will go round

M: does any other person have anything to say?

3: that is the way it is

1: if you take it through the community it will circulate. As you have come here, you should go to the local government and ask for the date of their meeting, then the local government will take it to the state and then from the state to the federal, that is the way they do it. Those that come to introduce things to us will go through the local government, the whole local government chairman will be on seat. At the state level, the whole CDC will be there, right now Ibarapa is 21local governments to ogbomoso, so you go through the local governments and it will be well circulated.

M: does any other person have something to add? How can we get across to the adolescents?

3: what they can do is that they will organize programs for students so that they will tell their parents. Then you will inform the local government

4: if you do it the way they have mentioned it will get to everybody

M: that will be all, thank you for your time
