# Supplementary material for: “She must have been sleeping around”…: Contextual interpretations of cervical cancer and views regarding HPV vaccination for adolescents in selected communities in Ibadan, Nigeria
Source: PLoS One. 2018 Sep 17;13(9):e0203950. doi: 10.1371/journal.pone.0203950 (PMC6141096; doi:10.1371/journal.pone.0203950)
Supplement: S1 CaCx data — (ZIP) [file pone.0203950.s002.zip › FGD_FEMALE PARENTS_40AND ABOVE.docx]

**Interview group: older mothers of adolescents**

M: good morning ma, my name is …………………… and my colleague is ………………, we are here to ask you questions on cervical cancer and we assure you that everything you tell us will be used only for the purpose of this research. am I permitted to ask my questions? [all: yes] and I am permitted to record this discussion? [all: yes]. Has anyone heard about cervical cancer?

1: they say it in the hospital that there is a cancer that is usually in the vagina. They even said we should come so they can check our vagina but they have not called us back

M: so you heard about cervical cancer in the hospital?

1: yes I heard it in the hospital

M: who else has heard about it?

3: yes I have heard it before when I went to lafia hospital in mokola, they did test for us and they said I was fine

8: I have heard about this cancer before but I have not experienced it and I pray that we will not experience it in Jesus name

M: where did you hear it?

8: I heard it on the television and in a hospital

M: this one we are discussing is cervical cancer, so I will explain how it presents incase it has another name that you call it. if we see a woman that is 40years and above, discharging blood and she is not on her period, it will be producing odour, some will have back pain, some will start loosing weight and can lead to death for some. Has anyone of us heard such before?

11: no I have not heard it. the one I know is breast cancer and it happened to someone close to me

M; that is breast cancer

8: is it this cancer that makes a hole in someone’s vagina?

M: that is different

8: it is that one I have seen before when I went to the east, there was one of my uncle’s daughter that had a hole in her vagina and urine comes out from there

M: so there is no one that has seen such before? What of ‘eda’?

4: ‘eda’ is when a woman has sex with a man, the sperm pours out of her vagina, it is not blood that she discharges

M: so are there other diseases that is close to what I have described?

3: no. see all these things you are saying we don’t know it. when we were giving birth there was nothing like all these diseases

M: so what do we think can cause this? what can make a woman discharge blood?

4: that is what I was going to ask, what causes it?

M: you know I already said I will be the one asking the questions, what can cause it?

All: we don’t know

8: what i think can cause it is civilization and there is economic recession. People need women and women that really want to make money can contact all these diseases when trying to collect money from men. Where I sell, I have heard that some women were kidnapped and then they will have sex with her and she will keep bleeding

M: so what else can cause it? we have heard different things. Some people have said it is because the woman sleeps around. Do you understand my question now?

2: I heard on the television that a woman may be pregnant and then aborts the pregnancy, they said a virus can enter her body in the process of that abortion, maybe that can cause cervical cancer

M: number 3, what do you want to tell us?

3: I spoke the other time

M: you will still tell us more

8: toilet disease can cause it and they say that we should not be scratching our private part or poking finger into our vagina, it can injure someone. It can cause an infection especially when you have scratched and the place is sore, any virus from the toilet will easily mix with blood and may lead to this type of disease

M: number 7 what do you think can cause it?

7: I don’t understand this thing

M: so if you see a woman discharging blood, you cant-

4: from what I have seen before, women usually have fibroid. Doctors say our womb is like a land where things grow and whenever the land is watered things will grow, this cancer you are talking about may be one of the things growing in a woman’s womb that is making her bleed. The disease will be inside but it isn’t until it is very chronic that the bleeding will start

M: thank you, I like how you explained that to us

6: I think it may be that someone has not stopped menstruation but the blood has stopped flowing, it is later that she will now start bleeding

M: number 5 what do you want to add?

5: I don’t know anything that can cause it

M: you don’t know what can cause it? number 7

7: we that are here do not have this disease you are talking about but the way the youth of these days live their lifes make them come down with these diseases.

M: so how can one prevent cervical cancer?

6: see some women have used themselves anyhow all in a bid to make money. They have forgotten the God that created them and done different things so there is no way they will not have all these diseases

M: number 10

10: I don’t have anything to say

4: I want to support what she said. We women that are mothers have a lot of work to do and God should help us. we that you called do not have much to say because everything that is happening these days are things we have not seen before. Many of those here gave birth at home and after labour will stand up to tell people she just gave birth and nothing will happen to her. See us here. But these days- there should be a stage a girl should get to before moving near boys and there should be a stage a boy gets to before he can meet girls but these days there is nothing like that, they don’t get to that stage. And in a bid to make money they just have sex with anybody. Please, is this not enough to cause this disease in the future? God should help us

3: when we were giving birth, there is this water that comes out, then after 40days another one will come out if you eat good food. But these days, our children do not expect that such things will happen, so after 8days of giving birth they will start having sex. We tell our children that though they are trying to show love, they should know how to take care of themselves. Having sex that early after birth can cause this disease. You know the womb is a hole, some things that should come out have not come out and you are pushing other things in, so the way to prevent it is to know how to keep yourself

M: thank you ma. I will still tell us what has been proven to cause cervical cancer but I wanted to know what you think. So in what ways can one prevent cervical cancer?

11: a way to prevent this disease is by being careful and keeping yourself from a wayward life, if you don’t do wrong things you cannot have the disease

M: so what are these wrong things?

11: an example is that you give birth and normally your body will not fully recover until 3months after but after 14days they would have started having sex, she may not even be having sex with her husband alone.

M: number 10 are you sleeping

7: after some people give birth- theses children of these days- in our own days we will not have sex until the child is 2years and has stopped breastfeeding but these days they will give birth today and have sex just one week after. Those are the things causing these diseases. Someone that just gave birth and she would start menstruating when the child is just 3months. how can someone be menstruating with a 3months old child? They will now use a cloth that they have used as rag, how will that one not contact disease. That is what I think can cause it. all these things you are asking us concern all those women that want to do something because their friends are doing it. those days, clothes are cheap but now they will buy clothes for 10000naira and they will want to buy clothes for occasions weekly, how will that one not sleep around? How will they not contact diseases?

3: what I want to add is that we should keep advising those that do all these things, God should be helping us. there are some women that cannot keep their eyes off men, those are the type of women we have these days, they are the ones that call men to come and help us, that is what is happening. God should just forgive us. they will have sex with this one and with another without knowing the disease the person carries. You still have your own in your body you will now add another person’s own to it. they will sleep with two to three men within two weeks. That is where all these things come from, God should not kill our children for us.

9: I want to add to what our mummies have said. In our own days, there was nothing like pad, we would use cloth and our parents would teach us to spread it in the sun and when there was iron they would tell us to iron it so all the germs in the cloth can be killed. But nowadays , these children do not have time for that, if you see the pad some of them use- they will throw their pads in very open places where people will see and curse them, how will such a person not have all these diseases. Secondly, cleanliness, children of these days don’t have time for cleanliness especially when they are menstruating. And to add to what she said they cannot stay away from sex. You will see many of them that just gave birth will start been so cuddly while still in the hospital, what do you think will happen when those ones get home. They will be romancing the husband in the hospital and put him in the mood for sex, what will happen when they get home. Most of the ladies these days will not be okay until they sleep with about three men, we didn’t do that in our days. That is why they have different diseases now. We feared God and our husbands, that no longer exist. And that is why they have all these diseases.

5: I want to add to what our mummy said. What she said is a way, but what I want to add is that we parents should talk to our children, let us teach them the word of God. The muslims have the word of God and Christians have the same thing. If you also make them to know the fear of the lord, they will be afraid of these things. Our imam will say that if you make them fear you as parents but do not teach them to fear God, you have made a mistake. He said this is because there is a stage a child will grow to that he will think he is already too old to be corrected or beaten, he will not fear you again but then the word of God will keep getting stronger in his heart. Fear of parents gets reduced in some areas but the fear of God cannot reduce, rather it will keep getting stronger especially if he hears about it weekly. So we parents should talk to our children about the fear of God. All these things are as a result of civilization and this is what is causing all these diseases. We didn’t know about all these cancers growing up. It is possible there was cancer than but it wasn’t this common and there were herbs they would use to cure it that nobody will know. But these days it is so common and it is from sleeping around. You doctors tell us that there is a stage that a child should get to before having sex, but now we will see an old man sleeping with a nine year old girl, how will she not have disease? They have ruined her life. so the cause of all these diseases is civilization and the search for money. God should help us.

M: thank you ma. You mentioned that there are herbs used to cure cancer

5: yes

M: can you tell us about these herbs, asides the white man’s drug that they have brought, what are the local things available to cure cancer?

4: in those days- I don’t know what they use for cancer specifically but those days we used to have worms, such that a child will eat so much but will keep loosing weight. My father will enter the farm and cut a leaf, he will tell us to eat two cubes of sugar, then give us the herb , we will pass out all the worms. I am sure they had things like that they use for cancer.

M: thank you ma

8: on that question you asked that is there any local thing that can be used to cure it, I know there is something they use for cancer of the breast. I was aware of it when my mother had breast cancer, she had it like two different times. And there was something she used in the village, it is called ‘pandoro’. It is long like this. I know they used it with its bark for cancer of the breast. That was what they used for my mother and it worked. They ground the ‘pandoro’ with ‘atare’ to form a paste that she was licking. I heard that one must not walk across that ‘pandoro’ whoever does will have cancer on the thigh. And it is the ‘pandoro’ they will now use to cure it.

11; I want to add to what the other mummy said. When we were young ladies and is on our period, we will buy a yard of white cloth and divide it into three. When you use one you will wash it and take another one. I was not educated, my mother was not educated but when it comes to taking care of children, they did it well. If one give births and put a used cloth to stop blood, you can get diseases from there. We didn’t have what they use these days, it is the white cloth we will use and there was nothing like disease.

8: what I want to add is that it is not just from clothes that one can get infected. See all these pads and toilet roll they use now, if one uses it from morning till night, she will be smelling. Whatever you use should be kept clean and should not be on you for too long. It is irritating for me to take a cloth for menstruation, then was it to reuse. The only reason I may wash is that so it will not irritate another person that sees it. I can use it again. Then the talk of this menstruation we have talking about, how many children these days tell their mothers when they start. The first time I saw blood, I was crying. My siblings were making jest of me that I have an injury in my vagina. It was my grandmother that called me and sent my siblings to get a chicken, she killed it and told me I had become a lady. She didn’t tell me what happened to me all the while, it was later she calmed me down and explained to me. These days, there is no child you will ask- infact you will not know she has started menstruating. The child will be disvirgined and you will not know. Those days, my father would tell us that if a man touches you, you will get pregnant and you must not abort because if you do you will go to hell, that God will not forgive you for murder. That created a fear in us. I hated boys while growing up, not the type of hatred that- my younger sister had gotten married and her child was 5years before someone could ask for my hand in marriage. That’s for someone like me that is this young, now imagine what it was like for those that are way older, adults had a way of putting fear in the hearts of children but these days, our children are exposed. The things they teach them in school is more than what we teach them at home and they will use their brain and try to practicalise what they are taught. Now they say we are in computer age, the things we did not know, they know. They say we are old school, they know too much. Before you ask them one thing they already know everything. They know the different drugs, they will use another one before one finish its work and that can also cause damages in the body.

M: thank you ma

9: in addition, all of us that are parents should go back to the lessons our parents taught us. if we teach them at home to fear us and fear God all these diseases will reduce. It is our job to counsel them, both boys and girls, let us counsel them. We should not be too strict with them or chase them out of the house. You can’t be beating them for everything, beating makes some children become very naughty, you just have to talk them calmly and advising them will make them listen to you and not copy their friends that have three to four girlfriends.

M: thank you ma. Thank you all, you have explained these things well. The other thing I want to ask is that has any one of use ever heard of HPV? ((no response)) okay. HPV is the organism that causes cervical cancer. And it is contacted from sexual intercourse. It is possible to contact the virus at a young age but not develop the disease till old age at about 40years. So this virus is contacted from sexual intercourse. It may turn to the cancer in some but for some it will not. There is something else we call vaccine, has anyone of us heard about HPV vaccine?

All: no

M: so there is a vaccine available for those that are not sexually active to prevent HPV. It is in two doses and each dose is 7000naira making 14000naira. They said children should get the vaccine before they are exposed. Do you think this vaccine is a good idea?

4: it is necessary with what children do nowadays because we do not go out with them ((session was rowdy))

M: let us talk one after the other. Let us start from number 1

1: it is necessary to administer this vaccine at a young age so that they will not have this cancer later

M: number 3, you were saying something

3: I was saying this vaccine is good since we will not be following them out. Even men- God should not let them contact all these diseases. So it is good to give them all these things to protect them. We will tell our children and grandchildren to get this vaccine because when these problems start, some people will just keep losing weight till they die. Is that a good thing so God should keep our children from diseases.

4: it is the same thing they have said, the vaccine is a good idea. We will tell our children that have not started having sex to get the vaccine, God should provide money for us because money is everything. Now you said it is 14000naira, God should provide for us. We will allow our children to get the vaccine

M: thank you for helping me say the amount in Yoruba, I was thinking about it ((respondents laugh))

1: I will suggest that the money be reduced ((all respondents agreed)) because some of us don’t have the money to eat. They should help us reduce it, if possible. That way we will all get it

8: that is the same thing I was going to say, if they can reduce the cost, more people will go for the vaccine. Some people will be interested but the money is too much so they will not be able to get it. but if it is reduced, it will help more people to get the vaccine.

9: that money is too much. We all know what is happening in the country now, there is no one that will this and not be interested but that money will put them away. If they reduce the money, people will get it.

M: thank you. My next question is that what are the challenges you foresee in administering this vaccine to adolescents? Are there some fears you have about this vaccine?

9: there are challenges there. It is not all parents that follow their children to school or go out with them. Most children will say since their parents have allowed them to take the vaccine, it means they have freedom to do whatever they like, they can now have three boyfriends that can buy things for them and give them money. The mother will just notice that the child is changing clothes, buying shoes, she will give her food and she will refuse that she cannot eat without meat. That is the challenge I see there

M: number 3, you have not been talking, I am coming back to you.

4: I just want to support what she said. This vaccine that you said can be used to prevent cancer- we don’t go out with them, they will say they have been vaccinated so nothing can happen to them. They can have ten boyfriends, sleep with one today and they will be fighting on boys ((laughs)) we see these things on television, they will beat themselves up. So how are we going to tell them about this vaccine that you said can protect them from cancer and not push them to get worse?

M: number 10, you have not said anything. You will say something after number 8.

8: please what I want to add is that if people start turning out to get this vaccine, they should keep singing it to them that AIDS is real, they should let them know that it does not protect them from other diseases. Let them know that AIDS is real, they should not think that since they have this disease that they are free from all diseases. They should let them know that it is just for HPV

10: with all these things we have been discussing, the only thing is that God should help us. Those days, there was nothing like taking an injection for family planning, this is what is causing all these issue of sleeping around, they will say they have already gotten an injection to prevent pregnancy. You will see them in uch, adeyoro with very young babies, the husband will hold the baby and they will go in to get the injection. If we now have that type of injection for adolescents, things will get worse. Because the family planning injection that some ladies have gotten has spoilt their wombs and they can no longer give birth, and she will be saying she has never aborted. Her parents will also say she never got pregnant or had to abort, they will know that she did family planning before. I have seen a nursing mother that did family planning and she has not been able to give birth to another child till today. So with these things, I will say that God should help us put fear in their hearts and they should listen to the things we tell them. This vaccine you have brought- we will tell them our own at home but they will introduce them to it in school. There are a lot of things we do not know that they already know about from school. Do you understand? There are things we do not know that they have seen in films. With this civilization, if you leave a 4year old girl with a 4year old boy on their own, don’t let me deceive you, they will do things their parents do. [yes they do it] it was not that they learnt it from their parents but they saw it in films. They can see this vaccine you are talking about in a film, something that we do not know. They would have heard of the vaccine and know what it does, they will go for the vaccine without informing any of the parents.

M: thank you ma, we have discussed the challenges around administering this vaccine to adolescents. For each one of us, will you allow your adolescents take this vaccine?

10: for me as a person, I cannot allow my adolescent to take the vaccine

M: why not?

10: with the way I am saying I cannot take it for my child but if the child gets it on her own, there is nothing I can do about it. but for me to take my money and give her to go and take the vaccine, I will not do it. I know before I get to know about it, the child would have known about it

M: thank you

1: it is the same thing for me, it is very delicate. They are all too smart for us their parents ((all agreed))

M: please let us take it one after the other

5: yes they already know about all these things, they would have been looking for the money for the vaccine and we will not hear about it

8: for me, I am sorry to mention this. I have never taken the family planning injection because I have been scared, I see people that say they do the family planning but after 4years, they will want to get pregnant but it will not be possible. Asides that, my senior sister did family planning and she fainted two different times after bleeding for so long because what she did wasn’t good for her. She had to go back to another one. Her menses want regular. Because of the risks involved, I cannot say I will do it for my child [m: including vaccines?] yes even this one we are talking about. This is because nobody knows the risks involved yet [you mean side effects?] yes side effects. There is nothing that has a good side that does not have bad side. I cant say I know exactly what it will do. So I cannot tell my child to get the vaccine. Also, it will give the child confidence to do whatever she wants, she will say you have given her the vaccine so she can sleep around. So for security purposes, to guide the child, it is not okay to give the vaccine to adolescents. The idea of the vaccine is a good thing but it is not good enough for a parent to take the child for such. It is not okay. If the government can make it free and they give the children in school, that will be fine but for a parent to take the child for the vaccine, it will mean you are giving the child freedom to sleep around

6: I do not support the vaccine and will not allow my child take it because it will mean I have opened the floor for her to sleep around. Her mind will be at rest that since she has been vaccinated she can do anything with her body

M: does that mean there is no one here that will allow her child to take the vaccine?

11: for me, as an educated person I cannot allow my child take the vaccine, because it is as if I am showing the child how to sleep around. We are in computer age and those children are smarter than us, they know these things more than us, they will sit like this and they know things we do not know. If they see a film, they will tell you things they have seen it. the best thing is to sit with them and teach the word of God, train them with the bible, that is the only way I think they can be helped. We should not be the ones showing them the way to destruction

4: as they have said, I cannot allow my child to take that vaccine. As she said that there is nothing that has a good side that does not have a bad side. For example, I did family planning 20years ago, it got a stage that I was menstruating every 16days. I had to go to the hospital to tell them to remove it. I kept loosing weight, if there was cancer in those days they would have said I have this cervical cancer. But then I was still menstruating before I was told that I was reacting to the family planning I did. If we now get this vaccine for our children, do we know what the side effects will be? So I cannot do it, God should help us. as she said, what we should teach them is the word of God. God should keep them for us. it is a different thing to teach them something and they hear [God will let them hear] another thing is for them to hear but not use it. God should help us. there is none of us that serves tree, we all serve God and we teach our children. God will let them hear. So the important thing is to teach them the word of God and we should keep advising them, God will let them listen [amen]

M: so there is none of us that can allow her child take it? ((no response)) answer me, is there anyone?

All: no

M: so none of us will allow our children to take it

4: there is no one, but if there is a reason why you think we should let us know

M: I have explained the advantage of the vaccine, to protect from contacting HPV

1: but what is the essence if the child will now start sleeping with 4 to 5 men because she has taken the vaccine

M: someone is talking there, let us hear her

12: you know with the kind of our own job, we cannot take a stand here on whether to take the vaccine

M: thank you ma. So if the vaccine should be added into the routine immunization schedule in Nigeria, what should be done to ensure the adolescents get it. someone mentioned that the cost should be reduced, so what are the other things that should be done?

9: they can take it to phc and in the hospitals that are capable of administering such

M: are there programs that should be put in place?

8: my own is that those that want to introduce this thing should check the side effects, that is the main thing. A vaccine that I will get for a child that the child will be having problems, so they should let us know the side effects, if there will be no side effects they can take it to phc centres, to schools, wherever they want to put it but they should first check the side effects

M: does any other person have something to add? Number 2 we have not heard your voice

2: it is the same thing they have said

M: say your own, what can we do to ensure our children take this vaccine?

2: it is still what she has said, they should put it in phc centres, like we are here now, you can use this same place. They can take it to adeoyo or other government hospitals, places where they have good nurses that can test the children and know that it will not have side effects

3: it is the same thing, God should not let our children have all these diseases. You cannot be taking care of a child that is now a vaccine that will cause a problem for you. God will not give us mothers and grandmothers a bad name, if it is now time for the child to give birth, and there is no pregnancy. We will now have to start looking for money to get a solution for her to get pregnant when we do not even have enough to eat. God will not make any of our children barren

10: what I want to say about that injection is that- it is in two ways, most of our children, especially the ones in secondary school, there is none of them that will see a boy and get home to tell the mother about it that they slept with a boy and you mentioned that the vaccine is for those that have not had sex before. If it is possible they should not give these children the vaccine. It is only in church that you can indeed know those that have not had sex, if they say those that have not had sex to pray for someone, you need to see the youths’ faces- [they did it in my church, only one person came out] you need to see them, nobody could come out. Is it those people that you are now saying should come for vaccine, she will be shy and go for the vaccine so that people will not know that she has had sex. So it is better if they do not introduce it to them. These vaccine we give children, someone once said we should be careful with it because it was too much, there was one every month, he said the idea of the children immunization is to reduce the number of children we give birth to. Even me I did not allow my child get immunization after then and told the one that was going to school not to take any vaccine in school, that she should tell them she has gotten. I know they will just drop it in their mouth, those days ours was on the arm but it is not like that. They will put it in their mouth, you don’t know what it will do in their stomach. The way things are these days, you cant just take chances, you cannot turn another person’s child to your own. We cannot say because we don’t want to get a disease now get a vaccine that will not let us get pregnant. The joy of marriage is childbirth, if after three years of wedding and there is no child, everybody will be disturbing you. They will be giving you different suggestions. The best thing is for everybody to talk to their children, God will make them hear.

M: thank you so much for your time, that will be all.
