# Supplementary material for: “She must have been sleeping around”…: Contextual interpretations of cervical cancer and views regarding HPV vaccination for adolescents in selected communities in Ibadan, Nigeria
Source: PLoS One. 2018 Sep 17;13(9):e0203950. doi: 10.1371/journal.pone.0203950 (PMC6141096; doi:10.1371/journal.pone.0203950)
Supplement: S1 CaCx data — (ZIP) [file pone.0203950.s002.zip › FGD_JUNIOR STUDENTS_FEMALE_PUBLIC.docx]

**Interview group: Female Junior students public school**

M: good morning [all: good morning ma] my name is ………. and with me is ……… [all; nice meeting you] nice meeting you too. We want to ask you questions about cervical cancer, hpv and hpv vaccine. We want to know what you know, what you have heard, what you have seen and I assure you that everything you tell us will only be used for the purpose of this research. feel free with us. am I permitted to ask my questions? [all: yes] and you permit me to record? [all: yes] thank you. Who has heard about cervical cancer?

8: I have not heard it before

5: we have not heard it before

M: don’t speak for others

5: I have not heard it before

4: I have not heard it before

3: I have never heard it

M: so you are all saying you have not heard it before? [all: yes] do you know what cancer is?

7: I have not heard it before

9: they say cancer is a disease that happens on the breast and may lead to cutting of the breast. I have heard that putting phone near the breast can cause cancer.

M: so you know that cancer is a disease?

9: yes

5: cancer is a disease that one gets when you put money near the breast and when you sleep with bra [no]

M: let her say her own

5: one should not put phone near the breast

M: what will happen if one puts it there?

5: the person will have cancer

M: so some of us know what cancer is [yes] as they have said cancer is a disease. You know someone mentioned that cancer occurs on the breast, cancer can affect different parts of the body, if its on the breast that is breast cancer, it can be on the skin that is skin cancer. So the name attached to it depends on the location. What we want to discuss is cervical cancer. Do you know what womb is? [all: yes] number 8 what is it?

8: where the foetus stays before giving birth

4: where the child passes through during birth

5: womb is the place where the child stays when pregnant

M: what number 4 explained is vagina, so yes that is womb but this one is cervix, the entrance of the womb. That is cervical cancer. Has anyone heard that before?

All: no

5: no but we hear of cancer

M: so you have heard of cancer but you have never heard of cervical cancer?

5: no

M: so let me describe the cervical cancer. Number 1 I have not heard your voice. Let me explain the symptoms, maybe you have seen it before but you do not know the name. number 8 wants to say something

8: I have heard that they had to remove someone’s womb because of cancer and that the person cannot give birth again. They brought it out through operation.

M; where did you hear that?

8: there is one of my neighbours that works in UCH

M: so she is the one that told you about it?

8:yes

M: so let me explain cervical cancer. If you see a woman that is 40years and above that is discharging blood from her vagina and she is not on her period, it will have odour and some will start loosing weight, some will have back pain and some can die from that. And it occurs in women. Has anybody heard that before, or you saw a woman that is above 40years that was discharging blood?

5: yes. The woman was not pregnant and there was day that she sat infront of her house and she was bleeding. They had to rush her to the hospital and they said she has cancer.

M: how old was she?

5: she was about 30years

M: and they said it was cancer. Okay. Any other person

8: when my sister was about 10months, my mother used to complain that she was having back pain and that she has not menstruated since she gave birth, that maybe is because she had not menstruated. She went to kola daisy and they told her it is the menstrual blood that wants to come out that she should be taking blood tonic

M: that is not cancer. Has anyone seen that before?

5: yes. She was loosing weight and was having fever

M: is there a name they call that?

5: I only know it as cancer

M: there is no other name?

5: no

M: so what she described may be cervical cancer. Can anyone think of what can cause this cervical cancer?

4: using another person’s bra and pant

5: number 1 is not talking, are you hungry?

9: wearing a pant for two to three days and if such a woman is raped by many men

5: wearing a pant for long. Some people complain of burns in their thigh, is that cancer?

M: no

4: some put money and phone near the breast

M: that is breast cancer, what of cervical cancer?

5: a woman that sleeps around can contact it from a man

M: what caused it in the woman you saw?

5: I don’t know. We go to their house because it is not far from their own but I didn’t hear what they said caused it.

M: but generally you think if someone sleeps around, she will have cervical cancer?

5: yes

9: if one urinates and does not clean the vagina with water but clean with paper, she can have cervical cancer

7: wearing a pant for long

10: if one wear a pant for three days

8: if someone buys drug to use instead of going to the hospital and then some people can wear a cloth fro 3days

M: number 6 I have not heard your voice

2: if one use a pad for long for long. Some people use one pad from the beginning to the last day

6: if someone uses cloth instead of ad for period

M: so how can it be prevented?

3: once one sees that blood is coming out from her vagina she should go to the hospital

4: by not sharing pant

5: by not wearing a pant for long when on your period

6: by not wearing a pant for 2days

8: when on your period you should change from time to time

9: by not using cloth during menstruation and even if you have to use cloth, you should keep it clean and if it is pad you are using you should change morning and night

10: have your bath everyday when menstruating

1: you should have your bath twice in a day when on your period

M; you all talked about period, someone mentioned that cervical cancer can be cause by sleeping around, so what can be done to prevent that?

3: I want to say something on the causes, if after giving birth and the placenta is not brought out it can cause cervical cancer

5: by not sleeping around while menstruating

8: you should not have sex just after giving birth and you should not sleep around because you don’t know if the person you will sleep with already have the cancer or another disease

M: thank you. Has anyone heard of HPV?

All: no

M: let me explain it. do you know what virus is? [yes] so hpv is the virus you contact during sexual intercourse that will be in the body and then cause cervical cancer later in life. you know someone mentioned earlier that cervical cancer is caused by sleeping around, so cervical cancer is from sexual intercourse and it is the virus hpv that one will contact during the sex that will be in the body and will then cause cervical cancer. has anyone heard about hpv vaccine?

All: no

M: do you know what a vaccine is?

All: yes

5: the injection they give children

M: so the vaccine we are discussing is the type they give children so as not to contact a disease. So there is a vaccine for hpv that we discussed, so the vaccine will protect you from the virus. Has anyone heard that before?

9: what if someone gets the vaccine and sleeps around, will she not contact the virus?

M: we will get there. What are the vaccines they give children? Let us use meningitis for example, they give them that vaccine to protect them [yes, they gave us in jss1] . now they have said this vaccine is for those that have not even initiated sex so that you are already protected before you start having sex.

5: hope it does not involve money?

M: it is money. Is the money a challenge for you?

All: yes

M: okay. Let us continue. Do you think this vaccine is a good idea?

5: it is not a good thing

M: why?

5; because it is not a disease that you can see and then go to the hospital to complain

3: the disease is not a good thing because it can prevent you from giving birth

M: I didn’t say the vaccine is for those that have the disease. Do they give the meningitis vaccine to children that already have it? [no] the same way they give hpv vaccine to those that do not have the disease and he=ave not even exposed to what can make them have the disease. So what I am asking you is that is this vaccine a good idea?

9: yes it is a good thing because after getting the vaccine and sleep around you cannot contact the disease

1: it is a good thing because you will be protected

5: if one contacts this disease, how will you know?

M: you will go for test

8: it is good to get that vaccine because if you later start having sex you will be protected ad can just go to the doctor to check if there is another thing wrong later

7: the best thing is to go to your family doctor for check up

4: the vaccine is to protect someone so that someone that has it will not infect you

5: the vaccine is good because you cannot know if someone has it or not

3; this vaccine is a good thing, they have used it to help us

6: it is good to get this vaccine because when you get married you don’t know what your husband will have

9; it is good to get the vaccine now because you will be protected if your partner has the hpv

10: it is good because even if you are sleeping around you will be protected

8: it is good, there is a woman in my area that they she did not allow her child take all those vaccines now the child cannot stand and till now her neck is not stable. They told her to take the child to the hospital and she said she has gone but she was told that she should not have gone to a private hospital but she go to adeoyo or uch

M: does anybody have something to add?

5: at what age should one take that vaccine?

M: from 10years and above before having sex. Number 1 wants to add something

1; if someone does not get this vaccine and then gets pregnant, it can affect the child

M: you know I said it is for children that are 10yeasr and above, do you think there is any disadvantage in giving children of this age?

8: what I can say is that if one gets that vaccine…. Some parents can lie about the age of the child just so she can get the vaccine and it can affect the child negatively

5: it is good for people that are 10years and above because most of them even start sleeping around by 9years. So it is good to get the vaccine early so as not to contact the virus

1: it is good to get that vaccine because by 15years everybody will have started having sex

M; so there is anyone that thinks there is a disadvantage in administering the vaccine to adolescents?

1: so they will not contact the disease

7: so that they will not sleep around

M; I don’t understand you, is it that they will sleep around after getting the vaccine or they will not?

All: they wont

M: they will not sleep around after getting the vaccine

5: they will. I believe that they will know that even if the guy they sleep with has the virus they will not contact it

M: what are the disadvantages, number 9?

9: someone that is just 10years may lie that she is not yet 10years, so there should be something that they will be using to check age

10: some parents can lie about the age of their children so that they can give the child the vaccine

M: okay, let me tell you that this vaccine is not free of charge. You will take two doses and one dose is 7000naira, making 14000naira [all: ehn] ((participants exclaimed)) I just said I should tell you that one. So what are the challenges that you can see? That maybe they give a child that has not started having sex the vaccine-

5: she will now start sleeping around

M: I was asking before you didn’t respond. Say your own

4: if a child wasn’t going to have sex before, if they give her the vaccine that is when she will start sleeping around and having sex anyhow

9; the challenge I can think of is the cost, I will suggest that they reduce the cost of the vaccine because most people cannot afford that

5: if we that are students want to take that vaccine and we go there to beg them to give us free of charge, will they give us? Is the cost from the government or from you?

M: I am not the one that determines the price, I just told you how much they administer it in the hospital

6: a child that does not want to sleep around will start sleeping around if they give her the vaccine because she will believe she cannot contact any disease again

3:if they give a child that hpv vaccine she will believe she cannot contact the disease

1: some people will think that hpv vaccine will protect them from all sexually transmitted infections

2: if someone does not have the money to get that vaccine she can end up contacting the virus

8: what I want to add is that some people cannot afford that money, is there no way they can help us reduce the money so that it does not become a disease we cannot handle in the community

M; thank you for everything you have said. So what are the things that may stop you adolescents from getting the vaccine

3: I have not taken the vaccine because I did not know about it before

9: what can stop most people from getting the vaccine is the money because the money is too much and once someone does not have the money, she will just say the thing cannot kill her

5; someone can have 7000naira but with many children and they do not have what to eat, she will think that instead of spending the money on one vaccine she should use it to buy something for her children.

1: there are some people that believe that they are too old for all these vaccines

2: someone may have 6000naira and needs 10000naira more to be able to get the vaccine but she will not get anywhere to borrow the money

3: some people may even have that money but because they have given birth to many children they will not do it

4: some people may want to take the vaccine but they do not have the money so they will say since they do not sleep around they don’t need the vaccine

8: some people may have the money for the vaccine but they will say their children are not sleeping around and do not need the vaccine but the child will now marry and contact that virus from the husband, then they will end up spending more money to treat it

10: some people may say they do not have the money and so do not need the vaccine

M: you as an individual, will you take the vaccine?

((session became rowdy))

M: let us talk one after the other. We will start from number 1. Will you take the vaccine and you will give us the reason

1: I will take the vaccine because you never know the disease you can contact later and the type of husband you can marry

2: I will take the vaccine because of my future so it will not affect me later in life

3: I will take the vaccine because you don’t know the type of husband you will marry so he does not infect me and get my children into trouble

4: I will take the vaccine so that I will not contact hpv

5: I will because I don’t know the type of husband I will marry, all men now sleep around so that I can protect myself. So I will take the vaccine if the money is not too much

6: I will take the vaccine so that I will not contact the disease in the future

7: I will because I don’t want to have the disease

8; I will so that when I marry I will be protected if my husband has any infection that can cause problem for my womb

9: I will so that I will not have the disease

10: I will because of my future

M: will all your parents allow you to take the vaccine?

All: yes

M: who are those that their parents will not allow them take the vaccine?

9: my parents will not allow me because they will say the money is too much. If it is free they will not be against it but with that amount they will say school fees is still there for them to pay

5; they can let me take the vaccine but the money is the issue. I will still pay school fees and pay for junior waec. If the money is like 2000naira I will take it

6: my parents cannot let me take the vaccine because if I take it my older siblings will also say they want and the money will be too much for my parent to give all of us

4: my parents will not allow me because my sister will also say she wants to take the vaccine and the money will be too much

2: I will because my mother may have the money that she will give me

M: if this vaccine is now introduced into the routine immunization schedule in Nigeria, what can be done to ensure that adolescents take the vaccine?

4: the money should be reduced

8: so that adolescents will not contact hpv

M: you do not understand my question, what can be done to make adolescents take the vaccine

6; they should reduce the money

10: they should reduce the money

M; are we all going to talk about the money?

All: yes

5: we are young and some of us live with our grandparents, if we drop 7000naira just for vaccine, what are we going to eat after that, so they should help us reduce the money to like 3000naira

3: if that vaccine works well and people can see results they will go for it

M: how are they going to know that it works well

3; they will get someone that has the disease and has tried different drugs but it did not work and give them this one to see if it will work

M; remember we said the vaccine is not for those that already have the disease

9:they should announce it on the radio and television

5: they should get a popular person or a rich person to talk about it on the radio, people like us that do not have money will also try to get the money for the vaccine

1: if someone gets ((inaudible))

M: who has something to add, some people have talked about the money, someone said they should talk about it on the radio and televeision, is there any other thing you want to add?

8: they should advertise it on the radio and give a contact on the radio that people can call to ask questions and they should reduce the money

1: I will say they bring that vaccine to schools and give children in the school

5: if they announce it on the radio, if it is a governor, he should be able to reduce the money for children

9: then they should show the people with the disease on the television so that people will see and also reduce the money

8: on what she said-

M: does any other person have something to add?

5: that is all

M: so you have said everything

9: I am also thinking that they should take the vaccine into communities and go from house to house, such that any day they are going into a community the children will be told not to come to school

5: if they take it school, will people bring money to school to pay for the vaccine? Some of us here have not eaten today , you will now tell us to go and bring 7000naira

M: any other person?

1: I think if they bring it to schools, some people may think they it is something bad they are doing

M: so what should they do?

1: if they want to bring it to school, they should first call a meeting to inform the parents before administering it to students

6: what I want to say is that they should not bring it to school, instead we should go to the hospitals for the vaccine and it should be reduced to 1000naira

5: these ones are saying they should bring it to school, some parents will believe they want to do something bad to their children. You will see a child that already has an illness- aunty please how will they know that someone has the disease?

M: you asked before and I told you it is by doing a test

7: they should advertise it on the television and also reduce the cost

9: they should call a pta meeting because some parents may not want to agree

2; some parents do not even come for pta meeting normally it is now this one they will come for. Because of vaccine, they will now come.

M: thank you so much for your time, that will be all. Clap for yourselves.
