# Supplementary material for: “She must have been sleeping around”…: Contextual interpretations of cervical cancer and views regarding HPV vaccination for adolescents in selected communities in Ibadan, Nigeria
Source: PLoS One. 2018 Sep 17;13(9):e0203950. doi: 10.1371/journal.pone.0203950 (PMC6141096; doi:10.1371/journal.pone.0203950)
Supplement: S1 CaCx data — (ZIP) [file pone.0203950.s002.zip › FGD_JUNIOR STUDENTS_MALE_PRIVATE.docx]

**Interview group: Junior male students private school**

M; good afternoon, my name is ……….. and with me is ………... I will be asking you questions to explore your knowledge about cervical cancer, human papilloma vius and HPV vaccine. Feel free to tell me whatever you know, this will be kept confidential that is why we are using numbers and not your names. You wont hear what you tell me somewhere else, it will only be used for the purpose of this research. do I have your permission to continue? [all:yes] and I also have your permission to record your voice? [yes] has anyone of you heard of cervical cancer?

1: yes I have heard it

M: what did you hear about it?

1: cancer is a disease that can lead to death and can lead to so many diseases

M: where did you hear it?

1: I heard it on television and radio

M: has any other person heard about cervical cancer? Don’t shake your head, please talk [all: no] so no other person has heard about cervical cancer except number 1? Okay. As he has said, cancer is a disease and there are different t- did you hear about particular type of cancer, or you just heard of cancer generally? [yes] okay there are different types of cancer, has anyone of you heard of breast cancer? [yes] so that one affects people on the breast, we can have skin cancer, cancer can be anywhere, do you understand? [yes] but the one we want to talk about now is cervical cancer. And you said you have not heard that before? [yes] do you know ‘jejere’? [yes] who knows what jejere is?

All: (quiet)

1: I know

M: what do you know about jejere?

1: (quiet)

M: jejere is the Yoruba word for cancer [1:yes] this cervical cancer is the cancer that occurs at the entrance of the womb. Do you know what a womb is? [yes] who has womb? [girl, women]. So let’s say this is the womb, this entrance is what we call cervix. So when there is a cancer here, that is what we call cervical cancer. Let me describe the symptoms incase you have seen one. Has anyone of you seen a woman that is about 40years and above, you know it’s women that have womb [yes]. So a woman that is 40years and above keeps bleeding from her private part and it is not that she is on her period, she may also be loosing weight she will have a very bad odour and sometimes it can lead to death if it is not treated. Has anybody seen such before?

5: yes I have seen it

M: where?

5: the person that sells eba in our shop

M: is she an old woman?

5: like 30years

M: what happened to her?

5: she was bleeding, my mum said that when she went inside there was bad odour in the place.

M: where did they take her to?

5: I don’t know

M: what did they say happened to her?

5: they did not say the name of the disease

M: okay. Number 1, you also said yes

1: yes it happened to my neighbor

M: can you tell me what happened to that your neighbor?

1: she was bleeding, they took her to the hospital but they did not see what was happening to her

M: where did they take her to?

1: highland hospital

M: did they take her somewhere else after they said they cant see what was wrong with her?

1: I don’t know

3: when we were in the market one day, there was a woman bleeding, they had to call her people to take her to the hospital. She has a shop in the market. The next day they said she did not come to the hospital. After three weeks they said they took her to the hospital and was told to pay a lot of money that they cannot treat her in the country.

M: they said they cannot treat her in the country? Do you know what they said happened to her?

3: no I don’t

M: okay, so what I have described for us is cervical cancer. So what do you think can cause such? (no response). If you see a woman in your neighbourhood, bleeding from her private part and it is not that she his menstruating, what will you think caused it?

4: I don’t know

5: if she is fornicating

3: maybe there are some diseases in her stomach

M: what can cause that disease?

2: abomination

M: how? That she has committed an abomination? [2: yes] what sort of abomination? Say what you want to say

2: if she has been pregnant for about 5months, it can lead to cervical cancer

3: typhoid?

M: any other one? Just think about it, what have heard?

3: maybe she lost her pregnancy

7: I don’t know

8: maybe unwanted pregnancy

6: I don’t know

M: so if you see a woman bleeding now, you will just say you have no idea what could have caused it?

6: maybe stress

M: so you have said the things that you think can cause it, how do you now think it can be prevented?

1: by going to the hospital regularly

4: by taking out time to rest and to work

2: by eating fresh fruit

8: by stopping fornication that they are used to

4: by doing exercise

7: by not having sexual relationships

M: thank you, you want to add something?

3: by doing test regularly to know if there is something inside the blood

M: I will now explain what- you know you have said your own [yes]. I will now explain what causes cervical cancer. During sexual intercourse, you know some things are passed from one body to ther, that is how a virus we call Human papilloma virus is passed from one person that has it to another person. It is that virus that will be in the body till when she is about 40years it will now develop into cervical cancer. Has anybody heard about human papilloma virus before?

All: no

M: human papilloma virus is the organism, do you know what a virus is? [yes] an organosism that we cannot see with our naked eye, it is that virus that is passed from one body to the other and it can only be transmitted during sexual intercourse. If one has sex at 8years with someone that has the virus, she can contact it at that age but it is when she is about 40years, that it will cause disease in her body. Do you understand that?

All: yes

M: do you know what a vaccine is?

1: I have heard about it but I don’t know the meaning

M: vaccine is what we call ‘ajesara’ you know what they give children to prevent meningitis? Yes that is a vaccine. Now there is a vaccine that can be used to prevent cervical cancer before sexual initiation. So that when sex is initiated, one will not contact this virus, do you understand? [yes] so even if one has sexual intercourse with someone that has the vaccine, one will not contact the virus from him or her. Has anyone heard of that vaccine before?

1: yes

M: where did you hear about it?

1: they brought it to my school

M: your school? And they said it is HPV vaccine?

1: yes

M: where is your school?

1: ***** at the back of this place

M: and who were those that they administered the vaccine to?

1: primary 3, 4 and 5

M: how much was it?

1: it was free

M: who brought it to your school?

1: ((stutters)) this people….they were carrying it in a box

M: they have not started administering HPV vaccine like that, do you understand? I don’t think that was HPV vaccine, even now it is just available in some hospitals and it is 7000naira [ehn] I can see your expression. As I have said it is 7000naira and one will take it twice. And they have started people that are 10years to 13years should come for it, how much is that? [14000] you know they said it will be administered twice. Do you think it is a good idea that we have that vaccine? Please speak up

6: yes because ((inaudible))

2: yes because it will help to prevent the disease

1: to prevent

M: any other person? What are the advantages of administering this vaccine? ((no response)) do you all think it is a good thing? [yes] okay but do you think there can be some disadvantages in administering this vaccine?

2: yes if one takes too much of it

M; what can happen if it is too much?

2: it may turn into another thing

M: like what ((2: ….)) you just think into another thing

4: if that person use overdose, it can lead to death

M: you will be given the exact dose you should get, it is not that they will release it like paracetamol so issue of overdose may not come up. Any other person? What reasons can you give on why this vaccine should not be administered to adolescents?

7: if the person grows up and get married she may not get pregnant

M: you think one of the side effects is that it can hinder pregnancy?

7: yes

1: they can misuse it there

M: who can misuse it?

1: the people that are administering the vaccine, they can give someone overdose

M: maybe the nurses that will administer it to someone can give the wrong dosage?

1: yes

M: okay. Any other person?

4: did they say the young ones should not use it?

M: its 10years, those are still young. The vaccine for now is been administered to people that are 10 to I think 12years

4; I am thinking about those that are 1 to 8years

M: for now they have said it is for 10 to 12years. So what do you think can be the disadvantages? Somebody has mentioned that it may have side effects. You want to say something?

3: if someone that is older than 10years takes the vaccine, it cannot work for them again

M: they will take it when they are 10years.

3: if they are 8years and they take the vaccine, will it still work for them by the time they are 10years?

M: yes

5: ((inaudible)) some people may lie about their age

M: why will people lie about their age, it is their money they will pay

5: when they know it is a good thing

M: say it in Yoruba

5: once they know it is a good thing, they may want to lie about their age and take too much and it can lead to unexpected death

M: how?

5: maybe if someone is having sex every day, he may not grow and when the person is 50years or 60years, the person will die

3: they can decide to use plenty of it when they have money

1: if someone wants the injection or what is it now, can they sell it to him?

M: yes it is already available in uch

1: they will give the person the vaccine?

M: they will inject you with the vaccine, not that you will pay and they will give you to go and use it yourself. Has anybody here taken the vaccine? [all:no] number 2 why have you not taken the vaccine

2: because I did not know about the vaccine

1: ((inaudible))

M: did you know about the vaccine?

1: no, maybe my grandmother knows but she did not take it because of the cost

M: now that you all know about the vaccine, will you take it?

7: no ((laughs))

M: why not?

7: because I do not have sex with anybody

M: so you don’t think you need the vaccine

4: because I do not have enough money to take the vaccine

1: it can be useful for the future

M: will you take it or not?

1: I will take it [because you believe it will be useful in the future] yes

3: we can take it but they will use [not we, you?] I can take it but I will be considering the money that it is too much, money is not easy to get

8: I cannot take it because I am above that age

M: it is also for those that have not had sex at all, you are still an adolescent. Will you take the vaccine?

All: yes

M: let him answer, will you?

8: no

M: why not?

8: because I do not have the money

5: I cant take the vaccine because I hate uch ((all laughs))

M: why do you hate uch, let us hear him

5: I do not like going to the hospital

M: is it hospitals generally, or is it just uch?

5: it is uch

M: so if it is in another hospital you will go there

5: yes I can go

M: who have I not asked? You, will you take it?

6: no because I do not have the money

1: I have a question, if someone already has the disease and she takes the vaccine, will the vaccine cure her?

M: the vaccine is for prevention. Do you know the difference between cure and prevention? [yes] prevention is that you don’t have the disease yet, cure is that you already have the disease, that person needs treatment after she must have been tested. That is why they are saying the vaccine is for those that have not initiated sex, so they have not been exposed to the virus at all but someone that has initiated sex, she will first go for test. Were you raising your hand? [I have a question] you should be the ones answering the questions, okay what is your question

4: how will someone know that the virus is in his body?

M: he will go for test

2: can someone that is about 40years and already has the disease get the treatment?

M: yes she needs treatment and not vaccine at that point. For any disease, vaccine is for those that do not have the disease yet. Is it question you also want to ask? You will ask at the end. So I was asking you why you will not take the vaccine as an individual, who have I not asked? Number 9

9: I will not take the vaccine

M: why not?

9: because I don’t have sex

M: and you don’t think you will still have sex?

9: no ((laughs))

M: let us hear number 2

2: I will take the vaccine because it can be useful in the future

M: thank you. Let me ask you another question, will your parents allow you to take the vaccine?

8: ah

2: yes

M: somebody said ‘ah’ let us hear him, why do you think they will not allow you?

8: because I did not inform them before

M: so if you inform them, they will allow you

8: yes

5: maybe they have not heard it before, they will assume that it is dangerous

4: like they have said, if they have not heard of it before they will think that I am lying and will not give me the money

M: any other person? Number 2 will your parents allow you?

2: yes because if they have heard about it they will allow me

M: hope you know you will have to tell them the disease the vaccine prevents, you will tell them that the vaccine is to prevent cervical cancer and that the hpv that causes it is a sexually transmitted disease. What do you think will be their reaction to that?

1: they will be happy

4: they will be thinking about another thing

M: what is that another thing?

4: they will be thinking that I have been having sex

M: any other person? Number 6, what will your own parents say?

6: they don’t have that money but if they have they will give me

M: so they will not mind that it is for a disease from sexual intercourse?

6:no

3: my mummy will give me because they will know that it is useful for me. They will tell me to use it, that it can help me in the future

M; so they will give you the money. Looking at our community, what do you think the community’s reaction will be to it?

1: like how?

M: if people get to know that you took HPV vaccine, what will be the reaction? What will your neighbours say? What will your religious leaders say?

9: they will think that someone is already having sex

7: they will think that you are planning to have sex

5: they will think that once they give me the money I will start having sex [laughs]

2; they will be thinking about many things

M: like what?

2: they will think I am lying and I just want to use style to collect the money. Then they will think I want to have sex

3: they will be thinking that I will start sleeping around after taking the vaccine

M: what of religious bodies, do you think church will accept it? do you think churches will accept it?4

4: no

M: churches will not accept it?

4: no

M: why not? Is it churches or mosque, specify the one you are referring to

4: churches because they will not believe, they will keep thinking that it is because you want to have sex

5: they will be thinking that the vaccine is dangerous

3: if they have not heard it before in a mosque, they will think that the person that brought the vaccine just wants money so they will not take it

M: thank you for your comments. Now we want adolescents to take this vaccine, what do you think can be done to ensure that adolescents get this vaccine and who should do it? you all said you have not taken it, what can be done to make you all go for the vaccine?

3: it should be free for adolescents

4: they can announce it throughout the world

M: who should announce it?

4: government

M: where? In his home? How should they announce it?

4: on the radio, talevision

3: they should involve the ministry of health. Once the minister approves it, they can go to the television to announce it. after announcing, they can release the money for it

7: they should take the vaccine from house to house

8: they should make it free and they should take it from house to house and school to school

3: they should share themselves within the community and have a place where people can go to meet them. They should get a spacious place in the community

1: they should stay in one place and people will know that this is where you administer hpv

2: hey should reduce the amount

M: number 9, what can be done?

9: I have no idea

7: if they tell the world that the vaccine is good and show corpse that it was the diseases that caused the death, and tell them if they don’t want the vaccine to spread in the community, they should take the vaccine

M: how will they tell the world?

7: by using radio, television, newspaper, internet

M: any other point?

2: they should reduce the cost of the vsccine

M: that is my last question but does anybody have something to add? If not, that will be all. Thank you all so much. clap for yourselves
