# Supplementary material for: “She must have been sleeping around”…: Contextual interpretations of cervical cancer and views regarding HPV vaccination for adolescents in selected communities in Ibadan, Nigeria
Source: PLoS One. 2018 Sep 17;13(9):e0203950. doi: 10.1371/journal.pone.0203950 (PMC6141096; doi:10.1371/journal.pone.0203950)
Supplement: S1 CaCx data — (ZIP) [file pone.0203950.s002.zip › FGD_SENIOR STUDENTS_FEMALE_PUBLIC.docx]

**Interview group: Senior female students public school**

M: Good afternoon [good afternoon] my name is ………… and with me is …………, we want to ask you a few questions, it is a discussion and you are the ones that will teach me. Do you understand? [yes] We want to know the things you know about cervical cancer, HPV and HPV vaccine. We want to know what you have heard, what you have seen, and I implore you to open up to us. We assure you of confidentiality and everything you tell us will only be used for the purpose of this research. Do you permit me to go ahead with the questions? [yes] You did not all answer [all: yes] I am permitted? [yes] And I am permitted to record this discussion? [yes] some people did not respond [all: yes] Number 3 did not respond, do you agree? [yes] Thank you. So when I ask question and you know the answer, raise your hand, there should be no chorus answer.

M: Has anyone heard about cervical cancer? ((silence)) who has heard about cervical cancer?

P2: I have heard it, I do not know anything about it but I have heard it.

M: where did you hear it?

P2: on the radio they use to talk about it that was where I heard it

M: Any other person that has heard about it? Maybe you just heard it around. Nobody? Don’t shake your heads, you should talk. Can you see it is not video recording we are doing, please talk. So has any other person heard about it asides number 2?

All: no

m: number 3 wants to talk [no] don’t shake your head. You want to say something? ….[no] she has changed her mind. Okay I will describe cervical cancer, and then I will ask if we have seen it before, we may not what it is called. If a woman that is 40years and above bleeds from the vagina and it is not menstruation, you know when a woman is on her period she has blood coming out from her vagina. Now for the woman, she has blood coming out from her vagina with an odor and she is losing weight, for some it can lead to death. But the starting point is that the woman has blood coming out from her vagina. Has anybody seen that or heard about it before?

2: I have heard it before; it happened in this our school

m: in your school, who did it happen to?

2: it happened to one of our seniors, we were in the class when we heard the news that one senior was bleeding and they had used cloth to keep the flow but it kept pouring. We don’t know what happened to her, we thought it was menstruation but it should not be that much.

m; okay. What number 2 just described to us just looks like it but it is not cervical cancer. do we all understand me? Cervical cancer happens to those are 40years and above. You want to say something? If you see a woman that is 40years and above and she is bleeding, the one you mentioned now is not cervical cancer. Asides number 2, is there anyone that has heard about it? Maybe a woman in your neighborhood was bleeding till she died. Talk, don’t shake your head, number 5 have you heard such before? [no] number 4 [no number 7....I can’t hear you [no] so we have not heard about cervical cancer. What I have explained earlier- Number 8 what is it? Number 9 I’m listening

9: Aunty I have heard it before but not in Ibadan, it was in my grandmother's house in Ilesha.

m: ah ah is Ilesha not in Nigeria?

9: they said she was bleeding and when they took her to the hospital, they said it is cancer she has. It was a week after that they said she died.

m: and they said she was discharging blood from her vagina?

9: yes and she was losing weight

m: with what number 9 has described, did you hear what caused that?

9: I did not hear that but what they said was that they told them at the hospital that it is cancer. That if they had known earlier before it got that bad they would have saved her but it was late and she died.

m; so your grandma did not mention what could have caused it?

9: no

m: she did not tell you? Okay so we all said we don’t know anything about cervical cancer except number and number 9. Even after I explained to you, you said you have not seen such before, am I right? [yes] Okay, the organism that causes cervical cancer is called human papilloma virus that one contacts during sexual intercourse. It is that virus that will be in someone's body...do we all know what virus is? What class are you in? [Science class] good. Cervical cancer is the disease; human papilloma virus is the organism that causes the disease. It is that virus that would have been in someone's body that will now develop to cancer when the person is 40years. Do we understand? [yes] Has anyone heard the word HPV before?

all: no

m; none of you has heard it before? [no] number 6 have you heard it before? [no] number 5? [no] you said what?

5: I have not heard it before

m: number 9 have you heard HPV before?

9: no

m: none of us has heard it before? Okay so there is something we call HPV vaccine. You remember I said one can get HPV during sexual intercourse and that is what will cause cervical cancer. There is now a vaccine that one can be given that will not the person contact HPV. Such that if I should have sex, I will not be at risk of contacting HPV. Has anyone heard about the vaccine before? ((quiet)) Has anyone heard it before? Number 1?

1: no

m: number 2?

2: no

m: maybe on the radio or on the internet? Number 9 you want to say something? [no] number 6 [no] number 7 [no]nobody has heard about it, so we have not heard about cervical cancer, human papilloma virus and HPV vaccine? do you the think the HPV vaccine is a good idea? They said there is a vaccine that will not make someone contact the virus, do you think it is necessary to get that vaccine?

6: yes it is good

m: why did you say that?

6: you know you can trust your husband that he cannot contact HPV but he may end up contacting it

m: okay so that you will not end up contacting it from him?

6: yes

m: what are the advantages in getting the vaccine? if they say the vaccine is available now, will you get it? why will you get the vaccine?

2: i will get the vaccine so that I will not get the disease

m; what are the benefits in getting the vaccine?

4: so that you will not get the disease because the person you marry may end up having the virus so that I will not get it from him

8: ((quiet))

m: these girls want to stand up....everybody get on your feet and you will dance for me, will you dance for me? [no] and you are doing as if you have not heard anything before, this your face I am seeing, don’t you watch TV? We will all dance for me, even those that don’t know how to dance and I will record it and show those boys in your class to see you. Will you talk to me now? Can we continue? [yes] so we have discussed the advantages, we said to prevent contacting the disease, what do you think the disadvantage will be? That if they say they are giving people, you will refuse to get vaccinated. Number 3?

3: I can’t get vaccinated because most times when you get too much of anything it will have something it will damage in your body. [like what] you know there are different drugs for different body, what is this thing to prevent pregnancy called? [family planning] people react to it in different ways, I know someone that said the injectable type did not work well with her body, that she reacted to it, she just started changing but she said she has been okay since she removed it. So some people may not react well to it because our bodies are different.

m: do you mean the side effects? That some negative things can occur in the body as an effect of getting vaccinated?

3: yes

m: okay, let me explain another one, you can tell me if I am right or wrong. You know we said one can contact HPV from sexual intercourse? [yes] don’t you think if we give adolescents this vaccine they will start sleeping around? [all: yes] you said yes, let us hear you number 4

4: someone can say that she already has what can prevent her from getting HPV and so she can do what she likes, since she cannot contact the disease. But at the end of the day, she may not know that the vaccine is already expired in her body and she will end up contacting the disease

m: number 6 wants to talk

6: someone can think that she can do whatever she likes not knowing that the vaccine will only prevent cervical cancer and not other infections like HIV

m: number 9 wants to talk.....she has forgotten what she wanted to say. Try to remember. ... .... so we have said someone can start sleeping around. You know the vaccine is an injection, do you think some people will not get vaccinated because of the pain?

7: yes that is what I was going to say

m; okay so explain, how?

7: as in....

m: please help me beg her, everybody say please [please] so I am listening. Number 8, if you are told there is a vaccine for HPV, what will prevent you from getting vaccinated? number 8

2: I can’t stand injection and since I know I will not move near anything that will make me contact HPV and I don’t even sleep around so I don’t have to get vaccinated

6: that is what I was going to say [say it your own way] that i fear injection, I would rather take drugs than get injection, if it is injection I will not take it.

m: even if they tell you it is preventing a disease, you will not take it?

6: I can try but next time I will not take it

m: just once, but they said you must take it twice, what will happen?

6: then I don’t have a choice so I will take it

m: okay considering our parents, will your parents allow you to get vaccinated?

4: what I think is that my parents will think since they have never caught me sleeping around they will believe I don’t need it. Some parents may not trust their children so if the child asks for such, they will believe that it is because she has been sleeping around.

2: its some parents that will tell their children to get vaccinated. They will tell the child to do what her mates are doing, that since they said it will prevent a disease, they will tell her to get it.

1: some parents may think the child wants the vaccine so that she can sleep around

11: some parents may allow their children to take the vaccine if they are made to understand that the vaccine will prevent a disease that the child can have at old age, they will be interested.

m: If we look at it from the religion side, do you think your religion will support adolescents taking the vaccine to prevent an infection from sexual intercourse?

1: it is for own good so they will let you get vaccinated but there are some that will say it is against the law of GOD

m: they are awake ((laughs))

4: a pastor's child can get the vaccine and people will be backbiting that is because she is sleeping around. Someone can tell her close friend that she got vaccinated and that one will tell the whole world. If someone should take that vaccine and start getting fat at that time, they will say it has started affecting her.

6: some religions like Islam will say it’s against their religion and so their adolescents cannot take it because they term everything in their religion

m: so in this community, does that happen already?

6: not yet but some people will be telling their children not to get vaccinated. We have that happening. They will say they are clerics.

9: especially the muslin clerics, they will tell you everything does not go with their doctrine so they cannot allow them to do it.

4: some people prefer taking local herbs; they do not know that what they are taking cannot prevent these infections. Some of those teblics do not even allow ordinary vaseline for babies, they will refuse, some people will say it does not let the child stand early. They have a lot of things they say about these things.

m: that is on a general basis, now as regards giving the HPV vaccine to a child that is between 10years and 12years, someone that has never had sexual intercourse. number 5 will your parents allow you?

5: yes they will allow me to get the vaccine

m: why will they allow you?

5; because they know it is to prevent contacting an infection

m: do you think money can prevent some people from taking this vaccine? number 3 I have not heard your voice

3: yes [how] because there are times that parents may not have money to even buy books and the adolescent may not have not other means of getting the money so she will not be able to get the vaccine

6: some parents will tell their children they don’t have the money even when they have it. Some really do not have the ability to get the money but if it is free they can get it.

4: there are some parents that even if they have the money they will not allow their adolescents to take the vaccine. [why not] they believe that the vaccine will make the child sleep around, they say it is civilization that has ruined everything, they believe a lot of these injections are bad. And even some children, once they know they have the vaccine that is when they will really go ahead and sleep around, she will become a flirt. She could have been interested in sleeping around, if she now sees something that will protect her from infections she will now go into it full force. Some adolescents already have lovers that can give them the money and nobody at home will know. But for someone that doesn’t have any lover that can give her the money, she will have to ask her parents and once she tells her parents and mention anything sexual intercourse, they will say she has started sleeping around. We young girls, once we see something that will protect us we will have the boldness to keep having sex.

2; i want to add to what number 5 said. some girls are too flirty, I think it is because their genital was not cut when they were young, if they now tell her there is something they can use that will prevent them from getting pregnant, they will take it. Their only fear is pregnancy.

3: what we do in the Islamic religion is that before a man marries your child he will go for test to know if they can get married or not. That way they will know if he has anything. I have a friend and she is like a dog [how, let her talk] there is nobody she cannot have sex with, there was a time she got pregnant and she used to tell me everything. I used to tell her that she can get infected from anywhere, she later got infected to the point that she ended up in the hospital.

m: what happened to her?

3: I know they said it was cancer

m: what type of cancer?

3: I don’t know, she did not tell me. She hides everything even from her parents. There was a day I caught her swallowing some pills but till now she did not show me the drug

m: so you don’t know the cancer she has?

3: I just know it is cancer. She used those drugs and I asked her what the pills were for but she said it was not my business. At a time she got pregnant and aborted the pregnancy, they were asking her what happened and you know I will not want to expose her so they took her to the hospital and treated her but she has not recovered till now. There are times she will add weight, there are times she will be very lean. It was when they took her for test that they discovered that it was cancer. They asked her for those that she had sex with but she did not say the truth, they ended up taking her to a traditional healer [was she healed there?] she is there till now

m: number 5, will you take the vaccine?

5: yes [why] because you will not tell us to take the vaccine if it is dangerous

m: who among us here will not take the vaccine? You will tell us your reason

2: I won’t get it because you have said the infection is sexually transmitted and I don’t sleep around, so I know I can never contact the infection

3: since I know the vaccine is to prevent an infection from sex and you can prevent it by keeping your body, all I have to do is to stay away from sex so as not to get the infection.

4: as for me, I can take the vaccine since they said what it will prevent will not show now, it is in the future that one will have the disease. You said 40years. You don’t know the type of man you will marry; husbands hide things from their wives. some parents already know the infection their child has but some people they will just have to go for a test, and they will tell them to do one test they will do another test. I believe that some people will not even do the test at all.

m: number 6 can you remember what you wanted to say now? number 9

9: I can take the vaccine because the prevention is not just for now. Some children that sleep around can get any infection but such a vaccine will protect her.

7: for me I cannot take the vaccine because I don’t sleep around. There are some guys that when having sex with them, they will discharge bad sperm into the girl's body and it will affect the girl. Some already know the infection they have

1: yes i will, because some men will go out to have sex anyhow then come back to infect the wife. And some men use their wives to make money and she can get infected from there.

m: but you know the vaccine is for those that are not yet sexually active, you will get vaccinated before you start having sex at all. do we think that is a good idea? [yes] how can we ensure that adolescents like you get the vaccine if they include it in the routine schedule of immunization?

4; they should make it compulsory. When they brought meningitis nobody wanted to get vaccinated even with all the awareness. That day my friend and I was just... we got the vaccine in oje market that day, even our third friend refused it. It was after then they brought it to school. I agreed to take the vaccine that day because they already took it to my brother's school and I already heard about it and I was thinking that they may not bring it to our school. So if they should make the HPV vaccine a compulsory one, people will get it.

7: there should be something to know that someone has already being vaccinated, maybe a card.

m: I want you to tell me the programs or other things that can be done to make adolescents get vaccinated?

3: if they want to do it in schools, there should be things like biscuit and sweets there, when children see that they will be attracted to it. Then they like music, they should inform the principal, if they are playing music everybody will go there. Even those that were not called will go there. They will now be using age to screen them, once you tell them there is a gift they will rush there. They would have gotten there before those that want to give them the vaccine.

m: number 8 I have not heard you voice, you will dance for us now

4: I think the government should do a public enlightenment. they believe that if the vaccine is not a good thing, they will not be carrying it around, showing it on tv, on radio and posting posters around. There are some people that will still not take the vaccine if its brought into the school because their parents already told them that they must not participate in a social thing in school. And there are some other ones that once they see that one of their friends has been given something, even if it’s just an ordinary sachet of pure water, the fact that it is free they will also run there. They will not ask for what the friend did before she was given the pure water, they will just run there. It is when they get there that they will tell them that it is a vaccine but they will not mind as long as you are giving them the free water.

1: I don’t have anything to say

m: you should have something to say, what can be done to ensure that you get vaccinated?

1: they should announce it everywhere that this HPV vaccine is important for everybody and if my parents give me the go ahead to get the vaccine I will do it

9: what I want to add is that, when mothers give birth they should tell that at a particular age they will give the child the HPV vaccine, so they should plan to bring the child

m: for a child that is 10years?

9: they will tell the parents ahead, give them the venue and the date that the children will be vaccinated, they will allow the children to come.

6: is the HPV vaccine only for females?

m: no, it is not for females alone. We have mentioned age, so I want you to explain what we can do to ensure people of that age range gets vaccinated. Number 7, what can be done to ensure you get the HPV vaccine? Where should it be done? On the road or in your sitting room

7: if I see that people are getting the vaccine and they are fine that is it does not have any negative effect on them then I can also take the vaccine.

m; number 3, you were saying something to your friend

2: I want to add to what number 4 said, public enlightenment is a good idea because if it is advertised on tv and radio and show how people get vaccinated with no side effects, for sure people will get it, I will get it then so I can also get the benefits

5: yes i can get the vaccine

m: what should we do to ensure you get vaccinated?

5: ((inaudible)) if they put it in a private place I will get the vaccine

m: you mean where people will not know?

5: yes

m: does anybody have something to add pertaining to the things we discussed

2: what could have been happened to the girl I mentioned that was bleeding in the school or the one I heard in Labeorun, the woman gave birth and wanted to use the child for ritual so she said before she gave birth she was bleeding till her entire house got stained in blood. And you said the disease affects 40years and above. What could have happened to that woman?

m: until you do a test, I can’t say what happened to the person but you ask your questions after this session

9: why are they not giving those that are above 10years?

m: another question/ I am the one learning from you, it is a discussion. I will answer your question but for this discussion, does anyone have something to add? ...if there is none, that will be the end of our discussion. Let us clap for ourselves. Thank you for your time, we appreciate you.
