# Supplementary material for: “She must have been sleeping around”…: Contextual interpretations of cervical cancer and views regarding HPV vaccination for adolescents in selected communities in Ibadan, Nigeria
Source: PLoS One. 2018 Sep 17;13(9):e0203950. doi: 10.1371/journal.pone.0203950 (PMC6141096; doi:10.1371/journal.pone.0203950)
Supplement: S1 CaCx data — (ZIP) [file pone.0203950.s002.zip › FGD_RELIGIOUS LEADERS_PENTECOSTAL.docx]

**Interview group: Pentecostal Christian religious leaders**

M: good afternoon sir, my name is …………….. and my colleague is ……………. As I have said earlier, we want to explore your knowledge about cervical cancer, HPV and HPV vaccine. What you have heard, where you heard it, even if you have not heard it before, telling us that is also an answer. Do we all understand? As I mentioned earlier, we will not be using our names and I assure that whatever you tell us will only be used for the purpose of this research. do I have your permission to ask my questions? [yes] he is not the only one that will agree [he is our chairman] okay. ((greetings from a respondent that just joined)) and do I have your permission to record? [yes]. Welcome sir, before you came I explained that we are carrying out a research on cervical cancer, we want to know what you as a religious leader know about it, if you have heard it before and where you heard it, the causes and the means of preventing it. then what the bible says about this disease, the community members have told us their own view but we want to know what the religious leaders know. And I mentioned that we will not use our names and there will be no chorus answer, anyone that wants to respond will say his number before commenting. Can I continue?

Participant: please where are you from?

M: from …..

Participant: is it government or what? [1: ….]

M: my first question is that has anyone of us heard about cervical cancer?

2: please before you continue, I will want you to explain to us. when we say cervical [cancer], what part of the body is that? For example I know it is here on the arm, I know this is cervical vertebral, right? [okay sir] Cervical cerebellum and so on and so forth. So because of others that are here, explain it so that we can answer you well.

M: thank you sir. I didn’t want to jump to explain it so it will not be that I will call it a name and you will have another name for it. cancer is what we know as ‘jejere’ cervical cancer is when the cancer is at the entrance of the womb which is called cervix. To explain it, it occurs in women, if we see a woman that is 40years and above , that is bleeding form the vagina, with foul smell, back pain, weight loss and then leads to death in some, that is what we call cervical cancer. So if I may ask that question again, has anybody seen such before or heard about it?

7: I have heard it before

M: where did you hear it?

7: it happened to someone and they had to take her to uch for operation, they did it the first time, she was a bit better then they had to go and do it the second time before she became well

M: thank you sir, you want to add something?

5: Yes, I have also heard about cervical cancer. Someone was sick and was referred to the hospital. It was when she did test that she was told that she was cervical cancer and I know that they said it is cancer then

M: has any other person heard about it?

8: I have also heard about it, it happened to someone close to me. We took her to uch, they took care of her but she didn’t get healed. We thank God, it was this pastor here God used for us. At the hospital they said the cancer had ruptured her womb, she was about 90years then but with God’s grace, when we got back to the hospital, they said it had disappeared. it happened to someone close to me

M: is there anyone of us that heard it on the television or heard it in the church? Nobody? Okay so what can we say cause this cervical cancer?

7: there are two ways to it. some are caused by satan, because every disease in this world is from him but what I have noticed is that- there was a woman that I heard it happened to then, her husband is not in the country. They told her to be having sex but since her husband is not around, she cannot go and commit sin. Later she came down with cervical cancer and died from it. so it may be as result of not having sex. I just think so but the important thing is that it is from satan, particularly because the once someone has the disease it is straight to death. It is only the miracle of God that can save someone from that disease.

M: does any other person has something to add on the causes of cervical cancer?

1: thank you. In my sojourn with the word of God, disease is one of the things that sin brought into the world. Before man sinned, there was no disease but the moment man sinned different diseases came upon him. Till this present day, the diseases we have now is from sin. so if anyone wants to prevent such diseases, he should stay away from sin. If you stay away from sin, you will not have any of these diseases. And what I believe is that before you can be free from sin, you must accept Jesus Christ as your lord and saviour because he is the one that can free us from sin and diseases. God said in his word that he will not visit his children with the sickness of the Egyptians. The Egyptians are those in the world that have not accepted Christ, any disease can plague them but God’s covenant with His children is that they will not have such disease and he can even heal you if you have the disease. That is my response with biblical findings.

M: does anybody have something to add? We have heard from two people, does anybody have something to add? [no. let us make it fast, we have a meeting] okay so he has mentioned a way to prevent this disease, are there other ways that we can prevent cervical cancer?

8: the bible tells us that love is the greatest of all commandments, so the only way is to stay away from sin.

7: another thing I think can cause it is using drugs without prescription; all these drugs they sell in the neighborhood can cause such a disease. Or by sleeping around, if a woman has sex anyhow with different men, it can cause cancer. If you sleep with 5men that is 5 different diseases, 8 men means 8 different diseases. So if a woman sleeps around she can have cervical cancer.

M: thank you sir. Has anybody heard about HPV? Your no is useful

Participant: no I have not heard it before

M: so HPV is transmitted during sexual intercourse and that is the organism that causes cervical cancer. One could have contacted the virus at about 10years, for some it will cause the disease at older age but for others it will get cleaned out on its own. It will be in the body and when it is about 40years, it will cause cervical cancer. So cervical cancer has been traced to HPV which is transmitted during sexual intercourse. Also there is now a vaccine HPV that is available for children that are 10years and above and have not initiated sex. Do you think it is a good idea?

4: I don’t have anything to say yet, let us continue

6: I think it is a good idea because it will even help the church to prevent diseases from the devil

7: we that are Christians, we fortify ourselves with vaccine of prayer because you don’t know when trouble will come, it is the prayer you have already said that will protect you. In the olden days, the elders did not know Christ then, they had things that they eat to protect themselves incase of war. It is like building a house with stones, if anything falls against it, it will stand. So that vaccine is a good idea. People should take it because you never know what people have done. You can marry a wife that will have such virus, you can marry a man that already has the virus but if you have taken the vaccine, it will protect you. It is good

2; what age did they say a child should get that vaccine?

M: 10years. For now 10 to 13years that have not initiated sexual intercourse

2: what of someone that is about 18years that has not initiated sex?

M; yes she can take the vaccine. But someone that has initiated sex, it is a test she will go for and the test will determine if it is treatment she will go for or the vaccine. Do we have fears or concerns about this vaccine? ((no response)) just to give you more information on the vaccine, a child will take two doses of the vaccine and each dose is 7000naira making 14000naira and it is available in hospitals like uch. To ask a personal question, will you allow your child to take the vaccine?

3: yes if the money is available

4: yes I can if there is money

5: what I have to say is that people are saying the government is using such vaccines to reduce the population because the population is too much for our economy. Infact I heard that one of such means is the family planning government introduced and that the government does not want them to have more than 2 children. The government is saying those that give birth to more than 2 children don’t take good care of them and that is why they are introducing all these things. That was why I kept quiet since

5: yes I can since it is something that will make them healthy.

7: prayers are enough for my own children. For me I cannot allow my children take the vaccine. I fortify them with prayers

M: the other side I want to go to is that what does the bible say about this vaccine? Are there scriptures that you are holding on to for not taking the vaccine?

1: on the issue of health, the way God has given us is by accepting Christ because the bible tells us that by his stripes we are healed and salvation has been brought to us by his stripes. Healing has been brought to Christians by the beating of jesus Christ and even if we come down with any disease, I believe we can be made well because he had been beating for us. I believe in first praying about any disease before using any drug. I believe in the name of jesus and his blood for healing beyond anything that we can see. Most of the diseases are from the works of men ((phone rings)) and if someone has a demonic spirit that brings about diseases, even if you use a thousand drugs, the disease will still be there. It is until the demon leaves his body that he can be healed, it can even be by drinking water and he will be healed. That is my belief and that is why I do not believe in using that vaccine to prevent cervical cancer. But we cannot stand against what the government is doing because it is not everybody that believes in the name of Jesus or in his blood. And someone that does not believe will have to follow the one the government has prescribed

7: you asked for what the bible says about it. I want to mention two things and he has said one of it. Isaiah 53:13 says your children shall be peaceful and you will be far from oppression, that goes down to verse 17, it says our children will be peaceful and they will not be sick and have to go touch or any hospital. An incident happened that God worked out a miracle. A white boy was sick, he had cancer of the stomach, and you know white men don’t lie, they told his parents that he would soon die. Then his mother opened Isaiah 53 that he quoted the other time before him to keep reading. He kept reading but he was wounded for our transgressions, he was bruised for our iniquities, the chastisement of our peace was upon him and by his stripes we are healed. Then the boy asked his mother if that was written for some particular people and the mother went on to explain that it is for everyone that accepts Christ and that it will come to pass for anyone that believes. The boy then stood up and said he was going home, the boy got home and started playing ball. After one month that they thought the boy will die, he was still alive and strong. So they went back to the hospital for test and they found out all the cancer had disappeared. so having faith works. Isaiah 53 and Isaiah 54 all works. Then he said something, that not everybody believes and we as religious leaders are not against what the government is doing about health. People should do whatever the government tell them to do for their health. We are not against the vaccine but we just said the things we believe in

M: does any other person have something to add on a final note

3: they have both said it exactly the way it is. The other thing I want to mention is that scientifically virus itself- why is that you scientists have not found something. Is there any cure for any viral disease? No, till now nothing, you only prevent. I have tried to talk to about 3 virologists to explain to me why viral disease does not have any cure. Please how far have you gone? We have many viral diseases, even ordinary cold comes with no cure, help me explain that. So let people just put their hope in God, you have said that this vaccine may prevent but there is no cure. So let me know, how far have you gone to provide a cure to viral disease?

M: those that work in that line will be able to give you a clearer picture. How far they have gone will mean giving you figures and I should not give you wrong information. But what we are discussing right now has to do with the vaccine, so does any other person have a comment on my last question on what the bible says about the vaccine?

Participant: I want to comment on what he talked about, the way you answered is right but he was also right. We know that prevention is good but since you are in that line you should not one or two things about the outcome in that field, you should know what they have found out already

M: okay sir. It’s because you joined us late, we are here to carry out an investigation to explore your knowledge and not to give a talk

Participant: but you should still answer him and let us know [she has told you]

M: okay we can continue that after this discussion but we are recording now and that is outside our topic. So back to my question, will you as a person allow your adolescent take this vaccine?

5: why is it that it is from 10years? [they have said it]

M: you also want to ask a question? Because of our time- [you have used up our meeting time]

2: they have said the reason for that but then in conclusion we have seen that some people have said they can take it and some have said since it is not free of charge but if the money is available they can take it

M: thank you sir. We appreciate you for the time you have given us.
