# Supplementary material for: “She must have been sleeping around”…: Contextual interpretations of cervical cancer and views regarding HPV vaccination for adolescents in selected communities in Ibadan, Nigeria
Source: PLoS One. 2018 Sep 17;13(9):e0203950. doi: 10.1371/journal.pone.0203950 (PMC6141096; doi:10.1371/journal.pone.0203950)
Supplement: S1 CaCx data — (ZIP) [file pone.0203950.s002.zip › FGD_MALE TEACHERS.docx]

**Interview group: male teachers**

M: I hope you can all see this? This is to record our voices, ……… cannot take everything down so that we can have something to fall back to. Are we permitted to record? [yes continue] So to start, have you heard anything about cervical cancer?

1: I have never heard it

3: I have heard about cervical cancer

M: what do you know about it?

3: I just know it affects the ladies, maybe adults or the younger ones and there are a lot of awareness that people are trying to create about it. I have heard about the vaccine, they will do test and after the test maybe they will now give the vaccine or whatever.

M: okay, you did not hear about the causes?

3: no

M: any other person?

2: I have heard about it but I’ve not seriously studied it to know more about it. But from my knowledge, cervical cancer is a cancer and when we are talking of cancer it is a malignant growth and since we said cervical cancer, it means cancer in the private part of a woman but one thing I am not sure of is if it is the same with prostrate cancer. But from my knowledge when we say cancer, they said, not that I have studied it, they said when you are eating a lot of meat it can cause cancer. They said taking all these can something, when you put water inside the nylon and you put it in the freezer, I have heard a lot about all those things but I did not know there is a vaccine for anything called cancer. I have never heard that. All I know is that once you have a cancer like this, you have gotten your death warrant. So I pray that even my enemy will not get cancer ((laughs))[all:amen]

M: where did you hear all these things?

2: At times I browse, and I jam it. [jam it on the way?] You know you can see things when you are browsing but since I don’t have much interest in it, I just face what I am trying to look for. I am less bothered about this cancer maybe on facebook or whatever, since I don’t have much interest I will just leave it aside but at least I know about it but since you are now talking about it maybe I will go and sit down now and learn more about it. I will study or browse to know more about cervical cancer.

M: Thank you sir. What of number 3, where did you hear the little you mentioned about cervical cancer?

3: I heard it from our church

M: was it during…pastor was preaching or they organized a-

3: the women normally have this meeting so during their anniversary they said they are going to do cervical talk and free test and then give vaccine, I don’t know whether they are going to pay or not. I was planning to take my daughter but because she would be in school at that time. I know I will still do it because I have been hearing about it that people been doing it around just to be on the safer side.

M: Does any other person have something to add? What you have heard about cervical cancer

4: I heard that it affects ladies in particular when they have many sexual partners, something like that and maybe someone gets involved in early sexual intercourse. I heard something like that. And I got that from the net. [you were browsing] That time, I think….

M: did you intentionally go online to get the information?

4: I did not, that day maybe they were talking about something from that one I now begin to check it. I wanted to know the different types of cancer. I saw that we have many cancer.

M; okay so let me describe cervical cancer, as someone have mentioned, it affects women and it is cancer of the cervix, just at the entrance of the womb, that is cervical cancer. If we see a woman bleeding and she is not on her period, it is producing a foul smelling odour. Most times we see it in women that are 40years and above, if we see a woman of that age bleeding with a foul smell. Has anyone of us seen such case before?

2: I have heard it, someone testified in my church about that thing [that something like that happened] yes. She said she had been to uch later she was directed to some- [what is the number of the person talking?] number 2. She was directed to ABU from there she was put through series of tests. Then she came to church and testified…. ((inaudible))

M: did she talk about what caused it?

2: No, you know it is testimony [okay]

M: has anybody heard about such in the neighbourhood? ((silence)) So we have not heard about it before?

7: this is my first time

M: of hearing about that kind of thing? Okay. Number 1

1: I heard it in church just like my brother said, somebody also testified that maybe it is spiritual attack or something like that. She slept then discovered that something attacked her and she started bleeding from the vagina and it was giving that foul odor. She went to uch, after a lot of series of teststhat even from there they said it can never be healed, so she came to church. Later did this marathon fasting and then she got healed. I heard it in the church when she testified. [please speak up] I heard it in the church when someone testified that she had something like that and it was attack through the dream. asides church, have we heard anything of such in our community, maybe in the neighborhood. Maybe we heard that a woman was bleeding till she died, maybe with a foul smelling odor till she died. Has anyone heard that before?

All: no

M: for those that have heard it before, do we know what causes it? Maybe in our neighborhood, what do they say about it? Someone has mentioned spiritual attack, what are the other things that can cause it?

4: ((inaudible)) if a young girl is engaging in sexual intercourse, I heard she can have such. That is what I know about it.

M: so we have mentioned spiritual attack, number 4 has also told us about sexual activity. Any other person?

1: it is true that… our mothers when we were young used to tell us that any man that loves sex too much at old stage will experience such that later she will be bleeding and the blood will be smelling. It is too much of sex, at old age

M: okay can we now mention the ways we think it can be prevented, we have described it, we have mentioned the causes. Do we know how it can be prevented?

((inaudible))

2: with what my colleague has just said now, particularly my brother number 4, he said that among the causes, if he is right, that indiscriminate sex can cause it. which means one should avoid such. Another person said, I think number 1 talked about spiritual attack. They said good things need prayer and from the testimony I heard from the woman in my church, I believe we need prayer, serious prayer [yes] because all these things that are happening in this generation now did not use to happen. Which means we need to be prayerful. Then I want to add another thing with what I just said now, in the olden days, all these cervical cancer and that was not rampant then like it is these days. I tried to look at it, I read it somewhere that when we eat too much of flesh it can cause cancer whether cervical or other ones, which means what we eat these days may cause cancer. All these- what do we call it? pizza [all laughs] all these things we are eating- I am not discrediting MR BIGGS, you understand what I am saying but all these junk foods can cause cervical or whatever cancer. By the time you eat these things, you will see some with too much salt, too much maggi and the seasoning they put inside it. You know it wasn’t like that in the olden days. I look at another thing apart from that, look at the environment, sorry to say, all these things we are inhaling- I read physics and I learnt something there. They said when we expose ourselves to much of these tv hat you are exposing yourself to xray. These things cause problems little by little when we expose ourselves to such. Look at this pepper we eat, our mothers in the olden days would use grinding stone, sorry to take you back to the bible, it says we are sand, if one eats sand it is okay for the body but when one eats little iron and you swallow it. You know by the time you blend pepper nowadays, you know little iron will be there, they will say the blender is blunt, what is that? It is iron that has dissolved into the pepper. We use aluminum pots, very soon we will say it has holes in it, where do the particles go to? Our stomach. All these things we are swallowing everyday, you know I said something the other time that when you pack your water inside nylon- I was at UCH in a professor’s office, so there I read the book on such. I looked at it and saw that when you pour water inside nylon and put it in the freezer, there are some reactions with that nylon. All these things can- [lead to cancer?] yes. The nylon we also use for amala, moinmoin, everything is nylon and I look at it that all these things cause problem for our bodies. Infact I told someone yesterday, I didn’t know you would come today, that maybe I would go to oje and buy this local pot. We are killing ourselves with all these things.

M: thank you sir. Number 5 we have not heard your voice.

5: through sexual education for young ladies

M: number 6 we have not heard you also, how do you think we can prevent cervical cancer? What are the ways you know through which people prevent cervical cancer in the community? Maybe there are somethings done in this community

6: I have not even heard that before

M: any other person. Number 7, we have not heard you

7; I already said it that this is my first time of hearing about that disease

M: okay, number 1

1: The only I can think of is that- the solution lies on the medical officers to enlighten women about the dangers and causes of this disease. So you need to maybe sensitize especially women or ladies that are very prone to that disease. They should be counselled and they should be told the consequence of the disease.

M: moving on, can we- okay sir you want to add something? [yes]

8: thank you very much, I don’t know how you can do it but it will do us a lot of good if you can educate all these public canteens especially the big ones, the ones called MR BIGGS , they should put in mind the medicinal value of food given to us and not the taste. They are always after the taste and the taste is killing, it causes more of these problems than curing it. so if they can place emphasis on the medicinal value of their food than the taste it will help us to prevent all these diseases

M: so you are saying now that food causes cervical cancer

8:I believe that we have- if diseases or sicknesses are natural we have things that can cure them naturally but all these things [like what sir] that are no longer allowing us to eat naturally. They do not allow us to eat naturally again

1: I also want to contribute on the use of drugs, chemical drugs. We should try to emphasise our local herbs. You see in the olden days our forefathers before the arrival of this modern medicine, our fathers had ways of curing all these diseases

M: can you tell us the things they use to cure a disease like this cervical cancer?

1: they can use herbs

M; which one in particular? [herbs] so there are herbs that can be used to cure cervical cancer?

Participants: yes yes

3: I believe that because at that time or in the olden days there was nothing like cervical cancer [maybe it had another name] because of their lifestyle and hygiene, no matter how we see it they had a lot of things they use to take care of themselves naturally like my mother will say don’t sit on the mortar…they had their own way of hygiene. There was nothing like cancer. In the olden days, in the 60s, we didn’t think about cancer, look at them after work they will go and rest but for us we work throughout the day and night. We eat junks, we don’t sleep properly. Our lifestyle is different from our parents lifestyle.

M: thank you for that sir. Moving on, have we heard about Human Papilloma virus?

((inaudible- chorus answer))

Participant: what is that? You can break it down for us

M: HPV is a virus that we contact during sexual intercourse and causes cervical cancer that we discussed

Participant: this one you just said I have not heard it but the cervical cancer I heard that it is when you have too many sexual partners. It is from sexual intercourse

M; so there is nobody that has heard about HPV?

All: no

M: as I have said it is the virus we contact during sexual intercourse that causes cervical cancer. It is during sexual intercourse that the virus is passed from one person to the other and then cause the disease later. So the disease is the cervical cancer. So nobody has heard about that virus?

All: no

M: if I now mention HPV vaccine, has anybody heard that before?

1: let me ask one question, you know you said this virus is passed from one person to the other, it means the virus can be in the body of man.

M: yes sir

8: I want to disagree with that, where did it start from? Where did it come from? You said it is from one human being to the other, where did the first person contact it from?

M: There is always a primary- that is not our own discussion for now. What we are doing right is to know what you know about it. for the purpose of this discussion, have you heard it before?

All: no

M: what of HPV vaccine, has anybody heard that before?

8: we know virus but we don’t know vaccine.

4: I said I have never heard it before, this is the first time I will hear it

M: this is the first time you would hear it?

4: yes both the virus and the vaccine

M: do we all know what vaccine is?

All: yes

M: if we now say- we have said what HPV is, if we now say there is an injection that people will get so that they will not contact the virus, that is what I mean. [okay] It is like my grammar is too much ((laughter)) do we now understand? [yes, those are the terms you use in your line] so if we now say there is a vaccine that people can use that will prevent them from contacting HPV, do we think it is necessary?

All: yes why not

M: one after the other please

8: it has been established that this thing is in humans so we should look for a way to combat it. so we need the vaccine

3: we should go ahead because the effect of the virus is disastrous, it should be for every individual in the society

1: it should be compulsory, we need it. the problem has been identified so we should profer solution and in as much as vaccine can be used to prevent it, why not

2: there is nobody that wants to die, why will we say it is not necessary? even the woman I told you about the other time, when the problem started, when she was told that she has cervical cancer in uch and she came back home she saw one man- let me say they called one man, an herbalist. He charged the woman around 80thousand. The woman deposited 50thousand, she gave ((murmurings)) [you mean the herbalist?] yes the herbalist, he charged her 80thousand and she deposited 50thousand according to her testimony. She was given a drug to use before eating in the morning [what drug is that?] it is not a white man’s drug, it is a local herb. She said she discovered that the blood increased and she started loosing weight. She said she thought she would kill herself that way so she went back to uch after about a month. My point is that nobody wants to die but what happens is that genuine drugs- if this one you are talking about is genuine and people know it, people are ready to pay no matter the amount.

M: thank you sir

5: what is the correlation between the causes of this disease and gonorrhea or other STIs?

M: thank you for that question, but we will leave that question for now. What we are doing is a discussion and I am the one learning from you [no we are also learning from you]. If we now say we should be administering this vaccine to adolescents, children that are 10years to 19years. Do you think there are advantages in administering this vaccine to adolescents? Do you think we should even administer it to them?

8: we have said it the other time

3: it is necessary

8: that is just repetition

1: it is important especially if you consider the future

2: we said that this thing is for prevention, so by the time we administer it to them we are reducing the number of people that will have the disease

M: let me explain why I asked that question, you remember that number 4 told us that we contact this virus during sexual intercourse, don’t you think if we administer the vaccine to adolescents, it will make them sleep around

4: let us put the administering of vaccine on one side, sex education is very important. It should be given to them from time to time. So that one will teach them to abstain from sex

M: so number 4 is saying we should give them the vaccine with sex education. [yes]

3: he talked about it that whether we like it or not they are already having sex, so what can we do on our own part to save their generation. Also let them have the values, if you are not a virgin when you enter your husband’s house you will be returned. Let us have these values again; whether muslim or Christian, let us give them religious training . then sex education

M: before I take number 2, number 7 you were saying something, do you think there are disadvantages in administering this vaccine to adolescents?

7: we were talking about the solutions the other time, ordinary mosquito bite has prevention. There is net but anyone that is not interested will not use it. so to prevent this cancer, we need to administer this vaccine and most importantly to protect the others that are not sleeping around. Then sex education is very important

5: I hope you know more about the disease, do you think the vaccine is ((inaudible))

M: I will sanction number 5 ((laughs)) he is not answering my questions but asking me more

5: it is because I don’t know anything about the disease ((laughs))

2: I want to answer your question and what happened t number 5 is what I want to say because 80percent of the people in the community are like number 5, sorry my friend. Let me say 99.9percent. Before giving people this vaccine, there should be an awareness, we need to educate people because by the time you start giving people- I know what happens in the north even down here in the south, you want to give people vaccine for cancer they will be thinking it is to reduce their population, when God has said we should give birth to as many children as we want. Their own information will spread faster than your own if you are not careful. They will begin to tell people not to mind you that you want to kill their reproductive organ, they will say don’t mind the government, they are trying to reduce the population because of recession and before you know it you will just see that people are not coming out for the vaccine. So we need awareness even from what we have been discussing and the way number 5 has been asking questions, we need serious awareness on this thing, we need to educate people about this this thing so that government and the people sponsoring the program will not just be wasting their money. You know what happened with the mosquito net they shared, people were saying the government wants to know the number of their house so that they will be collecting tax, so they did not go out to collect it. something that will help us, the same thing we are discussing now. Then people don’t have much interest in white man’s drug, you will think we are in a computer generation, no we are just the same like our forefathers that did not want white ma’s drugs. See the woman I was talking about,, she went to uch but when she got back home they advised her to call herbalist. That is what is happening to people, they carry their uch card but immediately they diagnose them they go back to the traditional medicine. There are many like that they go to uch then come back home and spend more on this traditional medicine. It is because of the belief they have in this local- I will not call it useless something because I am also a black man- no measurement, no testing, no nafdac number and they are killing people. So we need serious awareness for this disease

M: thank you sir

8: I want to make sure I say this. Please don’t allow the idea that people may not support this to erase the idea of giving the vaccine to people. It can never be a license to abuse of sex, it can never be

M: why did you say that?

8: it is because to a level, we have been educated. The issue of vaccine is not just starting today, I can remember when we were in primary school our parents were told we must be immunized and they will take us there and we will take it and we have been seeing the results. And like my brother said, nobody wants to die, once you are told that this thing kills and somebody provides a solution somewhere, people will not hesitate before taking it

M; thank you for that, so let me go personal. Will you allow your adolescents to take it knowing that the vaccine is to protect the child from a sexually transmitted disease?

4: I will [why] because it is good to prevent the virus

1: I will allow my child or daughter to take it but before I will allow it I will advise them that the vaccine will prevent this disease but it is better you abstain from this act until you get a stage

8: thank you very much, I will support the idea of allowing adolescents take this vaccine. I will allow my adolescents to take it but what I will do is to tell her that it is not only cervical cancer that she can contact, there are other infections.

M: number 5 will you allow your adolescent take this vaccine, then we will take number 6

5: I can allow my adolescent to take it so far it is not a license to have sex around

6: I will allow it

2: I will allow it, infact there was a time some people came like this to my school for hepatitis, we as teachers were treated and I even carried my children to their office. Once the thing is good for our health- as I have said earlier, nobody wants to die, including my children, I don’t want them to die. Since we are told this thing is good I will carry them there myself ((number 8 interrupts)) [m: number 8 we will sanction you] so I will take them to replace myself. The only thing is that if the thing is happening in their school, they should inform me or tell my children to inform their parents to get permission first not that they will just grab my children and they will say they are giving them this cervical cancer vaccine. I must be informed before they administer it, that’s all

M: thank you sir. We all said we will allow our adolescents but that we will add something to it, is that because we have some fears or concerns about adolescents taking this vaccine?

8: it isn’t, we were adding- or I personally added that because of our Nigerian mentality. We will take one drug and believe it cures all sicknesses. So this one is just for cervical cancer there are still other STDs.

M: so we don’t have any fears or concerns as regards administering HPV vaccine to them?

8: once we are fully carried along, on that condition we are in support

2: then if I know the people, the reason I said I should be informed is that I want to know the people that will administer it because you know in Nigeria now people are looking for money. They may say they are from uch but it is a lie, they will just gather themselves and say they have NGO and begin to look for money, so I want to know the people that want to administer it [the source]. The source, that is it

M: so let’s say we want to make the HPV vaccine a routine vaccine in Nigeria, what do you think are the ways we should go about it? you all said we should have the vaccine, how do you think that the adolescents in this community for example get the vaccine?

1: first thing is that you go through the government, through the local government, the ward, the local government constitutes many wards, so when you see the local government chairman after taking permission from the state or federal level because the local government is the closest to the grassroot so when you go through the chairman of a local government, he will direct you to the ward and from the ward it will get to the people at the grassroot. And then people can be sensitized and the vaccine can be administered through that way

M: thank you, number 7

7: it is through government

M: ((participants laugh)) let me ask my question ((laughs)) is it that they should go to the government house to take it? [no] so how can we go about making sure adolescents get vaccinated?

7: as they have said before, it is through the government. Before we can do anything in this country it is through the government. We will inform the government then you will be free to do anything you want to do. Then …. People get permission through CAC, once that is there- I think that is the license to do whatever you have to do so-

2: I will also say through the government but when we say through government we have to define it. if the government has interest in this thing they can do it, for me when we talk about health or something like this, the government does not have much interest. Let me expatiate it, when they want to do election, they want to get into power, they want to enrich their purse they will say if your parents do not have voters card you will not enter school, you will not do this you will not do that, because that one concerns them. But when it has to do with health like the one we are talking about now, they can also do that, that before a child can enter school there should be a card for all these vaccines you are talking about. Its only because we are not sincere in Nigeria

M: so what should be done?

2: the way they do with that voters card, that children were not allowed to enter school, they should do the same thing

M: so you think there should be a card for these vaccines?

2: yes actually in Nigeria, this national ID card that they said we should do, you know that thing is just data base for us, all these things suppose to be there. Why are we having drivers license separately, this one separately? If I carry my national ID card to uch, they should know everything about my life, even concerning this vaccine you are talking about, whether I have done it or not, whether my children have done it or not, by the time they insert it the thing ought to be there. If the government can do that it will expose a lot of things. Even on the road, if I commit an offense once they press my number they should see- but because we are not sincere in Nigeria, all these things we are doing it is scattered. Government is not sincere

M: so where should the vaccine be administered? In the community? Someone said wards

5: we can employ the role of religious leaders before we can start such

1: we can go to the market places an as my brother has rightly said that there must be a database so that one person will not get it twice or thrice. Someone may get it at oje here then see it at beere, then get it again. Once you have done it, that should be all for the period of 6months or if it is a lifetime. So you need to let them know that- you know most of this people will not leave their workplace for anything because you know if they don’t go their market they will not eat. So it is better you carry this thing to their doorstep maybe their market place even some shops and once you have gotten this thing there should be a sign maybe a fingerprint so that there will be evidence that you have done it

M: thank you sir, number 8 you raised your hand

8: I will prefer the government or body in charge to use the style used by NPC, they go their houses to get the data and let everybody take it and mark the houses

2: I want to agree with number 5, I think using religious leaders is the best [why] number 8 we should move from one house to the other, even this mosquito net they shared, we did not receive it in my house because by the time they are moving round I would have been at my workplace so by the time they are moving around people will be in their market place. So the point of using religious leaders is that Nigerians are over religious, one way or the other on Friday or Sunday we will be in jumat or in our churches, whatever we are- some people may not even be in the market place on those days they will be in church, let me say 90% will be in church or mosque [nasfat]. That is why I say the best is take the vaccine to churches for Christian and mosques, that is the best. Because all these things- I am not saying will not be effective but this is the best. Infact people in Nigeria today, they believe their pastors and imams more than God, except we want to waste this thing by taking it to market and local government- how many times have I gone to the local government, when they are saying they are giving something in the local government, why will I go there when I don’t work there. My wife will say what am I going there to do, unless the government declares a public holiday- even if they declare public holiday people will use it to sleep at home they will not go to that place. But religious places, people will always go there, even if someone is dying he will get to church.

M: if we now talk about adolescents specifically, what can we do, where can we do it?

3: what I can think we can do is to take it to schools since the government is aware that this is what you want to introduce to them, you can go to schools

M: thank you sir, any other person?

5: government will have circulars that will notify religious leaders because if they are not involved they can campaign against the program [yes] they will tell people not to do it

2: yes they will quote quran to prove why they should not take it [yes] , if they are not carried along they will stand against it

M: thank you all for your time and all your points have been noted. I have learnt a lot from you, let us give ourselves a round of applause
