# Supplementary material for: “She must have been sleeping around”…: Contextual interpretations of cervical cancer and views regarding HPV vaccination for adolescents in selected communities in Ibadan, Nigeria
Source: PLoS One. 2018 Sep 17;13(9):e0203950. doi: 10.1371/journal.pone.0203950 (PMC6141096; doi:10.1371/journal.pone.0203950)
Supplement: S1 CaCx data — (ZIP) [file pone.0203950.s002.zip › FGD_MALE PARENTS_LESS THAN 45YEARS.docx]

**Interview group: Young male parents**

M: good afternoon sirs [good afternoon ma]. My name is ………… and my colleague is ………… and the third person is ………….. We want to ask you questions on what you know about cervical cancer, HPV and HPV vaccine. I assure that whatever you tell us will only be used for this research. do you I have your permission to go ahead with my questions [ask] . thank you

M: has anyone of us heard the word cancer before?

5: I do not know anything about cancer but I know I will get to it during the course of this discussion

1: I have heard it before

M: where did you hear it sir?

1: they say it on the radio, television that cancer killed someone but I have not personally seen someone that died from cancer or someone that had it but I hear of it

4: I have also not seen it before but I know there is a disease called cancer that everyone is shouting but I have never seen anyone that it happened to

M: where did you hear it?

4: as a person I am a bike man, I can go somewhere and they will be talking about it in an outreach. I hear of it very often

M: so some people said they have heard the word cancer before. Cancer is a disease and it affects different parts of the body. There is breast cancer, skin cancer but the one we want to discuss now is cervical cancer, that is cancer that affects the entrance of the cervix. Has anyone heard of cervical cancer before?

2: since I was born, I have never heard of cervical cancer

6: I did not even know there is something like that. I have not heard it before and God will not let anyone of us experience it [amen]

M: so I will describe it, maybe you saw it but did not know what it was called.

1: can we see an example?

M: we don’t have a picture but I will describe it. if we see a woman that is 40years and above and she is discharging blood from her private part and it is not that she is on her period. Some will have back pain and then lose weight and some will die from there. Has anyone seen anything like that before?

6: I have not heard that before, what I know is that a woman discharges blood when she is on her period

3: or a woman that aborts , she can also be passing out blood

2: I have not heard it before

M: so what we want to let you know is that there is a cancer that manifests like that and it is caused by human papilloma virus that can be contacted at a young age but manifests as cancer at old age. This HPV is contacted from sexual intercourse. Can you now see that we are also concerned?

5: you cannot say that, it does not concern us as we have said before, a woman can go and contact the disease from outside

M: but it will be from a man. You know you mentioned earlier that this issue does not concern you men

1: if a woman leaves the house, her husband will not follow her, does that mean she contacted it from her husband?

M: it may not be her husband but I mean that it concerns men. So as I was saying, cervical cancer occurs in women and they contact the hpv during sexual intercourse. There is what we call hpv vaccine. Do we all know what we call hpv vaccine?

1: just continue what you are saying

M: so there is a vaccine that can be given to people to prevent contacting this disease. Do you think it is a good idea, that is where we are going to. You know I have mentioned that the hpv is contacted during sexual intercourse, how do you think it can be prevented?

5: with what you have explained it is a good thing you have come to do, but then what can we do not to contact the disease? The disease can be in a woman and a man will contact it or the other way round. If my wife has it, I will not know so what can we do not to contact it?

M: you just rephrased my question, remember I said we came to ask you what you know? So who can answer his question?

6: how can one prevent this disease?

M: you are still asking me questions (laughs)

3: can one use condom to prevent it?

M: do you think condom can be used to prevent it?

3: that is why I am asking, except if there is a drug or an injection that can protect someone

M: okay, any other one. You know we have said hpv is sexually transmitted, so if I ask that how can one prevent contacting a sexually transmitted disease?

5: on what he has said, we can use condom but if there is something we can use so that we will not contact it, you should be the one to tell us.

M: I will still tell you, but you have not answered my question. Uncle how do you protect yourself from contacting a sexually transmitted disease?

5; I don’t know anything. I only sleep with my wife

M: so that is one, only sleep with your wife

5: yes I was the one that said it but what if she goes out and get this thing, how will I protect myself?

M: I will answer your question. Number 6, how can you protect yourself?

6: I don’t use condom and I don’t sleep with prostitute, I only have sex with my wife, maybe she now goes out, I do not know

7: what I can say is that, we don’t like saying the truth. We have heard that we can use condom to protect ourselves and we use it, that is the only way I know

3: it is the same thing number 5 has said, stick to your wife alone. We didn’t know all these diseases when we were young, it is these days that we have all sorts of disease like this cancer. The only way to protect yourself is to stay with your wife alone, don’t sleep around

2: our pastor used to tell us in church one thing, that its not all the time we should be having sex, you can just play with your wife without having sex. Its when you are always having sex that you can have such disease

1: this cervical cancer is not pimples, how will I know that my girlfriend has the disease, will I tell her to remove her skirt and let me check? That cannot happen. I work in the hospital, once they bring in a patient, the older ones can easily say what is wrong. It is something like fever you can see and know that it is fever. Except the lady is a good person and she choose to tell you and if he loves her, they will go for test and get medication. She will tell the man to give her time to use her medication

7: what I want to add is that, as long as I don’t sleep around, I cant contact the disease

5: does-

1: you have already talked

M: he still has something to add, please let him talk

5: how can one know that someone has it?

M: it is by going for test, as we said earlier it is not something you can see on the face.

2: you said a child can contact it by 10years but will not turn to disease till she is 40years, how will someone know?

M: I will give you the same response, test. Now we have discussed the virus and as I was explaining there is now a vaccine that one can get to prevent this disease before initiating sexual intercourse. Does anyone know about that vaccine?

1: the ‘ajesara’ I know is the local one (gbere)

M: that is something

3: I don’t know about that local one ((laughs)) I know there are vaccines for children but I didn’t know there are vaccines that concern adults. I know of the ones for children that are 5years to about 10 or 7years, that is the one I have heard before

M: so you didn’t know that there is a vaccine called HPV?

3: no

6: still on this thing you are discussing, when we were young, if the parents those days do not want someone to be sleeping around there are some things they will give her, they may put a ring on her finger that it is when she is ready that it will be removed. Some do it so as not to get pregnant and it is when she is ready for the pregnancy that they will remove it. some it is ‘gbere’ they do such that when she is now ready there are things they will do. Infact if you want to have sex with a woman that has all these diseases, you will know. Sometimes your body will not just respond, some may vomit once they climb such a woman. And these things for this kind of cancer, if you are already fortified like that and you want to have sex with a woman that has this cervical cancer, your body will not move and you may just start vomiting. That used to work those days but now that we are doing things like the white people, it may not work

M: you didn’t tell me all that before, that asides the vaccines that we are discussing, that there is a local vaccine

5: on what my friend has said, you know when one just starts a program he will not really be into it at the beginning, it is later that he will start responding. You know you may like someone and want to have sex with her even with what she has but you don’t want to contact the disease she has. With what my friend has said it is possible not to contact it. [m:how?] men of those days were well fortified, these days we don’t have anything in our body. You will see that an elder will put a ring on a boys toe but they will not tell him what it is meant for because he will remove it if he knows. And they know removing that ring will not let him enjoy his life. for some it will be ‘gbere’, for some it is ring and for some they will give them something to eat such that if he is close to contacting such disease, something will just happen that will prevent him from contacting it. all these is aside the white man’ s medication but these days we don’t take our traditional things serious. All we know now is to use condom

M: thank you sir, number 7, you want to add something?

7: no but you were saying something

M: okay I was saying he should explain the ‘ajesara’ he mentioned and they have done that. So you said you have never heard about HPV. This vaccine is for children that are 10years and above that have not initiated sexual intercourse [that are not yet matured (‘balaga’)] it is not that they are not yet matured, but they have not started having sex[ it is the same thing] okay thank you sir. It is available for males and females, that they should be protected before they have sex at all. My question is that do you think it is a good thing? Are there advantages in administering this vaccine

2: yes there is an advantage. The government has been shouting that we carry our children to the local government to get immunization so they will not be infected with some disease. It is a good thing that there is a vaccine like this one that will prevent them from contacting this cancer in the future. Now the chairman tells us there are vaccines for 5years and above, if you get card and go to Kola Daisy they will give you. So vaccine is a good thing

M: thank you. Let us not forget we are discussing HPV vaccine and not just vaccines in general. This one is for 10years and above to prevent them from contacting hpv

4: number 5 said something the other time on what our fathers used to do to prevent all kinds of diseases. But these days we don’t have any of those things they use so this type of vaccine is a good idea. The way we have sex these days is different from the fathers used to have sex and we don’t even know the other diseases that will still come up when our own children are older. So this vaccine is a good idea

6: if indeed we have a vaccine like that, it is a good thing. And the reason is that those days when a woman gives birth, our fathers will check the child’s destiny, they will see what the child will become and if they see that a child will face some difficulties in the future, there are some things they will start doing for the child to protect him and to make him rich. Some people do these things first day of birth. All these things I have mentioned are also vaccines, to make the child successful in the future. So if we have a vaccine for this cancer, it will also prevent hiv [m: this one is only for hpv] it can only prevent hpv?

M: yes

6: it will be good if they can get other diseases that it can prevent

4: why is it that it is only for children, it should be for us

M: to answer your question, you know I said it earlier that it is for those that have not initiated sex. For those that have started having sex, they will go for test and if he has the virus, he will be treated. But the vaccine is for prevention, the treatment is for someone that already has it so that he does not infect another person

7: but you know girls of these days are already exposed, some are not even matured yet and they are already sleeping with them and it is the early sex that will make her start maturing fast. I heard that there is an injection you can give to children that are having sex so that they will not get pregnant, please will there be no consequences when the child now grows up and wants to get pregnant?

M: that is called family planning

7: oh okay. But can a child like that take this vaccine?

M: she will go for test first to know if she has contacted it or not. So we have mentioned the advantages, what are the disadvantages that we think are in administering tis vaccine to adolescents?

5: we cannot know the disadvantages, it is you that want to give them that can say that

6: we are not doctors, the disadvantages will depend on what the vaccine is made of. For example see those ones that do rat killer, some will use carbide it will smell when the rat dies but there are some that will not smell. So it will depend on what is used in producing the vaccine

M: maybe you don’t understand what I mean by disadvantage, let me explain. What are the reasons why this vaccine should not be administered to adolescents? For example, we said the vaccine is for those that have not initiated sex, do we think it is possible that a child may start sleeping around after getting the vaccine?

3:on what you said, if you give a child- these children are exposed, if will be difficult to get three 15year old that has not started having sex so if you will do such for a 10year old you will not tell her what the vaccine is meant for, it will be between the parent and the person that will administer the vaccine, they will just tell the child to come for it. they can say the vaccine is for malaria or something. You cannot tell her what the vaccine is meant for. Even if you want to give her family planning drug you should not let her know. A parent that tells the child everything will just ruin the child’s life.

5: you know when we were immunized as children they did not tell us what it was meant for, we didn’t know what it will protect us from. If it is the government that wants to give us this vaccine and they expose what it is meant for, some people will not get it but if they just say it is for an infection people will allow their children take it. they should declare what exactly it is meant for

6: if they want to give this vaccine to adolescents, it is the parents they should educate, so it is the parent that will decide to let the child take it or not. But if you tell a child that it prevents one disease, they will believe it can prevent other diseases and turn promiscuous. So it is the parent that should be educated. For example, if there is something wrong with my wife and I want her to take a medication but I know she will not want to take it, I will not tell her what it is meant for, I may tell her that it is for her to sell well. I am just using that as an example. [not with injection] there are some women that don’t take tablets but will rather drink something so you will have to look for a way to make her use it

M: thank you sir. Speaking further on the vaccine, it is 7000nairaan each child will take two doses. For you as an individual, will you allow your child take the vaccine?

5: is the vaccine only for girls or also available for boys?

M; it is for both sexes

5: there is no one that will hear the benefits of something and say he does not want. The only hindrance may be the money because most people cannot afford it. we need to find a way around it, my child will be 11years next year but I cannot afford that, what can be done to ensure that she can take the vaccine?

M: you are the one that will give us the suggestions, that is my next question

6: I can allow my child take the vaccine since you have explained what the vaccine is for but it is too expensive with this economic situation. If they say it is 1000naira or 1500naira. Now I have 7 children, where will I have 7000naira in 7 places [14000naira], 14000naira in 7 places, if I want to build a house, that money will do something on the land. So that is reason I cannot take the vaccine for my children now.

2: if there is money I can allow my children take it, that is 14000naira in 4 places for someone that has 4 children. These vaccines used to be free those days, I didn’t know they collect money for vaccines. When you came the other day and those women were complaining, I thought it was just 500naira for each child. The economic situation in this country is terrible, we have been sitting all day with no work to do, the little money we make is what we use in sending these children to school. If I give my wife 7000naira to buy food in the house she will be blessing me. That money is too much

1:you know I argued with you before we started this program and I will go back to that argument, we that are here have things we are doing. We are gaining from the discussion but you are the one gaining from us more. Next time you want to do this thing find a time that we will be free. People like us that do not have children should also go and look for 7000naira

M: we only asked for your opinion sir. Maybe you do not understand my question

1: I understand your question, some people get where I am going already

M: okay because of our time, what do you think can be done that our adolescents get this vaccine?

3: there is no one that does not know what the government can do and even the government knows what they should do to make things easier for us. there is none of us that is not interested in this vaccine but the money is the issue. For someone that has 7 children and can only afford to pay for one child, he will not want to do it for only one of the children and that can cause a problem in the future. Then there are some benefits that we should be getting from the government, they should make this vaccine free or reduce the price

6: what I think the government should do is to reduce the price of the vaccine, it should be 1000naira [even 500naira] if it is not more than that everybody will go for it as long as they get the kind of explanation you have given us

2: my own suggestion is that- last year when they wanted to share mosquito net they were shouting on radio and tv and telling us where to get it so if the government can announce this one vaccine on the radio, the cost and all. Parents will strive to get it if they hear it on the radio if it is not too expensive. But that 7000naira is too much in this period that everybody is complaining. So let the government do awareness for this vaccine

1: is it the government that cannot give us free education that will reduce money for vaccine? My suggestion is that- none of our children will contact any disease, the government does not have anything to offer us, they are just gaining from us, we don’t have anything to gain from them. All these will not even get to their gate, those that have children already should take good care of my children, that is all I have to say

M: does anybody have something else to add? [Let us round up] If there is nothing else, we will stop there, thank you so much for your time
