# Supplementary material for: “She must have been sleeping around”…: Contextual interpretations of cervical cancer and views regarding HPV vaccination for adolescents in selected communities in Ibadan, Nigeria
Source: PLoS One. 2018 Sep 17;13(9):e0203950. doi: 10.1371/journal.pone.0203950 (PMC6141096; doi:10.1371/journal.pone.0203950)
Supplement: S1 CaCx data — (ZIP) [file pone.0203950.s002.zip › fgd_senior students_male_private.docx]

**Interview group: Senior male students private school**

M: good morning [good morning ma] my name is …………… and with me is ………. We want to ask you questions to explore your knowledge about cervical cancer, HPV and HPV vaccine. We want to know what you know, what happens in this community, what you have heard and where you heard it. Do you permit me to go ahead with the questions? [yes ma] and I have the permission to record [yes ma]

Has anybody heard about cervical cancer? Number 8 you are shaking your head, it is not video recording we are doing so my recorder cannot pick your head. Number 8?

8: no I have not heard it before

M: any other person?

4: I have not heard of it before

2: I want to say that I have not heard of cervical cancer but each time I hear about cancer I don’t really pay attention to it because it scares me so I don’t pay attention to it [okay]

M: why does it scare you since you don’t pay attention to it, so you don’t know what it is about. So what do you know about cancer?

2: I have met with someone who has cancer before and what I noticed was that it is a very terrible disease, her fingers were black, her tongue was…her feet was also black, likewise her face. It was something so touching for me so… and that made it a terrible case for me that I don’t even want to go close to anyone that has cancer

M: do you know the type of cancer the person had?

2: ehn it was cancer of…..

M: you cannot remember?

2: yes

M: as he has said, cancer is a disease. Who is there, can you hear me? I think you should come to this place. Come here. So as he has said, cancer is a disease that is now here in our community, has anybody heard of cancer before? Number 7 are you with me? You are looking at what they are doing. So there is cancer here. Have we at any time or the other heard about cancer? [yes] we have all heard of cancer, where did you hear about cancer?

1: from the hospital

6: hospital

M: okay . there are different types of cancer, you can have it on the skin, in the eye but the one we want to talk about is cervical cancer, that is at the entrance of the womb. Do we all know what womb is? [yes] so the tip of the womb is where cervical cancer occurs. Let me describe the symptoms, maybe you have heard or seen it before. It happens to women that are 40years and above, when you see a woman bleeding and it is not that she is on her period. You know when a woman is menstruating she discharges blood from her vagina but now she is not on her period but she is bleeding, she will be losing weight, some will have back pain and for some, it may lead to death. Have you seen any woman that something like that happened to?

4: no ma

M: any other person? ((quiet)) so what do you think can cause such? What do you think can make a woman bleed? I just want you to think through it before I tell you

3: if a woman aborts her pregnancy

M: okay any other person?

4: if she has stopped having sex that can happen

M: if she is no longer having sex. Does any other person have something to add? ((quiet)) you are not answering me, she is talking to only one person. So any other person?

All: no ma

M: cervical cancer is caused by a virus and the virus is transmitted during sexual intercourse and it is called human papilloma virus. Has anybody heard that before?

All: no

M: it is this virus that is transmitted during sex and then leads to cancer at about 40years. The disease only occurs in women, they can get the virus even as at 10years but it causes the disease later. So the virus would have been in the body till 40years when it causes the disease cervical cancer. And you said you have not heard of the virus before?

All: yes ma

M: so I have told you about the virus, it is the organism that will enter someone’s body, it is this virus that will cause the cervical cancer disease later in life. It is when one has this disease that she starts bleeding. If I now say human papilloma virus vaccine, has anybody heard that before?

All: no ma

M: you have not heard it. Do you all know what a vaccine is? ((quiet)) you know what ‘ajesara’ is?

2: a vaccine is… this is my understanding of a vaccine, it is sometimes an injection or drug to prevent against an illness that may happen

M: okay so before we even go into the vaccine, how do you think cervical cancer can be prevented?

5: people should be protected during sex

M: how can they be protected?

5: by using condom and the a woman and man that are not married should protect themselves from such virus

M: how will they protect themselves from such virus?

5: they should use condom

M: number 6 how can it be prevented? It is like I am speaking too much of English, we have mentioned the things that cause cervical cancer, we have discussed the virus and where it can be contacted, we said it is through sexual intercourse. Now how do you think one can prevent contacting the viruses, what will you advise someone to do to avoid contacting the disease?

1: by not having sex with one another

M: so sex should be cancelled, there should be nothing like sex? You are not talking

8: by keeping off sharp objects like blade

M: okay we should not use sharp objects. You want to say something. What is your number?

4: they say maggi causes it, so I think people should reduce how they eat it

M: so you think maggi causes cancer?

4: yes they usually say it

M: where did you hear that?

4: atleast they show it on television and I experienced it, someone has cancer because he was eating maggi, so they should reduce the intake of maggi

3: … …

M: it is not an exam why are you all looking so serious

2: you should be sure of your partner, when you have a partner you should know that this person is free of such virus. So when you know that your partner is free of such then you can go ahead with the relationship

1: someone that has cervical cancer should not use any sharp object

M: number 4 you want to say something?

4: I think if you are in a relationship, you should each know other’s blood group

7: maybe they should go for test before having sex, you should test if the person has any disease

M: so you should ensure your partner goes for test

M: okay any other person? Other ways we can prevent cervical cancer?

2: excuse me, is cervical cancer transmitted only through sex?

M: yes the virus can only be transmitted during sexual intercourse, and it is the virus that will now cause the cervical cancer

8: how about using sharp objects?

M: now you are asking me questions. You are supposed to be the ones giving me answers. I have told you how it can be transmitted, it is not by sharing sharp objects, it is through sexual intercourse that the virus is assed. A can pass it on to B, if a woman has the virus, she could have contacted it since 10years, when she is about 40years it can cause cervical cancer. Can we move on? [all:yes] so as I was saying, there is a vaccine that one can take before sexual initiation, you will take two doses and each dose is about 7000naira. And you will take it twice before sexual initiation, after which you can have sex if you choose. The vaccine is meant to prevent you from contacting HPV. We all know what a vaccine is?

All: yes

M: when we even say ‘ajesara’ in local terms, what does it mean? Number 5 what does it mean?

5: vaccine is an injection that is given to babies or it may be liquid

M: okay number 6, do you know what a vaccine is?

6: they can give children an injection so that the child can grow well

M: for the child to grow well and to prevent a disease. Number 8, do you know what a vaccine is?

8: they have said a lot

M: say it your own way

8: I don’t know

M: number 3?

3: a vaccine is to protect children

M: do you all know the vaccine they gave children for ‘yinrunyinrun’? [all: yes] you know they gave it to children that do not have the disease yet and not those that have the disease? [yes] now there is a vaccine like that for HPV. And remember we said it is contacted during sexual intercourse? [yes] so can someone that has never had sex contact the disease? [no] now they are saying those that have not initiated sex should get the vaccine, do you think it is a good idea? I am listening to you, number 4

4: we can call that prevention, so that they will not contact it

M: okay any other person? He has said it is for prevention, what are the other advantages that are in administering this vaccine to adolescents. Now they are giving children that are 10years to 12years that have not initiated sex. What do you think are the advantages in giving this group of people? Number 2

2: ….

M: if you think there are no advantages there, let us know

2: what an average person will think when you tell her to take the vaccine is that she may not even contact the virus so why should she take the vaccine, so she may feel it is a waste of money but as he said it is good for prevention but what I will advise is if the money is reduced, it can encourage people to take it. I don’t know if you get what I am saying.[explain so that I can get you] if it is at a minimal rate such that anybody can easily take it they will know that it is for their benefit and it is not too expensive. But if, it is expensive people will not go for it, that is when you will hear different ideologies…. How will I even put it [put it anyhow, say it in yoruba] they will say they cannot get it, you will be hearing different things about it. So I think it should be at a minimal rate

M: okay. Number 1 what are the advantages in administering this vaccine to adolescents?

1: I don’t know anything about it. My partner has said something, [what did he say] that …. I can’t say anything about it

M: okay. Number 4, you spoke the other time. What of number 3

3: ….

M: you can say it in Yoruba, will you be beaten?

2: excuse me ma, I have something to say, we should create educative measures. We should educate people on this vaccine so that it will not be like they are just bringing an idea like that, if they have deeper knowledge they will know that it is good

M: number 3 do you know what you want to say now?

3: no

M: help us beg him to talk

3: if those that administer this vaccine to children, if anything happen to a child that is 4years and 5years and they call them to give the child a vaccine, I believe nothing will happen to the child

M: okay. That is a general one, if they now say a child that is 10years should come for the vaccine to prevent HPV, what do you think is the advantage in that?

3: I don’t know the advantage

M: number 4 you raised your hand

4: one of the advantages is that it promotes healthy living in the community

M: number 7

7: … I cannot say anything about it

M: okay so what are the disadvantages that can be in administering this vaccine to adolescents? We have mentioned the advantages, what do you think can be the disadvantages? ((no response)) Maybe you think they are too young for such vaccine or that if they are given, they will see it as an opportunity to sleep around. Number 3 you shook your head, are you agreeing or disagreeing?

3: I don’t know

M: number 1 you raised your hand

1: what I can say about it is that if the vaccine is administered to children that are 10years they will believe that they are now old enough to have sex but if it is administered to those that are 15years and above they can still manage themselves.

M: so you think children of 10years are too young to be given this vaccine?

1: yes

4: what I want to say is that some are not- … they can just go to kid now and say they want to give her the injection and she is not old enough to receive that injection, it can affect her

M: so if I ask a personal question, number 5 now the vaccine is available why have you not taken the vaccine?

5: because I did not hear about it

M: now that you have heard about it, will you take it or not?

5: I can if I have the money

M: but you will not if you don’t have the money?

5: yes

M: number 3, why have you not taken the vaccine?

3: I think I … ((inaudible))

M: you said you did not hear about it before?

3: yes

M: now that you have heard about it, will you take the vaccine or not?

3: I can

M: why the can? We don’t have the vaccine here, so that you don’t think we are trying to convince you.

3: I will take the vaccine

M: why?

3: because it can protect me

M: what can prevent you from taking the vaccine

3: I don’t know what can prevent me

M: so it is sure, nothing can prevent you? We even brought it an you will take two doses, each dose is 7000naira [3: ah] ((all laughs)) but you said you will take the vaccine

3: I have not even finished paying for my WAEC

M: but you did not say that before, what can prevent you? Number 1 you raised your hand

1: I can take the vaccine and it is because of the things you have said, you have told us what it works for but what can prevent me from taking the vaccine is if it is too expensive. If it is something that is cheap now, we will take it but you know they cannot give us for free ((all laughs))

M: number 6

6; I will take the vaccine if there is money

M: so it is only money that can stop you?

All: yes

M: number 8

8: ((inaudible))

M: and you were making jest of someone, now we cannot hear you

8” I will take the vaccine

7: I will take the vaccine. If they reduce the price like 500 to 1000naira everybody will have the opportunity and will rush to get it but if it is like 3000naira not everyone can afford it

M: number 5 you are pressing phone [I have already talked], today is valentine, you are texting your babe ((all laughs)) so you have all said you can take the concern and the only concern you raised is money. Are there other concerns? Maybe your religion dos not allow such or you don’t like been injected, are there other concerns or fears as regards this vaccine. None of you mentioned parents, what will the community say about it, what will your neighbors say? You only mentioned money

1: they can think that one already has the disease. so when you are going for the vaccine they will think it is because you already have the disease so they will be telling people to avoid you ((laughs))

M: number 2 are there other fears? Maybe your religion does not support it

2: I think …. Let me gather my thoughts

M: okay keep gathering your thoughts, number 1

8: there is no religion that can say one should not get vaccinated because soon everybody will get it.

4: I will say you should let our parents know about it because this is a great opportunity [yes] because if they hear about it they will be moved and if the school let us- … the only concern is money

M: will your parents allow you get this vaccine?

4: yes

M: number 3 will your parents allow you take this vaccine?

3: yes

M: so if you go to them now they will give you that 14000naira?

3: ah ((laughs)) once it has to do with money, except it is free

M: any other person? Number 5, will your parents allow you?

5: I am not sure

M: why not?

5: because if they give me the money to take such vaccine they will think they have given me the license to have sex anyhow because I will feel I am already protected. So I don’t think they will give me the money

M: thank you. Number 6

6: I don’t have anything to say about it

M; it is a yes or no thing. Do you know your parents? [yes] will they allow you to take the vaccine? [yes] number 8? [yes] okay so if they now say this vaccine- you know I have said the vaccine is available, if we now say we should make it a routine vaccine like the children’s routine immunization, how can we ensure that adolescents take this vaccine? Number 3 what did you say?

3: I said there are several ways you can attract them to take the vaccine

M: tell me some of the ways

3: ….

M: say it anyhow. Is it by keeping quiet in front of them? We will gather them then keep quiet, will that work?

3: no

M: so what will work? Number 4 you raised your hand

4: some people are so stubborn that even if they are advised about their health they will still be insisting on what they have to do. Although those people we are talking about will not cooperate because they do not know anything about it

M: how are we going to make them cooperate?

4: we should advise them

M: who should advise them?

4: programs on television and radio, with time we will see people that will be cooperating little by little

M: okay.

7: there are some families that will tell their children to go and take this vaccine and they have the money but there are some children that do not like injection, they will refuse it

M: why do they not like it?

7: because it is too painful

M: okay any other thing?

2: they should make them realize the possible consequences of not taking it. If they know the possible outcomes they will take it. One other thing I will keep saying is about the money, the money is a way to encourage them to get the vaccine. Then what he was about to say is that people prefer drugs to injection, so the vaccine should be in different forms, so someone who like injection should take injection and those that want drug can have it

M: number 3 can you remember what you wanted to say? What can be done to make you take the vaccine?

3: you some people don’t like to take injections

M: so what can we do to ensure he takes it?

7: if they can get a drug that can make the person sleep off, they should give her the drug and when she sleeps off they will call the nurse to give her the vaccine

M: so they should drug her? ((All laughs))

4: I will say that is not good enough [why] because there are some people that will not allow such; even if they are sleeping it will be like poison. So it is not good enough. The person may not wake up again

M: thank you, so what should we do instead? You have mentioned earlier that we should say it on radio, if after doing all that some people still do not take it, how can we attract them?

4: it is their health; they will say they can do anything they want to do. Maybe they should be helping them in prayers

M: do you think the location of the centers where the vaccine is available can affect how people take it?

All: yes

2; in the sense that some people will not want to go as far as anywhere to get the vaccine or go for medical checkup, they just have that wrong mindset about medical issues. So if it is somewhere very close to their house, it will encourage them and they will go for it but if it is now one far place, the thought of going there can scare them off

M: thank you

7: actually if they say people should come for the vaccine at yemetu and maybe the person is living at monantan which is far from here. It will be hard for that person to come down to this place

M: so what should be done instead?

7: they should take it center by center, if they put it here, maybe they should distribute it round and share it area by area it will be easier for people

M: number 4 you raised your hand

4: I think if they prevent it for a person once it will attract others that these people are real, they will stop believing that they want to use them for something, because they believe that-

M: they believe what?

4: they believe that caring for their health- that they want to use them for another thing

S: so people have that believe now?

4: yes, those people that go round to give children vaccine, that is why some people do not allow them to give their children

M: thank you. Any other person? If there is nothing else that will be the end of our discussion. Thank you.
