# Supplementary material for: “She must have been sleeping around”…: Contextual interpretations of cervical cancer and views regarding HPV vaccination for adolescents in selected communities in Ibadan, Nigeria
Source: PLoS One. 2018 Sep 17;13(9):e0203950. doi: 10.1371/journal.pone.0203950 (PMC6141096; doi:10.1371/journal.pone.0203950)
Supplement: S1 CaCx data — (ZIP) [file pone.0203950.s002.zip › FGD_FEMALE PARENTS_LESS THAN 45YEARS.doc]

**Identifier code: FGD_FEMALE PARENTS_LESS THAN 45 YEARS**

M: good morning, my name is ………. and my colleague is …………... We will be asking you questions on cervical cancer, Human Papilloma virus and hpv vaccine. As I ask questions, you will mention your numbers before answering the question. Before I continue, do you permit me to go ahead with my questions? [all:yes] and I am permitted to record? [all:yes] thank you. I assure you that everything you tell us will only be used for the purpose of this research. Has anyone heard about cervical cancer?

5: this is the first time

6: I am hearing it for the first time

1: I have not heard it before

3: no

M: let me describe it, then I will ask that question again. Cervical cancer occurs in women that are 40years and above that is bleeding and it is not that she is one her period, the bleeding will be associated with foul smell, she will be losing weight, some will have back pain and can then lead to death. Has anyone seen that before?

5: and it is not that she is in labour?

M: no not labour

1; I have not seen it before

M: number 6 is still thinking about it

5; I have not seen such before

3: I have not seen it before

4: I have not heard it before

6: the one I know is that after she gave birth the blood did not stop, she was bleeding, that is the one I have seen before. But I have not seen a woman that is bleeding and it is not that she is on her period

M: okay let me explain further. Cervical cancer is a sexually transmitted disease and it is caused by HPV. It is the HPV that one contacts during sexual intercourse, you will pay for the one I am explaining ((laughs)) because you should be the one telling me all these. One can contact the disease at a young age but it is not until at an older age that it will develop into a disease. So it is possible to have that virus in the body but it is until 40years and above that the it can develop into cancer. Do you understand? You all said you have not heard it before. My next question is how can this disease be prevented?

5: based on what you said, it is because of this thing that there is condom. I don’t know of any other drug that can be used to prevent it. but I know the use of condom is important. Though some people feel that if they see condom with you they will say you are a prostitute so they do not use it. that is my own take

M: any other person? Do we understand what I explained? As women, how do you think it can be prevented? Number 1 wants to tell us something

1: it is the same thing she has said, it is by using condom. God should help us with these our men because they contact different things and bring it home to us their wives.

3: another way I think this can be prevented is to avoid sleeping around

6: what I can say about it is that, it is men that have that disease in their body [no] I mean a woman will contact it from a man.

3: what I want to ask is that is that virus in a woman’s body, maybe it has been there since she was a child

M: she contacts it from sexual intercourse and the virus can now develop into cervical cancer

6: okay what I can say is that if the man uses condom, it is not enough. If the woman doesn’t sleep around, it is not enough. Some people only have one husband and will still contact diseases from that partner. If someone that has never been disvirgined meets with someone that has it, she will contact it. so it is women that should use medication so that nobody can infect her. You say they should use condom, will they not have children? What will happen if they want to have children? So it is the woman that should know what to do. The woman can go for a test or check up before they have anything together so that she will not infect the man. There is none of us here that- do you want to tell me that it was only a man that was following you when you were in school. Some people before marrying- because it is character you will check before you marry someone, that is the way it is. So it is the woman that should know how she will check herself. But most of us now only know how to eat, visiting the hospital is hard for us. They will be thinking about the money for food. It’s we women that should know how to take care of ourselves.

M: do we all know what ‘jejere’ is?

3: ‘jejere’ is cancer

M: and have you heard that before?

All: yes

2: on the television

M: which type of cancer did you hear of?

2: the one I heard was breast cancer

3: I heard that cancer can happen on any part of the body, it can be in the eye or breast but I know it is breast cancer that is very popular.

M: where did you hear that?

3: ah ah I have seen it before, I am not a young girl so I have seen an old person who had cancer and that was what killed them but that was breast…

5: based on this thing we are talking about, I know someone that has cervical cancer

M: and you didn’t tell us before

5: they have taken her to uch and they had to trim the cervix but it still didn’t work. They had to remove her womb. What surprise me is that with everything that has been done she is still not okay. Everybody is praying for her. Till now, she cant toilet, she can’t eat what the whole world is eating. We just pray for her ((inaudible)) we have seen cancer of the eye, cancer of the mouth.

M: but the one we are talking about is cervical cancer

4: the one I know is the one that happen in men to their scrotum

M: I’m happy that we all know what cancer is. But the type of cancer we’re referring to ((welcome ma)) is cervical cancer. What does number 5 think caused the one you talked about?

5: sexual intercourse that is not protected and dirty. Yorubas say ((inaudible 13:26)) no matter what your husband does, don’t say what he does is what you will do. After respect yourself always. I’m still hammering on it, if you want to be a sex worker, ensure to go with condoms. If he insists he is not using, then let him go. But respect yourself first because whatever he wants to do with you, you’re not satisfied with it. Since you’ve tendered a codom and say he doesn’t want, then let him go. We shouldn’t meet again. Do you understand? [yes ma]. Let your friend see it in your purse and make jest at you. It shows how knowledgeable she is. Even if I have fifty condoms, it’s my business as I cannot have dirty sex ((inaudible 14:30)) it’s all about our kids. Education is the best and if I have the grace to get money, I’ll collect it to send my children to school. There is no inheritance a parent can’t give her children. My daddy has a house at magodo but was never educated. So a woman will do everything possible to ensure her children live well. I’m sorry to say, I’m not from Ibadan, but from Ede. Apologies to everyone from Ibadan. But if you marry a man from Ibadan, you made a big mistake. They have nothing to offer. Struggle to take care of your child.

M: ok, if I may ask, you know I called it cervical cancer, is there any other name it’s been called in this community? You know you didn’t know the name until I explained, are there other names?

5: cancer is the only name it has. In those days, if our parents want to talk about it, they don’t voice it all out. But I remember in 2005, my dad was sick, he had cardiac arrest. We took him to UCH, I was asked not to sit at the emergency section. There was this lady there, I know she must have been pretty but by the time she was moved out, she was green. I wondered to myself what type of sickness it was. But the woman who came with me told me it was cancer

M: so that was where you heard about cancer?

5: yes, that was how I got to know about it. But there was this brother in our area, when I knew him he was fine but now, you know tauts and the work they do. They said he has cancer. We didn’t use to hear about this disease before, this is what I know

M: thank you ma. Number 7 and 8. You're welcome ma. Have you heard about cervical cancer before?

7: no

8: I have, but not the cervix but the womb

M: you have not heard of the cervical cancer before

8: No. but I can't call that one cancer because ((inaudible 17:56)) when we get to the hospital, there is no name that can't be given to diseases, when tests are run, they say it’s cancer. There’s civilization now and it has wiped away traditional healing because if it was treated the traditional way, it won't be like this.

M: thanks for that. What are those things that one can do to prevent this cervical cancer. You said earlier that there is a traditional way.

8: eehn…you know you educated people [I’m not educated but on the same level with you] if you get closer to those who sell native herbs, I mean those that really learnt it, I know they have herbs that can cure it. I remember a relative who had it and at UCH she was told it was cancer, but we went to a woman that sells native herbs, she was given different herbs and roots that she would use to wash different parts and it was cured. At UCH we were told it was going to be cut off because she couldn’t sit down and we were asked to pay like two or three million Naira. But after taking the herbs, it was cured and she's presently in the UK. She goes to the toilet well now. What I know about cancer is that of the breast. I also have another relative like a big sister, she had cancer. Things were not like they are today, she was taken to UCH and her breast was cut off. Right now, she has just one breast to feed her children and wears foam in the other. God blessed her with six children with that one breast and she's alive and well.

M: thank you. We are not talking about cancer of the breast but cervical cancer. I have explained what happens to women who have it. It happens mostly in women from 40 years and above that bleeds without being on her period and is accompanied with a foul smell. They begin to lose weight and some die. Have we seen anyone like that before?

8: I heard about someone who just started bleeding and she wasn’t on her period. To my knowledge, I felt maybe stress or she thinks too much. Because the bleeding just started and it wasn’t diagnosed as cancer

M: did she do any test?

8: you know that it’s not something that was happening before, it just started all of a sudden and she was being and she was being taken care of but she still thought a lot this led to high B.P and then death.

M: where was she taken to?

8: Hospital

M: but you can't tell what exactly was wrong with her

8: no, I don’t

7: but what exactly is the cause of cervical cancer?

M: hmm… I am not here to teach, you are here to teach me. I am here to ask questions and you are to answer questions.

7: ok, but we don’t know what causes it

M: you don’t know what causes it, but I'm sure there are some people here who know what causes it. Who knows?

8: you see, this sister talked about protection the other time. You see some men today have different things in their body. [Do you want to answer her question?]. No, you asked the causes,[yes, ok] that is why I'm referring to what she said the other time. This disease can be contracted from men, the sperm from different men into a particular woman. The reason is that you don’t know how neat that man is. I remember when I was in secondary school, a man came from America, though he is not in Nigeria anymore. He slept with a woman and didn’t use a condom. By the time the sperm came out, the colour was like that of this cloth, orange. So when she cleaned it with a tissue and saw the colour, she kept it and later showed me. I just told her to go do a check up and take care of herself, because that is not the colour of sperm. Though the man helped her to travel out but before they left Nigeria, they used condom all through. She went to UCH then to see a particular doctor, who told her to do some tests. The tests showed that there was nothing wrong with her, which was why she insisted on using condom. We really have to be careful when it comes to intercourse as the sperm coming from men could cause so many things and it is in the woman that the disease shows. As for men, it is easy for them to know that there’s something wrong with them. But in a woman, it takes a longer time.

M: thank you ma. So, you also agree that it can be transmitted sexually. Does anyone have any other thing to say?

1: the daddy that left here now called us one day to gate and said they want to do a test for cervical cancer but the conditions for the test was that, anyone who had sexual intercourse with a man the previous night could not do the test [please don’t talk in parables as we are all women here]. Ok but whoever has not slept with a man for like 3-7 days could do the test. So many people left as they slept with a man the night before. When it was my turn for the test, an instrument was put in my private part. It was then I knew that there was cervical cancer, not just cancer of the breast. After the test, we were asked to write down our numbers as they will call us for the result. But after six months, I received a call from them saying I should come to a particular location to get my result. I later went there to collect it.

M: so that was when you heard about cancer? That was how long ago?

1: that was 2014

M: so before that time, you never heard about anything called cancer.

1: no

M: ok, so we have talked about the causes and prevention. You mentioned it that we should be using condoms. But are there other things we can do to prevent it?

5: I'm sorry to say, ((inaudible 26:58)) most of the time when I woman has sexual intercourse with a man and goes to the chemist and says she wants to buy prevention, hope you know prevention has side effects? [all: yes]. I saw someone who used prevention and at the end of the day, oh God…they use four of her mother’s wrappers to soak her blood. After she was treated and was ok, I discovered that anytime she bends to sweep, its painful straightening her back. She said that that she gets irritated and usually has stomach pain, all in the name of using prevention. Since then, she hasn’t been able to conceive. When she wants to urinate, she cries because it’s painful. When she returns from the toilet, after 20 minutes, she's still writhing in pain. She was later asked at the hospital the particular prevention drug she used. I think she said postinor. She stopped using it and she became better.

M: so what did they say was the name of the disease?

5: it was the drug that cause it not that it was a ailment

M: ok, its not that she contracted a disease?

5: no, it was the prevention drug

M: number 6, you were saying something in low tones to her, why not say it so we can all hear it?

6: not at all

M: maybe you know the name of the drug [no] ok, I thought you wanted to tell us something. Ok. Are you going ma? Thank you ma. Number 5, it seems you have seen a lot and don’t want to say it all. You just want to be saying it little by little. Ok, so we have been able to say what can be used to prevent it. Like the usage of condom, is that not true? [all: yes]

5: I want to ask a question. Are all diseases cancerous?

M: you see that question, the one we are focusing one is cervical cancer.

2: please I want to say something.

M: is it a question?

2: yes, this cervical cancer, can everybody contract and what causes it?

M: you are asking me a question. It is also part of the questions I'm going to ask you. I also came to get information from you.

8: they are asking us to get information about it from us.

M: Thank you number 8. We came to get information from you. We can ask those questions later but right now, I want to know what causes it in this community.

2: it’s something that will cause it

M: but one can run a test

2: if one does a test, is it the blood that comes out of the woman that can be used to detect she has it?

M: those are the symptom, which was why I said earlier if we had seen a woman who is about 40 years and above who bleeds…

2: yes, that’s why I asked if it’s only blood

M: yes, it’s the main symptom but that does not mean that the person does not have the disease yet in the body. Do we understand? It's not that I came to lecture us, you are to tell me how it happens. If I may ask, has anyone heard about the virus that causes this cervical cancer? Don’t nod your heads, the recorder can pick it

All: we never heard of it before

8: one can contract it from the toilet. The heat from the toilet can cause it. For those you who use potties should be careful. Don’t use a general potty because you don’t know who had used it before. That is how I feel one can contract it.

5: it's a good question. But you in cities let us say Ibadan generally. You know Ibadan is very dirty. Do you understand?

M: number 3, are you sleeping?

3: no

M: please stand, don’t sleep.

5: Ibadan is dirty, with the way we eat, in our journeys, in making friends with men. In this life, one has to really be careful. As we are ((inaudible 33:10)) God forgive me. Child bearing is important to God. It's not easy but we have to be watchful of what we wear, maybe…like I heard…I and someone were going somewhere, while we were still in the house, she took her pant and I asked her if that wasn’t the pant she wore the previous day. I said her why she wear the pant she wore the previous day. God forbid. Since your uncle isn’t coming home, I'll be sleeping naked. I can out on a gown but that I'll wear anything under isn’t possible. Maybe because I'm on the big side that is why I don’t fancy it, but by 8pm, I remove my pant

M: do you think such things can cause cervical cancer?

5: it can cause it, because if you put on underwear since yesterday and you meet a man, won't you have things to do with him? ((inaudible 34:19)) [p: please clap for number 5. They all clap and laugh] he can perceive an odour and…do you understand? [I do]. It can cause it

M: number 7 said something about heat.

7: eehn…the heat from the private part is much. We must endeavour to always clean up. How can someone wear a pant for 2 days? Even if you have 2 pants, you will put on one today and the other tomorrow. ((Inaudible 34:00)) is it not the type of work we do that made us contract it? It is easier for a woman to get it because of the kind of body we have

M: ok, thank you. Number 4 wants to talk. Ah! Ok, number 3. It seems you wanted to say something the other time?

3: what I feel may be the cause of the disease is that, you know there are some women who use cloth during our period. That type of cloth… it was cloth that was used in those days, if one wants to use cloth, one should use soaps with fragrance and dettol to wash it. When it's dried, one still needs to use the pressing iron to kill any germs on it. But if you just spread it somewhere and you also keep it somewhere and put it on later, it can cause such diseases.

M: so you think that one too can cause cervical cancer?

3: yes, it can cause it

M: ok number 5 or number 7. Ok, number 3

3: it is only cancer we are treating, there are different types of diseases, you said there is bacteria, you also say there's toilet disease, you also say…it is you educated people that tell us all these diseases which is what can be you treated. If you say someone has toilet disease, you know it appears usually on the private part. I never saw it before but the first time I saw it, it happened to my friend. Some things happened to me that made stay at her house. She later told me that her private part itches; she scratches it a lot, so much that it comes out. But the first day I would see it, I saw that it has made dots on her private part. But this cancer you are asking questions on is the only one you're concentrating on. What about others? When you get to the hospital, they will say it's toilet disease and by the time you scratch it, it causes the whitish discharge that comes out of the private part of a woman. What can't cause cancer?

M: you know toilet disease to can cause cancer

3: yes, there is toilet disease, by the time you scratch it too much, you'll discover that what God created to be inside, will come out. It happens. Twelve years ago, there was this old woman who was like my mother-in-law, when she wants to urinate, I discover that something whitish comes out of her like egg and she will get up and say nothing to anyone. But there was something wrong with her. I later knew what happened to that woman because of someone it happened too. That woman was very secretive, so I called her one day to ask her what was wrong because I saw some things when she urinated, she replied, thank God you are someone that knows me well. I can say you are my biological child because I was really close to her, she told me it was because she had given birth many times I wanted to hear more. So I ask someone who was a specialist in caring for the aged, I explained how close I was with the woman and that she had given birth many times and right now, I know it's her womb I can see now.

M: ok, thank you. So let’s go back to what we are really talking about. Thank you for sighting those different interesting examples. Your child is smiling at me

P: please ma, I believe we are rounding off

M: don’t worry we’ll soon round off. So the human papilloma virus that causes cervical cancer that I told us earlier that it is gotten from sexual intercourse and later causes cervical cancer and we said we have not heard about it before. But have we heard about human papilloma virus vaccine before?

6: I'm just hearing about it from you now for the first time

M: ok, I just added vaccine to it. Do we all know what vaccine is? Vaccine is like immunization. Has anyone of us now heard about the vaccine for cervical cancer?

3: not heard about it before

All: not heard about it before

M: ok, we have said there is a disease called cervical cancer, what it looks like and what causes it. If we say there is a particular vaccine that young men and women will get from age 10 who has not had sexual intercourse with any man, to prevent cervical cancer, do you think it's a good idea?

3: it makes sense

6: it's very okay

M: what advantages are there for young people from age 10 have in getting this?

5: that’s a good word. I'll talk based on what the Hausas. They marry away their children at a very tender age. At the end of the day, those children get pregnant and give birth. If it now gets to the age where they are supposed to get married properly, do you know they will have problems as they won't be able to control their urine? Other diseases also set it. That is why government should go into their schools to run a test. There is no child that we can predict that hasn’t had sexual intercourse. May we not cry over our children. Do you understand? If the government can bring it, it's accepted by me.

M: it is even available now. The vaccine is available

5: accepted. It's not only kids they we give vaccine to

3: the idea is okay, but help us do a very good research on it to know if it has side effects on the children in future. So it doesn’t damage anything in their body.

8: the reason why she said you should check for side effects is because children from age 11-12 years, you'll see that sperm has developed. You will hear now that a 12 year old boy impregnating a 13 year old girl. When we were younger and matured, did we even know what pregnancy was? [Forget it] I was about 15 years of age before I started menstruation. Though there were some who had started, but the very first day I started, my mum isn’t literate, so she told me to go inside. She then took one of her clothes attached a rope to it and asked me to use it. I told my friends and they said they have been doing it for long. They said they were going to follow me home. When we got home, they asked me to bring one of my mother’s clothes and she used blade to cur it into sizes. You know people were not exposed then, but now, they go to schools teaching and distributing always pads and condoms. And you see SS1-SS3 who now use it and are already matured. That is why you see a 12 year old boy impregnating a girl. That was why number 3 talked about side effects. Because the world we are now is different from the olden days

M: ok

7: how do we know those children who are not yet sexually active? There was this child of about 9 years old who was already sexually active. I also have a daughter and I checked her private part every morning for any changes. Because a child who hasn’t being disvirgined, should it be like this? You are our aunty now. She said that something whitish comes out of the girl’s private part because they check where she urinates and concluded that something must be wrong as this is not the way it should be. Do you understand? At least, that part of a lady who had not disvirgined should not be this wide. I told her that I can show her my daughter’s own because I checked it every morning and hers is small. She said she will interrogate the girl as hers was wide as the thumb can go into it and if she doesn’t talk, she will use hot iron on her. I was baffled because the girl was still under her parents and had an elder brother who watches over her when the mother wasn’t around. But anything could have happened between the two of them if they are not God-fearing. I told her I was going for a program and she needed to be patient but she said she was going to bring the army to arrest the father because she was the one who pays the house rent. Those kinds of children that are sexually active, how are we going to know to give them vaccine?

M: so you think that they should give them…

7: that they should run tests on them first

M: so there won't be… [uh hun]ok, but my own question is that, you have agreed that they should be given the vaccine

7: it is good but they should do more research to ensure there are no side effects in the future.

M: because of side effects. So that there are no side effects [yes]

5: ((inaudible 47:45-47:58)) do you understand? Let the government make this vaccine public.

M: that was why I said the vaccine is available. What I want to know is that, do you think there can be some disadvantages in giving small children. What disadvantages can be found there?

5: let them give them, if it is something that will sustain their lives

M: but I am thinking about something…

5: just like what this person said now, what type of disease can you say happened to a 9 year old, that something whitish is coming out. One must not keep quiet when going through anything so it doesn’t become something big in future. Just like others have said, please ask the government, and hope it won't have side effects? [Ok] (Sound of a baby crying) I'm sorry to say, I have a boy and a girl and I don’t look at them like they are immature because they know more than us. Then they talk to us and run inside, but now they will tell you they can't stay inside which made them to be more exposed than their age more than we know [thank you ma] ok. Margret, my friend’s child, when she gets to where she is going, she changes into bum shorts, the children of these days are really exposed but I just look at them. The kinds of character that I never showed while I was with my parents, these children are doing it now. But are we going to kill them with cutlass?

M: thanks for that ma, but the other side I want us to look at is that, this disease can be gotten from sexual intercourse, I'm just thinking now, if we give our younger children from age 10 this vaccine, won't they say that they already have immunization, I can't have any disease again. Let me live my life the way I want it. (All respond) let's say it one after the other.

2: will you tell them the reason for administering the vaccine?

7: we will just tell them that it is immunization. We won't let them know. It’s like a woman who went for family planning, is that not a license for prostitution?

8: you see on that family planning…

M: don’t let us talk about family planning, let's finish this immunization

8: ehn… it's into each other. It's just like a parent who took her child to do something like that. She will not tell the child what she's going to do. I remember there was a time there was a particular vaccine that was being given to adults only and we were given cards like 2 years ago. That vaccine now, though we were told what it was for but there were some women who did not tell their young adults what it was for but told them it was immunization. As for the other woman, she could not tell her child it was for family planning because the lady is much matured and the parents can't monitor her again. The lady does not know till today because she takes it every 3 years (baby cries) [please attend to the baby]. We advised her not to give the lady again as she is already suspecting what the mother was doing to her because she was sexually active and there should have been a mistake of getting pregnant. Maybe she was now using something, I don’t know. That is why the woman cannot tell her child what she was giving her because what is their age

5: we lived with our parents and got moral lessons from them.

8: when they want to make it public, you doctors will sensitize the people on its importance and when the people know what it's used for they won't come out. Like my daughter now, I’ll ensure we go together to get it. But some children are very smart now…

5: when I was younger and lived in the north, there was this vaccine that was made public and we were given, those who were older usually turn it into songs. Even though I didn’t know what it was used for then, but all the children who partook in it then, survived and are doing well. In was in the north. LEA Baptist school.

M: hmm…so we are saying if…ok, before I say that…

5: I believe that if the government can do it in such a way that they won't tell them the exact thing that the vaccine is for, like saying it is used to cure chronic malaria. There's no need of explaining to the parents. But they will ensure that they start from children from the age of 10. Because of the way children are been assaulted today, they said if they catch anyone, they will jail him. If they jail him, are there not others, if is just to achieve whatever they want in them

M: ok, just like I said the vaccine is available now, what are those things that you think might make our youths not to partake in this vaccine? What are those hindrances as a lady or as a parent?

5: what do you mean by saying we might not allow the child or parent to partake in it?

M: it might be because of money. Do you understand now? Even if I want to partake, what are those hindrances?

5: thank you. If you say money can cause it, is it that they will be going to hospitals to take it or is the government taking it to schools? If it's taken to schools, it has to be reduced maybe like hundred naira and look at the number of students in a school, even in a class. Even if we say we should take it to a community, there are some people who are very local till they die, who will not come out for anything, including the vaccine being given to the children by mouth because there was a time government brought a vaccine for birth control and they woman can only give birth to two children [ok]. Then, the first set of vaccine that was given was DA. I was there and even worked with them, when I heard the way the second batch was handled at the local government that we were misbehaving because of the money given to us, I went to tell my boss that I was done and not interested anymore.

M: thank you ma, number… you wanted to say something earlier?

P: Yes, what can make the woman or child to reject it is because, when it gets to a time… you said it's available

M: yes, and it costs seven thousand Naira [eh hen] and you will take it twice in a year

P: how much is that?

M: that is fourteen thousand Naira

P: fourteen thousand Naira. Ok, in the era of this our governor, if someone gives birth of four children and you tell them all with the children to get the vaccine. Is it possible? Even if they have the money and can pay, favouritism will set in as the rich will be attended to first while others will be on the queue in the sun suffering while they will be in the AC. That is why a woman selling wares by the side of the road will say to her daughter, I don’t have money and nothing will be wrong with you. Things like that can hinder a parent from getting the vaccine. It is possible for a young woman to think that way saying she is fine and there's nothing wrong with her as her mother took very good care of her. That could be a hindrance

M: ok, thank you

1: you didn’t do the vaccine for the local people in the community, you did it for the wealthy people.

M: why did you say so ma?

1: it is the truth. Fourteen thousand Naira. Is it the person that has ten thousand Naira and used it to sell pepper [laughs] or ‘agbalumo’ by the road side, her children were sent home from school and gave them one thousand Naira or who is thinking about the gain and how when she gets home, she will cook ‘Eba’ and ‘ilaasepo’, is that the person that will be looking for fourteen thousand Naira? That was why I said the vaccine is for the rich. If I have fourteen thousand Naira with me, vaccine will not be the first thing on my mind

8: if we look at it, that money is too much, please we plead with you the bosses who sent this aunty to us, kindly reduce the money because our people are really enduring. Even the money for WACE for this year hasn’t been paid because of money and you want me to pay fourteen thousand Naira for vaccine.

M: ok, we have talked about money; can religion or culture affect it too?

P: is there a woman in purdah that is not sexually active?

M: that’s why I'm asking

P: there is no problem with that, the summary of this matter is money because money is the root of sin. If there is no money, we’ll just look at you coming to address us with the way you are dress shabbily. You are coming to talk to us and you have different diseases showing all over your body, what we will tell you is to go treat yourself before talking to us. It's not right

9: I greet you all, if you look at that religion, we Yorubas condole anything, before the Hausas accepted the vaccine, it wasn’t easy for our people as those from WHO will go, from UCH, from state, they will all go. They will administer the vaccine from house-to-house. We will go to the ‘Seriki’. Still they won't allow their children to come out for the vaccine. You know the Hausas could be difficult at times. But we the Yorubas can still control ourselves, but when the Hausas say no, that’s it. but thank God things are better now because of the ‘Seriki’ at sabo… even right now, most of them that are educated still prefer to go to UCH than getting it from the government. There are some people among them that will tell you that they have collected money from Alhaji and told them to go to UCH, can you give us for free? I answered them that we can't do it for free, it would be better you go to UCH he's asked you to go. You see those Hausas, if there's something we can do, like picking someone from among them, both the Hausas and the Yorubas who are educated and enlighten them

M: thank you ma. So you think religion can affect…thank you ma. I want to ask the next question now. Will you allow your young ones to get the vaccine?

8: they can get it if it's not too expensive

M: ok, if it's not expensive. Number 2, will you allow your young ones form 10 years old to get the vaccine?

2: why not, I will allow her. It's for her good and also mine.

7: I can allow her but that money is too much. I still have other things to pay for.

M: number 5, will you allow and if you won't, what will be your reason?

5: it is God that gave me the child, so it is what I want that he will do. It's just the money. If the money is too much, they can have the money but will be skeptical saying this vaccine is to rescue people, why is the price that high? If they really want to do it, they can still send it through, that everybody will be able to get it. So later when they bring it again, they can now ask people to pay. But now, asking people to pay a high sum for the first time, is not possible

M: ok, so the next question is related to what you just said now. If we decide to make the vaccine like immunization, like the way children are given, in what way do you think it will be made possible? This is the way we want it done so that our youths can partake in it

P: if we say they should do it same way immunization is done and they sensitise people, you will see that people will go for it but not all

1: during my last pregnancy, I went to Adeoyo. They did blood tests and they said the blood was good but later they said there was too much sugar in the blood and asked me to come back for the test. When I returned, they asked me to come back to check my blood pressure. When I went back, they said I much take a soft drink everyday. [Ah!] By the time I went back after taking all the drugs I was given, I was told the blood had reduced. But I was told that one must collect five vaccines before delivery. I think I took maybe 2-3 vaccines before I put to birth. But what you want to do is not for pregnant women only (a baby cries)

M: what way do you think (for the community, right?) yes ma

1: there was one at alaafia hospital some time ago and we were asked to go there. I went there but it was only the blood pressure I could do. I was told my blood pressure was too high. I couldn’t go to do the test for cancer again. They asked what I was thinking about and prescribed different drugs for me. But I later went to do the cervical caner test and I was told there was nothing found in me.

M: You have not mentioned the ways

1: That’s it, you should take it to hospitals for free.

8: we can have radio sensitization. Each local government can have a social walk carrying megaphones going around communities telling them the day, date and time, the vaccine and its importance. Then each local government will bring doctors and their social workers to sensitize the entire public before the day and even during the program.

7: what I want to say is that if you take it from one community to the other, most of them won't partake in it. It will be like the immunization which people didn’t come out for. There was a rumour that they wanted to reduce the population of Nigeria.

M: so what should be done, since we shouldn’t bring it to the community?

7: what I will suggest is this, you can take it to (hospital) local government by local government and they should announce on the radio and television and usage of megaphones in the community. They can take it to schools or health centers.

M: Thank you so much ma. I am very grateful. Or does anyone still have anything to say? Ok, Thank you, I have learnt so much from you all. That’s all.
