# Supplementary material for: “She must have been sleeping around”…: Contextual interpretations of cervical cancer and views regarding HPV vaccination for adolescents in selected communities in Ibadan, Nigeria
Source: PLoS One. 2018 Sep 17;13(9):e0203950. doi: 10.1371/journal.pone.0203950 (PMC6141096; doi:10.1371/journal.pone.0203950)
Supplement: S1 CaCx data — (ZIP) [file pone.0203950.s002.zip › IDI_TRADITIONAL HEALER .doc]

**Identifier code: IDI_TRADITIONAL HEALER**

**Study participants: Traditional healer/Traditional worshipper**

M: Good morning Sir

R: Ooh

M: My name is …………….

R: You’re welcome

M: and my colleague is ………… [You’re welcome]. We want to ask you questions on cervical cancer, the causes and its preventive measures. We want to assure you that every information you give us shall be used for this research only and will not be heard else where. Do you give us your consent to ask these questions?

R: by the grace of God

M: and also to record your voice

R: no problem

M: thank you sir. Please sir, can you tell us your age, the kind of job you do, and your religion. Let’s get to know you Sir, without mentioning your name.

R: I am about 78 years and my father was a traditional healer and that’s my religion. To worship ‘ifa’ and it can also be called ‘idol worshippers’. I didn’t do any other work. Though I travelled but I returned home to worship ‘ifa’.

M: ok sir. Where did you learn to worship ‘ifa’?

R: Ahh! There are many ways. In our village at the very beginning was where I learnt to worship ‘ifa’ with my father. Everybody in our family, are traditional healers and are also hunters. So, there is no how a child…even outsiders do come to learn from us from villages to villages. At Ilogbu, where my father was born at Okunyemi’s house, they were also traditional healers before the new religion came. We used to worship ‘ifa’ and we also do herbs for the sick or for women who are pregnant. Maybe pregnant women who have turbulent pregnancies (hisses). So those are the small jobs I do

M: so can we say you have been doing this since you were young?

R: I learnt how to use herbs since I was young

M: but like how many years did you use to learn this job

R: I was asked to live with the chief traditional healer named ‘ifayemi Okunsumbo’. I lived there for eleven years. Thereafter, I went to Lagos, to in an area called ‘Moshalashi Alalukuranin’ at Iya gbogbo’s house. That woman knows ‘opele’ but at times do the two together. I stayed there for six years before my father came to take me saying it’s not that I shouldn’t go to school because I go to school there, but that everything in my head will be written and recorded and our forefathers do not want there to be any record book. That is why the villagers took ‘ifa’ worship as a good religion, because you can’t write the herbs down. If you take a biro and write down everything you learn, they will be wondering what you are using your brain for and you will be beaten. That was why I went back to read and when I finished school and returned home, I was asked to go the ‘ifa’ shrine they didn’t ask me to go to school. But with the help of my mother I was able to go to school, though I didn’t study much in school.

M: but what’s your qualification sir

R: I finished secondary school

M: ok, thank you sir. You mentioned earlier that you’re from Ilogbu

R: that’s where my father came from and that’s where I am from too [thank you sir] but we have a farm at Ibadan and my father established his name there

M: thank you sir. So if I can start with my questions. If there is a woman who is about 40 years old and above and bleeds from her private part and causes it to smell. It’s not that the blood is as a result of sexual intercourse or menstruation, she is just bleeding from her private part and she’s aged 40 years and above. The blood is accompanied with a smell. What do the Yorubas call this disease?

R: there were two names given to it before civilization came. We call it ‘arun ehoro’, we also call it ‘jejere’ because the pipe above the womb, there’s something small there at the private part of a woman. It is at that part that the disease originates from. It is that place that has the problem before she starts bleeding. It pulls the muscle of the woman. I’ve forgotten what the white man calls ‘ido obinrin’ [you mean liver?] ‘ido’- that where urine comes out from. [Ok, vagina] Vagina. So, that’s where it leads to and will pull the muscle and at night, it walks round the body and won’t allow her to sleep. It might cause hiccups, do you understand? [Yes sir] it causes hiccups. So it is a disease. It is not contracted like sexually transmitted diseases

M: but it’s dangerous?

R: eehn… [But is it also ‘jejere’] yes, cervical cancer. If this disease has not really shown and such woman has intercourse with any man, she will bleed because she’s not a virgin. So it can not be transmitted, it just comes. But we as a person cannot say what the person ate that caused such disease but we know the part of the body it affects. That part that it affects, you see, God did those body parts that have names and also put a taboo there also. Do you understand? [yes sir] if we want to look at it and say these disease should not occur again that this woman should be in her good state, it’s not until she clocks 40 years plus, even 35 years does it too. That woman at ojoo, is she… (talks to someone else in low tones), she is really not too old in age and she has this disease and has used drugs. When they say they were going to…it is a scan pictures that is used. They will ask her to run different scans, saying they are carrying out research….until someone asked, I thought you are a Yoruba person, don’t behave like the educated people, let’s go to an ‘ifa’ worshipper. But she answered him that she is a ‘shehu’. He then said he hopes it won’t be too late for her. But she couldn’t sleep, as it bites her from the upper part of the vagina to the navel. She puts her hand into her private part to scratch it hoping she can get the thing that is causing the itching out. You know there are veins there. So the veins begin to show all over her body as she scratches it hard. I mean she puts her hand in her private part. I don’t want to mention her name because she is an elderly woman (laughs) [yes sir]. So when she wasn’t getting better, my younger one brought her to me. She stays at ojoo. She then explained everything. Let’s go downstairs. I asked if she won’t be shy. She answered no. Can I see what your private art looks like? She said many people including nurses had checked and seen her private part so it’s no big deal. She opened it. When I saw it, ahh! I said, ‘Friend, something is wrong with you’. She was shaking. I told her it’s not spiritual. It’s her womb that has the problem. The sitting of the womb… [not the womb itself] no, not he womb itself. The way a person sits might affect the bum [ok sir]. Like the way we are seated, the pipe in the body is being sat on. Anybody who has never experienced any form of sickness at the private part cannot be sick. Like those who have diabetics, they know that…it is with the hole in the body that one sits on. The way you are seated, if there are problems, one will sit with one side and the anus will have no problem and one cannot locate or say that that this is what is actually wrong with the person. So, I told her that, but you educated people will ask her to do a blood test first and run scans. But we use ‘opele’, this disease; this is what will kill it. If ‘ifa’ says it bone, this is the sickness… (different sounds)do you understand? If ‘ifa’ says it’s a bone, we’ll know since the bone is one the right. It will kill her? ‘ifa’ says yes. If there’s a need for sacrifice, which our fore fathers do, will this help to beg death? ‘ifa’ says yes. We will now look for what it carries. If we say hold it in your hand and she holds it this way, we won’t look at her direction when she holds it. But if it’s the right side that ‘ifa’ drops the bone, it means that thing is not good. But there is a prayer or other things that can be done (phone rings) that will change the face of ‘oku’. Not that the days registered by God for man are to be changed, but our fore fathers then, God do allow them to alter the number of days of a man. Do you understand? God allowed them to alter the number of their days. But since it doesn’t exist anymore nowadays, because there is a drug to be used as the scientists will say no. that means the person will die. But if they eat some herbs and some native drugs, it is used to beg God, like bay leaf, we will beg God to shift when the occurrence will happen to a later day. They usually live longer between the ages of 50 and 70 years before they die. They say he’s an old man. He really isn’t an old man, as old age starts from 90-100 years. So if they use do the sacrifice, they will be healed. That woman has been healed now. So she can give me my balance that was why I didn’t give her all she is to use. After getting all the herbs, we then grind it into powdery form. We’ll now put it before ‘ifa’s consultation that was made the first time she came. We’ll then consult ‘ifa’ since I know I can do it and tell ‘ifa’ that this is the way we want the fingers, legs, water and blood to look like. Then, we say “the one that is supposed to be inside, should be inside. The one that is supposed to be outside should be outside. It is where we ask the pigeon to stay, it will stay”. A declaration has been made already. The blood that is not supposed to be a problem will not be a problem. The water in the body that is not supposed to be a problem will not be a problem. She will be using the native drugs. There is also native water that she will sit in and ensure that the bum sits in it. The heat/steam from the native water will go into those affected areas. Do you understand me? [yes sir]. Just like what you educated people do to cure fever, we get herbs and cook it and cover up over it with a cloth. The heat from it will enter…you know that every part of our body has holes [yes sir] apart from the … that you’re looking at, where the disease is, if the steam meets with it, one will sweat it out but we won’t drink it. But there are others that can be sniffed, drink and bathe with it. But there are some that are just to be sniffed alone without drinking. That is how it kills the disease.

M: thank you sir. You have said a lot beyond those aspects I have not asked you (laughs). So if I may ask and take you back a little, what do you think is the cause of this disease? You have said it can’t be transmitted through intercourse. But what do you believe causes it?

R: what causes this is the kind of food we eat nowadays. I believe that the usage of maggi that NAFDAC endorsed is a part of family planning. If you see any that eats maggi well, such person has a big belly. Where foods are not supposed to get to, they will get there. Just like you said, if you look at our men, between the ages 40-45 years will not be sexually strong with women again because they consume too much sweet things. This is what causes these things. Those days that these diseases were not rampant, or common, we wonder at anyone who gets it. We need things that are bitter in the body. Someone like you now, you can’t drink bitter leaf [smiles], we cut and eat it. Those women who drink ‘asa’, you educated people say it kills the heart. What about those educated people who have cancer, do take drink it? Have you seen anyone who drinks it? If the person uses Tobacco leaf or potash, and it gets into the root of his teeth and breaks it, except the person is very old. So if you say it’s because of the ‘asa’ he drinks that killed him, I don’t know. You see, those foods we eat those days, like in the morning, you will take tea. We do drink herbs. This works well in the body than tea as it is being taken very early in the morning. If it’s the one that cures worm and kills the worm in the body, like my wife cooks the herbs the day before or extract water from it. Very early the following morning, before I take in anything, she will bring the cooked herbs, I’ll cover my nose so gas does not enter where it shouldn’t go. I can eat meat first, or take sugar or stew, within 5 minutes I’ll drink the herbs. The herbs could be very bitter and could irritate one. Do you understand what it means to irritate? [yes sir] and cause one to vomit but the person won’t be able to vomit anything. Those types of worms can from the mouth, from the anus or from the nose when it becomes very difficult. So junks cause…what we call sweet things that it makes the body fresh…if someone says he lived in the village and you check his blood…the person that lived in the village and farms very well or climbs palm trees, do you know palm trees? [yes sir] if you check his blood, if he is about my age, and compare with this other man, his blood will be fresher than this other man who just stays in the house. This is because the person, who lived in the village, eats fresh foods. If someone goes to the farm, he’ll see different vegetables to eat. All these vegetables that have chemicals also cause these diseases. Because they are not foods our blood is used too. Carbides are used to ripen banana. You can ask market women. Carbides that forcefully ripen fruits, does not be sweeten it. But it will be ripe. Being ripe is different from the one still fresh on the tree. So these are things that cause diseases. Fertilizers…if you go to Bodija and you buy a big yam and you cut it open, give it 5-10 minutes, the colour changes. If a person eats that, it is poisonous to the body. Look at apples that are being imported, they are poisoned so it won’t spoil. We that don’t know, if we eat it, the chemical there is harmful to the chest or the throat. That is why we that are uneducated don’t eat it. Do you understand? All these are foods that scientists say enriches the blood, give bright eyes. But in the 1940s, did we have more blind people, or people using thick glass lens? There aren’t because they take herbs. There’s a saying that “the dirt in the river does not kill one”. Not all these things are transmitted through intercourse but by what we eat which is different from what God had placed in our blood. Your service to a village is establishing that village. In your family, there are things you do there, so also in our family, we have ours. In this lady’s family, they have what they do. But if you now say what they do in your family is useless and goes to do what is being done in the other family, won’t that person be hurt since we are not related? So civilization brought about these diseases. Look at the Americans, they like countries in distress so they can sell their products there. Pharmacists that produce drugs give a particular drug today for headaches. The research I did in science is that if you use drugs continuously, it could affect the neck or chest. Because he has done it such a way that if this drug is for headache, then the other will be for the chest and other parts of the body. There are some chemicals that are harmful to the body. Some parts of the body will be reduced or affected. You don’t understand? [I do sir]. Those affected parts…you will now ask people to go do scans. When I started this traditional healing, we did not have scan pictures. All we did was to ask ‘ifa’ if the disease will kill the person. If it won’t kill, then what steps are to be taken? We consult the elders. If somebody’s child had it and used certain herbs, go and meet him for the herbs. We take the herbs and drink it. But they tell us that our herbs can not be measured. But Yoruba herbs have measures. Do you understand? Just like if someone is sick and you give him water and you also give different injections to different parts of the body, if we have emergencies, we use herbs first but it doesn’t affected us. But since it has become 11 tablets morning and evening, it affects us. These pharmacists are the ones introducing these diseases and also the government who asks us to eat chemicals. Their fertilizers are not helping us. But listen, if it helps those abroad, it doesn’t help us here, because the yam grown with fertilizers do not bring forth good food. Go do a research at the market. Those that grew that type of yam in our country, they don’t eat it. The type of yam that changes colour, they won’t it because they know that they used fertilizer to grow it. The Yorubas have their own fertilizers that are used to grow kolanuts, cocoa and maize make them grow well but does not affect us. Do you understand? [yes sir]. Like the family planning you do now, we use rings to do ours. We don’t do incisions. We’ll put something in the woman’s hand and we’ll tell her things that she shouldn’t eat like locust beans and snail. Do you know what snail is? [yes sir]. Those are the two things she mustn’t eat. Peradventure she eats it in a soup, what was used to prepare the soup must be given to her because of the day she dies. The father might have died prior to when she wants to get pregnant. If she eats the taboo, she will definitely get pregnant. We’ve seen someone who took family planning injections who have not been able to get pregnant till now, because the injections have overdone the work. Certain things like that are brought to us.

M: thank you sir. So you have mentioned what you believe are the causes of this disease. But if we look at it from the perspective of traditional worship, what do the Yorubas think is the cause of this disease?

R: what Yorubas believe is that whoever does not use drugs for sicknesses called diseases because they don’t know the names like you know them now. We just say the person has a disease. Do you understand? [yes sir]. If we focus on what you’re talking about now, cervical cancer and epilepsy, sometimes work hand-in-hand. Cancer is not always wet, do you understand? [yes sir], so also epilepsy is not all the time. But there is a particular time when it arises in the pus. Do you understand? [yes sir]. But if it gets its cure, it will stay.

M: thank you sir. Is there… if we look at it…I’m still on what Yorubas believe is the cause of this disease. Can we say its spiritual or like you said, maybe the person didn’t use some herbs might be the cause? But more importantly, could it be spiritual? Can it occur?

R: (laughs) well-done. Everything that has advantage has its own disadvantage. Do you understand? [yes sir]. The one that is good will have side-effects. Truly, Yorubas do such. Especially, what you’re talking about, it teaches us not be abusive. We shouldn’t keep malice with either men or women. Now, if I see a beautiful lady, you know everyone likes something good. I’ll approach her that I want to marry her or have her as a mistress. Eeh (claps)… with the way you look, old man, with your big eyes (laughs). You mean me? I will make you know that I am a man. He can know where she urinates and pour something there, something that has to do with the private part and pour it there. The woman now becomes sick. Just like the one done to the leg, the one called ‘Elephantia’ ehn? [Elephantiasis] have you seen it before? [yes sir] they are all diseases. We call something ‘obe ilase’, if the person was disobedient, it will be removed. One can say he wants to see it and it will be cut open. But it won’t be cut towards him so the pus does not touch him if not the person will have it too. If the person has it on the face and there is an area that can be cut open, that is the way the face will look like. That part of the face will be cut open, that’s all. After some days, it will begin to itch, and then veins begin to appear and hard. When it itches, it then begins to develop according to the herbs put there and the pus will be there but won’t flow out. After sometime, after the herbs begin to work, it starts itching well and becomes wet (chuckles) so as regards that, it could be spiritual. But in Yoruba, they learnt what causes diseases in man. There is something we call ‘ori tulu’. It is what you asked for that prompted me to go deeper into this [thank you sir]. Though it’s not proper in religion. But traditional worshippers, Imams and Prophets are ‘Alamala’, because they all use herbs but lie to us. Especially those ‘shehus’, they use herbs but are liars. Likewise, other religions, they are liars. They say they don’t use anything but there is something fishy about them. Do you understand? [yes sir]. If you make jest of them, they can afflict the person with a particular sickness. There is something that is called ‘oku oru’ and also an ailment called ‘gbari’. ‘Oku oru’ does not happen during the day, only at night. There’s also another ailment called ‘tulu’ that happens in the head. I don’t know the English name. It is a serious headache. Ehn… [Migraine] Migraine… all parts of the body is being troubled. It can be done by using someone’s hair. That’s for those that know it (laughs) we didn’t learn that. Herbs that can be used for a sick person and get healed, for a woman to get pregnant and anyone with sexual transmitted diseases to heal them, those are what we learnt. [Thank you, Sir]. But those days, there are people who do it and are dead now.

M: thank you so much sir for go deeper into those things [phone rings]. Like how many women have you come across that have this cervical cancer we are talking about? How many have you been able to heal?

R: ah! When I was still under my boss at Ipetumodu, my boss healed three (3) cases. We were the ones that got all the herbs that were used but we were not the ones that examined her private part to know what exactly is wrong with her. It was our boss who saw it and told us that it is the cervical cancer which is called ‘ehuru’. There are two types of ‘ehuru’, one is when that woman has intercourse with a man and he stands up, she starts bleeding but after 2 or 3 minutes, the blood will stop. Even if she has intercourse with another man 2-3 days after, she will still bleed. You know this is different from the one of the womb? Eh hen! I as a person, I healed someone of it in a town called ‘tola’ in Ibadan here. I also cured someone of cancer of the breast in this same village. My elder Sister… ah ha! I healed her. There was a day… not knowing that the people I was treating are also learners. I never knew I could see the private part or the secrets of a woman and run. Do you believe? [I don’t believe sir. laughs] what actually got me scared that day… very good… this child is around now. They alerted me that there was a woman who had stomach pains and water was coming out from her private part. I said it was something small, not knowing she had cancer. She lay down on a long bench, something that looked like this. She lay down and spread her legs, it was the first time I saw something worse. Even her elder sister couldn’t look at it. The cancer had really eaten deep into her which brought the womb completely out in between her legs. It’s like when a man sits and his scrotum pours out. I stood at the door and was shaking. The elder sister then said looking at her sister’s private part got her scared. Ah! There’s something wrong. Like what I was told, they said she told her husband that she’s more beautiful than he is, the husband responded that he’ll destroy her beauty. He used charms on her and her private part came out. I told them this is not charms, but that she ate something she wasn’t supposed to it. There is a particular incision that we do for this, if it then does not go and troubles are more, will keep the drug and put it one after the other. After that, it kills the disease. We do incision, which is our own injection. Is that not so? [yes sir] I didn’t see the labia anymore. I meant she has been healed because the labia came out more but I couldn’t see it after that. When we were done, I was given money. But I couldn’t go back there because I didn’t want to see anything bad. You child…you know children don’t mind, so I told the children to go and apply the herb and get her blood. He will go because I never saw her for the first time, it’s just that I don’t want to mention names; she is at ‘oranyan’ today. She has given birth after that. She delivered at Ajao Hospital at Olorunsogo and went to celebrate with her. When she delivered the second one, one hospital, Molayo or so, it is also at Olorunsogo. I was told she had delivered and I went there to see her. We greeted and hugged each other. She was completely healed and she sells good presently at oranyan market where ‘oniparo’ usually are. But since we shouldn’t mention names, I can say she was completely healed. Is that not the second one? [yes sir] I also healed someone who hails from Ijeshatedo when I was at Alaba when she was brought to me. There is this hospital at ‘Alajameta’, called Lanporos hospital at Alaba-Ewe. The doctor said they will cut it off according to all the research made, it is better to cut of the womb but she won’t be able to conceive. I remember telling a woman selling firewood joking that I was going to marry her and impregnate her with twins, even if you are Ibo, as long as you are pregnant for me, I am your husband. I was joking when I saw her crying and saying won’t she be able to conceive again? She came down from where she usually sits. My house is on this side and I do sit there watching there. She was then discussing that something is wrong so she wouldn’t give birth again. Ah! I asked her what happened. She said her womb was bad. I didn’t know it was cancer because since I had not seen it. Then asked her what was it that they want to cut off. Taye is the name we call the Nurse at Alabameta. I do joke with her that I want to marry her. Let’s work together. I do traditional healing, you are educated. What you know and what I know can be put together to work. If you accept, it will be easy for you. So we flirt together, saying how can she marry an old man. I do tell her no to worry, I can sleep with her, and then goes out to whoever she wants then returns home to give me food, is that settled? Ah! People will say that’s that old man’s wife. You haven’t had any children I have. You can be going about or maybe she has children already and as we work together, it will be to my gain. If you use the educated people’s style, I’ll use the traditional style. Then I wondered how the womb could go bad, could it be that she committed abortion, they said no. she then explained that there’s a disease called cancer. I asked her to explain what cancer was. She said it’s a disease that affects the womb. As we were talking, I told her I could heal the woman but you won’t want to pay as they will say they have spent so much at the hospital. Because of the number of scans she had done on several occasions. This wasn’t available in those days. Scan then was about seventy Naira, but now it’s in thousands. But to the glory of God, she had given birth before I left that street. On the naming ceremony day, I was treated like they were treating their in-laws. I ate it with love and drank gulder. That’s all. But I don’t think…is available nowadays. Because any small problem, you will see her, even her mother’s elder sister visits me regularly without knowing each other before. It was during ‘ifa’ festival that they all came to give thanks. After that… I have healed six cases in all. The last person I healed was the first one I mention earlier about the woman at Ojoo. So they grow lean when they have that disease but now that woman has gone fatter all over with bigger breasts and bum [chuckles] ah! It’s true. She was really lean with very small breast when she came to see me but now, she’s bigger

M: thank you so much sir. May you continue to do well

R: amen and you too

M: amen sir. But how can we put an end to the disease?

R: a way we can put an end to it, you educated people want to know. Hmm… Yorubas used to say in those days that “when something is coming, we set a trap for it and watch over it”. A way I believe we can put an end to it is not with the usage of ‘chalk’ (drugs) that you give us to use. The most preferred is to go the traditional Yoruba way. Herbs that has to do with the throat, worms…there is nothing worms cannot do. There’s a disease called ‘Eela’ that affects the face…ehn? It also affects the leg. May you always be sane. If we can be drinking ‘Agunmu’ not the one that women who did not learn it sell. The government is not doing well. Whoever forms an association and has gotten license, that person knows the job well. You will see that some people do adverts on radio that there are no herbs that they don’t know. Someone like me will relax a little because if someone comes to learn from me and I teach him, he’ll then go to the radio and start adverts, I don’t do that anymore. If he is an ‘Alfa’, he should continue to use the Quran for his healings and a pastor should also continue with his water that Jesus taught him. I won’t teach again, because they lie a lot saying they don’t use herbs and you use and drink it, you will hear that everyone preaching nowadays, they don’t mention anybody other person than herbalists. Is it a lie? Herbalists are not good. If you know herbalists are not good, why collect things from them and use it. If they start a church and they want people to come, they will come and meet us. There are many things that we could not do because of money and they collect money per head and give the money to us and it is money a woman knows, while we do the native medicine for them and we are the ones they still preach about and it’s all lies. Jesus did not…whoever God calls die. But what I believe in his that the ‘Agunmu’ we take, if someone says he’s educated, he should drink it. Do you understand? [yes sir]. The herbs that make one vomit too should be taken at least once a week if he isn’t going to work. He can take it around 5, he would have to better now. If he drinks the herbs around 5, he will sweat. We never went to go to the hospital for healing those days and people get healed. When a baby does not come out in the mother, we do not do surgery and the baby will eventually come out. If the person is under a spell, we’ll call it out and the baby will turn downwards. And if the baby is upwards, he will come out easily without causing a tear on the mother. Or that they baby is big…what do they say they do on the women [tear. They cut open] eh hen! Tear. We don’t tear anything, we will call the baby forth, every part of the body conforms. If a baby comes out with the hand first, that means there’s something blocking it and we’ll speak to the blockage and it gives way for the baby to come. The baby comes out with the head or leg. The mother will be fine. As for our own medicine, let’s endeavour to use them. As powerful as the white man is, that power becomes bad. For instance, if something is wrong with my hand, the white man will pour something into it and the odour won’t change. They have that power. Our own medicine expires, especially the herbs but ‘Agunmu’ and any burnt herbs do not expire. Like the herbs taken with alcohol, though it was civilization that brought the alcohol usage too. Hot pap is used to take the worm expeller. But when civilization came and everyone fought for fame, this brought about the usage of alcohol to take herbs. Alcohol too isn’t good for the body. You will hear about that later [thank you sir] It is advisable to take our herbal medicines and stop eating maggi. Also all sweet things bring about one disease or the other. Those people… this secret I just revealed to you, if you ask the professors or consultants…those that did maggi should show them. If there is someone among your bosses who has not secrets will confirm to you that truly, it works. Development is not useful. A woman who hasn’t stopped childbirth begins to add weight and fat in places that there shouldn’t be fat. Maggi is not good for us to eat. There’s a root called ‘isingun’, locust bean, onions and ‘ogiri’ are what our fathers used to cook stew. If you use the isingun root as pepper on yam and you eat it ((inaudible 47:35)). If there any disease in the body and smell the food you just ate, it goes to the anus to come out as faeces. If there’s a disturbing gas in the stomach, once you eat isingun, it comes out but maggi does not come out, it will just be sweet. It’s been used for garri and yam which is not good. It is a chemical. Those are the things that cause diseases that we know. When grinding machine came those days, our fathers never allowed anyone to use grinding machine because we believe that the iron grinder will melt into what we will eat. The iron grinder in it…you know they file it. As it files, it get into the food we will eat. We use our hands and grinding stone to grind our pepper. Even if we say that style is not possible now, but my child still uses hand and grinding stone to grind our pepper. They are grown like you and are in the university. Do you understand? [yes sir]. When they are in their school, they can take two packets of maggi but when they come home, they know that they won’t eat it, because one will be beckoning on an ailment. White man’s medicine is not good. But a way that your drugs are better than ours is when something is wrong; you’re given a pain reliever. When used for headache, that’s all. But the pain that was thought to have gone, returns after two days or is it a lie [no sir] he will still have that feeling. But if you use native medicine like stomach pain during menstruation, or it hasn’t come and there’s pain, she cries that day and the following day. But when it eventually comes or during delivery, she would experience any pain. Whatever is wrong, the medicine would have killed it. But your medicine will make her sleep, by the time she wakes up, the pain is still there. Now, I have tooth ache, if I get our medicine and apply it, it will be healed. But the white man’s drug I used, if a sleep and the teeth touch each other, it’s a problem. I can’t eat meat. Do you understand? [yes sir]. If one goes to the hospital now, they will say they want to remove it and refill it. That’s it. But when we use our own medicine, it could be painful and might bleed. But if it kills what is below, what ever the odour, it will kill it. Do you understand? [yes sir]. But for quick relief, we credit you. For that minute, one would relax. But if we use your drugs too much, and the body gets used to it, it won’t work again. Or will it work? It won’t. if you take our medicine, nothing will happen. If you take Agunmu, nothing will happen. The incisions made today work well. But if we use white man’s medicine, they will even write another one for you to take to ‘Kunle Ara’ and get it. But this is the same thing that you said was wrong the last time so how come it’s still the same thing now. Let it work then. That means the pharmacists have introduced another drug. One will even have another disease. You observe the rich men when the new government came in, they said there is no one that will be sick and fly abroad for treatments. Is it a lie? They will look for herbs and roots and send it to them. Can’t our doctors cure diseases? You see someone who can cut open the head and remove whatever is disturbing the ear. Or is it a lie? [no sir] He will open it up and sew it back that it becomes the way God made it. Like those doctors at UCH, there are some that create their own, they don’t they go there. They won’t go there but they are wicked. That is why they introduced a drug that isn’t good for them, despite that God is greater than man. That is why they die there. By doing so, what I can tell you is for us to stop the usage of magi and taking of too much tea. If tea is too much and the sugar is much too, diabetes is knocking and can not be transmitted. Not to say that someone that has diabetes has an intercourse with a man now and the man gets it, no, but if the man also continues to eat what the woman eats, he can get it too. Is it a lie? Have you been told about it? [yes sir] hope it’s not a lie [ah no sir] and I know that…do you use maggi to cook yam? [laughs] what do you use to cook yours? Sugar? [daddy, it’s only salt, unless it is yam porridge. But it if is plain yam, then it’s only salt]. So if it yam porridge, you will add magi. [laughs] look at you, don’t eat it again. [thank you sir] Do you understand? [yes sir]. Fry pepper and eat with the yam, grind the pepper with locust beans, fry it and use to make the yam porridge. If you see good onions and slice it to the yam porridge (another man talks; buy onions that have roots) pour into it, you will see the difference. Eat pepper well. It’s just a lie when they say when one eats pepper, one would have cancer. Or which one is it? [Ulcer] ok ulcer, that affects the chest, it’s a lie. The Yorubas say ((inaudible 54:45)). Eat pepper… you see its good to eat something peppery because if there is anything that needs to come out of the body, it does so by sweating it out by eating pepper. When you need to pass out faeces, you feel the pepper at the anus. But when you came, you said we should add tomatoes to it to reduce the hotness of the pepper. If an educated person that eats these and takes all drugs talks, there will be saliva at the corner of his mouth. That shows he didn’t take care of himself. There is no hotness because pepper warms the heart. If the heart is warm, no disease can get there as the pepper would have killed it. Those people that eat pepper and grind with their hands don’t die at the age of seventy (70). When my father was still alive, when his friends die at the age of 65 or 70 years, he cries and he wont be able to attend his funeral ((inaudible 56:33-56:42)) but if it was in those days, people mourn. For 72 years, people still mourn, if they see an any of the children walk past, they cry and say because the children or friend have no one. But now, bodies are taken to the mortuary. Those days when they want to go bury someone, they roast the body first and every good part of the person is gone. The body is then thrown down. I remember going to UCH, the smell was bad. So I took kolanut and began to eat it, an educated person saw me and said I shouldn’t eat anything. I told him I was going to eat, is the person not part of us? By the time the body was brought, I thought the hospital workers were going to bring him in the cloth but they carried the body and threw in up and it came down [so the ice can fall off the body]. That’s a punishment. If someone dies and the family doesn’t have the money to bury the person, cover the person up. This is the day I want to bury my father and I told them I don’t have money for him to be kept in ice and put in a cold place. They will inject the intestine [they even cut them open, remove the intestines and sew them back] ah! Jesus (different sounds) its all chemical [ah…daddy, this is another topic]

M: thank you sir. So you have told us how to prevent cancer

R: the way we can prevent them is to go back to herbs

M: thank you sir. So how can we take care of people with this disease?

R: someone like that, if he has money, one can prepare herbs for him, prepare Agunmu and also perform a sacrifice for him. That sacrifice is our own scan pictures. By the time with use opele, we’ll see if whatever is wrong with the person came from birth or not, or it’s just a disease. If its a disease, if we do it this way, will the disease go? It says yes. That’s all.

M: thank you sir. Can this disease be transmitted?

R: I don’t know. I never learnt that. I have not seen a woman contract it from another woman or man. I don’t know about that. But I believe it can’t be transmitted.

M: so you believe it can’t be transmitted from one person to the other.

R: for me, I believe it is not a communicable disease. But if it is transmittable, if any woman that have cervical cancer sleeps with a man that has many wives, then others would have contracted it too. Is it a lie? [no sir] but my own believe is that it can not be transmitted

M: thank you sir. But is it possible it runs in the family?

R: I have not healed two people from the same family so I can answer that question well. Like the one I told you I healed recently, if she came and said it had happened to her elder sister before, which means it is transmittable. But I want to beg us all, the bra and pant you wear should not be borrowed, weather washed or not because they emit different levels of heat. You see that, the odour of the sweat from the armpit differs in people. So I don’t know if sharing underwears could have any side effects, especially when we borrow each other’s clothes. There is a particular disease called eczema. Do you know what that is? [yes sir]. It is communicable [eczema]I have learnt an English word(he greets somebody). So for this reason, I have not learnt about it.

M: thank you sir. So presently, the white men have introduced an injection for immunization. And it is said that whoever takes it is prevented from cervical cancer. Will you endorse it so people can take the injection?

R: as a person, I don’t believe in it.

M: what’s the reason sir?

R: thank you. You see the very first time they brought… it was called an injection for prevention an we were given on the arm which later swells. They said it prevents different diseases. But someone still had a disease. That drug expires. At the hospitals where its being given, do they have stroke or not? I don’t believe in it at all. Other than, what the body rejects, one should not do it. These are part of what I talked about earlier. The pharmacists are the cause of the many diseases in our nation. If you say you specialize in a particular drug, you bring yours and another stands to say I specialize in fingernails. Another will say I specialize from the waist down. And as we use it, you see that before one disease dies, another springs up. If truly, these drugs work, then all these capsules and tonics will reduce in our stores. If truly, you are a specialist in a particular disease, it’s just a drug you will keep producing. But they are all liars. It is the person that has money that has the certificate. Money buys certificate. Those days, even the white men do not believe in certificates. If you go abroad now, as I am a traditional healer, if you say you are a mechanic, the white man will say… they will put you through first and learn it. Their own is to see that you did job well. I don’t believe in the injection given on the arm. I don’t believe. Even the prevention given to women should not be used again as it is a sin before God. Prevention: not getting pregnant after intercourse is a sin. Because now, you can’t tell how many children I’ll give birth too, but you have killed one there. We don’t know the good or the child that will die. Or is it easy for a child not to die? There’s a doctor at Ikeja, he is a top doctor not a learner at general hospital, Ikeja, my child was sick and I asked the mother to take him to the hospital when his temperature was rising. I had used everything I had at home, we were not happy because it took at while before conceiving him, I wasn’t just happy. So the neighbours asked us to take him to the hospital. So we took him to general hospital. My younger brother, God be with him. So we took him to the hospital. All the nurses on duty did not attend to my son. The doctor’s child that was looked after by someone at home, the wife has gone to work and the doctor was at ikeja that was how they rushed the child in. The doctor, nurses, hospital maid, everyone rushed to attend to the doctor’s child. But my son was very sick, I began to cry. An elderly matron came and asked why I was crying. I explained to her that I did not see any doctor or nurse to attend to my son. She said that their boss’ child was rushed in. I said it’s not the same section but she said they went to sympathize with him. I actually went to see the boy as they were pulling all his parts and sing a device on his chest. After sometime, another doctor who is albino, shook his head and said, “I’m very sorry”. As I heard that, I went to meet my wife to inform her. When they say I’m sorry, that means the person has passed on. I couldn’t laugh, neither could my wife. You know that everyone that rushed there will come and attend to us now. I told her to sit while I spoke to my son telling him to hold on as the doctors would soon be around. Told her to give him breast milk but he rejected it. I was wondering if the child will die too. Just then I saw the doctor coming, wiping his eyes with a handkerchief. The first person who went it, he shouted at the person to get out and go to LUTH. As it was our turn to see the doctor, I pray that the doctor will not give us a wrong prescription because she wasn’t concentrating because of the loss of the doctor’s child. Everybody left, even the pregnant women in labour. They wanted their own child to leave, but see God’s judgment. I was told the doctor has just two children, and one just died. Maybe they had removed her womb. They say children must go to universities; do they get jobs with their certificates? No, rather they ride motorcycles for a living. Family planning is not in God’s laws. Believers do it but herbalists don’t. If it not time for God to give a baby, if there’s intercourse, the woman will see her period. She will eat herbs and roots and it won’t come. That’s family planning by God. A man that moves love to a woman, 3-5 times before daybreak, when it comes to fasting, it’s really hard for him not to sleep with any woman. Even if he has sperm then, it will not fertilize, because he is forcing his body and drinking all sorts, nothing can happen, that’s family planning. But family planning by counting the number of children you want to give birth to, you are supposed to ask yourselves, if you give birth to three children, can God ask for one? Or die accidentally. We give birth to as many as possible until we can’t again. We marry wives because we don’t know the destination of the child that is coming. Look at Arisekola, he had a poor background but did he not prosper? What did he study? Those are rare children. Mofu Lanihun, what did he study? You have heard the name before, right? [yes sir] ((Inaudible 1:10:25-1:10:28)) did you hear about him or not? Many of those rich people like that. What about Raji Rasaki? You heard about him? [yes sir]. His parents were not rich and did not finish school but he related then with the vice president in Nigeria. Arisekola studied up to primary 6. Adedibu, what did he study? I’m not sure my father went to school (laughs) ((inaudible 1:11:18)) is it a lie? you were born then during Akala reign, was it his level of school that got him there? But if his mother had killed him then without knowing his destination, no one would have heard their family name. Please, tell your bosses, stop family planning. Any number a woman wants to give get birth to, she can go ahead and not hinder God. If she has 3 children and they all die in an accident and educated people like to leave home together during weekend. They 3 children then die in an accident. Then she cries. So look at that. We even understand the white men, there are those that have money and say she won’t have children and will all her properties to the motherless. Do you understand? [yes sir] what is the name of this white woman? [can’t remember]they usually bear funny names, she has money, she is a senior lawyer, she has about 10 offices in her building but does not want children. But when one of my children started dating her, she told him that she just changed her mind that she has fibroid and does not want to remove it (clears his throat) so when she saw that my son was genuine and wanted to marry her, she decided she was going to remove it and have children. He told her not to talk about operation that his father will remove it. But she asked how possible it is for his father to remove it. She asked funny questions. He answered her that she will be given a soap that she would use to wash her private part, though it will be painful. She said she doesn’t want any pain, that’s why they don’t give birth. The process of delivery is painful that’s why they don’t have many children. Because if she’s cut open at different parts, there is no other part to cut again. That is why they don’t have many children not because of education. All of them do not marry white men, did you know that? That does not understand English at all but speaks their native language. Just as I have tribal marks on my face, you come to meet me at the secretariat and greet me in your native language, I will answer in English. You will say that this is an educated person which is not meant to be. The Hausas did not stop speaking their language, but we did. That’s the way it is

M: thank you sir. Thank you for all you have talked about. We do not have any other questions.

R: thank you too. Hope you are satisfied?

M: yes sir. We’re satisfied. Thank you, sir.
