# Supplementary material for: “She must have been sleeping around”…: Contextual interpretations of cervical cancer and views regarding HPV vaccination for adolescents in selected communities in Ibadan, Nigeria
Source: PLoS One. 2018 Sep 17;13(9):e0203950. doi: 10.1371/journal.pone.0203950 (PMC6141096; doi:10.1371/journal.pone.0203950)
Supplement: S1 CaCx data — (ZIP) [file pone.0203950.s002.zip › FGD_OUT OF SCHOOL_FEMALE.doc]

**Interview group: Out of school female adolescents**

M: good afternoon, my name is ………. and with me is ………….. and ……….. We want to ask you questions about cervical cancer, hpv and hpv vaccine. We want to know what you know about it, what you have heard and where you heard it. do you give me permission to go ahead with my questions? [all: yes] and I have the permission to record your voice [all: yes] so have you heard about **cervical cancer before?**

7: no

M: any other person?

2: no

M: let me start from cancer, have you heard of cancer before?

1: yes

M: what have you heard?

1: what I heard about cancer is that….okay what I heard about cancer is that cancer is caused by what we usually eat like can food and…

M: any other person? Where did you hear that?

1: when I was in secondary school

M: they taught you in school? [yes] okay any other person?

3: cancer is a disease

M: okay where did you hear that?

3: from my sister

M: any other person?

4: cancer is a disease, I know there are different types like breast cancer and there is eye cancer but I don’t know how people get it

M: where did you get that information from?

4: from a movie, I learnt that the guy has eye disease and they said it is cancer

M: what of the other type of cancer?

4: from my brother

M: as they have all said, cancer is a disease and as she told us now cancer affects different parts. She mentioned breast cancer and cancer of the eye, it can be skin, it can be everywhere. Cancer just means a cell keeps growing out of proportion , it keeps dividing and keeps getting bigger

10: how are we going to know if someone has cancer?

M: by doing a test. So we said cancer can be in different parts of the body. But the one we want to discuss now is cervical cancer

2: what is the meaning?

M: I am the one asking the questions not you. [laughs] so as anyone of you heard of cervical cancer?

All: no

9: no

M:okay cervical cancer is the cancer of the cervix. Do you know what cervix is? [no]do you know what womb is? [yes] if someone has cancer at the entrance of the womb, the entrance of the womb is the cervix. This aunty that was asking for the meaning ((all laughs)) we have cancer of the womb but this one is the entrance of the womb. That is what we call cervical cancer. And you said you have not heard it before, let me describe the symptoms, maybe you have seen someone it happened to before but you didn’t know what it is called. So it happens to women that are 40years and above, they are the ones that manifest the disease. If you see a woman of that age bleeding from her private part and it is not that she is on her period, and produces a foul smell. Some will lose weight and back pain and may end up leading to death. Has anybody heard something like that? …. ((nobody)) so nobody has heard anything like that?

1: I have heard that a lady was bleeding through her nose

M: that is not cervical cancer, she would be bleeding through her vagina. You said you have not seen such before but let me ask, what do you think can cause such in a woman? What can make a woman to be bleeding and it is not that she is on her period? What do you think can cause it?

5: if a lady…

M; is it a question you want to ask?

5: no. if a lady just had an abortion

1: if a lady has early marriage

M: please explain that, early marriage as how?

1: if a lady marries at about 16, a7years and she gives birth before she gets to 20years, it can cause such later in the future

M: any other person?

4: Cancer can be cause by diseases [ cancer is a disease] infection [how] toilet infection

M: okay you think toilet infection can lead to cancer?

4: yes

10: HIV AIDS can lead to cancer

M: number 7, what do you think?

7: infections can cause it but I am not sure if it can also be caused by sharing sharp objects

4: having unprotected sex

8: toilet infection

9: toilet infection

M: say your own

6: maybe if someone is having stomach pain

3: like ulcer

5: floor disease

M: which one is floor disease?

5: if someone urinates on the ground and the heat and dust from the ground enters your vagina, it can cause cancer

M; any other thing? What do they say in the community? Number 5 where did you hear what you just said?

5: it was from my neighbours

3: diabetes can cause it

1: by eating too much rotten food

3: and by taking too much of sweet things

7: drinking too much of soda

M: you said you have not seen anyone that has this cancer before? How do you now think it can be prevented? You have said how you think it can be caused, now how do you think one can prevent it?

3: by using medication

M: is that for after one has the disease? You know prevention is before contacting the disease [yes]

1: by reducing the way we eat can foods

9: by going for regular check up

10: when a lady gets pregnant she should not attempt to abort it

4; by having knowledge about involving yourself in unprotected sex

M: has anybody heard about human papilloma virus?

7: no

9: no

M: but do we all know what a virus is? [yes] a virus is an organism that we cannot see, it is this virus HPV that cause cervical cancer. What does HIV cause? [AIDS] , that is the same way HPV causes cervical cancer. And how is HPV spread? It is during sexual intercourse that the virus is spread from one person to the other. One can contact HPV at 10years, but will not cause the disease until later age of 40years. Some can contact the virus and it will not lead to the disease. Do you understand me?[yes] now has anybody heard about HPV vaccine?

3: vaccine is an injection

M: what is the injection for?

3: it is for prevention

M: as she as said- but has anybody heard about any particular vaccine called HPV before?

All: no

M: as she has said a vaccine is an injection, it may not necessarily be an injection, it may be a droplet but it is to prevent a disease. It is like these ones they give children so as not to contact the disease. It is not for someone that already has a disease. Now there is a vaccine called HPV vaccine and they said people that are 10years above should get vaccinated before initiating sex, before they are exposed to a means of getting the virus. Do you understand that? [yes] it is available for 10 to 12years and you will take it twice. Each dose is 7000naira [ah] wait now ((laughs)). Taking it twice is 14000naira and it is to protect you that before you initiate sexual intercourse and get exposed to the virus. My question now is that what do you think are the advantages of administering this vaccine to adolescents, people that are 10-19years?

3: is it advantage or disadvantage?

M: advantage first, we will still go to disadvantage

8: it will reduce death rate in the country

7: it will make them aware of HPV that causes cervical cancer

3; prevention is better than cure

1: to prevent them from the disease

3: they should take their medication regularly

M: what is now the disadvantage of giving this vaccine to adolescents?

8: when they find out that they have the vaccine in them already, they will begin to do what they wish

M: what do they wish?

8: they will start having sexual intercourse anyhow when they are not supposed to have sexual intercourse: any other person? If you have another opinion, say it, just tell me what you think. All of us have once been adolescents, some are even adolescents here. So what is the disadvantage of giving the vaccine to you and our friends? Number 5 you want to say something?

5: no

M: so we only have one disadvantage?

7: once they get the vaccine they will believe they do not need any medication again

3: is this vaccine medicine or injection?

M: it is an injection. Say what you have on your mind, you have a reason for asking that question.

3:…

M: so what are your fears and concerns about this vaccine? ((no response)) has anyone here taken the vaccine?

All: no

M: why have you not taken it?

10: I had no idea of the vaccine

3: I have

M: where did you take the vaccine?

3: at uch

M: when?

3: last month

M: who told you about it? but you said you have not heard about it before

3: my sister told me about it

M; how many doses?

3: two

M: why did you tell us that you have not heard about the vaccine? So what made you take the vaccine?

3: when she explained to us [who?] my elder sister, she told us about the vaccine and what it is meant for and that we must take it

M: so for the ideas, why have you not taken the vaccine?

6; I have never heard about the vaccine

9: I didn’t hear about it

M: now that you have heard about the vaccine, will you take it?

5: when they said it is 7000naira ((all laughs))

M: you see, that is one of your concerns, she is concerned about the money. Do you understand my question now? [ yes] why will you not take the vaccine?

5; we will take it, we must take it

M: number 2, will you take the vaccine?

2: yes

M: why?

3: it is because of ourselves

M: let her answer

2: it is because of ourselves

M:ah ah say it your own way

2: to protect me

M: so nobody has any reason why she will not take the vaccine?

9: someone can take the vaccine and it will go and cause another disease

M: so she is concerned that the vaccine will cause another disease. So what are the other questions worrying you now? She has said her own that maybe the vaccine has side effects

11: I like taking drugs more than injection so I will not take the vaccine because it is an injection

3: if someone takes the vaccine and then go ahead to have sex with someone that has HIV, will the vaccine cure the HIV?

M: no the vaccine only prevents HPV. So you said you don’t have reasons why you cannot take the vaccine, so if I say we brought the vaccine-

3: I cannot take it

M: why?

1: because of the price

3: except if we will not pay money

M: I have told you the price. You will pay

8: but we are students

M: you did not say that before. Number 4 will you take it or not

4: no [why] because of the price

5: no because it can react

6: I would have loved to take it but I cant because of the price

1; the reason I will not take it is because I do not have the disease, if I know that I have the disease I will take it

M: but the vaccine is not for those that already have the disease, if you do test and it is found that you have the disease, it is treatment you will for and not vaccine.

7: I want to ask that how can you know that someone has been infected by this virus?

M: By going for test. If it has not developed into the disease, it will not show on the face, so it is only by going for test that you can know

3; what if someone does not have money for test?

M: you will look for the money for test

4: so you mean there is no sign and symptom of the HPV?

M: except it has developed into cervical cancer

9: can you tell us the signs and symptoms of cervical cancer?

M: I have said it before, who can tell us?

Participants: bleeding, back pain, odour

M: we already said it. you can ask questions later, for now I will be the ne asking questions

M: do you think your parents will allow you to take it?

7: yes and no. yes if they get to know what HPV is about but if they do not know they will not allow me

8: if I educate them on what HPV is they will allow me

9: yes if they have an idea of what the vaccine is for that it is to prevent a disease

10: yes

M: if you go home and tell your parents now there is a vaccine you have been told to get so that I will not contact a virus when I want to have sex and they said it is 7000naira

2: yes they will allow me if they have the money

4: yes because my mummy is a nurse and I am sure she knows about cervical cancer. So I am sure she will allow me to take it

M: even if you tell them that the virus is for a disease from sexual intercourse?

All: yes

M: what will the community, your neighbours, your friends say?

8: they will also like to get the vaccine

M; let us even go to the churches and mosques, what will they say?

3: because of the money , they will say the money is too much

M; so its all because of the money?

3: yes

7; some will discourage you

M: why do you think they will discourage you

7: because of the price and they will believe that you are already having sex, they will say you don’t need it at this your age

M; what of your churches and mosques, will they accept it?

((chorus answer))

3: they will accept it now

1; some will accept it and some will not

M: why will some not accept it?

3: because of the money but they will accept it because they know it will cure- prevent a disease

10: they will try to discourage it because they will think you want to take it so that you can start having sex

M: so you think churches will discourage you?

10: some churches

M: if we want to make it a routine schedule immunization for adolescents, in what ways can we ensure that adolescents take the vaccine?

10: the government should try to gather some educated people like corpers to help educate people in the community and try to advertise the vaccine. As we have had the opportunity to hear of it now, some people have never heard of it , so the government should try

8: through advertisement because most of the people have not heard about it before, so people will get to know

1: for the health workers, they can distribute themselves into areas, like 4 people will go to one house another group to another house

M: so they should go from house to house?

1: yes

9: through tv and radio

4: public enlightenment

M: number 6, say something. Nobody is saying anything about the money, nobody is saying anything about the location, where should they put it

8: the government should try to reduce the money

3: is it government that will do that?

M: that is her own opinion, let her say her own, you can say your own

8: the people that are producing it

M: number 3 you were saying something

3: nothing

4: the government should pay some of the money to those producing it so that the price will be reduced for those that will get it

3: they should have branches everywhere

M: where?

3: everywhere [in local governments]

7: they should advertise through hand bills

1: through mass media, billboards, posters

M: so you have said they should reduce the money, someone said government should subsidize, some mentioned public enlightenment, there should be branches in local government areas, so if all that is done, will you all take the vaccine

All: yes

3: if it is 2000naira ((chorus answer)) from 5000naira down, we will pay it

1: ((inaudible))

M: does any other person have anything to add? If there is nothing else, that will be all. Thank you so much for your time
